# Preparation of Simple Bicyclic Carboxylate-Rich Alicyclic Molecules for the Investigation of Dissolved Organic Matter

**DOI:** 10.1021/acs.est.4c00166

**Published:** 2024-04-12

**Authors:** Alexander J. Craig, Lindon W. K. Moodie, Jeffrey A. Hawkes

**Affiliations:** † Analytical Chemistry, Department of Chemistry BMC, Uppsala University, Uppsala 752 37, Sweden; ‡ Drug Design and Discovery, Department of Medicinal Chemistry, Uppsala University, Uppsala 752 37, Sweden

**Keywords:** dissolved organic matter, carboxylate-rich alicyclic molecules, synthesis, mass spectrometry, nuclear magnetic resonance, Diels−Alder reaction

## Abstract

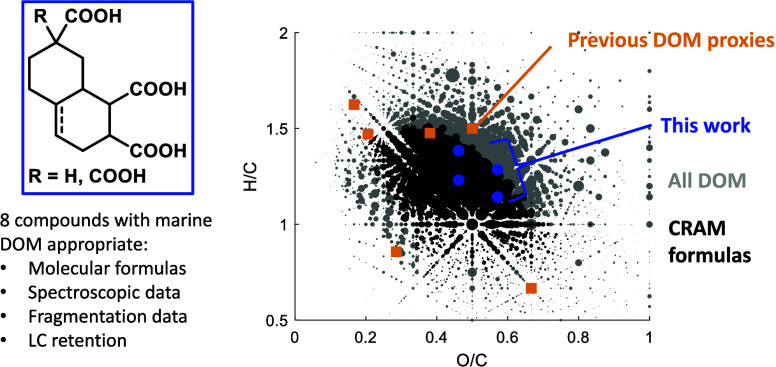

Dissolved organic matter (DOM) is a vast and complex chemical mixture that plays a key role in the mediation of the global carbon cycle. Fundamental understanding of the source and fate of oceanic organic matter is obscured due to poor definition of the key molecular contributors to DOM, which limits accurate sample analysis and prediction of the Earth’s carbon cycle. Previous work has attempted to define the components of the DOM through a variety of chromatographic and spectral techniques. However, modern preparative and analytical methods have not isolated or unambiguously identified molecules from DOM. Therefore, previously proposed structures are based solely on the mixture’s aggregate properties and do not accurately describe any true individual molecular component. In addition to this, there is a lack of appropriate analogues of the individual chemical classes within DOM, limiting the scope of experiments that probe the physical, chemical, and biological contributions from each class. To address these problems, we synthesized a series of analogues of carboxylate-rich alicyclic molecules (CRAM), a molecular class hypothesized to exist as a major contributor to DOM. Key analytical features of the synthetic CRAMs were consistent with marine DOM, supporting their suitability as chemical substitutes for CRAM. This new approach provides access to a molecular toolkit that will enable previously inaccessible experiments to test many unproven hypotheses surrounding the ever-enigmatic DOM.

## Abstract 2

In this study, we have synthesized and analyzed bicyclic tri- and tetra-carboxylic acid analogues of “carboxylate-rich alicyclic molecules”, a class of compounds proposed to be a key component of recalcitrant dissolved organic matter in aquatic environments. We compare and contrast their chromatographic and spectral data with a naturally occurring DOM reference material to confirm their suitability for future experimentation.

## Introduction

Dissolved organic matter (DOM) is a vast aquatic pool of reduced carbon that interfaces with both atmospheric greenhouse gases and stored sediments. The total carbon content of marine DOM is estimated at 662 Gt,^1^ exceeding preindustrial atmospheric carbon abundance in CO_2_. DOM’s role as a long-term carbon pool takes place predominantly in the ocean, with less than 1 Gt of DOM found in inland waters.^2^ In the marine environment, the majority of DOM is produced in the so-called “microbial loop” by phytoplankton, exists for only hours or days, and acts as a vector for the movement of nutrients between phytoplankton and heterotrophic bacteria.^3^ However, while this labile portion accounts for 84% of DOM produced in the ocean, it contributes only a small fraction to the marine DOM pool.^4^ More than 95% of DOM (ca. 630 Gt) exists on a millennia-long time scale with a half-life time of 4000–6000 years and appears to have very low chemical reactivity within a marine setting, leading to its designation as recalcitrant or refractory DOM (RDOM).^1^

With reduced carbon typically acting as a limiting substrate for microbiological growth,^5,6^ RDOM’s apparent stability appears somewhat paradoxical in the context of biogeochemistry.^7^ Historically, this has been resolved by attributing intrinsic chemical stability to the molecular contributors of RDOM.^8^ In recent years, however, the dilution hypothesis has emerged as a competing theory,^9^ proposing that any advantage an organism may gain from metabolizing these molecules is outweighed by the cost of collecting and processing them. While these theories offer compelling explanations for the stability of RDOM, they are system-level hypotheses that are not yet experimentally proven. Unfortunately, investigations tailored to probe RDOM’s apparent stability are difficult to design due to DOM’s extreme structural diversity, which limits the accurate quantification and comparison of components between samples or during experiments.

The application of mass spectrometry^10−12^ (MS) coupled with high-pressure liquid chromatography^13,14^ (HPLC) and ion mobility^15,16^ has indicated that DOM mixtures contain, at minimum, hundreds of thousands of individual molecular structures. Attempts to define the structural boundaries of these components have primarily used electrospray ionization MS (ESI-MS), tandem MS (MS2), and nuclear magnetic resonance (NMR) spectroscopy. However, the structural diversity of DOM places limits on the resolution of these techniques, such that any output highlights broad and prominent trends, rather than providing any level of specific molecular definition.^17^ For example, while partial separation of DOM by size exclusion chromatography and subsequent MS and abundance analysis shows that higher molecular weight molecules tend to be more hydrophobic,^18^ the molecular formulas present at all retention times are practically identical, even if in different ratios. Furthermore, current MS techniques have no means to delineate structural isomerism or differences in the ionization potential between molecules. MS2 and NMR suffer from similar problems, but through careful comparison of these somewhat orthogonal techniques, preliminary and idealized structural contributors to RDOM have been proposed.^19−21^

**Figure 1:**
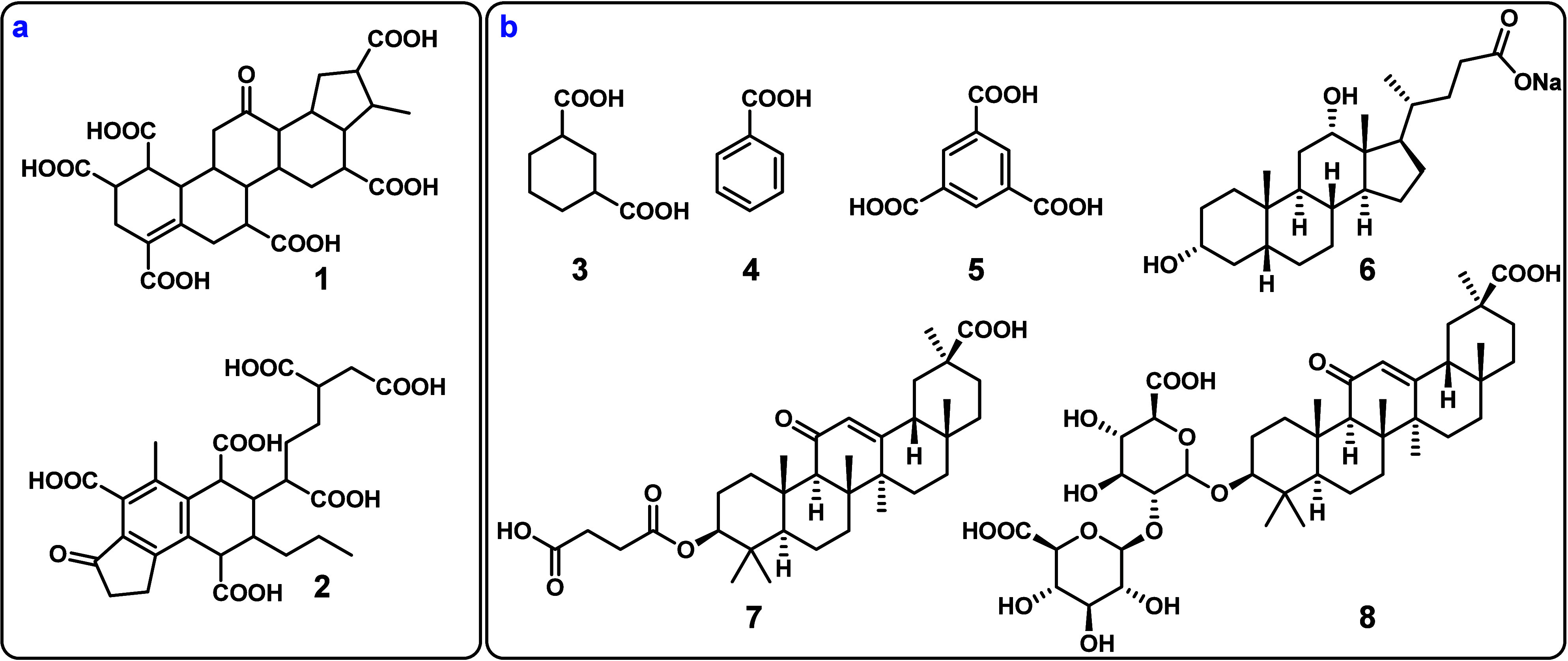
Idealized CRAMs proposed by Hertkorn et al. (a),^19^ and previously used substitutes for carboxylate-rich DOM (b).

**Figure 2:**
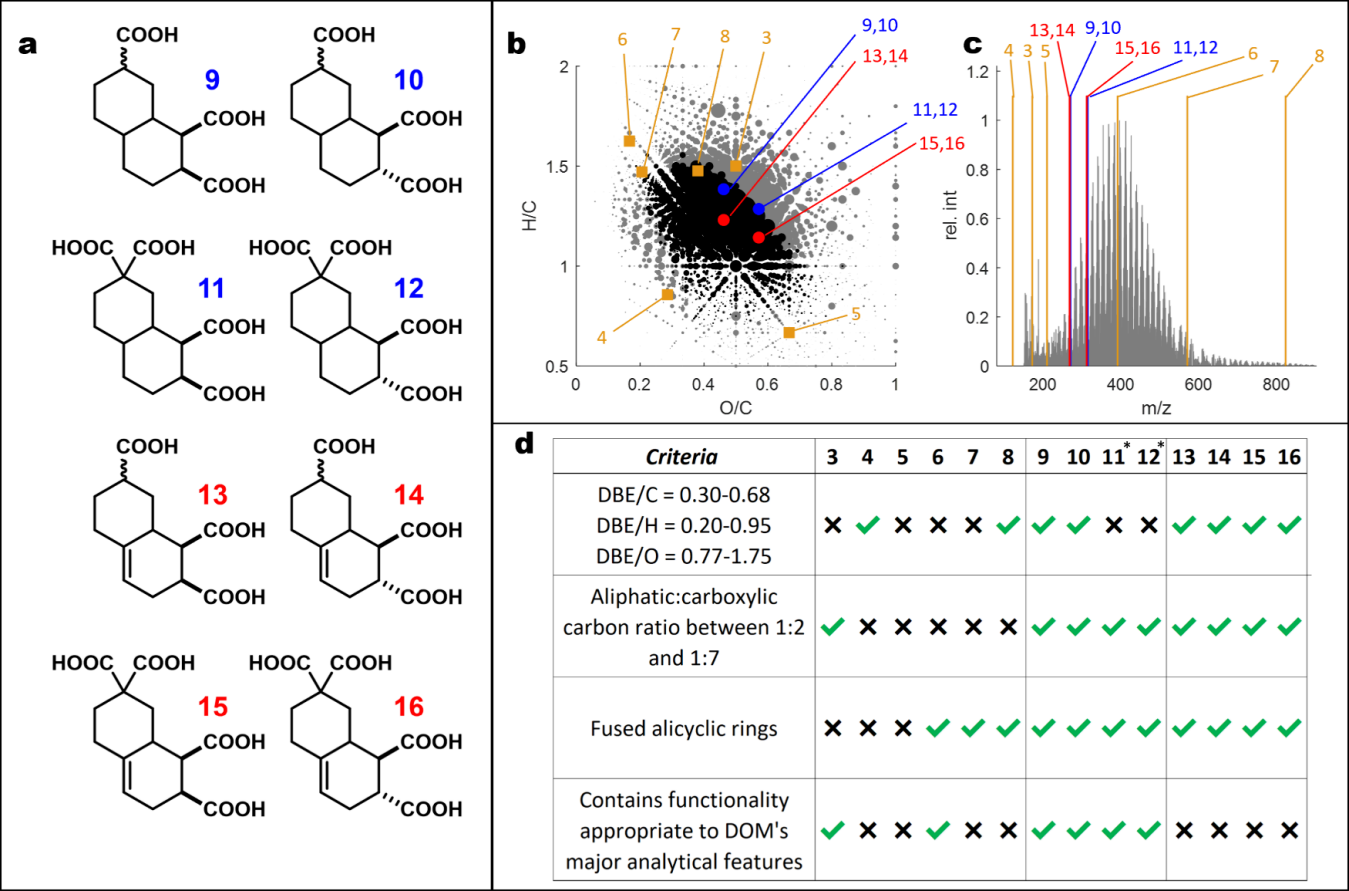
(a) Synthetic CRAM analogues **9**–**16** made for this work. (b) van Krevelen diagram of TRM-0522; black formulas represent CRAM, orange squares represent previously used molecular substitutes for DOM **3**–**8**, blue circles represent CRAM analogues **9**–**12**, and red circles represent CRAM analogues **13**–**16**. (c) MS spectrum of TRM-0522; orange lines represent previously used molecular substitutes for DOM **3**–**8**, blue lines represent compounds **9**–**12**, and red lines represent compounds **13**–**16**. (d) Table detailing appropriate parameters for previously used substitutes **3**–**8** and synthetic CRAM analogues **9**–**16**; *compounds **11** and **12** have DBE/O ratios of 0.75 but are otherwise consistent.

Carboxylate-rich alicyclic molecules (CRAM) are a class of compounds hypothesized to be present within DOM (Figure 1a, **1**, **2**).^19,22,23^ Originally proposed to explain signature regions in the NMR and MS data of DOM, CRAM was at first speculated to comprise up to 8% of the Earth’s total DOM pool, but this number is as of yet poorly constrained. Since their proposal, CRAM formulas have been detected in complex environmental samples from all types of ecosystem and are prominent in both freshwater and marine environments,^24,25^ indicating that they may have diverse biogeochemical sources. If these conceptual CRAM molecules do in fact represent such a large quantity of material within DOM, then they must also be responsible for a sizable portion of the properties of DOM. It might be expected that extensive work has been undertaken to obtain molecules that are representative of CRAM, so that its analytical, physical, chemical, and biological properties can be better investigated. However, upon examination of studies that sought to test the nature of DOM, it became immediately clear that appropriate control experiments with representative small molecules are almost entirely absent. In the case where substitutes are used, they are usually inadequate substitutes for the known molecular features of DOM. These include the similarly poorly defined lignin^26^ and humic acids,^26^ or small molecules (Figure 1b) such as cyclohexane 1,3-dicarboxylic acid **3**,^18^ benzoic acid **4**,^26^ benzene tricarboxylic acids such as **5**,^27^ sodium deoxycholate **6**,^26^ carbenoxolone **7**,^18^ or glycyrrhizic acid **8**.^18^

While all of these substitutes do indeed contain predominantly C, H, and O atoms and some number of carboxylic acids, they do not satisfy many definitions of DOM and theoretical CRAM. DOM that has been processed by solid-phase extraction (SPE-DOM) is consistently found to contain molecules that generate singly charged ions from ranging from *m*/*z* 200 to 800, and “CRAM” formulas (with appropriate O:C and H:C ratios) are found throughout this range. Furthermore, Hertkorn et al. specified the requirements for CRAM to comprise fused alicyclic rings, multiple carboxylic acids, minimal other oxygen functionality, a lack of extended aromatic systems, and a ratio of carboxylate carbon:aliphatic carbon atoms between 1:7 and 1:2. While Hertkorn defined CRAM precisely, the concept of CRAM is still abstract, and the definitions could be revised in the future.

One might assume that turning to synthetic or natural products chemistry could offer better options for substitutes for CRAM. However, while there are some procedures that detail the preparation or isolation of small-molecule organic polyacids, the majority of these compounds fall outside of the structural boundaries of DOM and CRAM.^28,29^ Furthermore, thorough examination of the structures available on chemical databases such as SciFinder highlights another crucial problem; a dwindling minority of these compounds come with reported NMR or MS data (see the Supporting Information for Chemical Database Experiments).

To address the lack of reliable substitutes for CRAM, we prepared a series of synthetic CRAM molecules for comparison to the natural data. We believe that the preparation of such compounds will enable future experimentation targeted toward mechanistic understanding of the generation and fate of RDOM. Furthermore, we suggest that the iterative synthesis of hypothetical molecular components of DOM will inevitably lead to more accurate representation of the structures that truly exist within it. Finally, we propose that the development of methods for the preparation of accurate RDOM standards will lead to improvements for the quantification of DOM globally. As a proof of concept, a series of four CRAM molecules (Figure 2, **9**–**12**) and their partially oxidized counterparts were prepared (Figure 2, **13**–**16**), and their NMR, MS, MS2, and HPLC spectra were directly compared with representative data sets from the marine DOM standard TRM-0522.^30^ TRM-0522 was chosen for comparison, as it is low in aromatic content and is the only standard mixture available from a marine source containing DOM that is recalcitrant in this environment.

## Methods

### Reference Materials

The reference standard TRM-0522^30^ was used as received and dissolved in 5% acetonitrile to a concentration of 2 mg/mL for injection by LCMS. TRM-0522 was isolated from a coastal setting at 45 m depth on the west coast of Sweden using an aqueous-compatible C_18_ sorbent. It is a well-defined reference mixture^30^ containing thousands of molecular formulas, the majority of which are defined as “CRAM” according to the definitions in Hertkorn et al.’s work.^19^

### Liquid Chromatography

Liquid chromatography was conducted with a Thermo Vanquish UPLC using a Phenomenex Kinetex C18 column (2.1 × 150 mm, 1.7 μm) at a flow rate of 0.4 mL/min. Mobile phase A was 0.1% formic acid (AnalaR NORMAPUR, VWR) in deionized grade water (Millipore Milli-Q), and B was 0.1% formic acid in LCMS grade acetonitrile (Lichrosolv, Supelco, Merck). A linear gradient started at 5% B and then increased at 1 min from 5 to 95% B at 10 min, followed by a 1 min washout phase at 95% B, a decrease to 5% B, and a 3.9 min equilibration phase at 5% B (method length = 15 min). The column oven was set to 50 °C to decrease back pressure.

### MS

MS was conducted with an Orbitrap Q Exactive instrument (Thermo Fisher). Electrospray ionization was used as the ionization source by using a heated unit running at 150 °C and −2.5 kV (negative mode). Sheath gas and auxiliary gas were set to 15 and 5 units, respectively, S-Lens was set to 60, and capillary temperature was set to 200 °C. For the experiments, resolution was set to 70,000 and a maximum injection time of 200 ms was chosen, aiming to trap 3 × 10^6^ ions in the Orbitrap. MS2 experiments were conducted at normalized collision energies of 35 and 75 by higher energy collision dissociation (HCD) experiments using the PRM method, and 2 × 10^5^ ions were targeted using automatic gain control for these experiments with a maximum trapping time of 100 ms. An isolation window of 1 Da was selected, centered on the deprotonated mass of the compound being investigated (i.e., 267.1, 269.1, 311.1, or 313.1 Da).

### NMR of CRAM Analogues **9**–**16**

NMR spectra for analogues **9**–**16** were acquired at 298 K at 600 MHz on a Bruker Avance Neo spectrometer with a TCI (CRPHe TR-^1^H &^19^F/^13^C/^15^N 5 mm-EZ) probe. The samples were dissolved in 0.1 N NaOD in D_2_O prepared by adding 23 mg of Na (>99.8% sodium basis, Sigma-Aldrich, Merck) to 10 mL of D_2_O (deuteration degree min 99.9%, MagniSolv, Sigma-Aldrich, Merck) and chemical shifts referenced to the residual solvent peak at 4.79 ppm in the ^1^H NMR spectrum, and approximately 5 μL of methanol (min 99.9%, Chromasolv, Honeywell) was added for referencing purposes in ^13^C NMR experiments. ^13^C NMR spectra were recorded in 0.1 N NaOD in D_2_O prepared by adding 23 mg of Na (>99.8% sodium basis, Sigma-Aldrich, Merck) to 10 mL of D_2_O (deuteration degree min 99.9%, MagniSolv, Sigma-Aldrich, Merck), and approximately 5 μL of methanol (min 99.9%, Chromasolv, Honeywell) was added, so that the samples could be referenced to the residual methanol solvent peak at 49.50 ppm. ^1^H NMR, ^13^C NMR, COSY, HSQC, and HMBC spectra for CRAM proxies **9**–**16** are provided in Figures S6–S53.

## Results and Discussion

### Design and Synthesis of CRAM Analogues **9**–**16**

In designing appropriate CRAM analogues, the foremost priority was placed on synthetic accessibility. While one might be drawn toward the generation of larger molecules such as Hertkorn’s et al.’s idealized CRAMs **1** and **2** (Figure 1), this would require a total synthetic style approach. The total synthesis of larger molecules can take years or even decades, and significant risk would be assumed in regard to the accuracy of such a target. Furthermore, the generation of large and specific structures within the total synthetic framework frequently leads to highly specific syntheses that do not allow for reliable addition, removal, or modification of functional groups. Instead, we wanted to develop a toolbox with the potential to access a wide range of possible CRAM analogues.

**Scheme 1:**
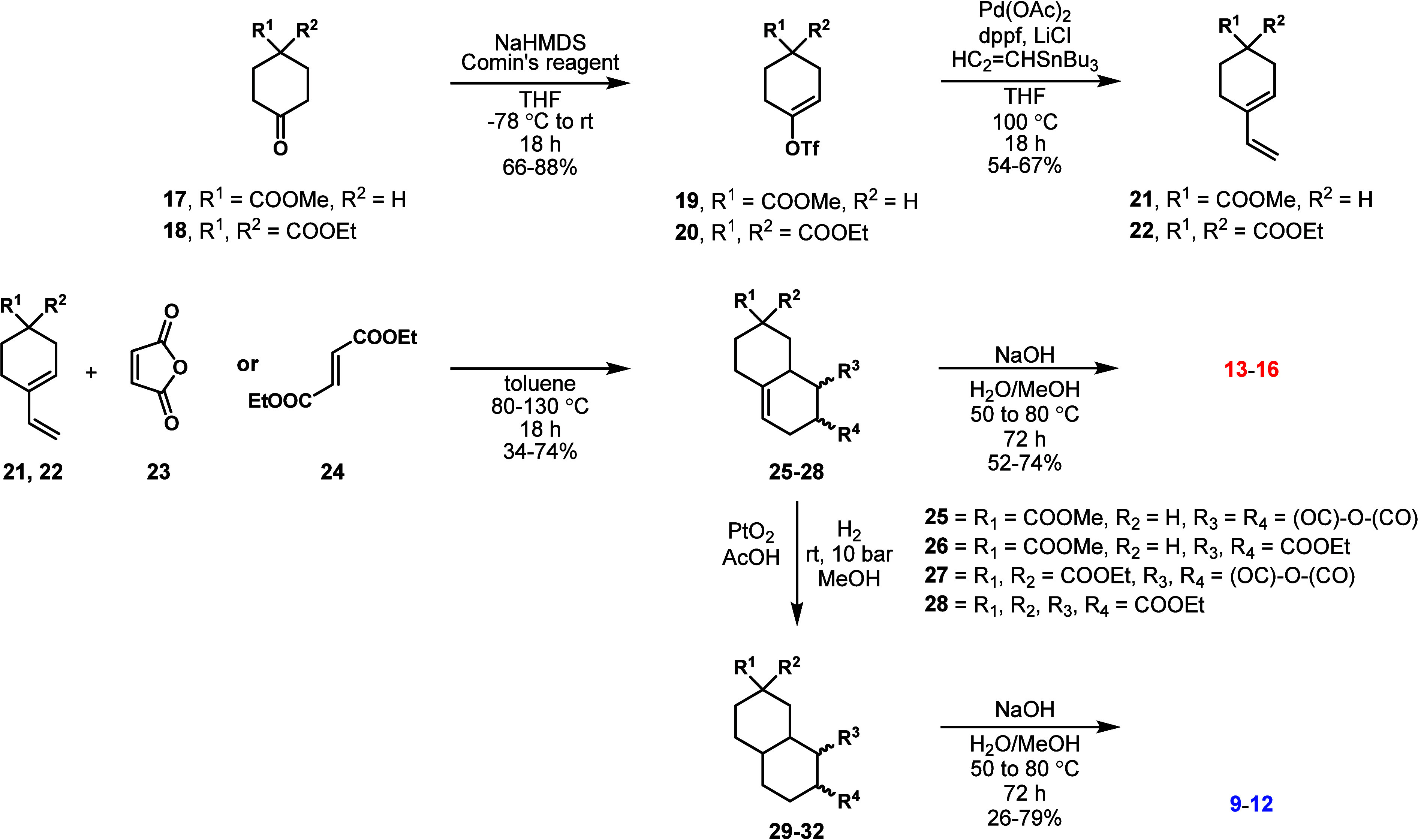
Synthetic Route to CRAM Analogues **9**–**16**

Thus, we approximated what the most basic requirements for a CRAM were. Using the guidelines of Hertkorn et al., these compounds should contain fused (i.e., multiple) alicyclic rings and multiple carboxylic acids. Compounds with three or four carboxylic acids were targeted, both to account for the alicyclic:carboxyl carbon ratios of CRAM (Figure 2) and the sequential neutral losses of CO_2_ seen in MS2 experiments on SPE-DOM. While another functionality was hypothesized to exist in the structures of CRAM by Hertkorn et al., their NMR data, and that of other marine DOM samples such as TRM-0522, shows that ketone, alkene, and aromatic functionalities are minor or trace contributors to RDOM. Finally, molecules at the lower end of the SPE-DOM *m*/*z* range of 200–800 were targeted. Given these requirements, structures with two rings and three or four acids were seen as suitable initial candidates. It was envisioned that the use of appropriate semicyclic dienes (Scheme 1, **23** and **24**) and dienophiles (Scheme 1, **25** and **26**) in Diels–Alder reactions would allow for the rapid preparation of these types of bicyclic carbon scaffolds.^31^

The preparation of CRAMs **9**–**16** began with the reaction of cyclic ketones **17** or **18** to form vinyl triflates **19** or **20** in good yield. Subsequent coupling between vinyl triflates **19** and **20** with vinyl tributyl tin provided diene products **21** or **22** in moderate yields (see the SI for a detailed discussion). Following this, semicyclic dienes **21** or **22** were reacted with dienophiles **23** or **24** to provide Diels–Alder adducts **25**–**28** in moderate to good yields as complex diastereomeric mixtures. We were not able to separate these diastereomers on a preparative scale, and as such, the decision was made to continue the synthesis using these diastereomeric mixtures. Next, alkenes **25**–**28** were hydrogenated to afford the corresponding alkanes **29**–**32** (see the SI for a detailed discussion). Finally, alkanes **29**–**32** and alkenes **25**–**28** were subjected to hydrolysis under basic conditions to provide CRAMs **9**–**16** (see the SI for a detailed discussion). We were unable to separate the resulting diastereomers using in-house purification methods, and they were used for further analysis as their mixtures. With CRAM’s **9**–**16** in hand, we next looked to investigate their properties using LCMS, MS, and NMR spectroscopy.

### Retention Time Comparison

**Figure 3:**
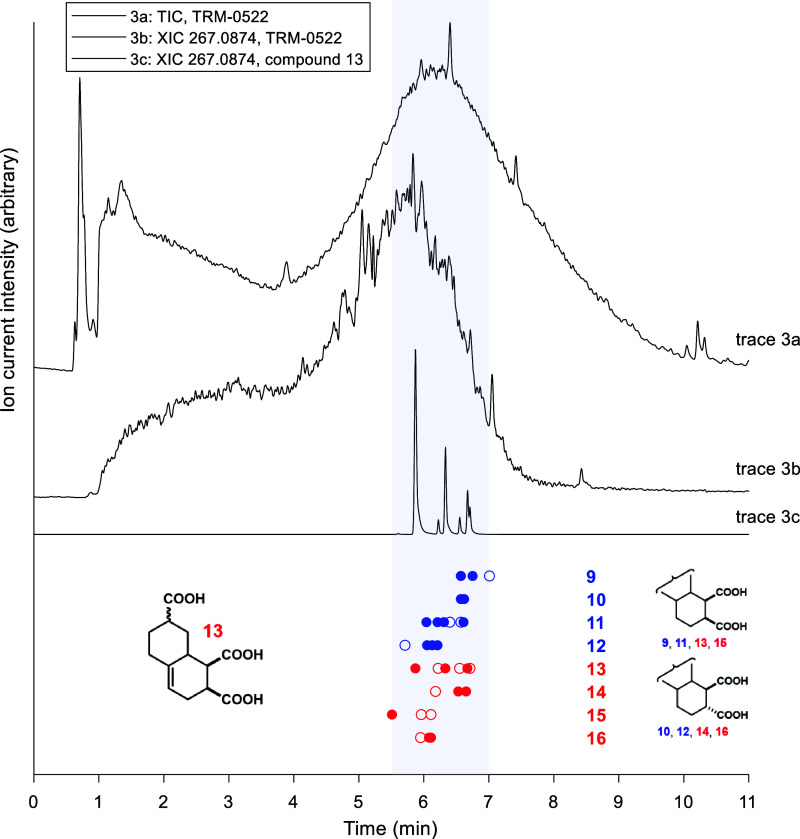
Trace a: total ion chromatogram of TRM-0522 (a natural DOM standard), with nine-point smoothing. Trace b: extracted ion chromatogram of TRM-0522 for 267.0874 *m*/*z* (C_13_H_15_O_6_^–^, molecular ion of compound **13**), with nine-point smoothing. Trace c: extracted ion chromatogram of compound **13**. Retention times for the isomers of synthetic CRAM alkanes **9**–**12** in blue, and alkenes **13**–**16** in red. The major isomers are filled in circles, while the minor isomers are shown as outlines. The relative stereochemistry at the 1,2-diacid position for compounds **9**–**16** is shown at the bottom right.

Compounds **9**–**16** were analyzed via reverse-phase HPLC (see Methods-LC), and the measured elution times of their isomers spanned almost 30% of the main DOM peak of TRM-0522 (trace 3a, Figure 3). Furthermore, at the same retention times for all diastereomers of the synthesized compounds, the same molecular formulas are observed as prominent peaks within the MS data of the natural mixture Thus, the polarity and molecular formulas of these synthetic CRAMs correspond well with the compounds found in natural DOM. Specifically comparing compound **13** (trace 3c, Figure 3) with the natural data, its six different diastereomers eluted across roughly 20% of the major DOM peak, indicating that only stereochemical differences could be an important and potentially overlooked factor contributing to retention time diversity of the natural compounds within DOM. The major and minor isomeric peaks observed for compound **13** are also shown alongside the extracted ion current for the same formula (C_13_H_15_O_6_^–^) in TRM-0522 (trace 3b, Figure 3). Here, they elute toward the end of the main peak for this formula, such that they are a good retention time match for the most prominent hydrophobic isomers with this molecular formula in marine DOM.

In general, the tetra-acids **11**, **12**, **15**, and **16** eluted earlier than the triacids **9**, **10**, **13**, and **14**. This is perhaps expected given the polarity typically attributed to an individual carboxylic acid moiety. However, it is somewhat surprising to see that this is not universally true. For example, the major diastereomer of compound **13** elutes earlier than the peaks seen for compound **16**. Importantly, these compounds bear different stereochemistry at the 1,2-diacid functionality, with both of these acids existing on the same face for **13**, and on opposite faces for **16** (Figure 3). This is consistent with the differences in retention time seen between the major isomers of compound **15** and compound **16**. Thus, we speculate that the presence of more acids on a single face of one of these CRAMs typically increases the overall polarity of that molecule. Possible explanations for this include the greater dipole moment of compounds with this configuration (i.e., earliest eluting diastereomer of compound **13** vs **16**), inter- or intramolecular hydrogen bonding effects, or trace-metal complexation.

### MS2 Analysis

**Figure 4:**
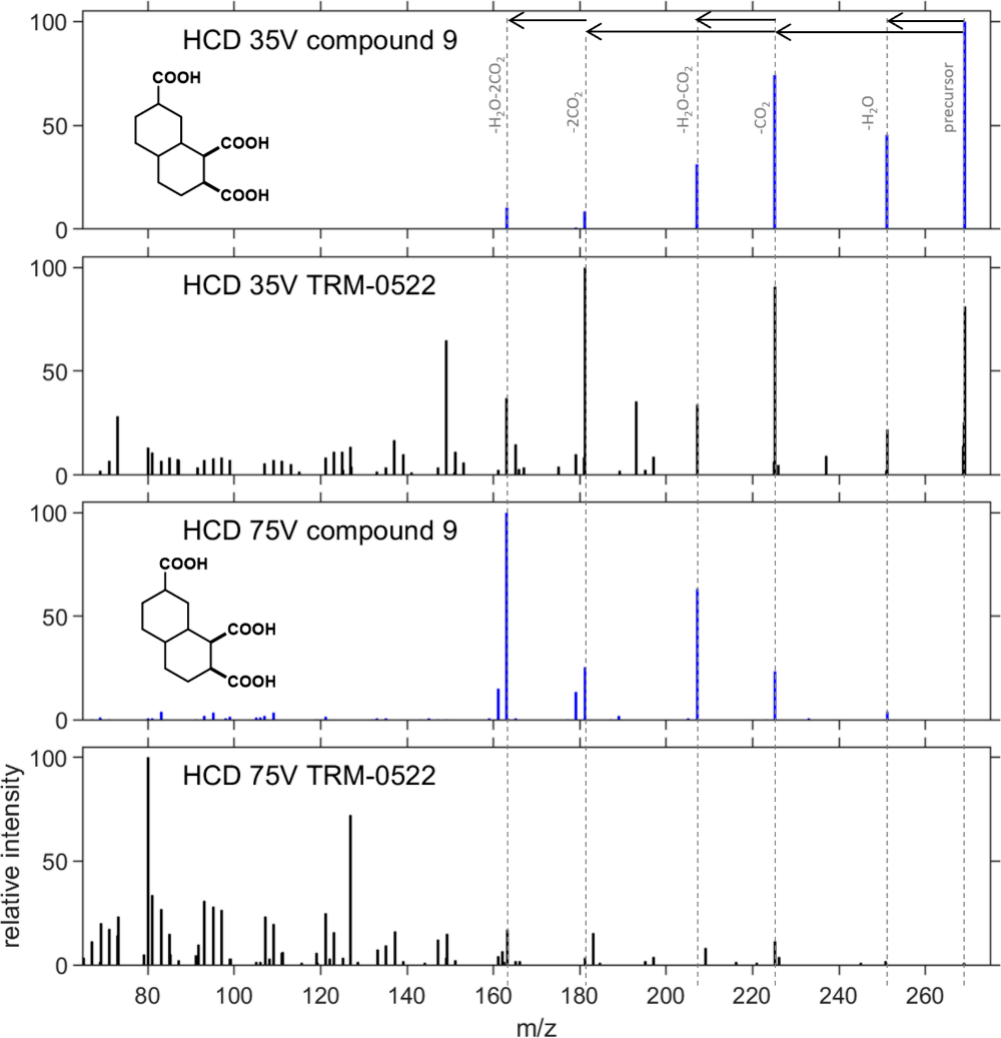
35 and 75 V HCD fragment spectra of compound **9** (blue, selected as a representative compound of the eight synthesized molecules) and the same mass (*m*/*z* 269) in coastal marine reference TRM-0522 (black). The data were extracted from QualBrowser and replotted in MATLAB. Note that the data are collected at high resolution in the Orbitrap after fragmentation in a low-resolution ion trap. This means that for TRM, multiple precursors are trapped and fragmented and most fragment masses also have multiple peaks, which are not visible on this scale.

Higher-energy collisional dissociation (HCD) was used at two different voltages to probe the fragmentation of the CRAM analogues. Generally, synthetic CRAMs **9**–**16** showed sequential losses of carbon dioxide and water at both 35 V and 75 V normalized collision energy (NCE), consistent with the most prominent trends seen in the MS2 fragmentation of natural DOM.^11,12^ Representative data gathered for compound **9**, as well as comparative data gathered from TRM-0522 at the same retention time, is shown in Figure 4.

In the 35 V HCD data, the most prominent ions derived from compound **9** are observed at 269 (parent ion), 251 (−H_2_O), 225 (−CO_2_), 207 (−H_2_O and CO_2_), 181 (−2CO_2_), and 163 (−2CO_2_, and H_2_O) *m*/*z*. Low-energy HCD analysis of TRM-0522 revealed similar fragmentation patterns, with three additional unidentified major peaks observed, occurring at *m*/*z* 149, 193, and 237, as well as many additional lower intensity peaks. It is important to note that using an ion trap or quadrupole for fragmentation prior to high-resolution analysis only allows isolation of a unit mass, not a single molecular formula,^11,32^ and as such, it is difficult to trace whether the origin of these peaks is from natural compounds with the same molecular formula as CRAM analogue **9**. We also noted the intensity of the second neutral CO_2_ loss, and subsequent water loss is somewhat higher for the natural mixture than for compound **9**. While the data are not directly comparable due to the resolution of the fragmentation trapping, the presence of common fragmentation peaks suggests that there is a high correspondence in MS labile functionalities between the synthetic CRAMs and natural DOM.

Within the 75 V HCD data, additional fragmentations of compound **9** are seen, with new major peaks observed at *m*/*z* 179 (−CO_2_, H_2_O, and CO) and 161 (−CO_2_, H_2_O × 2, and CO) *m*/*z,* and trace peaks observed between *m*/*z* 80 and 130. For TRM-0522, all corresponding major peaks from compound **9** are only seen at trace intensities, with the exception of peaks at *m*/*z* 163 and 161, which are seen as minor contributors, as well as complete loss of the peaks at *m*/*z* 251 (−H_2_O) and 207 (−H_2_O and CO_2_). Critically, in the natural mixture, there are many fragment masses below an *m*/*z* of 161, which are only seen in low or trace intensities in the high-energy HCD data from compound **9**.

Alkenes **13**–**16** showed similar intensities for neutral CO_2_ and H_2_O losses under high-energy fragmentation. However, they also showed greatly increased backbone decomposition, indicating that the presence of the alkene allowed for more diverse charge stabilization and thus more varied fragmentation pathways. Similarly, extensive fragmentation of the carbon scaffold was observed at the same retention times within the same experiments for isomers in the natural DOM sample, with greatly diminished intensities of neutral CO_2_ and H_2_O losses. As alkenes are at most a trace contributor to the NMR data of marine DOM, this likely indicates that for the majority of natural CRAM-like molecular formulas, extensive fragmentation is dependent on those molecules bearing other oxygen functionalities, alkyl branching, or carbon-backbone unsaturations that allow for stabilization of a negative charge. The isolation of a single unit mass within these experiments leads to fragment ions coming from multiple parent ions, ultimately precluding extensive investigation of natural DOM fragmentation pathways.^32,33^ Future work utilizing FTICR and SWIFT techniques alongside LC is a key direction for the comparison of single molecular formulas from DOM with these synthetic CRAMs.^11^

### Nuclear Magnetic Resonance Comparison

**Figure 5:**
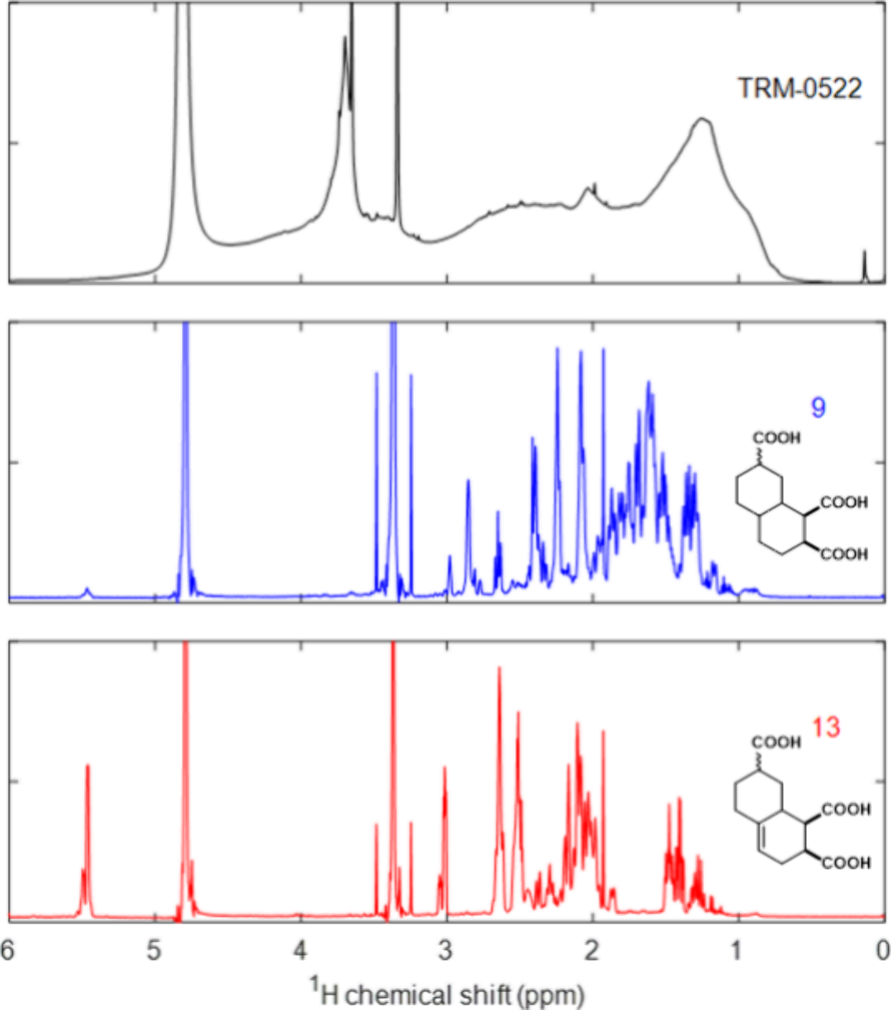
^1^H NMR data of TRM-0522 and synthetic CRAM compounds **9** and **13**. The residual D_2_O solvent peak is observed at 4.79 ppm, and MeOH added to reference the signal for ^13^C NMR is observed at 3.34 ppm.

^1^H, ^13^C, COSY, HSQC, HMBC, and NOESY spectra were recorded for compounds **9**–**16** (see the Supporting Information). Within the ^1^H NMR spectra of alkanes **9**–**12** (compound **9** shown in Figure 5), major resonances were observed between 2.0 and 3.0 ppm and correspond to hydrogen atoms either α to a carboxylic acid or at a bridgehead position β to a carboxylic acid. The remainder of signals corresponded to hydrogen atoms not at bridgehead positions that were more than one carbon atom away from carboxylic acids and occurred between 0.9 and 2.2 ppm. Turning to alkenes **13**–**16** (compound **13** shown in Figure 5), hydrogen atoms either α- to a carboxylic acid or at a bridgehead position β- to a carboxylic acid occurred between 2.1 and 3.1 ppm. Whereas the majority of the peak integral attributable to other alkyl functionality was observed between 1.4 and 1.8 ppm for alkanes **9**–**12**, the majority of this integral for alkenes **13**–**16** was seen between 1.8 and 2.2 ppm. Additional alkyl resonances were observed between 1.2 and 1.6 ppm and were initially assumed to be methylene functionalities. However, careful analysis of the HSQC, NOESY, and coupling constant data of alkene **16** highlighted that these peaks corresponded to diastereotopic protons that were on the opposite face of a cyclohexyl ring relative to a neighboring acid functionality. Alkene shifts showed little variation, occurring entirely between 5.4 and 5.6 ppm. Finally, similar trends were observed between the alkenes and alkanes within the ^13^C NMR data, where peaks are seen in the carboxylate, CRAM, and alkyl regions for all compounds, and additional peaks at around 140 and 120 ppm were seen corresponding to alkene functionalities for compounds **13**–**16**.

Of note, little peak intensity was seen between 0.8 and 1.3 ppm for CRAM analogues **9**–**16**, a region indicative of methyl and methylene groups that are distant to any oxygen bearing functionality. While this peak is prominent in many environmental DOM samples (including TRM-0522), its relative absence is consistent with the difference NMR spectra presented by Hertkorn et al. that established the expected chemical shift ranges for CRAM.^19^

Generally, the peaks observed in the NMR data of these compounds are consistent with both natural DOM and with the CRAM-like data presented by Hertkorn et al.^19^ The presence of the alkene in compounds **13**–**16** is a major outlier; both Hertkorn et al.’s data and TRM-0522 have almost no integral in this region, and Hertkorn proposed that if alkenes exist in CRAMs, they are likely to be tetra-substituted and therefore would have no corresponding ^1^H NMR signal.^19^ However, given the extreme complexity of DOM, the relative insensitivity of NMR and the presence of corresponding molecular formulas at the correct LCMS retention times and masses, the existence of hydrogen-bearing olefins is possible in some capacity (vide infra). Thus, it is useful to note that in the presence of such an alkene, the remaining CRAM-like and aliphatic-like signals still fit within the natural DOM data.

### Outcomes

In the current research, we aimed to design, prepare, and analyze synthetic CRAM analogues that match key analytical features within natural DOM. LC analysis showed that CRAMs **9**–**16** fit well within the retention profile of DOM. For each compound, we typically saw a range of retention times across diastereomers; suggesting that stereochemistry may be a contributing factor to the broad diversity seen within DOM. MS2 analysis indicates that compounds containing only alicyclic rings and acids are plausible but minor contributors to the fragmentation patterns seen in natural DOM. However, the MS2 data also demonstrates that other functionalities are required to explain the exceptional variety seen in the “fingerprint” fragmentations of high-energy fragmentation methods, as these lower mass fragment ions were mostly absent from the synthesized compounds. Finally, NMR analysis shows that the chemical shifts of **9**–**16** generally fall within the previously defined CRAM and aliphatic ranges observed in DOM, even when alkene functionalities are present. While direct investigation of compounds **9**–**16** by FTICR/SWIFT is still required to validate whether structures like these are present in natural DOM, the many similarities between these compounds and the natural NMR, MS, and LC data of DOM highlight their potential in future experiments. Critically, CRAM analogues **9**–**16** provide more accurate functionality, elemental formulas, and carboxylate:alicyclic carbon ratios than previously investigated substitutes such as **3**–**8** (Figure 1, vide supra).

Generally, within the natural MS data of DOM, one of the most striking features is the presence of regular patterns in the data, indicating common mass differences running throughout the chemical structures present. Of note are the common mass differences that correspond to the switching of CH_4_ with O (36.4 mDa), an additional H_2_ (2.016 Da), or an additional CH_2_ (14.016 Da).^34^ Within the compounds prepared here, the inclusion of an alkene functionality between compounds **9**–**12** and **13**–**16** represents a single degree of unsaturation (i.e., −H_2_). The data show that such a replacement had only minor consequences for retention time, fragmentation patterns, and the majority of ^1^H NMR chemical shifts. The similarities between the two types of compounds within this work indicate that alkene functionalities are a possible contributor to 2.016 Da MS intervals in natural DOM while not disrupting other key features of its analytical data. This of course does not rule out other functionality accounting for these differences, such as ketones, additional ring junctions, or aromatic functionality. We envision that further development of synthetic methodology for the preparation of a structurally diverse library of compounds will allow for the more in-depth investigation of these common mass differences and provide molecules with features appropriate to other spectral features of CRAM. Molecular features that will be targeted in future include tetra-substituted olefins, aromatic ring systems, oxygen-containing functionalities, and modified carbocyclic scaffolds.

The preparation of CRAMs **9**–**16** represents the first steps toward a robust and general methodology for the preparation of CRAM analogues that align with the chromatographic and spectral properties of natural DOM. The generality of the Diels–Alder reaction allows for the use of interchangeable dienes and dienophiles, such that new compounds can be targeted and quickly generated with specific experimentation in mind. Further work is required to explore the functionalities present, relative functional group arrangements, and carbocyclic backbones of CRAM within DOM, as well as to investigate their stability within both physical and biological experiments. The acquisition of these CRAM compounds enables targeted biogeochemical work to test theories such as the dilution hypothesis using defined and realistic structures and highlights an exciting new research direction within DOM investigation, using synthetic chemistry to unlock previously inaccessible experiments.

## Supporting Information Available

General methods, NMR analysis information, experimental procedures and tabulated data, additional synthetic details and chemical database experiments, and spectra, including MS spectra, NMR spectra, chromatograms, and tabulated chromatography data (PDF)
