# Supplementary material for: Preparation of Simple Bicyclic Carboxylate-Rich Alicyclic Molecules for the Investigation of Dissolved Organic Matter
Source: Environ Sci Technol. 2024 Apr 12;58(16):7078–86. doi: 10.1021/acs.est.4c00166 (PMC11044592; doi:10.1021/acs.est.4c00166)
Supplement: Supplementary file 1 — es4c00166_si_001.pdf [file es4c00166_si_001.pdf]

Supporting Information – Experimental

Preparation of Simple Bicyclic Carboxylic Rich Alicyclic Molecules for the Investigation of Dissolved Organic Matter

Alexander J. Craig[1,2], Lindon W. K. Moodie[2], and Jeffrey A. Hawkes[1,*]

[1.] Analytical Chemistry, Department of Chemistry BMC, Uppsala University, Uppsala 752 37, Sweden
[2.] Drug Design and Discovery, Department of Medicinal Chemistry, Uppsala University, Uppsala 752 37, Sweden
*Corresponding author: jeffrey.hawkes@kemi.uu.se

Summary: 91 pages, 74 figures, 8 tables

Contents:

Abbreviations	**S2**
Synthetic Experimental Details	**S3-S12**
Additional Synthetic Information	**S13**
Chemical Database Experiments	**S14-S16**
LCMS traces for compounds **9-16**	**S17**
HCD traces for compounds **9-16**	**S18-S21**
^{1}H, ^{13}C, COSY, HSQC, and HMBC NMR spectra, and TIC/CID traces for compounds **9-16**	**S22-S82**
^{1}H NMR spectra for known compounds **19-22**	**S83-S84**
^{1}H NMR and ^{13}C NMR spectra for novel compounds **23-32**	**S85-S90**
Bibliography	**S91**

Abbreviations List

app t	Apparent triplet
Bpin	Boron pinacolate
CAD	Charged aerosol detection
COSY	Correlation spectroscopy
CRAM	Carboxylate rich alicyclic molecule
d	Doublet
dd	Doublet of doublets
dt	Doublet of triplets
ESI	Electrospray ionization
Et_2O	Diethyl ether
EtOAc	Ethyl acetate
HCD	Higher energy collisional dissociation
HMBC	Heteronuclear multiple bond correlation
HSQC	Heteronuclear single quantum correlation
HRMS	High-resolution mass spectrometry
LCMS	Liquid chromatography mass spectrometry
m	Multiplet
MeOD	Deuterated methanol
NaHMDS	Sodium hexamethyldisilazane
NMR	Nuclear magnetic resonance
PET	Petroleum ether
s	Singlet
t	Triplet
THF	Tetrahydrofuran
TIC	Total ion chromatogram
TLC	Thin-layer chromatography
UV	Ultraviolet

General Methods

Thin-layer chromatography (TLC) was performed on 0.2 mm aluminium plates precoated with silica gel 60 F_{254} (Merck). Compounds were visualized with an ultraviolet (UV)-light, and stained with potassium permanganate. Column chromatography was performed with silica gel (40 – 63 μM). High resolution mass-spectra to obtain accurate masses of intermediate and final compounds were recorded on a LTQ Velos Pro Orbitrap (Thermo Fisher), with samples being analysed by direct infusion with electrospray ionization in negative mode (final compounds) or by liquid chromatography mass spectrometry (LCMS) with electrospray ionisation in positive or negative mode (intermediates). Liquid chromatography was conducted at a flow rate of 350 µl/min on C18 (Phenomenex Kinetex 150x2.1 mm, 1.7 µm pore size), in a linear gradient running from 5-95% mobile phase B, where A was 0.1% formic acid in deionised water (milliQ, Millipore), and B was acetonitrile (LiChrosolv, Merck) with 0.1% formic acid. Data were collected at 100,000 resolution (at *m/z* 400) and 50 transients were co-added for direct infusion data. ^{1}H NMR spectra were recorded at 400 MHz on a Varian Mercury Plus spectrometer, or at 600 MHz on a Bruker Avance Neo spectrometer with a TCI (CRPHe TR-1H &19F/13C/15N 5mm-EZ) probe. All spectra were recorded from samples in either $CDCl_3$ or MeOD, at room temperature in 5 mm nuclear magnetic resonance (NMR) tubes. Chemical shifts are reported relative to the residual solvent peak at δ 7.26 for $CDCl_3$ or δ 3.31 for MeOD. Resonances were assigned as follows: chemical shift (multiplicity, number of protons, coupling constant(s)). Multiplicity abbreviations are reported by the conventions: s (singlet), d (doublet), dd (doublet of doublets), dt (doublet of triplets), t (triplet), app t (apparent triplet), m (multiplet). Proton decoupled ^{13}C NMR spectra were recorded at 101 MHz on a Varian Mercury Plus spectrometer under the same conditions as for the ^{1}H NMR spectra, or at 600 MHz on a Bruker Avance Neo spectrometer with a TCI (CRPHe TR-1H &19F/13C/15N 5mm-EZ) probe under the same conditions as for the ^{1}H NMR spectra. Chemical shifts have reported relative to the residual solvent peak at δ 77.16 for $CDCl_3$ or δ 49.90 for MeOD. All solvents and reagents were used as received. Compound purities are reported based on integrated peak areas from charged aerosol detection (CAD) data using a Thermo Vanquish UPLC coupled CAD analyser with the gradient LCMS method as reported in the main text (see supporting information spectral file for compound purities).

NMR Analysis Information

The final carboxylate rich alicyclic molecule (CRAM) compounds **9**-**16** were complicated mixtures with major, minor, and trace diastereomeric peaks. For practical data reporting purposes, ^{1}H NMR integrals and peaks are largely arbitrary, aiming to define the amount of integral across a region between two relative minima, instead of integration occurring across every peak that represents a single chemical environment. Furthermore, a consistent but arbitrary manual threshold was used for ^{13}C NMR peak picking, such that anyone wishing to replicate this work can cross reference their own spectra for the highest intensity carbon NMR signals. Similarly, we are aware that different fields may be interested in using this data in different ways. Thus, for all displayed NMR data of final CRAM compounds **9**-**16**, a series of spectral windows are shown for each type of NMR experiment. NMR spectral data for all compounds can be found in the additional spectral data supplementary information file.

^{1}H NMR spectra of each compound are reported with: a broad spectral window (0–10 ppm), and a spectrum highlighting the peaks found within both the CRAM-like (ca. 2.0–3.0 ppm) and aliphatic regions (ca. 1.0–2.0 ppm).

^{13}C NMR spectra of each compound are reported with: a broad spectral window (0–200 ppm), a spectrum highlighting the carboxylate region (ca. 170–185 ppm), and a spectrum highlighting the sugar, CRAM, and aliphatic regions (ca. 20–60 ppm).

For correlation spectroscopy (COSY) spectra, the first spectrum shows the ^{1}H NMR range from 0-10 ppm, and the second shows CRAM-like and aliphatic signals. For heteronuclear single quantum correlation (HSQC) spectra, the first spectrum shows the ^{1}H NMR range from 0-10 ppm, and the ^{13}C NMR range from 0-160 ppm, while the second spectrum shows CRAM-like and aliphatic signals. For heteronuclear multiple bond correlation (HMBC) spectra, the first spectrum shows the ^{1}H NMR range from 0-10 ppm, and the ^{13}C NMR range from 0-200 ppm, the second spectrum shows carboxylate to CRAM-like and aliphatic signals, and the third spectrum shows CRAM-like and aliphatic signals.

Triflate **19**[1]

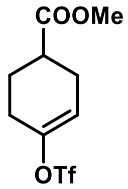

To a dry flask containing methyl 4-oxocyclohexane-1-carboxylate (4.6 mL, 5.0 g, 0.032 mol, 1 eq.) and *N*-(5-chloropyridin-2-yl)-*N*-(methanesulfonyl)methanesulfonamide (Comin's reagent, 13.8 g, 0.0352 mol, 1.1 eq.) was added dry tetrahydrofuran (THF) (100 mL) under a nitrogen atmosphere. The mixture was cooled to −78 °C, before 1M sodium hexamethyldisilazane (NaHMDS) in THF (35.2 mL, 0.0352 mol, 1.1 eq.) was added dropwise. The mixture was stirred at −78 °C for 1 hour, before being warmed to room temperature and stirred overnight. After this, saturated aqueous NH_4Cl (20 mL) was added, before the mixture was evaporated to dryness. Et_2O (150 mL) and 2M aqueous NaOH (75 mL) were added, and the mixture was vigorously stirred for 15 minutes. After this, the organic portion was separated, and the aqueous portion was extracted with Et_2O (x2). The organic portions were combined and sequentially washed with distilled water and then brine, before being dried over Na_2SO_4, filtered, and concentrated *in vacuo*. The crude mixture was subjected to silica gel chromatography (0:1 to 1:4, EtOAc: petroleum ether (PET)) to afford the title compound (6.08 g, 66%) as a clear viscous oil.

^{1}H NMR (400 MHz, 298K, $CDCl_3$) δ 5.78–5.76 (m, 1H), 3.71 (s, 3H), 2.65–2.58 (m, 1H), 2.48–2.39 (m, 4H), 2.18–2.11 (m, 1H), 1.97–1.87 (m, 1H) ppm. The ^{1}H NMR data matched that reported in the literature.

Triflate **20**[2]

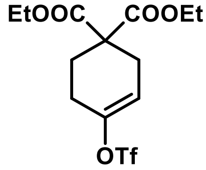

To a dry flask containing diethyl 4-oxocyclohexane-1,1-dicarboxylate (1.60 mL, 1.84 g, 7.60 mmol, 1 eq.) and *N*-(5-chloropyridin-2-yl)-*N*-(methanesulfonyl)methanesulfonamide (Comin's reagent, 3.28 g, 8.36 mmol, 1.1 eq.) was added dry THF (30 mL) under a nitrogen atmosphere. The mixture was cooled to −78 °C, before 1M NaHMDS in THF (8.4 mL, 8.4 mmol, 1.1 eq.) was added dropwise. The mixture was stirred at −78 °C for 1 hour, before being warmed to room temperature and stirred overnight. After this, saturated aqueous NH_4Cl (20 mL) was added, before the mixture was evaporated to dryness. Et_2O (150 mL) and 2M aqueous NaOH (75 mL) were added, and the mixture was vigorously stirred for 15 minutes. After this, the organic portion was separated, and the aqueous portion was extracted with Et_2O (x2). The organic portions were combined and sequentially washed with distilled water and then

brine, before being dried over Na_2SO_4, filtered, and concentrated *in vacuo* to afford the title compound (2.76 g, 88%) as a clear viscous oil.

^{1}H NMR (400 MHz, 298K, $CDCl_3$) δ 5.77–5.75 (m, 1H), 4.27–4.13 (m, 4H), 2.76 (dt, 2H, *J* = 4.6, 2.5 Hz), 2.47–2.40 (m, 2H), 2.30 (app t, 2H, *J* = 6.7 Hz), 1.26 (t, 6H, *J* = 7.2 Hz) ppm. The ^{1}H NMR data matched that reported in the literature.

Diene **21**[3]

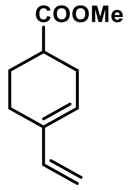

To a dry sealable glass vessel containing triflate **19** (0.826 g, 2.87 mmol, 1 eq.) lithium chloride (0.364 g, 8.60 mmol, 3 eq), palladium acetate (0.032 g, 0.14 mmol, 0.05 eq.), and (ferrocene-1,1′-diyl)bis(diphenylphosphane) (0.078 g, 0.14 mmol, 0.05 eq.), was added dry THF (10 mL), followed by vinyl tributyl tin (1.08 mL, 0.909 g, 2.87 mmol, 1 eq.) under a nitrogen atmosphere. The mixture was pump purged using nitrogen gas three times, before being heated to 100 °C and stirred overnight. At completion as monitored by TLC, the reaction mixture was filtered through Celite using Et_2O, and dried *in vacuo*. The crude mixture was subjected to silica gel chromatography (0:1 to 1:9, Et_2O: PET, immediately after loading PET (300 mL) was flushed through the column to remove as much leftover tributyltin hydroxide as possible) to provide the title compound (0.258 g, 54%, adjusted for 20% $SnBu_3OH$ contamination) as a clear oil.

^{1}H NMR (400 MHz, 298K, $CDCl_3$) δ 7.26 (dd, 1H, *J* = 17.4, 10.7 Hz), 5.74–5.72 (m, 1H), 5.10–5.05 (m, 1H), 4.95–4.92 (m, 1H), 3.69 (s, 3H), 2.60–2.53 (m, 1H), 2.39–2.28 (m, 3H), 2.20–2.07 (m, 2H), 1.78–1.70 (m, 1H) ppm. The ^{1}H NMR data matched that reported in the literature. Additional peaks corresponding to $SnBu_3OH$ can be found at δ 1.67–1.60, 1.45–1.21, and 0.93–0.90 ppm.

Diene **22**[4]

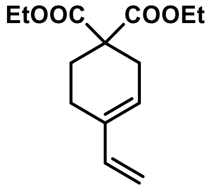

To a dry sealable glass vessel containing triflate **20** (1.117 g, 2.984 mmol, 1 eq.) lithium chloride (0.379 g, 8.95 mmol, 3 eq), palladium acetate (0.033 g, 0.15 mmol, 0.05 eq.), and (ferrocene-1,1′-diyl)bis(diphenylphosphane) (0.081 g, 0.15 mmol, 0.05 eq.), was added dry THF (15 mL), followed by vinyl tributyl tin (0.88 mL, 0.95 g, 3.0 mmol, 1 eq.) under a nitrogen atmosphere. The mixture was pump purged using nitrogen gas three times, before being heated to 100 °C and stirred overnight. At completion as monitored by TLC, the mixture was filtered through Celite using Et_2O and dried *in vacuo*. The crude mixture was subjected to silica gel chromatography (0:1 to 1:9, Et_2O: PET, immediately after loading PET (300 mL) was flushed through the column to remove as much leftover tributyl tin hydroxide as possible) to provide the title compound (0.516 g, 67%, adjusted for 16% $SnBu_3OH$ contamination) as a clear oil.

^{1}H NMR (400 MHz, 298K, $CDCl_3$) δ 6.32 (dd, 1H, *J* = 17.5, 10.8 Hz), 5.71 (m, 1H), 5.05 (d, 1H, *J* = 17.3 Hz), 4.92 (d, 1H, *J* = 11.1 Hz), 4.18 (q, 4H, *J* = 6.9 Hz), 2.68 (s, 2H), 2.21 (s, 4H), 1.24 (t, 6H, *J* = 7.0 Hz) ppm.

The ^{1}H NMR data matched that reported in the literature. Additional peaks corresponding to $SnBu_3OH$ can be found at δ 1.67-1.60, 1.45-1.21, and 0.93-0.90 ppm.

Alkene **25** and alkene **25a**

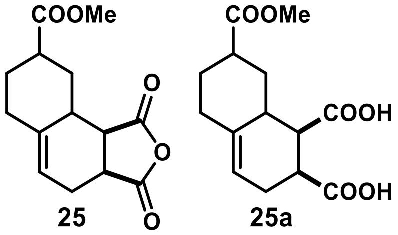

25 25a

A solution of diene **21** (0.150 g, 0.91 mmol, 1 eq.) and maleic anhydride **23** (0.089 g, 0.90 mmol, 1 eq.) in toluene (3 mL) was pump purged with nitrogen in a sealable glass vessel. The mixture was heated to 80 °C and stirred overnight. At completion as monitored by TLC, the solvent was removed *in vacuo*, and the crude residue subjected to silica gel column chromatography (0:1 to 2:3, EtOAc: PET) to afford the title product **25** (0.125 g, 52%, adjusted for 8% $SnBu_3OH$ contamination) as a mixture of diastereomers. An additional 41 mg of material was recovered by increasing the column gradient to 1:0 EtOAc: PET that corresponded to the product with the hydrolyzed anhydride **25a**. This could be incorporated to subsequent hydrogenation and hydrolysis experiments to provide compounds **9**, **13** or **29**.

^{1}H NMR (400 MHz, 298K, $CDCl_3$) δ 5.51–5.40 (m, 1H), 3.75–3.68 (s, 3H), 3.29-3.27 (m, 1H), 2.82–2.72 (m, 4H), 2.56–1.95 (m, 7H), 1.57–1.50 (m, 1H) ppm. Additional peaks corresponding to $SnBu_3OH$ can be found at δ 1.67–1.60, 1.45–1.21, and 0.93–0.90 ppm. ^{13}C NMR (101 MHz, 298K, $CDCl_3$) δ 180.5, 179.6, 175.3, 175.2, 135.2, 119.3, 51.96, 51.87, 50.7, 44.1, 43.9, 43.3, 41.3, 39.9, 39.2, 37.6, 34.5, 30.9, 30.6, 28.6, 27.3, 24.4 ppm. Additional peaks corresponding to $SnBu_3OH$ can be found at δ 27.9, 26.9, 17.7, and 13.7 ppm. HRMS (ESI-MS) *m/z* for **25** not observed. HRMS (ESI-MS) calculated for **25a** $C_{14}H_{17}O_6^-$ [M - H]$^-$: 281.1031; found: 281.1029.

Alkene **26**

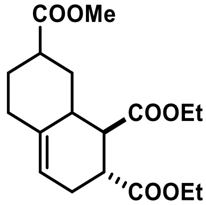

A solution of diene **21** (0.140 g, 0.845 mmol, 1 eq.) and diethyl fumarate (0.14 mL, 0.15 g, 0.84 mmol, 1 eq.) in toluene (3 mL) was pump purged with nitrogen in a sealable glass vessel. The mixture was heated to 130 °C and stirred overnight. At completion as monitored by TLC, the solvent was removed *in vacuo*, and the crude residue subjected to silica gel column chromatography (0:1 to 1:1, EtOAc: PET) to afford the title product (0.215 g, 74%, adjusted for 3% $SnBu_3OH$ contamination) as a mixture of diastereomers.

^{1}H NMR (400 MHz, 298K, $CDCl_3$) δ 5.43–5.36 (m, 1H), 4.27–4.03 (m, 4H), 3.69–3.61 (m, 3H), 3.06–2.67 (m, 2H), 2.62–2.21 (m, 4H), 2.20–1.88 (m, 4H), 1.50–1.30 (m, 2H), 1.29–1.21 (m, 6H) ppm. Additional peaks corresponding to $SnBu_3OH$ can be found at δ 1.67–1.60, 1.45–1.21, and 0.93–0.90 ppm.^{13}C NMR (101 MHz, 298K, $CDCl_3$) δ 175.7 175.6, 175.5, 175.3, 175.0, 174.81, 174.79, 174.6, 174.5, 174.3, 173.5, 173.3, 140.2, 139.2, 137.97, 136.91, 118.6, 117.9, 117.2, 116.3, 60.8, 60.7, 60.7, 60.60, 60.58, 60.5, 51.80, 51.79, 51.77, 51.7, 49.8, 49.5, 45.8, 45.5, 43.6, 42.8, 42.5, 42.2, 39.8, 39.5, 39.2, 38.1, 37.9, 37.7,

37.0, 35.7, 35.1, 34.7, 33.74, 33.70, 32.9, 32.8, 31.7, 31.2, 29.7, 29.5, 28.9, 28.1, 27.9, 14.4, 14.32, 14.30, 14.24, 14.22 ppm. Additional peaks corresponding to $SnBu_3OH$ can be found at δ 27.9, 26.9, 17.7, and 13.7 ppm. HRMS (ESI-MS): calculated for $C_{18}H_{27}O_6^+$ $[M + H]^+$: 339.1802; found: 339.1797.

Alkene **27**

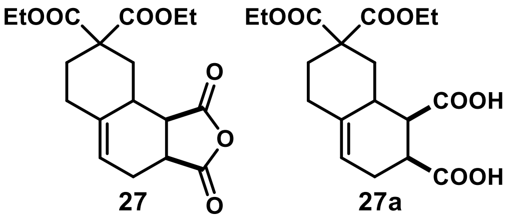

27 **27a**

A solution of diene **22** (0.090 g, 0.36 mmol, 1 eq.) and maleic anhydride (0.035 g, 0.36 mmol, 1 eq.) in toluene (1 mL) was pump purged with nitrogen in a sealable glass vessel. The mixture was heated to 80 °C and stirred overnight. At completion as monitored by TLC, the solvent was removed *in vacuo*, and the crude residue subjected to silica gel column chromatography (0:1 to 2:3, EtOAc: PET) to afford the title product **27** (0.044 g, 34%, adjusted for 4% $SnBu_3OH$ contamination) as a mixture of diastereomers. An additional 19 mg of material was recovered by increasing the column gradient to 1:0 EtOAc: PET that corresponded to the product with the hydrolyzed anhydride **27a**. This could be incorporated to subsequent hydrogenation and hydrolysis experiments to provide compounds **11**, **15**, or **31**.

^1H NMR (400 MHz, 298K, $CDCl_3$) δ 5.58 (m, 1H), 4.25–4.13 (m, 4H), 2.71–2.38 (m, 4H), 2.29–2.14 (m, 5H), 1.24 (t, 6H, *J* = 6.7 Hz) ppm. Additional peaks corresponding to $SnBu_3OH$ can be found at δ 1.67–1.60, 1.45–1.21, and 0.93–0.90 ppm.^{13}C NMR (101 MHz, 298K, $CDCl_3$) δ 179.3, 177.6, 176.7, 174.0, 173.7, 171.9, 171.8, 171.7, 171.6, 171.4, 171.4, 170.8, 139.4, 134.0, 133.8, 119.7, 118.2, 68.0, 61.94, 61.85, 61.74, 61.72, 61.6, 61.4, 60.6, 55.20, 55.19, 53.9, 53.6, 53.3, 52.2, 50.8, 44.1, 43.9, 43.5, 41.6, 41.4, 39.9, 37.8, 34.9, 34.7, 34.1, 31.5, 31.2, 31.0, 30.96, 30.88, 30.8, 30.5, 30.4, 29.6, 29.1, 25.7, 24.8, 24.5, 23.4, 21.1, 14.3, 14.2, 14.14, 14.10, 14.06 ppm. Additional peaks corresponding to $SnBu_3OH$ can be found at δ 27.9, 26.9, 17.7, and 13.7 ppm. HRMS (ESI-MS): calculated for **27** $C_{18}H_{23}O_7^+$ $[M + H]^+$: 351.1438; found: 351.1432.

Alkene **28**

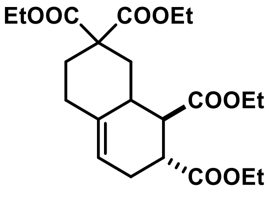

A solution of diene **22** (0.091 g, 0.36 mmol, 1 eq.) and diethyl fumarate (0.059 mL, 0.062 g, 0.36 mmol, 1 eq.) in toluene (1 mL) was pump purged with nitrogen in a sealable glass vessel. The mixture was heated to 130 °C and stirred overnight. At completion as monitored by TLC, the solvent was removed *in vacuo*, and the crude residue subjected to silica gel column chromatography (0:1 to 1:1, EtOAc: PET) to afford the title product (0.073 g, 47%, adjusted for 3% $SnBu_3OH$ contamination) as a mixture of diastereomers.

^1H NMR (400 MHz, 298K, $CDCl_3$) δ 5.44–5.41 (m, 1H), 4.26–4.04 (m, 8H), 3.05 (dd, 0.3H, *J* = 12.2, 6.5 Hz), 2.82–2.72 (m, 1H), 2.65–2.03 (m, 8H), 1.69–1.59 (m, 1.7H), 1.30–1.18 (m, 12H) ppm. Additional peaks corresponding to $SnBu_3OH$ can be found at δ 1.67–1.60, 1.45–1.21, and 0.93–0.90 ppm. ^{13}C NMR (101 MHz, 298K CDCl3) δ 175.6, 174.4, 174.3, 173.2, 171.8, 171.6, 170.8, 170.5, 138.8, 136.5, 118.6,

117.2, 61.7, 61.6, 61.5, 61.4, 60.76, 60.75, 60.72, 60.65, 55.8, 55.1, 49.5, 45.5, 42.3, 37.7, 37.1, 36.9, 35.2, 35.1, 33.5, 32.4, 31.7, 30.9, 28.9, 28.1, 14.4, 14.34, 14.28, 14.25, 14.22, 14.17, 14.1 ppm. Additional peaks corresponding to $SnBu_3OH$ can be found at δ 27.9, 26.9, 17.7, and 13.7 ppm. HRMS (ESI-MS): calculated for $C_{22}H_{32}O_8^+$ $[M + H]^+$: 425.2170; found: 425.2165.

Alkane **29** and alkane **29a**

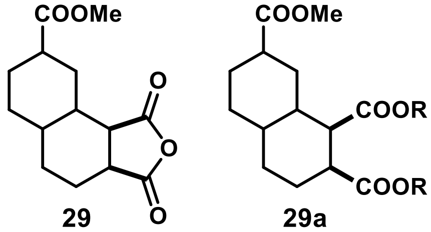

29 **29a**
R = H/OMe

To a solution of ester **25** and acid **25a** (0.032 g) in methanol (2 mL) was added PtO_2 (0.008 g, 25% w/w). The mixture was pump purged with hydrogen gas and stirred for 3 hours at room temperature and atmospheric pressure under hydrogen. After this, the mixture was filtered through Celite using methanol and concentrated *in vacuo* to provide 33 mg of the title compounds **29** and **29a**. Conversion of the starting alkene was monitored by ^1H NMR for the loss of the alkene peak, and crude spectra are included in the spectral SI. The mixture was carried forward without purification.

Alkane **30**

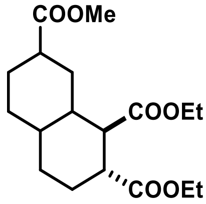

To a solution of ester **26** (0.096 g, 0.28 mmol) in EtOAc (2 mL) was added PtO_2 (0.024 g, 25% w/w) and a drop of acetic acid. The mixture was pump purged with hydrogen gas before being heated to 80 °C under hydrogen at 10 atmospheres of pressure and stirred overnight. After this, the mixture was filtered through Celite using methanol and concentrated *in vacuo* to provide the title compound (0.090 g, 94%) as a clear semi-solid. Conversion of the starting alkene was monitored by ^1H NMR for the loss of the alkene peak, and crude spectra are included in the spectral SI. The mixture was carried forward without purification.

Alkane **31** and **31a**

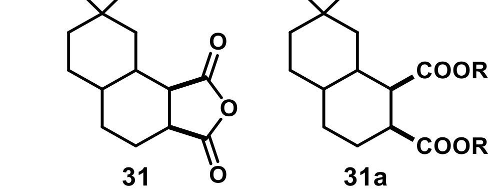

31 **31a**
R = H/OMe

To a solution of ester **27** and acid **27a** (0.134 g, 0.382 mmol) in methanol (2 mL) was added PtO_2 (0.044 g, 30% w/w) and a drop of acetic acid. The mixture was pump purged with hydrogen gas and stirred for 3 hours at room temperature and atmospheric pressure under hydrogen. After this, the mixture was filtered through Celite using methanol and concentrated *in vacuo* to provide 102 mg of the title

compounds **31** and **31a**. Conversion of the starting alkene was monitored by ^{1}H NMR for the loss of the alkene peak, and crude spectra are included in the spectral SI. The mixture was carried forward without purification.

Alkane **32**

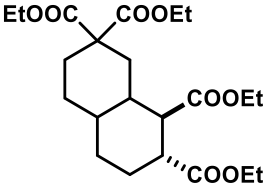

To a solution of ester **28** (0.035 g, 0.082 mmol) in EtOAc (2 mL) was added PtO_2 (0.009 g, 25% w/w). The mixture was pump purged with hydrogen gas before being heated to 80 °C under hydrogen at 10 atmospheres of pressure and stirred overnight. After this, the mixture was filtered through Celite using methanol and concentrated *in vacuo* to provide the title compound **32** (0.030 g, 85%) as a clear semi-solid. Conversion of the starting alkene was monitored by ^{1}H NMR for the loss of the alkene peak, and crude spectra are included in the spectral SI. The mixture was carried forward without purification.

CRAM **9**

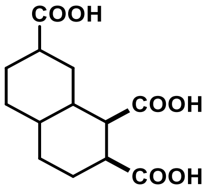

To a solution of ester **29** and acid **29a** (0.033 g, 0.12 mmol as calculated from anhydride **29**) was added methanol (0.25 mL) and 25% aqueous NaOH (5 mL). The solution was heated to 50 °C and vigorously stirred for 60 hours. At completion as monitored by NMR, the mixture was acidified to pH 1 with 1M aqueous HCl and extracted with EtOAc (x3). The organic portions were combined, dried over Na_2SO_4, filtered, and concentrated *in vacuo* to afford the title compound **9** (0.019 g, 58% as calculated from anhydride **29**) as an off-white powder as a mixture of diastereomers.

^{1}H NMR (600 MHz, 298K, 0.1M NaOD in D_2O) δ 3.06-2.29 (m, 2H), 2.27-2.00 (m, 3H), 1.98-1.01 (m, 9H). ^{13}C NMR (151 MHz, 298K, 0.1M NaOD in D_2O) δ 187.8, 187.7, 187.4, 185.4, 184.9, 184.8, 184.7, 184.6, 184.1, 183.6, 52.4, 51.9, 51.6, 49.5, 48.8, 48.6, 48.5, 42.8, 42.2, 41.8, 38.6, 38.3, 37.8, 37.5, 36.2, 35.6, 33.5, 32.8, 32.3, 31.6, 31.5, 30.9, 30.0, 29.7, 28.6, 27.8, 27.2, 25.9, 25.7, 25.4, 25.2, 24.9, 23.4, 22.8.ppm. HRMS (ESI-MS): calculated for $C_{13}H_{17}O_6^-$ [M - H]$^-$: 269.1031; found: 269.0972. LCMS-CAD purity: 92%.

CRAM **10**

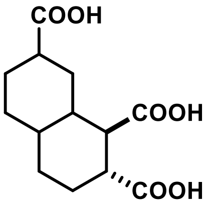

To a solution of ester **30** (0.051 g, 0.15 mmol) was added methanol (0.25 mL) and 25% aqueous NaOH (5 mL). The solution was heated to 50 °C and vigorously stirred for 60 hours. At completion as monitored by NMR, the mixture was extracted with Et_2O (x2), and these organic portions were

discarded. The remaining aqueous mixture was acidified to pH 1 with 1M aqueous HCl and extracted with EtOAc (x3). The organic portions were combined, dried over Na_2SO_4, filtered, and concentrated *in vacuo* to afford the title compound **10** (0.032 g, 79%) as an off-white powder as a mixture of diastereomers.

^{1}H NMR (600 MHz, 298K, 0.1M NaOD in D_2O) δ 2.78-2.33 (m, 2H), 2.19-1.79 (m, 3H), 1.77-0.95 (m, 9H). ^{13}C NMR (151 MHz, 298K 0.1M NaOD in D_2O) δ 187.4, 187.1, 186.9, 186.7, 186.6, 185.5, 185.4, 185.3, 184.9, 184.6, 184.4, 184.3, 184.0, 57.1, 56.7, 54.1, 53.8, 52.4, 51.9, 51.81, 51.75, 48.5, 48.4, 47.9, 44.4, 44.3, 44.2, 42.3, 42.2, 41.8, 41.4, 41.3, 39.1, 38.9, 38.3, 36.8, 36.6, 35.6, 35.4, 35.0, 33.6, 33.52, 33.47, 33.4, 33.2, 32.8, 31.63, 31.58, 31.5, 31.1, 30.9, 30.8, 30.4, 30.3, 30.2, 29.1, 28.2, 26.6, 25.9, 25.44, 25.38, 25.2, 24.92, 24.89, 22.8, 22.5. HRMS (ESI-MS): calculated for $C_{13}H_{17}O_6^-$ [M - H]$^-$: 269.1031; found: 269.0972. LCMS-CAD purity: 99%.

CRAM **11**

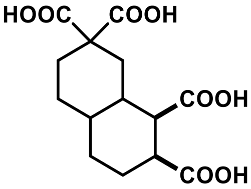

To a solution of ester **31** and acid **31a** (0.091 g, 0.26 mmol as calculated from anhydride **31**) was added methanol (0.25 mL) and 25% aqueous NaOH (5 mL). The solution was heated to 50 °C and vigorously stirred for 60 hours. At completion as monitored by NMR, the mixture was extracted with Et_2O (x2), and these organic portions were discarded. The remaining aqueous mixture was acidified to pH 1 with 1M aqueous HCl and extracted with EtOAc (x3). The organic portions were combined, dried over Na_2SO_4, filtered, and concentrated *in vacuo* to afford the title compound **11** (0.022 g, 26% as calculated from anhydride **31**) as an off-white powder as a mixture of diastereomers.

^{1}H NMR (600 MHz, 298K, 0.1M NaOD in D_2O) δ 2.88-2.25 (m, 2H), 2.22-1.77 (m, 4H), 1.74-1.30 (m, 6H), 1.37-0.98 (m, 2H) ppm. ^{13}C NMR (151 MHz, 298K, 0.1M NaOD in D_2O) δ 186.1, 185.0, 184.9, 184.4, 184.1, 183.7, 183.51, 183.45, 183.4, 183.3, 183.1, 182.9, 181.7, 181.6, 181.2, 63.8, 60.0, 59.9, 59.8, 55.9, 53.4, 51.3, 50.9, 47.9, 46.4, 45.9, 43.8, 42.6, 42.3, 42.2, 41.6, 40.3, 38.5, 37.9, 36.6, 36.5, 35.98, 35.88, 35.6, 35.3, 34.9, 34.3, 34.1, 34.0, 33.0, 32.2, 31.6, 31.1, 30.9, 30.7, 30.5, 29.8, 29.5, 29.3, 28.9, 28.9, 28.8, 28.5, 28.1, 27.7, 27.6, 27.5, 26.9, 24.8, 24.3, 22.8, 22.7, 22.4, 22.2.ppm. HRMS (ESI-MS): calculated for $C_{14}H_{17}O_8^-$ [M - H]$^-$: 313.0929; found: 313.0861. LCMS-CAD purity: 90%.

CRAM **12**

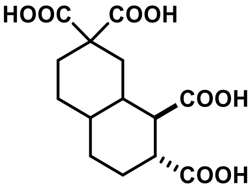

To a solution of ester **32** (0.026 g, 0.061 mmol) was added methanol (0.25 mL) and 25% aqueous NaOH (5 mL). The solution was heated to 50 °C and vigorously stirred for 60 hours. At completion as monitored by NMR, the mixture was extracted with Et_2O (x2), and these organic portions were discarded. The remaining aqueous mixture was acidified to pH 1 with 1M aqueous HCl and extracted with EtOAc (x3). The organic portions were combined, dried over Na_2SO_4, filtered, and concentrated *in*

vacuo to afford the title compound **12** (0.011 g, 57%) as an off-white powder as a mixture of diastereomers.

^{1}H NMR (600 MHz, 298K, 0.1M NaOD in D_2O) δ 2.97–2.42 (m, 2H), 2.32–2.03 (m, 3H), 1.94–1.66 (m, 3.5 H), 1.63–1.32 (m, 4.5 H), 1.14–1.00 (m, 1H) ppm. ^{13}C NMR (151 MHz, 298K, 0.1M NaOD in D_2O) 13C NMR (151 MHz, D2O) δ 186.10, 185.01, 184.90, 184.36, 183.65, 183.52, 183.28, 183.16, 182.93, 181.64, 181.22, 181.17, 59.78, 56.52, 53.39, 51.23, 50.86, 43.78, 42.68, 41.27, 41.07, 37.47, 35.99, 35.64, 35.35, 34.02, 32.78, 32.58, 32.18, 30.70, 30.46, 29.81, 28.97, 28.43, 27.75, 26.91, 24.75, 22.91 ppm. HRMS (ESI-MS): calculated for $C_{14}H_{17}O_8^-$ [M - H]$^-$: 313.0929; found: 313.0862. LCMS-CAD purity: 98%.

CRAM **13**

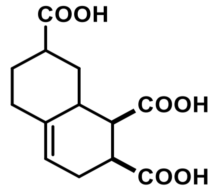

To a solution of ester **25** and acid **25a** (0.031 g, 0.012 mmol as calculated from anhydride **25**) was added methanol (0.25 mL) and 25% aqueous NaOH (5 mL). The solution was heated to 50 °C and vigorously stirred for 60 hours. At completion as monitored by NMR, the mixture was extracted with Et_2O (x2), and these organic portions were discarded. The remaining aqueous mixture was acidified to pH 1 with 1M aqueous HCl and extracted with EtOAc (x3). The organic portions were combined, dried over Na_2SO_4, filtered, and concentrated *in vacuo* to afford the title compound **5** (0.024 g, 74% as calculated from anhydride **25**) as an off-white powder as a mixture of diastereomers.

^{1}H NMR (600 MHz, 298K, 0.1M NaOD in D_2O) δ 2.76-2.20 (m, 2H), 2.16-1.86 (m, 3H), 1.85-1.39 (m, 5H), 1.33-0.98 (m, 3H) ppm. ^{13}C NMR (151 MHz, 298K, 0.1M NaOD in D_2O) δ 186.5, 184.8, 184.6, 184.5, 184.2, 184.1, 181.8, 181.7, 138.2, 137.3, 120.6, 119.9, 118.7, 117.8, 55.2, 50.5, 50.4, 47.5, 45.9, 42.0, 41.8, 39.6, 37.9, 36.7, 35.2, 34.2, 34.0, 31.8, 31.6, 30.5, 29.8, 29.2, 28.6, 25.8. HRMS (ESI-MS): calculated for $C_{13}H_{15}O_6^-$ [M - H]$^-$: 267.0874; found: 267.0819. LCMS-CAD purity: 100%.

CRAM **14**

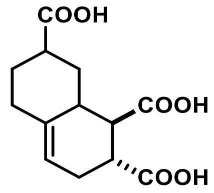

To a solution of ester **26** (0.051 g, 0.15 mmol) was added methanol (0.25 mL) and 25% aqueous NaOH (5 mL). The solution was heated to 50 °C and vigorously stirred for 60 hours. At completion as monitored by NMR, the mixture was extracted with Et_2O (x2), and these organic portions were discarded. The remaining aqueous mixture was acidified to pH 1 with 1M aqueous HCl and extracted with EtOAc (x3). The organic portions were combined, dried over Na_2SO_4, filtered, and concentrated *in vacuo* to afford the title compound **14** (0.028 g, 69%) as an off-white powder as a mixture of diastereomers.

^{1}H NMR (600 MHz, 298K, 0.1M NaOD in D_2O) δ 5.48–5.42 (m, 1H), 2.99–2.59 (m, 2.5H), 2.56–1.87 (m, 8H), 1.50–1.34 (m, 1.5H) ppm. ^{13}C NMR (151 MHz, 298K, 0.1M NaOD in D_2O) δ 180.7, 180.1, 179.7, 179.6, 179.5, 178.8, 178.5, 178.0, 142.1, 141.2, 139.7, 138.9, 119.7, 119.1, 118.0, 117.4, 52.3, 52.1,

48.2, 47.9, 47.6, 45.7, 44.9, 44.9, 44.8, 41.13, 41.05, 40.5, 40.1, 39.9, 39.4, 38.4, 37.1, 36.8, 35.9, 35.2, 34.9, 34.8, 33.9, 33.2, 32.8, 32.3, 31.1, 30.9, 30.7, 30.4, 30.3, 29.8, 29.7, 29.2 ppm. HRMS (ESI-MS): calculated for $C_{13}H_{15}O_6^-$ [M - H]$^-$: 267.0874; found: 267.0819. LCMS-CAD purity: 95%.

CRAM **15**

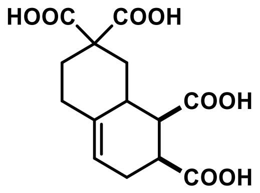

To a solution of ester **27** and acid **27a** (0.016 g, 0.046 mmol as calculated from anhydride **27**) was added methanol (0.25 mL) and 25% aqueous NaOH (5 mL). The solution was heated to 50 °C and vigorously stirred for 60 hours. At completion as monitored by NMR, the mixture was extracted with Et_2O (x2), and these organic portions were discarded. The remaining aqueous mixture was acidified to pH 1 with 1M aqueous HCl and extracted with EtOAc (x3). The organic portions were combined, dried over Na_2SO_4, filtered, and concentrated *in vacuo* to afford the title compound **15** (0.008 g, 55% as calculated from anhydride **27**) as an off-white powder as a mixture of diastereomers.

^{1}H NMR (600 MHz, 298K, 0.1M NaOD in D_2O) δ 5.41–5.36 (m, 1H), 3.12–3.11 (m, 1H), 2.93–2.85 (m, 1H), 2.80–2.78 (m, 1H), 2.56–2.53 (m, 1H), 2.26–2.20 (m, 2H), 2.02–1.69 (m, 2H), 1.69–1.36 (m, 3H) ppm. ^{13}C NMR (151 MHz, 298K, 0.1M NaOD in D_2O) δ 179.5, 178.4, 139.6, 119.2, 53.7, 42.8, 39.0, 37.7, 34.2, 33.1, 32.8, 30.8, 30.7, 30.4 ppm. HRMS (ESI-MS): calculated for $C_{14}H_{15}O_8^-$ [M - H]$^-$: 311.0772; found: 311.0707. LCMS-CAD purity: 94%.

CRAM **16**

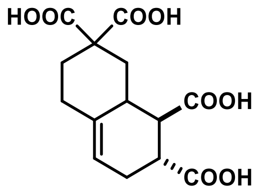

To a solution of ester **28** (0.026 g, 0.061 mmol) was added methanol (0.25 mL) and 25% aqueous NaOH (5 mL). The solution was heated to 50 °C and vigorously stirred for 60 hours. At completion as monitored by NMR, the mixture was extracted with Et_2O (x2), and these organic portions were discarded. The remaining aqueous mixture was acidified to pH 1 with 1M aqueous HCl and extracted with EtOAc (x3). The organic portions were combined, dried over Na_2SO_4, filtered, and concentrated *in vacuo* to afford the title compound **16** (0.010 g, 52%) as an off-white powder as a mixture of diastereomers.

^{1}H NMR (600 MHz, 298K, 0.1M NaOD in D_2O) δ 2.77-2.39 (m, 3H), 2.38-1.95 (m, 7H), 1.52-1.25 (m, 2H). ppm. ^{13}C NMR (151 MHz, 298K, 0.1M NaOD in D_2O) δ 185.6, 184.5, 183.9, 182.6, 182.5, 182.4, 180.8, 180.5, 141.9, 139.6, 118.1, 118.0, 116.8, 116.8, 60.4, 60.1, 59.5, 54.6, 50.5, 50.0, 47.00, 42.1, 41.2, 40.9, 39.3, 37.7, 37.2, 35.9, 35.4, 34.9, 33.4, 33.1, 32.9, 31.6, 29.6, 29.2, 29.1. ppm. HRMS (ESI-MS): calculated for $C_{14}H_{15}O_8^-$ [M - H]$^-$: 311.0772; found: 311.0706. LCMS-CAD purity: 97%.

Additional Synthetic Details

*Discussion Regarding the Coupling of Vinyl Triflates **19** or **20** to form Dienes **21** or **22***

The preparation of dienes **21** or **22** was investigated through Suzuki couplings of triflates **19** or **20** and vinyl boron reagents (H_2C=CH3BF_3K, H_2C=CHBpin), and Heck-type couplings using vinyl acetate. Unfortunately, while some of the product diene was observed in these systems (<10% by ^{1}H NMR), Suzuki couplings led predominantly to enol triflate hydrolysis to the parent ketones, and the Heck-type cross-couplings provided intractable complex mixtures. Pleasingly, the use of vinyl tributyl tin in Stille cross-couplings provided diene products **21** or **22**. Residual tributyl tin hydroxide was frequently inseparable from the diene products, however, removal of this material was observed in the final synthetic step, and its presence did not affect subsequent Diels-Alder or hydrogenation procedures.

*Discussion Regarding the Hydrogenation of Alkenes **25**-**28** to Alkanes **29**-**32***

Diels-Alder adducts **25**-**28** were subjected to Pd/C hydrogenation in efforts to provide bicyclic alkanes **29**-**32**. Curiously, the majority of hydrogenations performed at ambient pressure and room temperature delivered only starting material, or in the case of the parent Diels-Alder adducts of CRAM's **14** and **16**, the reduction of some diastereomers but not others. Increasing the pressure to 10 bar of hydrogen and the temperature to 80 °C returned the same results. In attempts to overcome this, several other catalysts were screened at these elevated conditions, including $Pd(OH)_2$/C, Rh/C, Raney nickel, and PtO_2. Of these, only PtO_2 provided the loss of NMR signal that corresponded to the alkene functionality. Notably, while at this higher pressure and temperature, anhydride functionalities within these molecules were found to be reduced to the corresponding lactone in significant proportions (i.e. greater than 50% of the recovered according to NMR). This could be avoided by running the reactions at ambient pressure and temperature, but required careful monitoring for the loss of starting material, as extended reaction times still led to the formation of these lactones.

*Discussion Regarding the Hydrolysis of Esters **25**-**32** to Acids **9**-**16***

While alkenes were omitted as intentional targets within the scope of this work, it was seen as trivial to subject esters **25-28** to hydrolysis to provide their corresponding tri- and tetra-acid counterparts **13**-**16**. Naturally, these hydrolyses were not without challenge, as the use of 4:1 4M aqueous NaOH:methanol at room temperature provided only partial loss of the NMR integral attributable to the ester functionalities. Modification of the procedure to use 4:1 6M aqueous NaOH:methanol at 50 °C for 24 hours, before the removal of the methanol and continued hydrolysis for a subsequent 48 hours at 50 °C provided CRAMs **9**-**16** as diastereomeric mixtures. Once more, these diastereomers were found to be inseparable using in-house chromatographic methods.

Chemical Database Experiments

To attempt to show the limited number of CRAM-like compounds that actually exist in the literature, two experiments based on parameter refinement within the chemical database Scifinder are presented.

Scifinder experiment one begins by searching for all compounds with one six membered ring, and at least 3 carboxylic acids. The initial search returns 59,405 results. Using Hertkorn *et al*'s carboxyl-C:aliphatic-C ratio of 1:2 to 1:7[5] allows a maximum molecular weight to be set at 429 Da, and reduces the number of hits to 21,405 (3 acids weigh 135 Da, 7 additional CH_2 units per acid weigh 294 Da. This mass is somewhat higher than the actual maximum, as it does not factor in losses of hydrogens on carbons to account for carboxylate incorporation or ring formations). Subsequently, multicomponent compounds are removed, as they nearly exclusively consist of polymers, with some of the main co-polymer components being small molecule acids such as acetic, succinic, or citric acid. These comprise a large number of hits within these types of search engines, as three separate components containing a single acid will be registered by Scifinder in this type of search. Additional non-polymeric mixtures are removed by filtering for incompletely defined substances, as this contains many chemical mixtures containing acetic or citric acid. This leaves only 1,115 compounds.

Removal of compounds containing atoms that are not C, H, or O, as well as compounds that have no references (i.e. at some point have been listed by a company on Scifinder), returns 610 results. Removal of compounds that contain extended aromatic rings returns 514 results. Out of these 514 compounds, only 43 have literature reported ^{1}H NMR, ^{13}C NMR, IR, or MS data:

- 13C NMR – 36 results
- 1H NMR – 20 results
- IR spectrum – 4 results
- MS – 3 results

Around half (21) of these structure are bridged bicyclic-like systems (figure S1, **a**)[6] or adamantane-like systems (figure S1, **b**).[7] These bridged ring structures have not been proposed as a component of DOM to our knowledge.

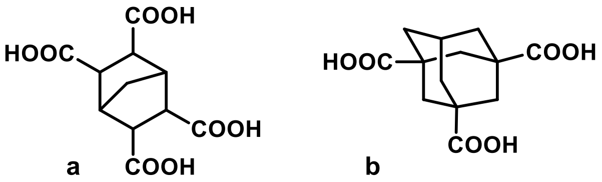

Figure S1: *Representative bridged bicyclic-like **a** or adamantane-like **b** acids within Scifinder experiment one.*

Of the remaining 22 compounds, 11 of them contain only a single ring, failing Hertkorn's *et al.'s* proposed requirement for CRAM to contain fused alicyclic rings (Figure S2, **a**). Furthermore, around half of these compounds exceed the number of carboxylic acids to satisfy the carboxyl-C:aliphatic-C ratio of 1:2 to 1:7 (Figure S2, **b**).

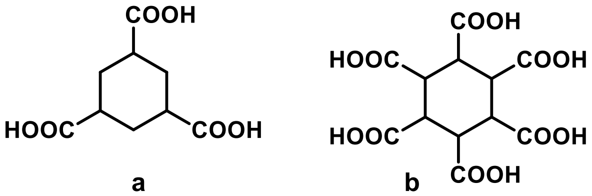

a b

Figure S2: *Representative single-ring systems within Scifinder experiment one.*

Four of the remaining 11 compounds are accounted for by gibbane-type natural products (Figure S3).[8] These intermediates are isolated in small quantities, not reliably available, and do not have established synthetic routes. Furthermore, in the context of CRAM, their exo-alkene functionality is highly sensitive to oxidation and unlikely to survive prolonged geochemical exposure.

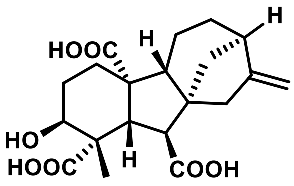

GA_{13}

Figure S3: *GA_{13}, a biosynthetic intermediate to gibberellin.*

Of the seven remaining compounds, two compounds contain no alicyclic acids, instead having their carboxylic acid functionalities derived from a citric acid ether (**a**, Figure S4).[9] They are both natural product isolates, with no established synthetic route. A second compound is a lactone fused to an aromatic resorcinol (**b**, Figure S4).[10] The remaining five compounds are non-fused bicyclic systems (**c**, Figure E4),[11] that apart from the lack of fused alicyclic rings, are perhaps the best remaining CRAM-like compounds from this dataset.

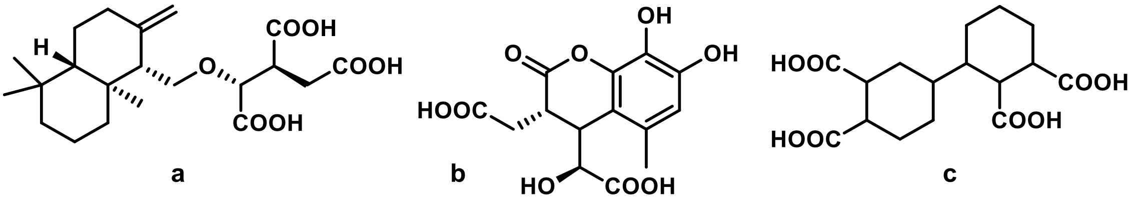

a b c

Figure S4: *Outlier compounds from Scifinder experiment one.*

Scifinder experiment two starts with two fused cyclic rings that have between 4 and 8 carbons in each ring. Initial searching gives 3238 hits, and then the experiment follows the same steps as Scifinder experiment one. Before the final filter that checks for data availability, there are 75 results. It should be noted at this point that entirely hypothetical compounds are found on Scifinder within this dataset, such as a CRAM discussed in a biogeochemical setting (**a**, Figure S5), that is given as a representative molecule with no associated data or discussion.[12] After filtering for compounds that have any data listed for ^{1}H NMR, ^{13}C NMR, MS, or IR experiments, only two compounds remain. These are highly bridged poly-ring molecules, that have little relevance to environmental CRAM (**b**[13] and **c**[14], Figure S5).

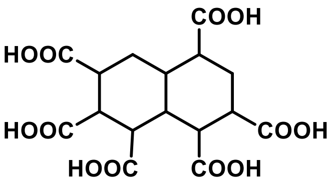

a

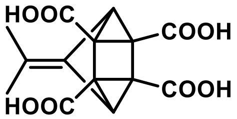

b

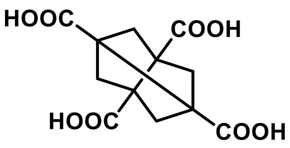

c

Figure S5: *Hits from Scifinder experiment two.*

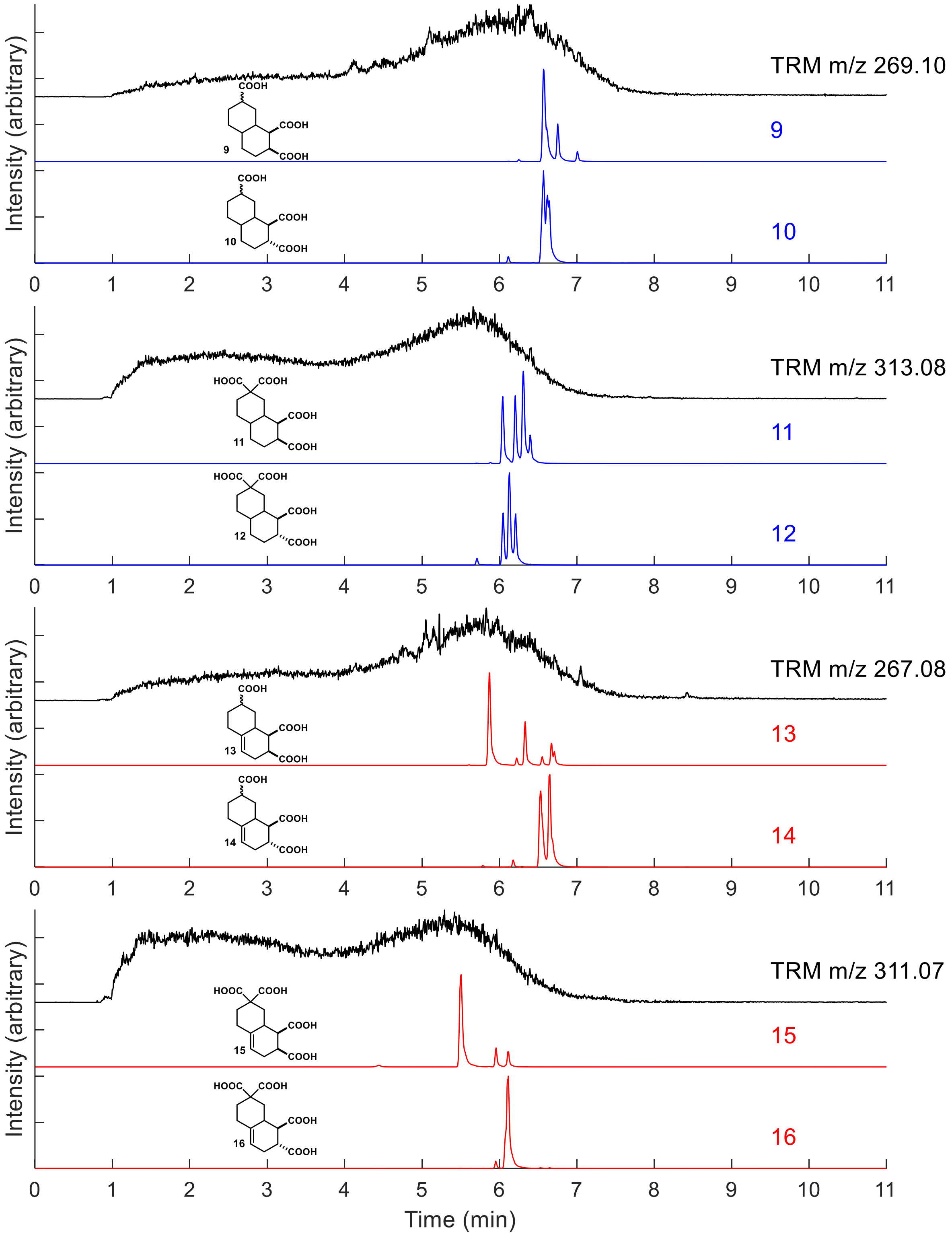

\

Figure S6: LCMS traces of synthesized compounds **9**-**16**, which are diastereomeric mixtures. Shown in each panel is the extracted ion chromatogram of the formula mass from the reference material TRM-0522 (black) along with the two synthesized compounds with the same mass.

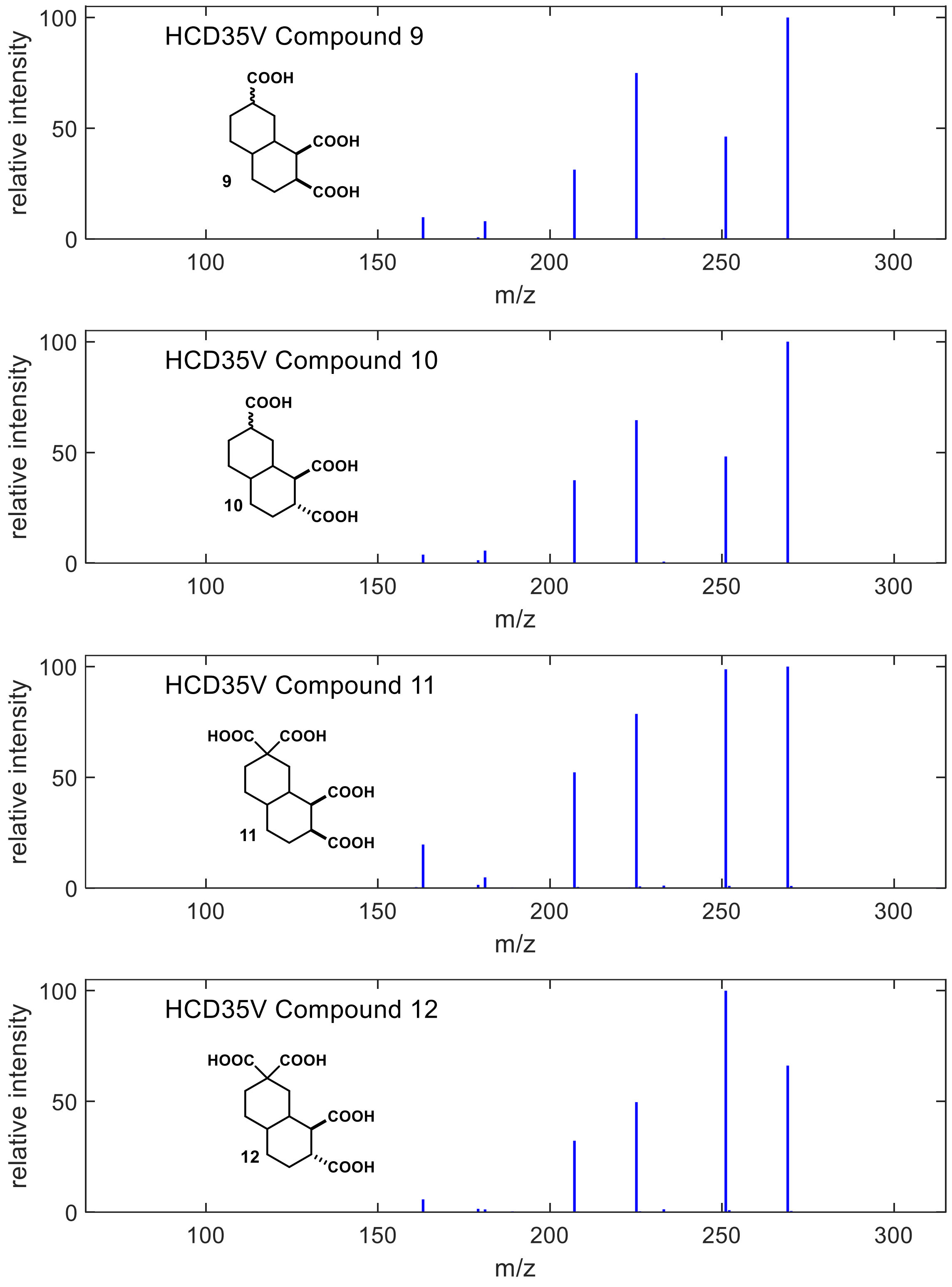

***Figure S7**:* HCD traces for compounds **9**-**12** at 35V relative energy**.**

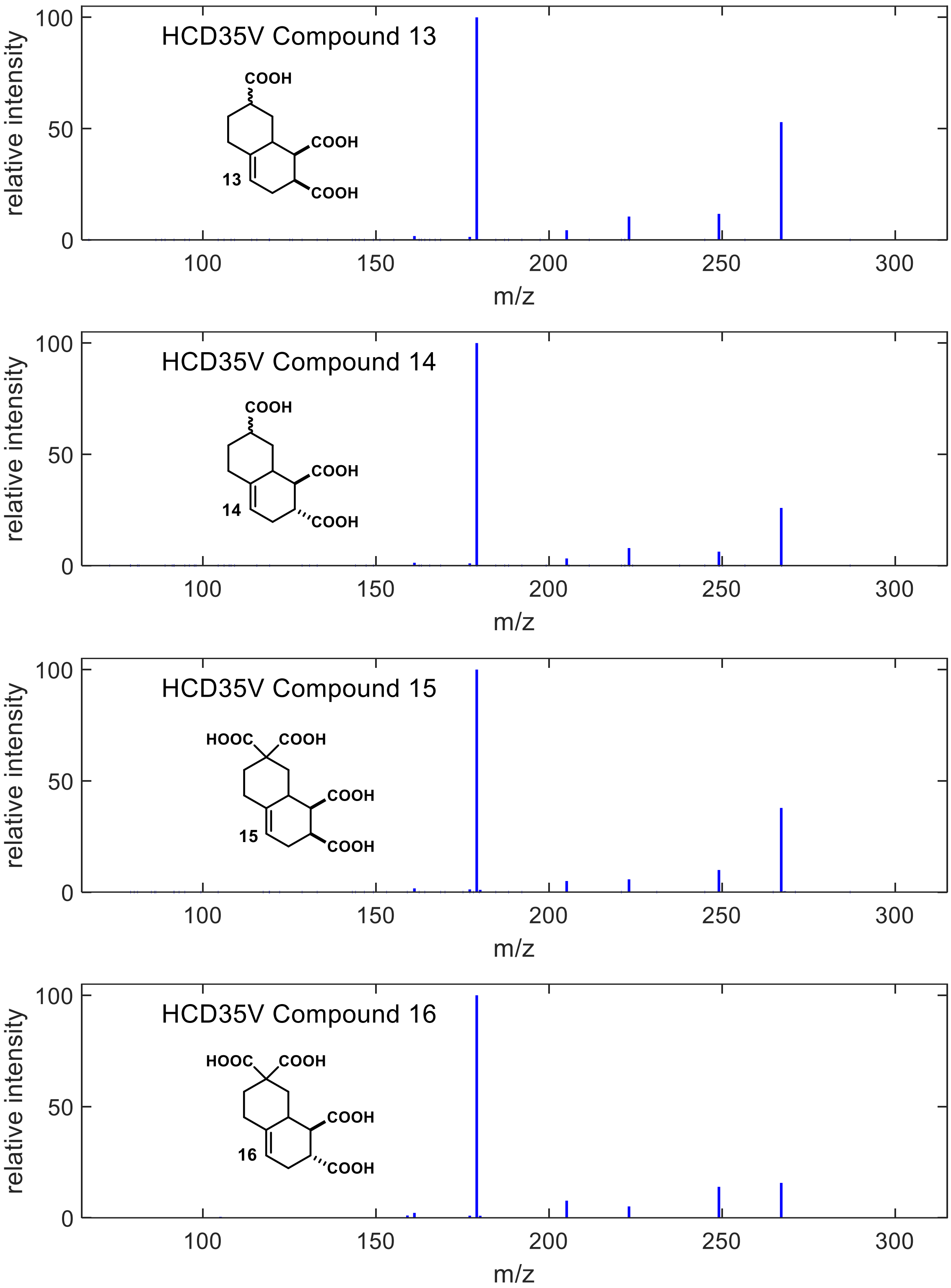

Figure S8: HCD traces for compounds **13-16 at 35V relative energy.**

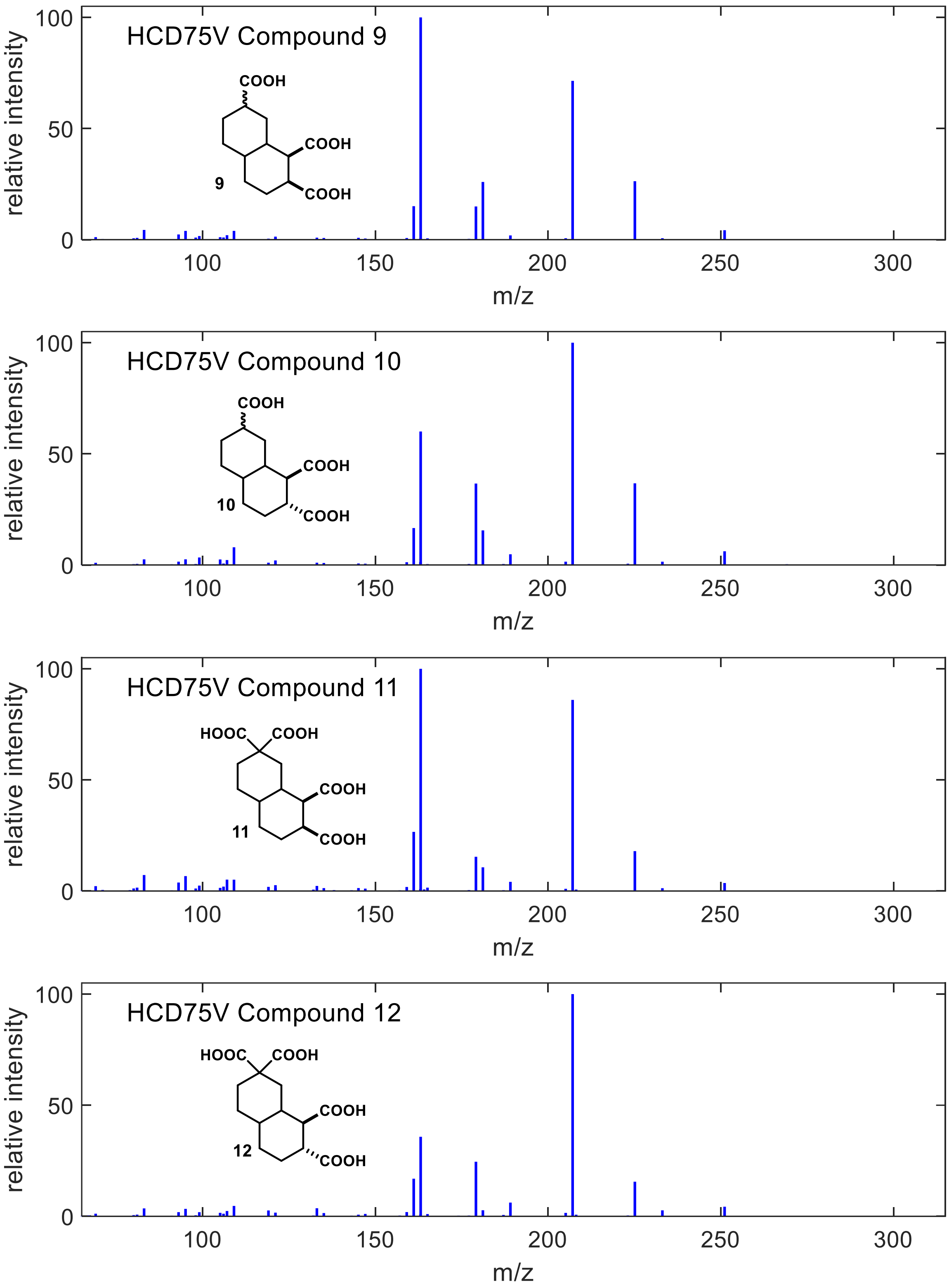

Figure S9: HCD traces for compounds **9-12.**

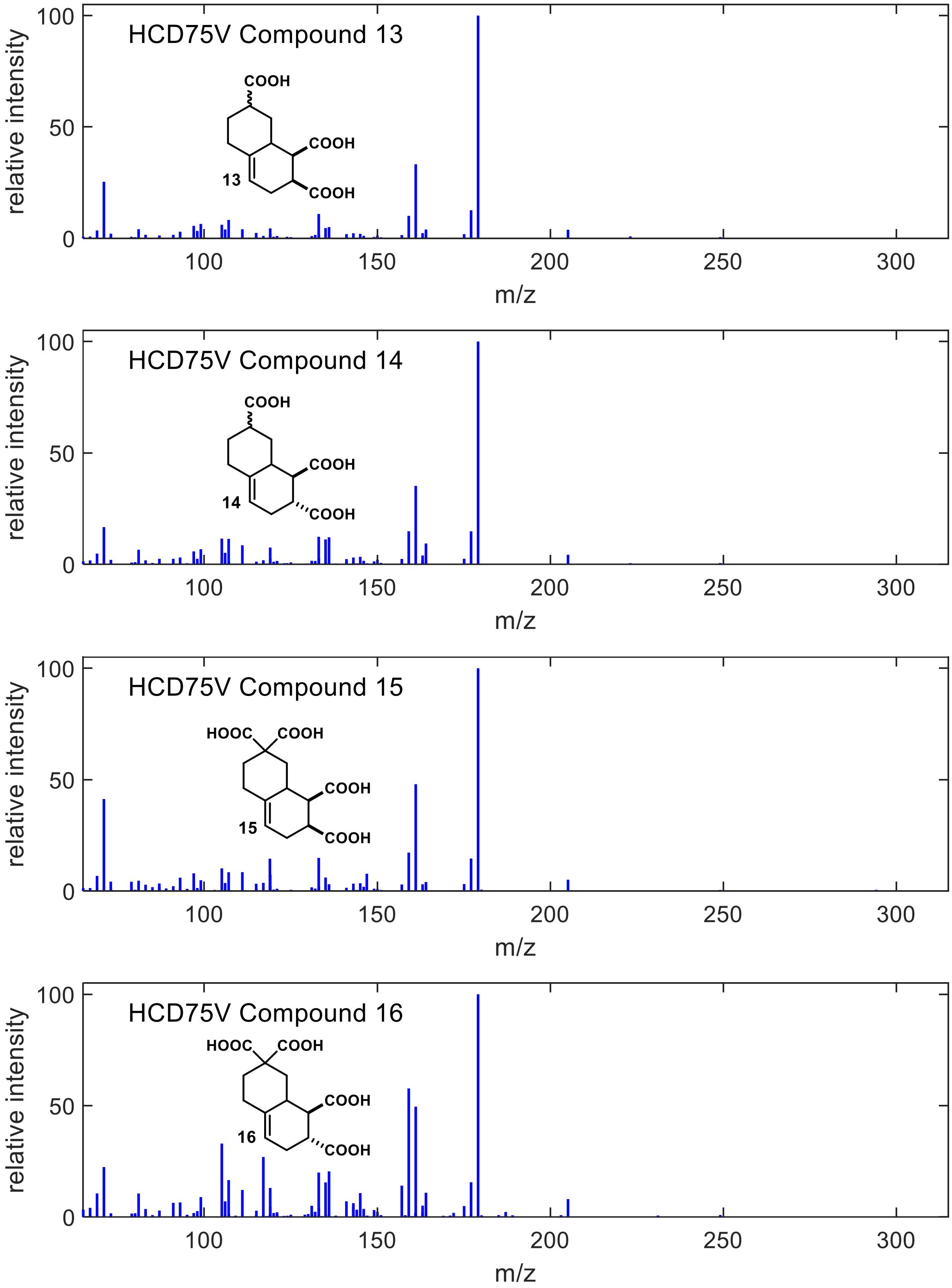

***Figure S10**: HCD traces for compounds **13**-**16**.*

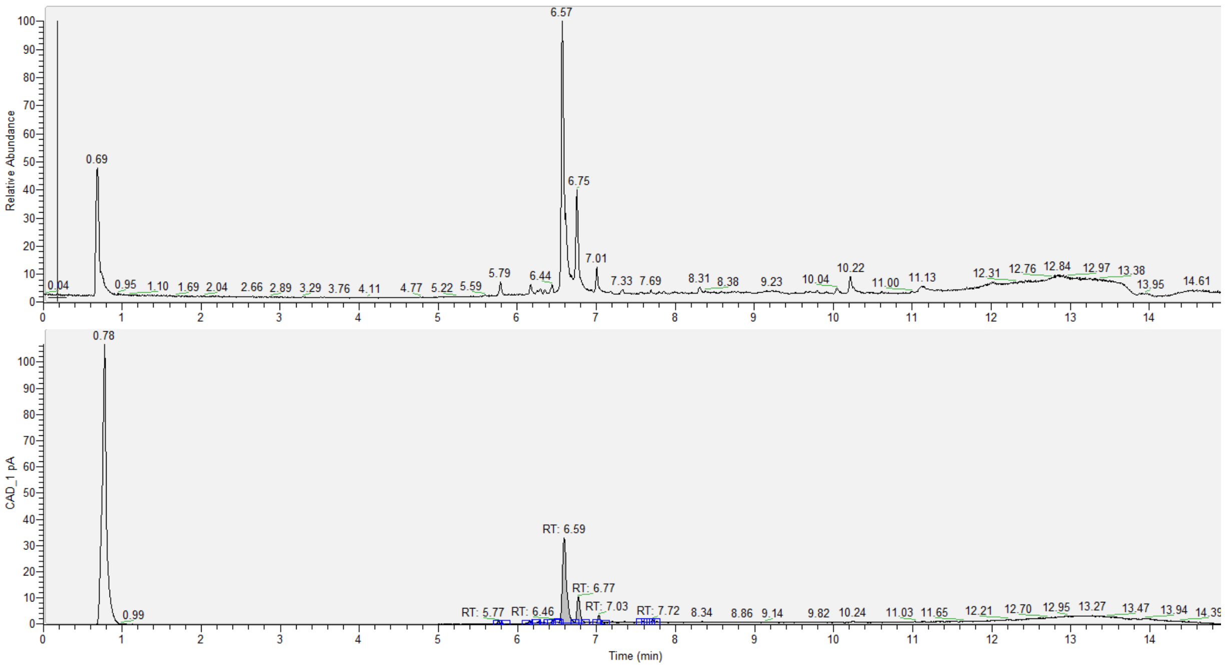

Figure S11: *TIC trace (top) and CID trace (bottom) of CRAM analogue **9**.*

Table S1: *LC-MS data and peak identities for CRAM analogue **9**.*

Apex RT	Start RT	End RT	Area	%Area	*m/z*	Identity
5.77	5.74	5.86	3.009	1.78	267	alkene precursor impurity
6.16	6.10	6.23	3.210	1.90	267	alkene precursor impurity
6.28	6.24	6.38	2.911	1.72	267	alkene precursor impurity
6.46	6.42	6.51	2.406	1.42	255	reduced carboxylate to alcohol impurity
6.59	6.52	6.72	117.313	69.64	269	title compound **9** isomer
6.77	6.73	6.86	29.328	17.53	269	title compound **9** isomer
7.03	7.00	7.10	8.048	4.94	269	title compound **9** isomer
7.72	7.68	7.75	1.773	1.05	225	decarboxylated title compound

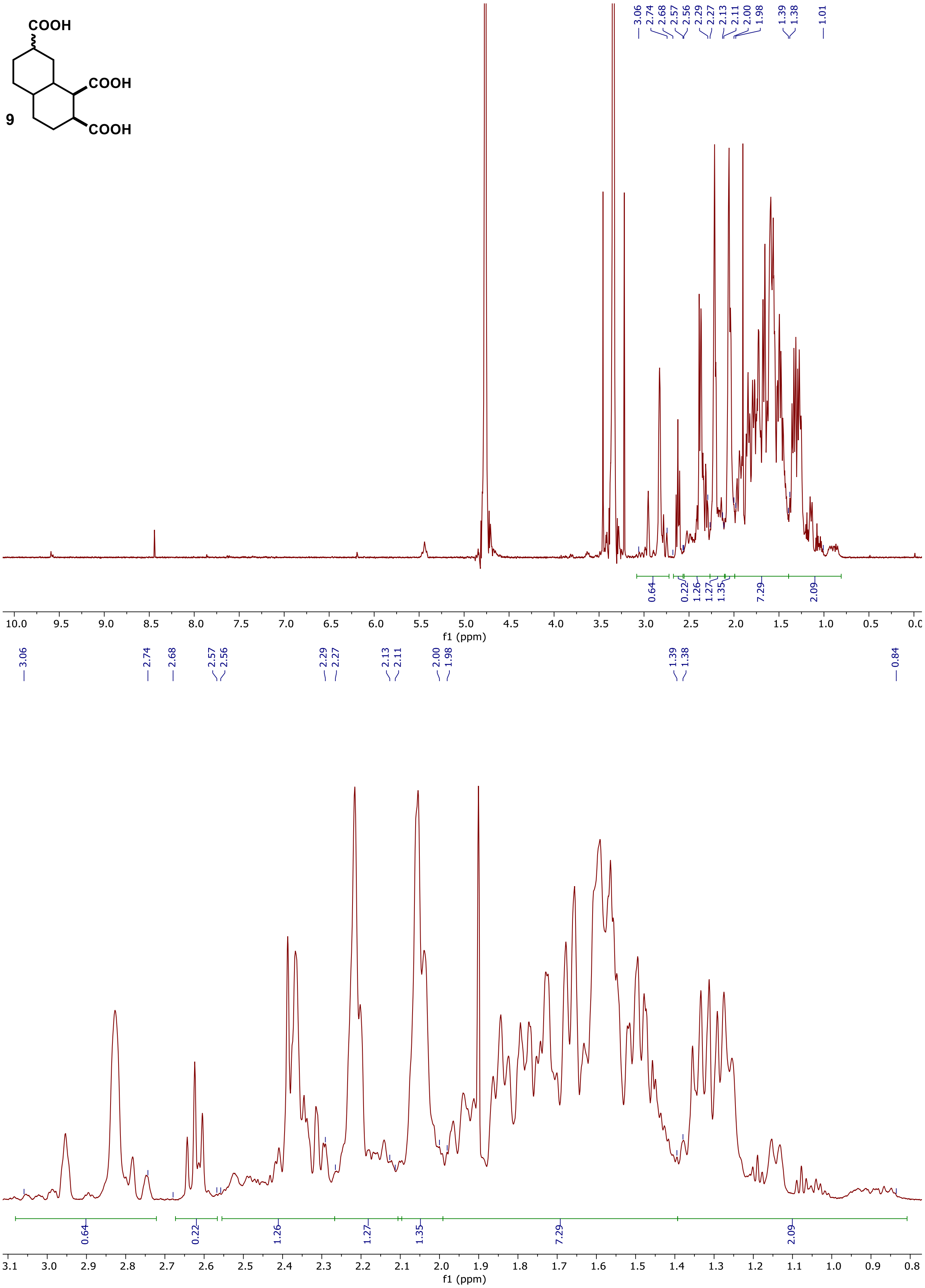

Figure S12: ^{1}H NMR spectra of compound **9** (600 MHz, 298K, 0.1M NaOD in D_2O)

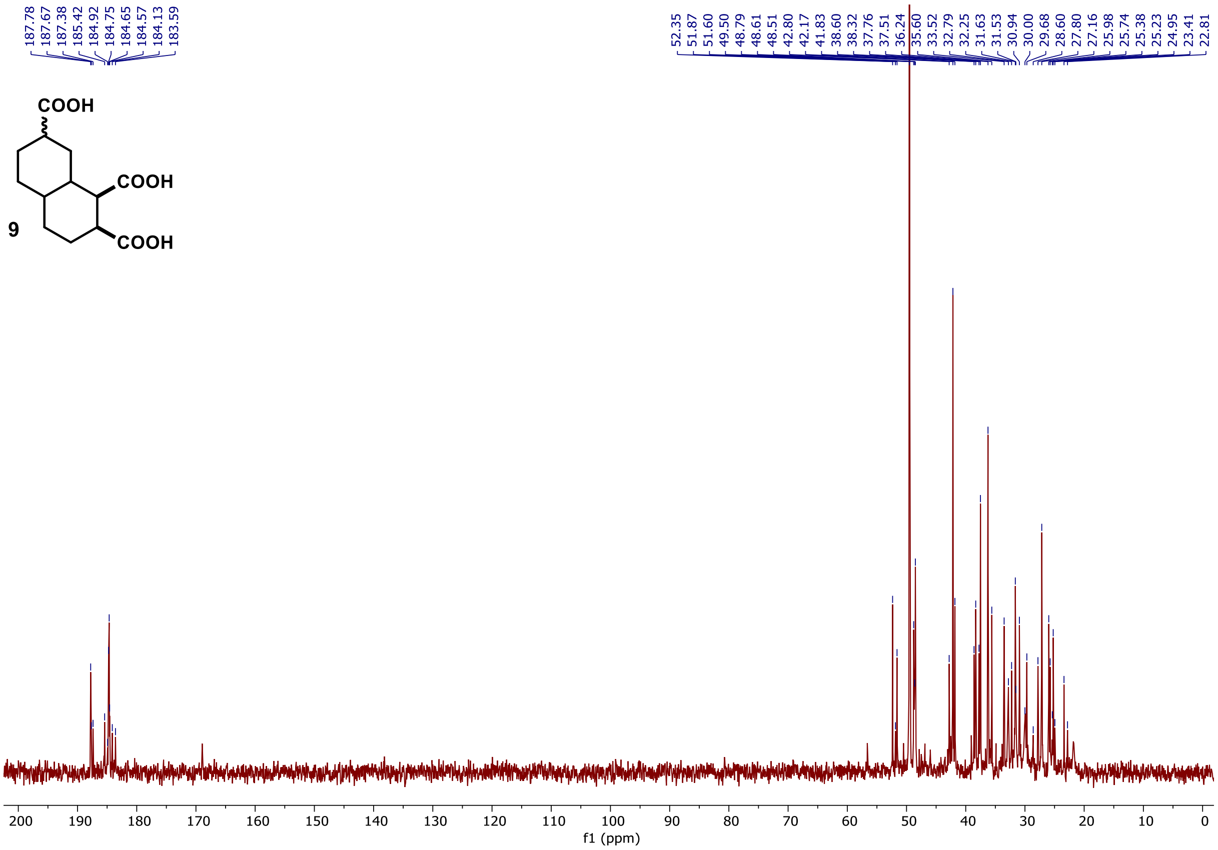
187.78
187.67
187.38
185.42
184.92
184.75
184.65
184.57
184.13
183.59
52.35
51.87
51.60
49.50
48.79
48.61
48.51
42.80
42.17
41.83
38.60
38.32
37.76
37.51
36.24
35.60
33.52
32.79
32.25
31.63
31.53
30.94
30.00
29.68
28.60
27.80
27.16
25.98
25.74
25.38
25.23
24.95
23.41
22.81
COOH
COOH
COOH
9
200 190 180 170 160 150 140 130 120 110 100 90 80 70 60 50 40 30 20 10 0
f1 (ppm)

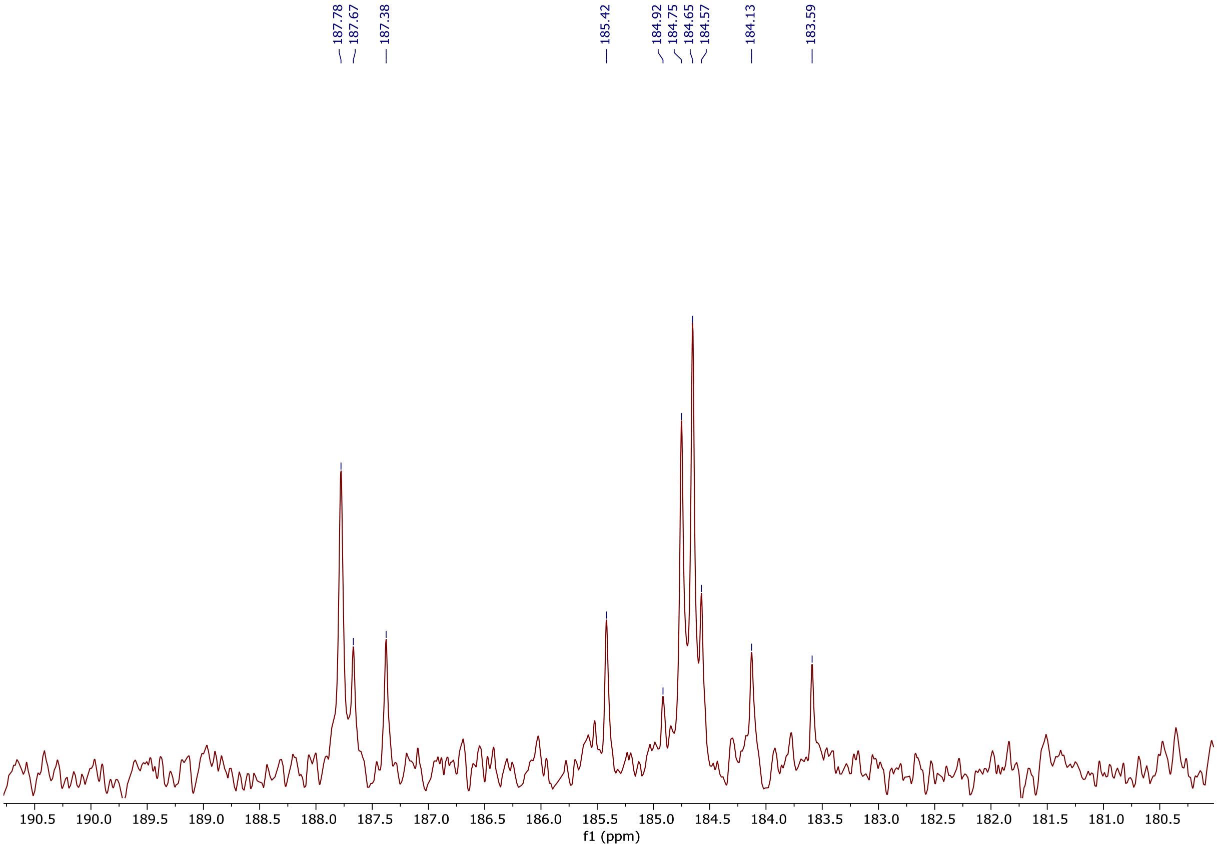
187.78
187.67
187.38
185.42
184.92
184.75
184.65
184.57
184.13
183.59
190.5 190.0 189.5 189.0 188.5 188.0 187.5 187.0 186.5 186.0 185.5 185.0 184.5 184.0 183.5 183.0 182.5 182.0 181.5 181.0 180.5
f1 (ppm)

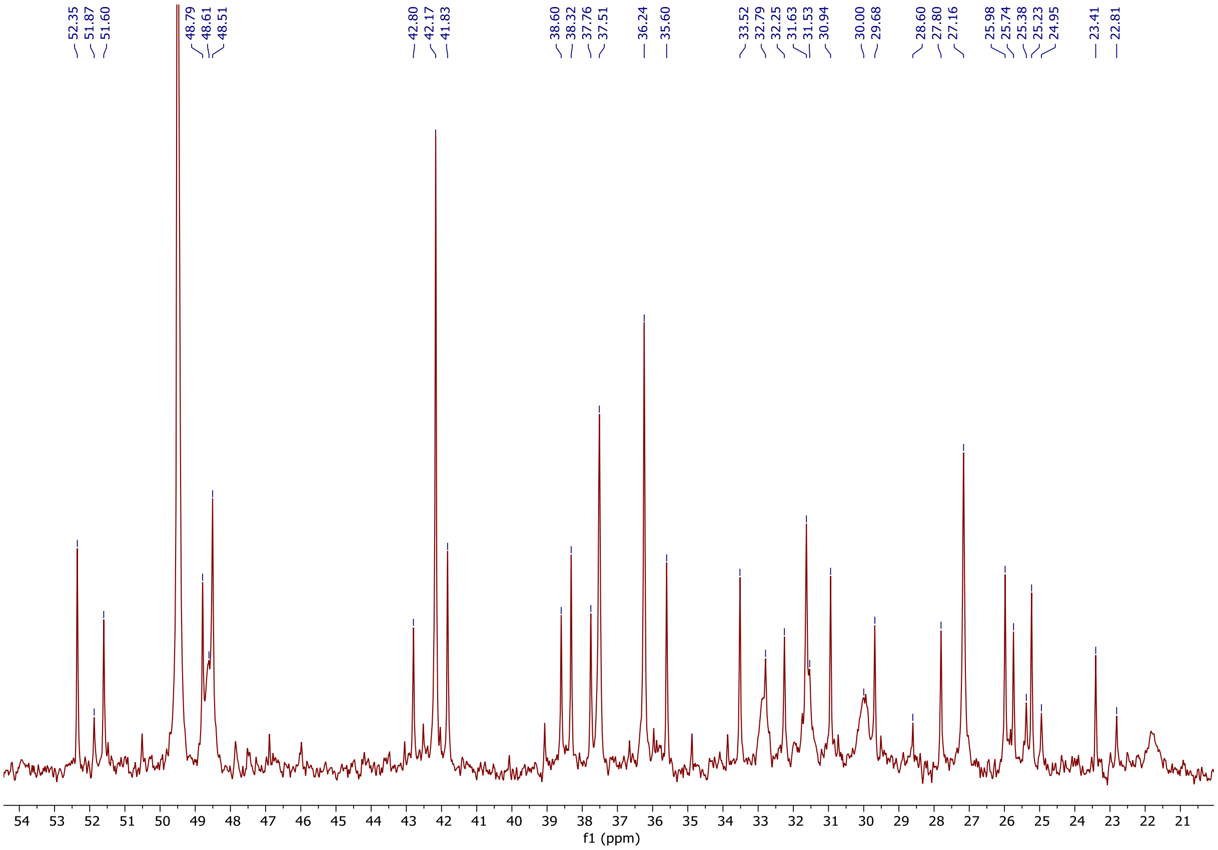

Figure S13: ^{13}C NMR spectra of compound **9** (151 MHz, 298K, 0.1M NaOD in D_2O)

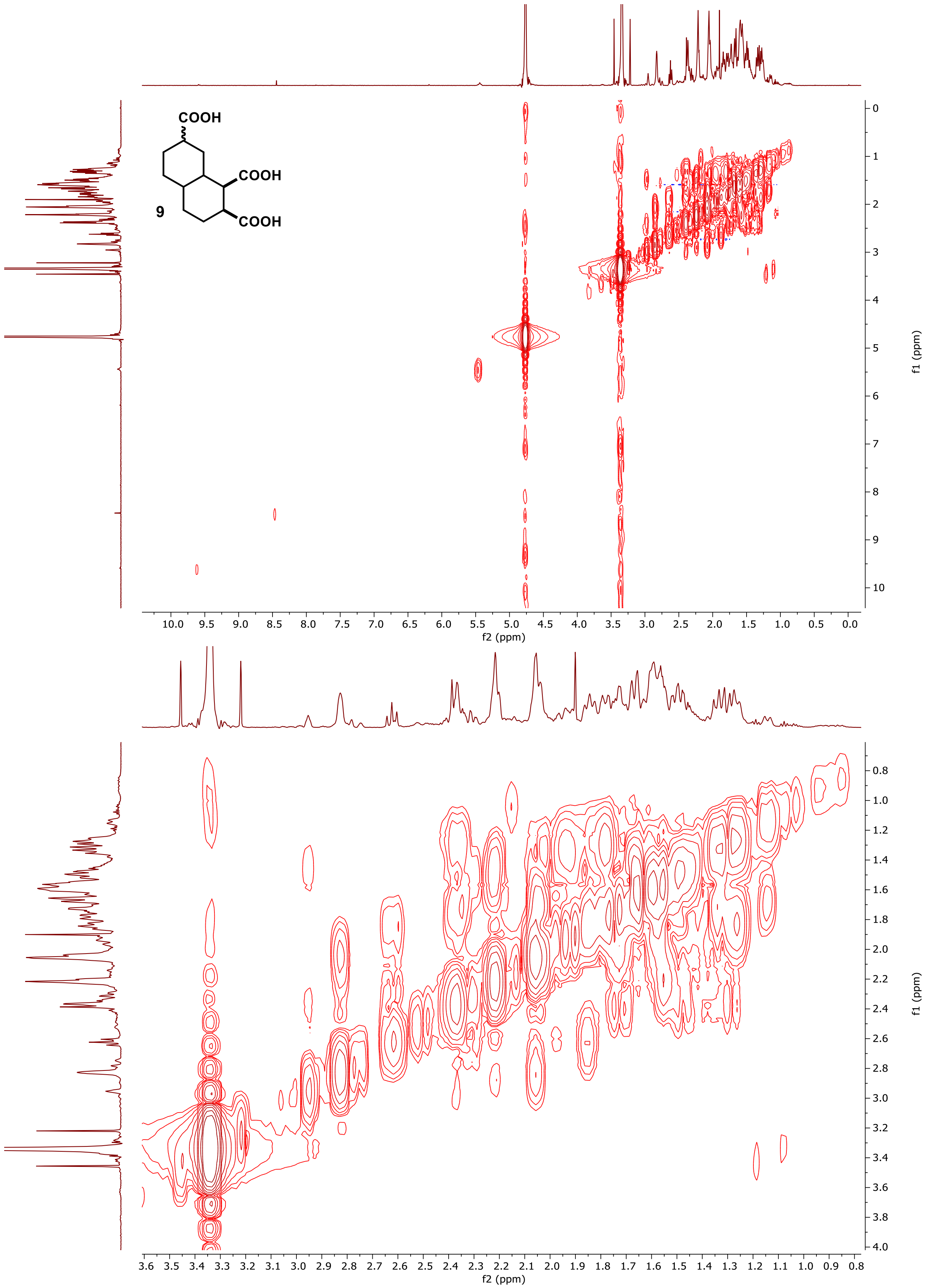

Figure S14: COSY spectra of compound **9** (600 MHz, 298K, 0.1M NaOD in D_2O)

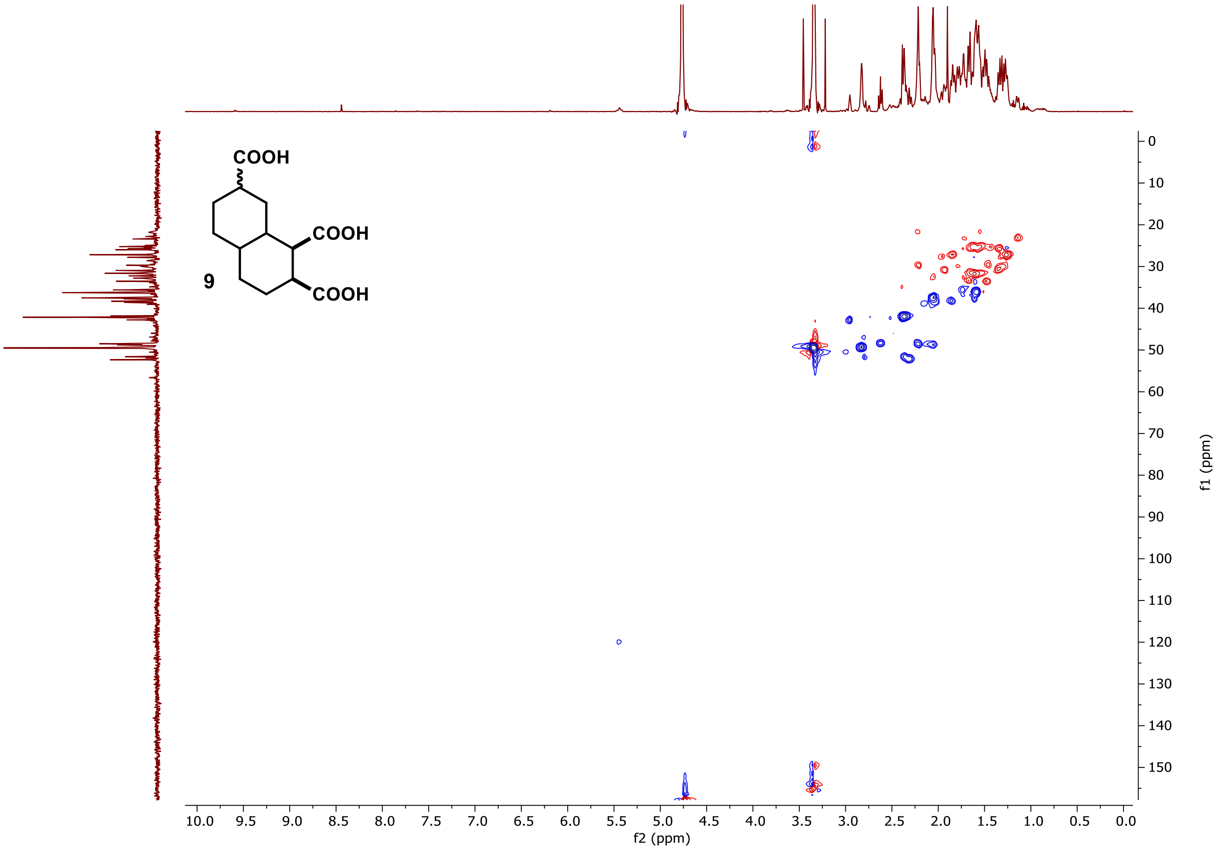

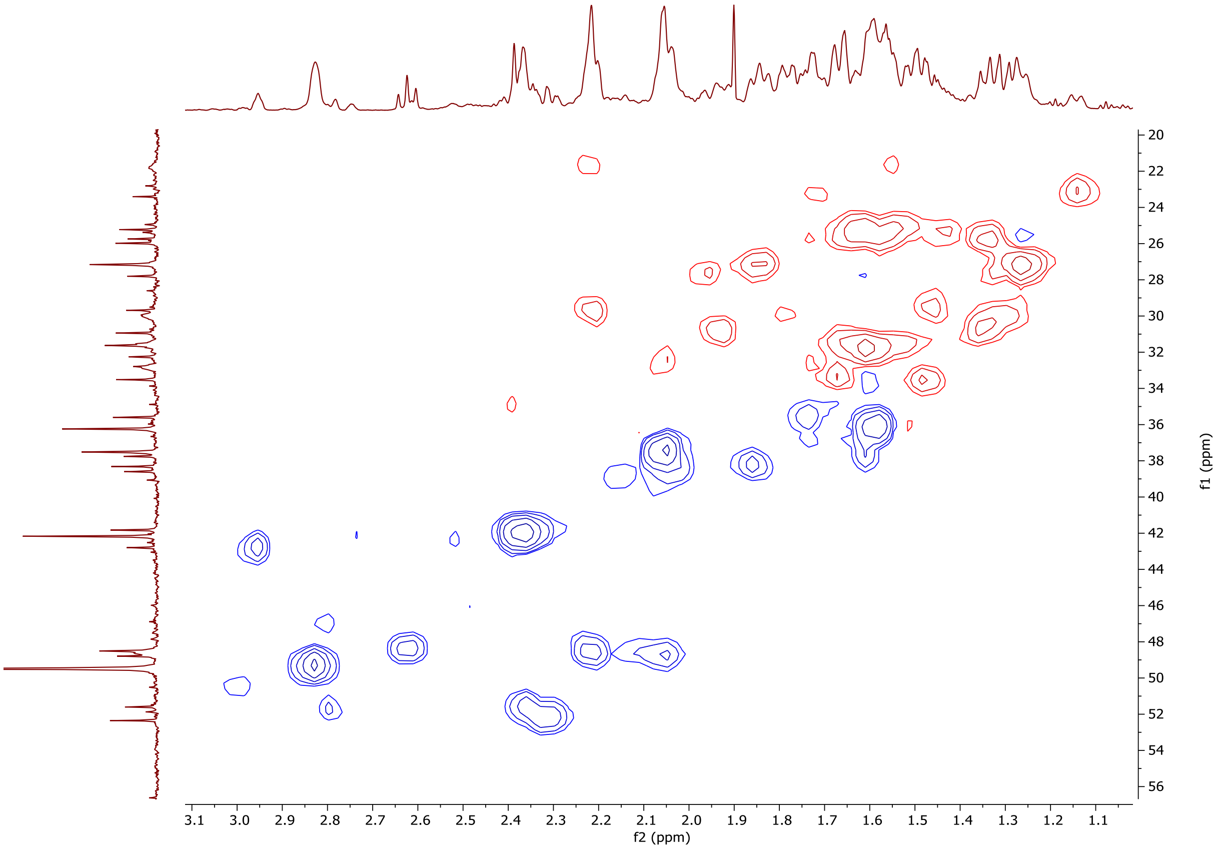

Figure S15: HSQC spectra of compound **9** (600 MHz, 298K, 0.1M NaOD in D_2O)

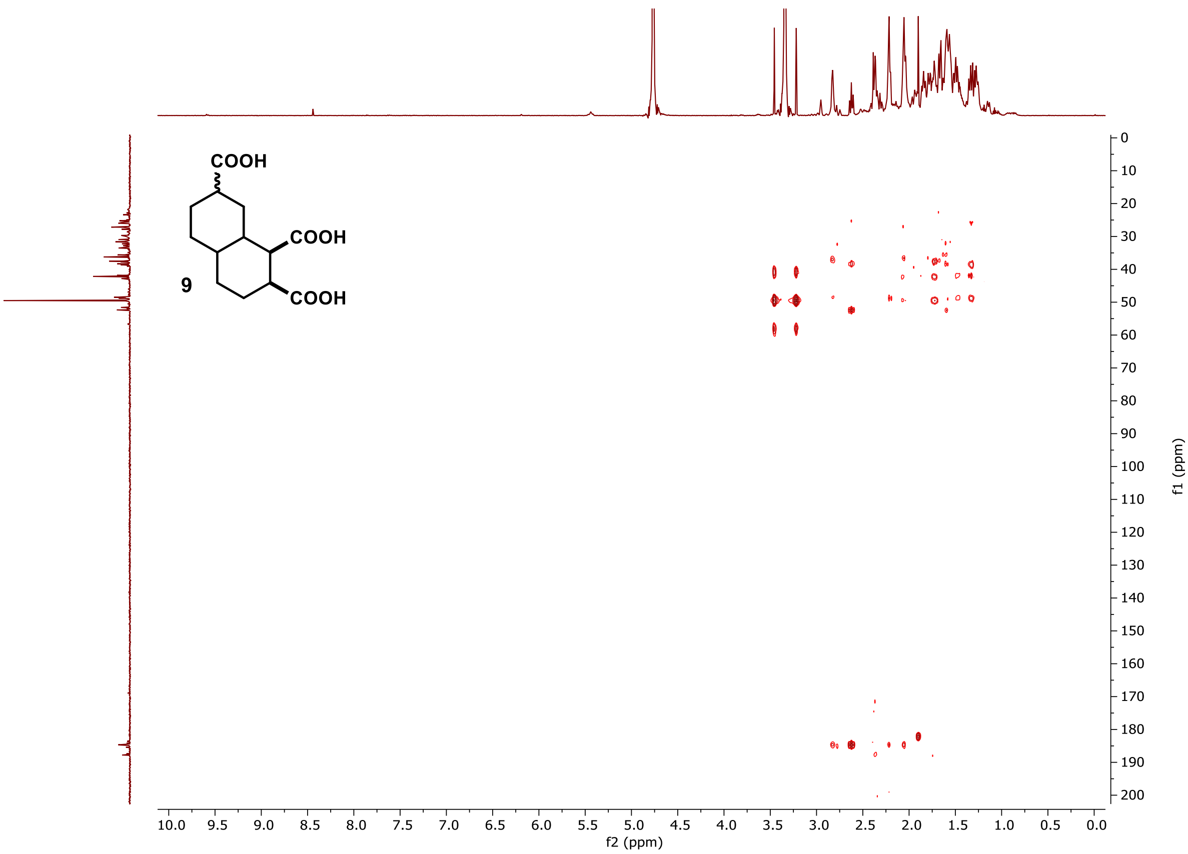
COOH
COOH
COOH
9
f2 (ppm)
f1 (ppm)

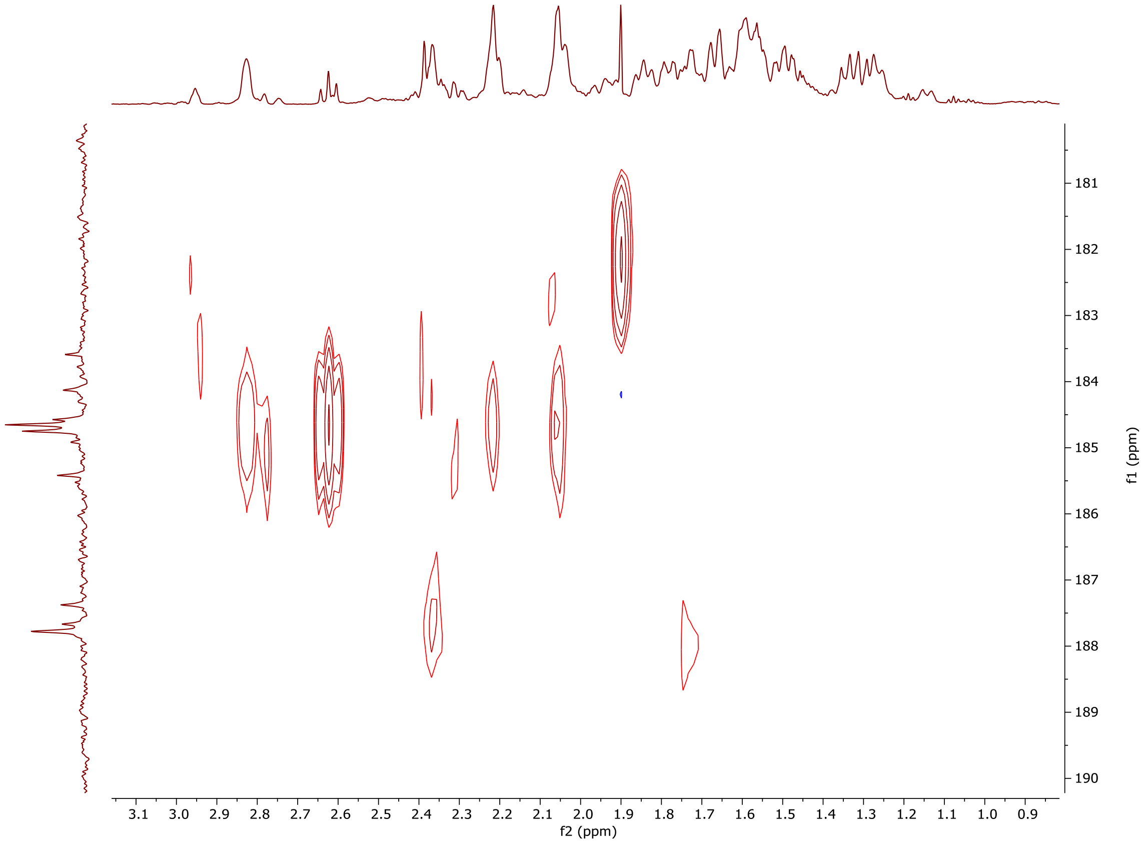
f2 (ppm)
f1 (ppm)

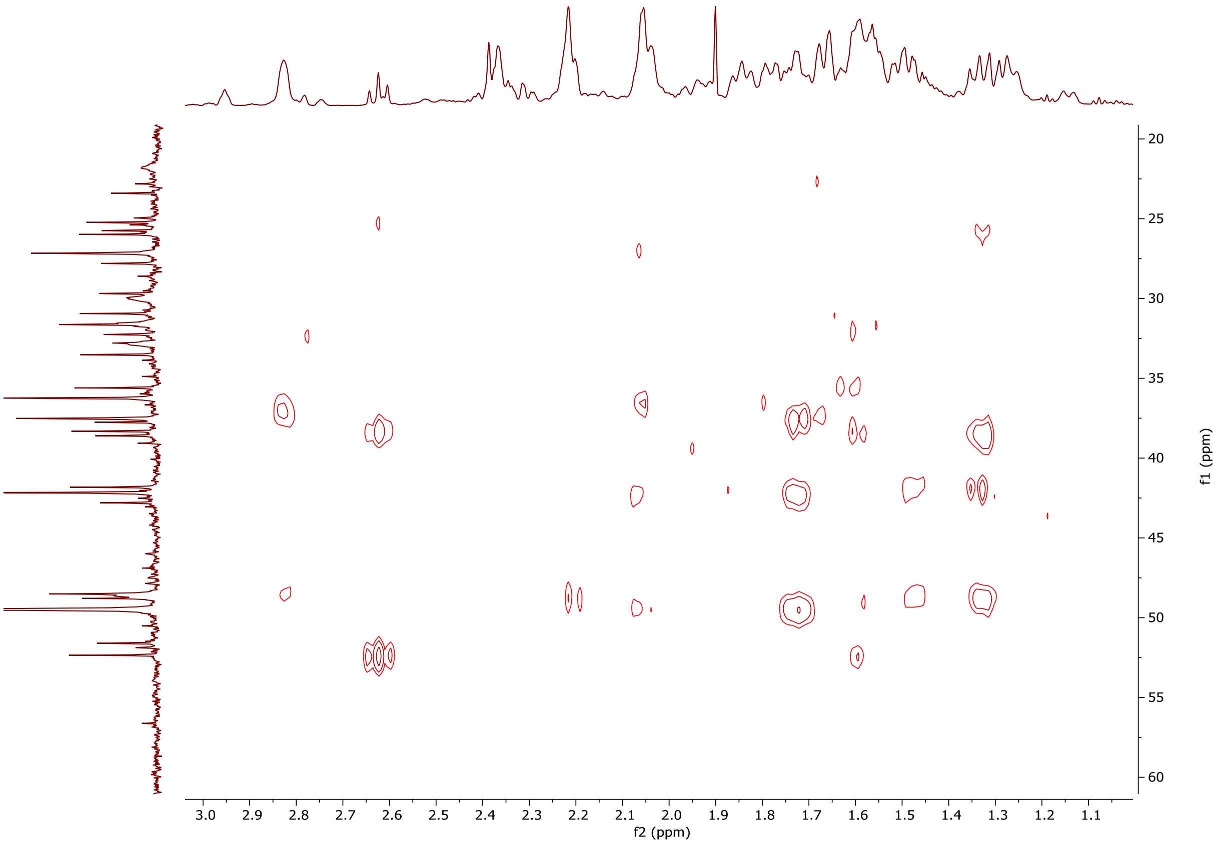

Figure S16: HMBC spectra of compound **9** (600 MHz, 298K, 0.1M NaOD in D_2O)

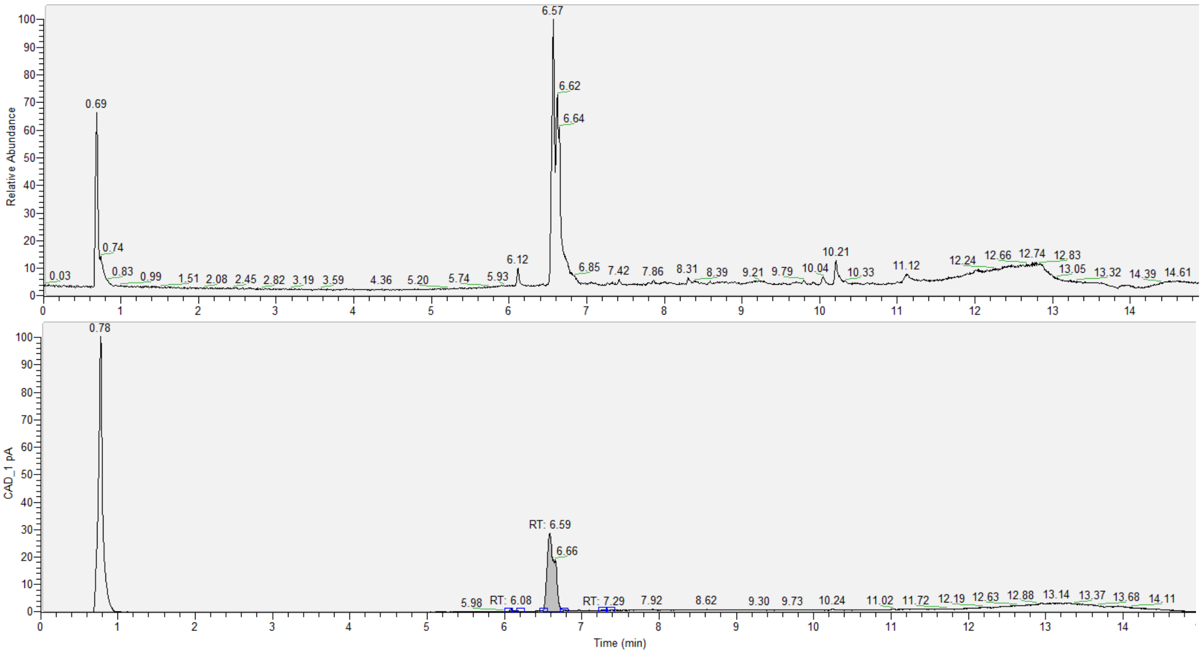

Figure S17: *TIC trace (top) and CID trace (bottom) of CRAM analogue **10**.*

Table S2: *LC-MS data and peak identities for CRAM analogue **10**.*

Apex RT	Start RT	End RT	Area	%Area	*m/z*	Identity
6.26	6.20	6.33	1.487	1.19	269	title compound **10** isomer
6.68	6.60	7.00	122.467	97.71	269	Overlapped title compound **10** isomers
7.34	7.29	7.44	0.442	0.35	297	unidentified impurity

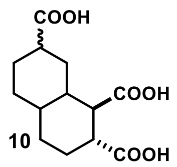

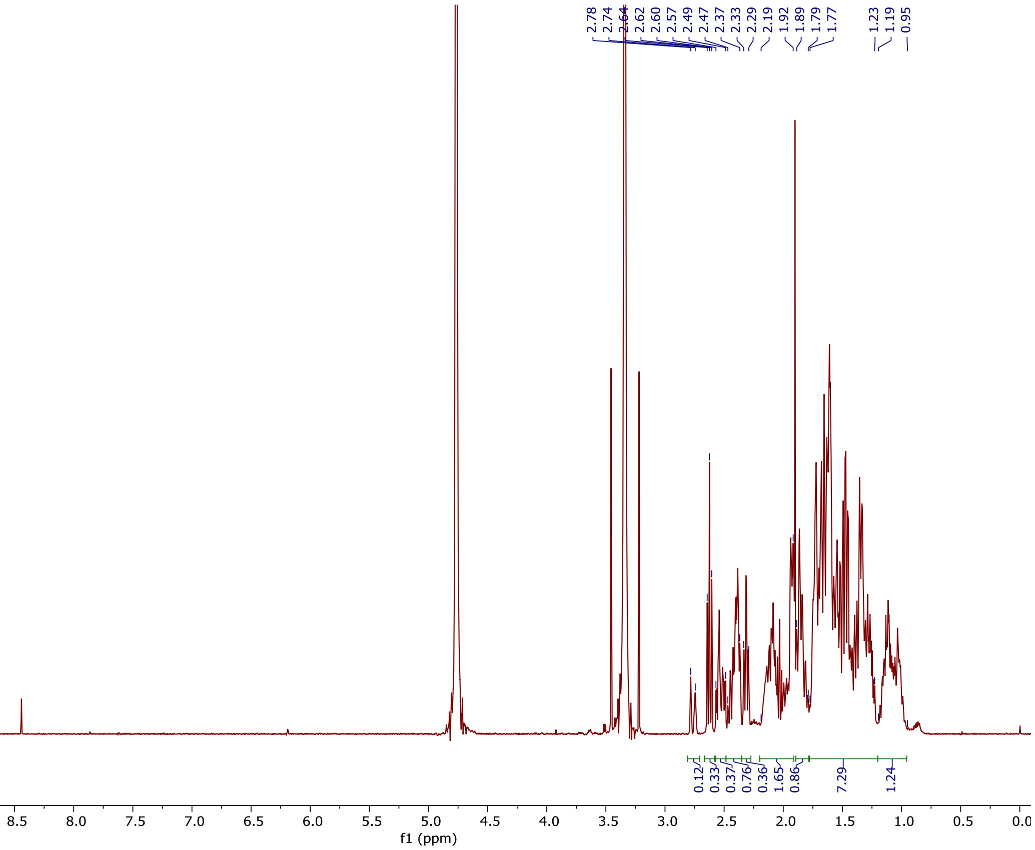

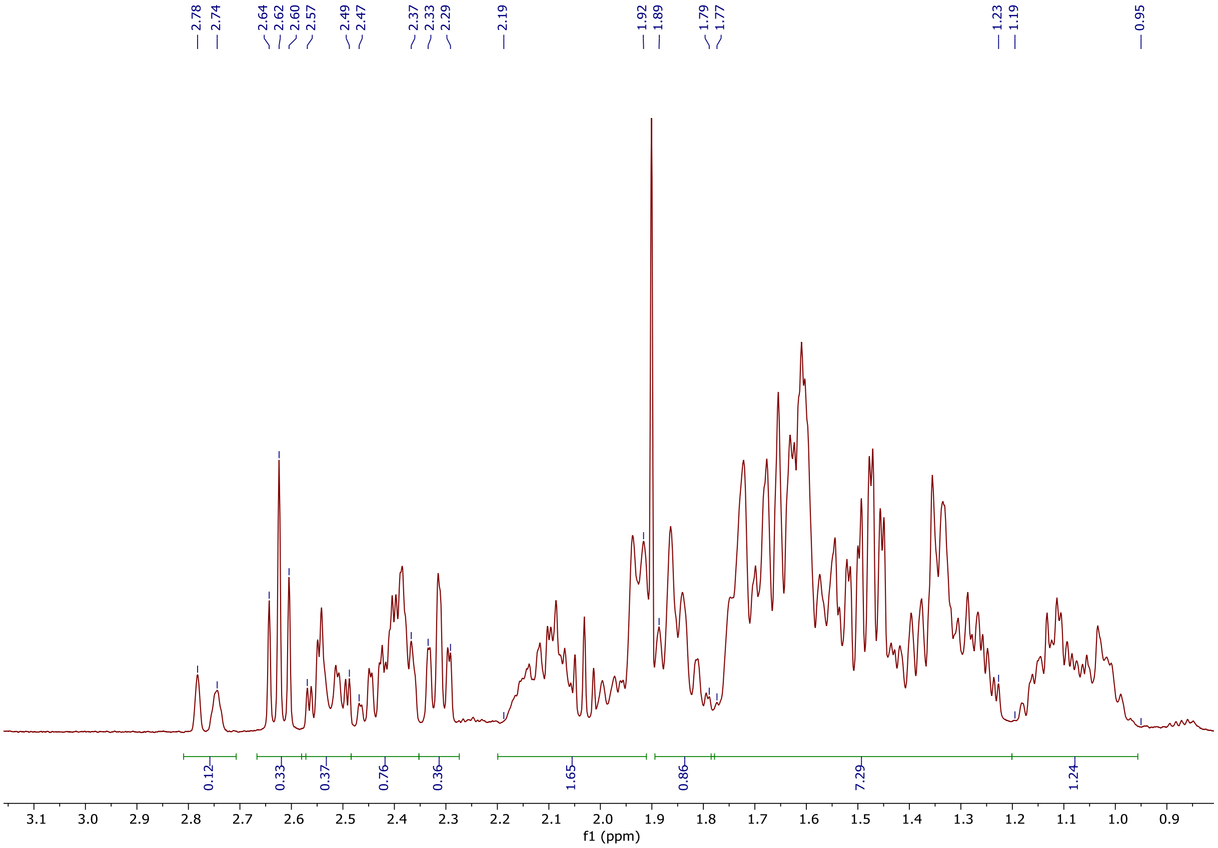

Figure S18: ^{1}H NMR spectra of compound **10** (600 MHz, 298K, 0.1M NaOD in D_2O)

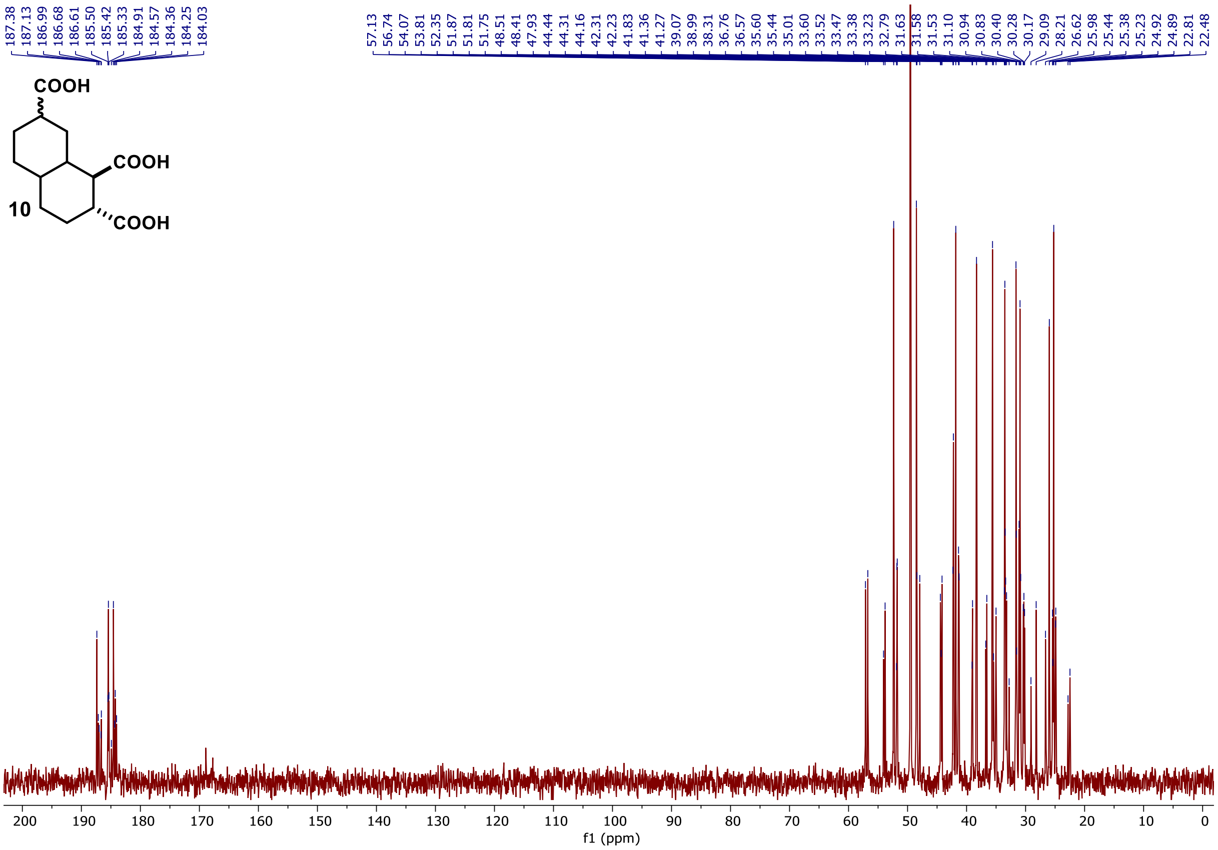

187.38
187.13
186.99
186.68
186.61
185.50
185.42
185.33
184.91
184.57
184.36
184.25
184.03
57.13
56.74
54.07
53.81
52.35
51.87
51.81
51.75
48.51
48.41
47.93
44.44
44.31
44.16
42.31
42.23
41.83
41.36
41.27
39.07
38.99
38.31
36.76
36.57
35.60
35.44
35.01
33.60
33.52
33.47
33.38
33.23
32.79
31.63
31.58
31.53
31.10
30.94
30.83
30.40
30.28
30.17
29.09
28.21
26.62
25.98
25.44
25.38
25.23
24.92
24.89
22.81
22.48
COOH
COOH
10
COOH
200 190 180 170 160 150 140 130 120 110 100 90 80 70 60 50 40 30 20 10 0
f1 (ppm)

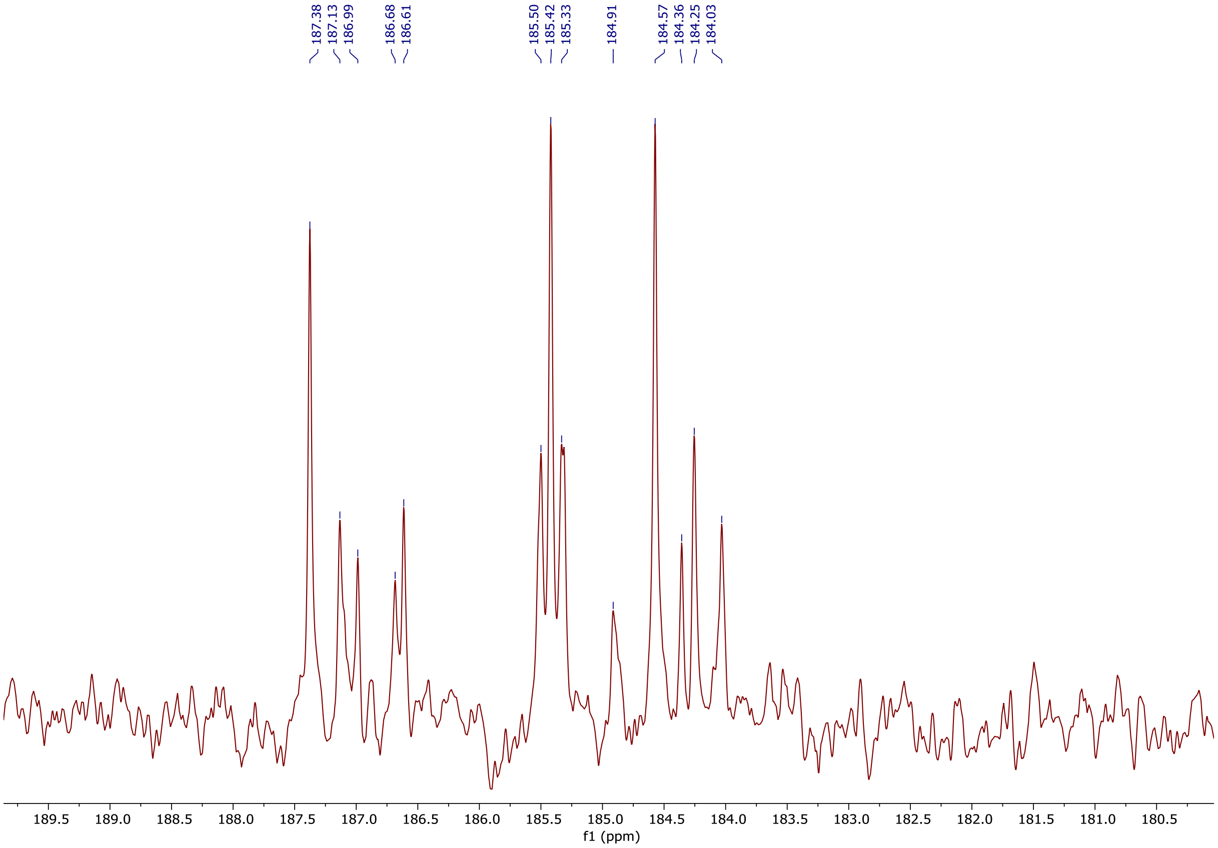

187.38
187.13
186.99
186.68
186.61
185.50
185.42
185.33
184.91
184.57
184.36
184.25
184.03
189.5 189.0 188.5 188.0 187.5 187.0 186.5 186.0 185.5 185.0 184.5 184.0 183.5 183.0 182.5 182.0 181.5 181.0 180.5
f1 (ppm)

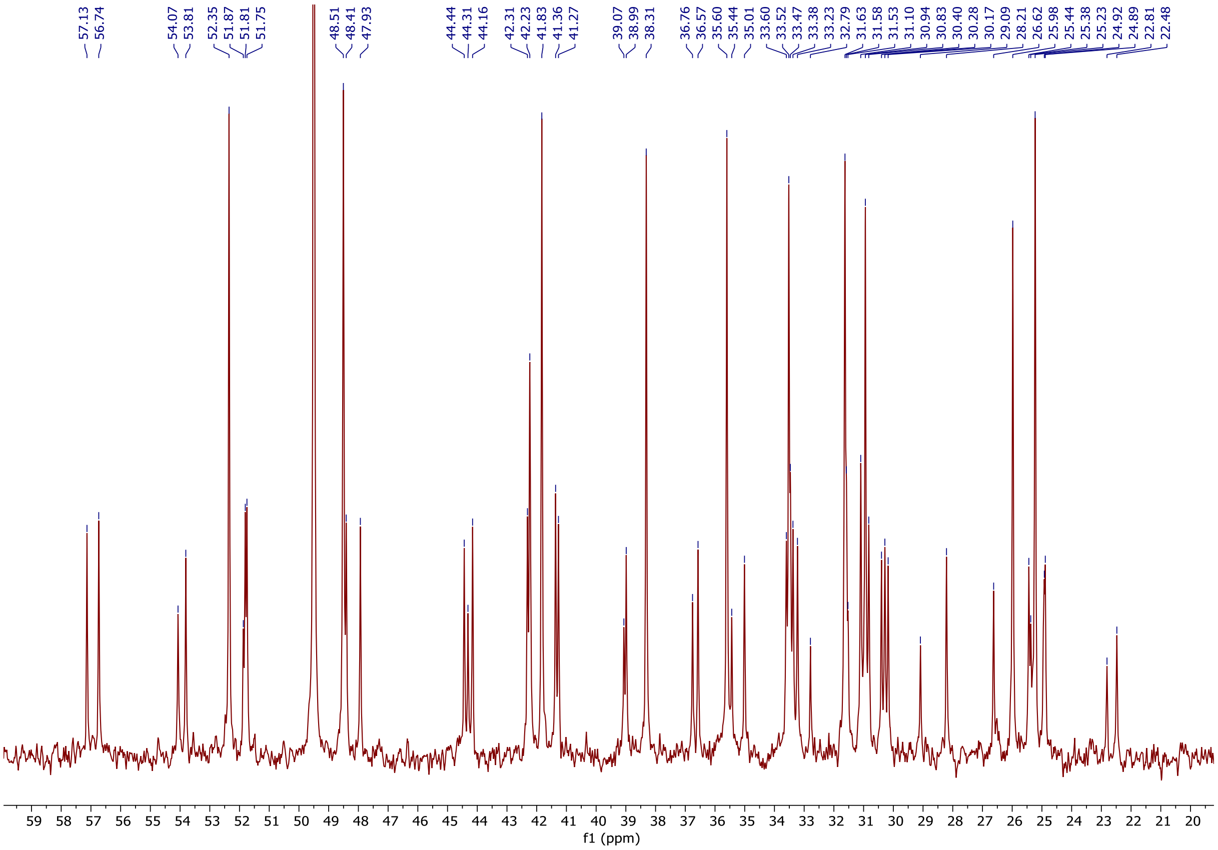

Figure S19: ^{13}C NMR spectra of compound **10** (151 MHz, 298K, 0.1M NaOD in D_2O)

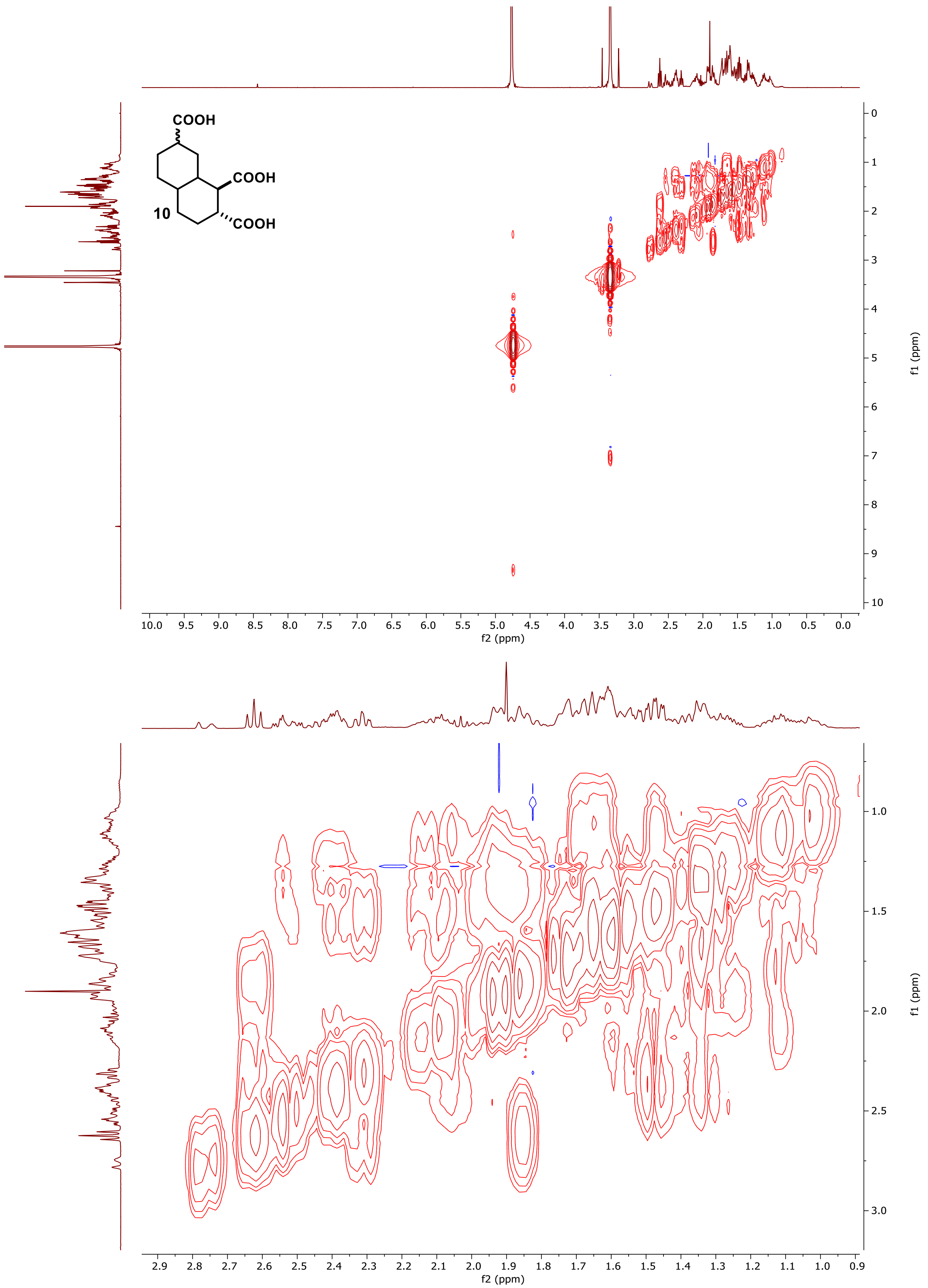

Figure S20: COSY spectra of compound **10** (600 MHz, 298K, 0.1M NaOD in D_2O)

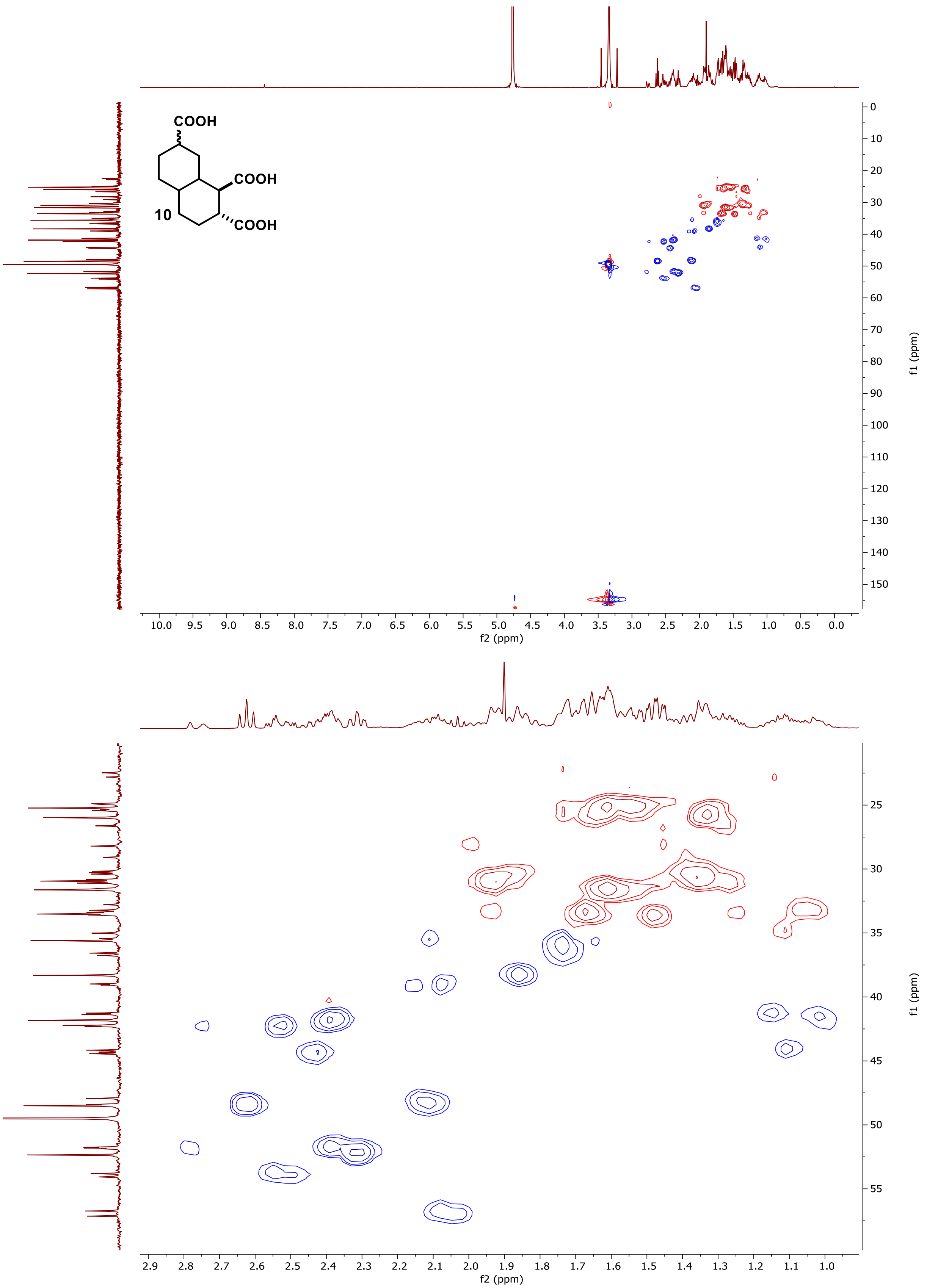

Figure S21: HSQC spectra of compound **10** (600 MHz, 298K, 0.1M NaOD in D_2O)

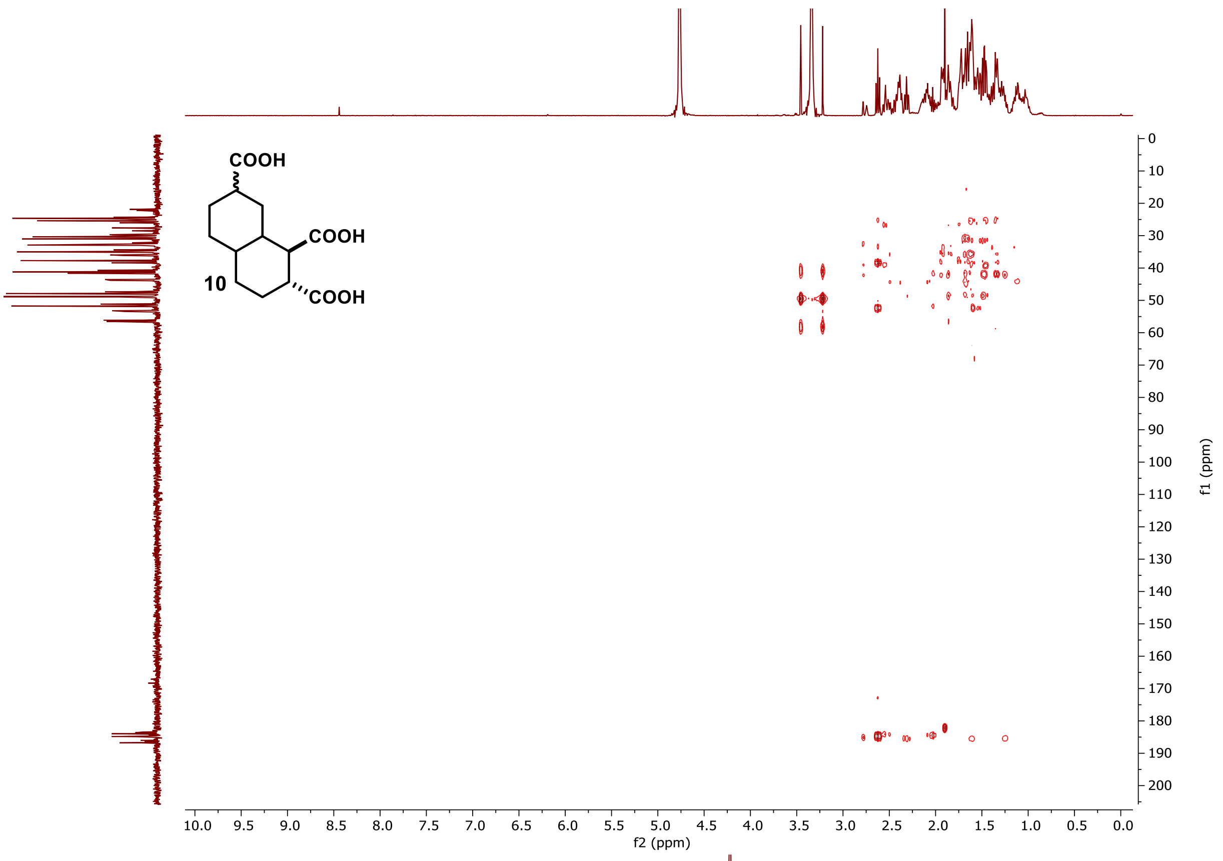
COOH
COOH
10
COOH
f2 (ppm)
f1 (ppm)

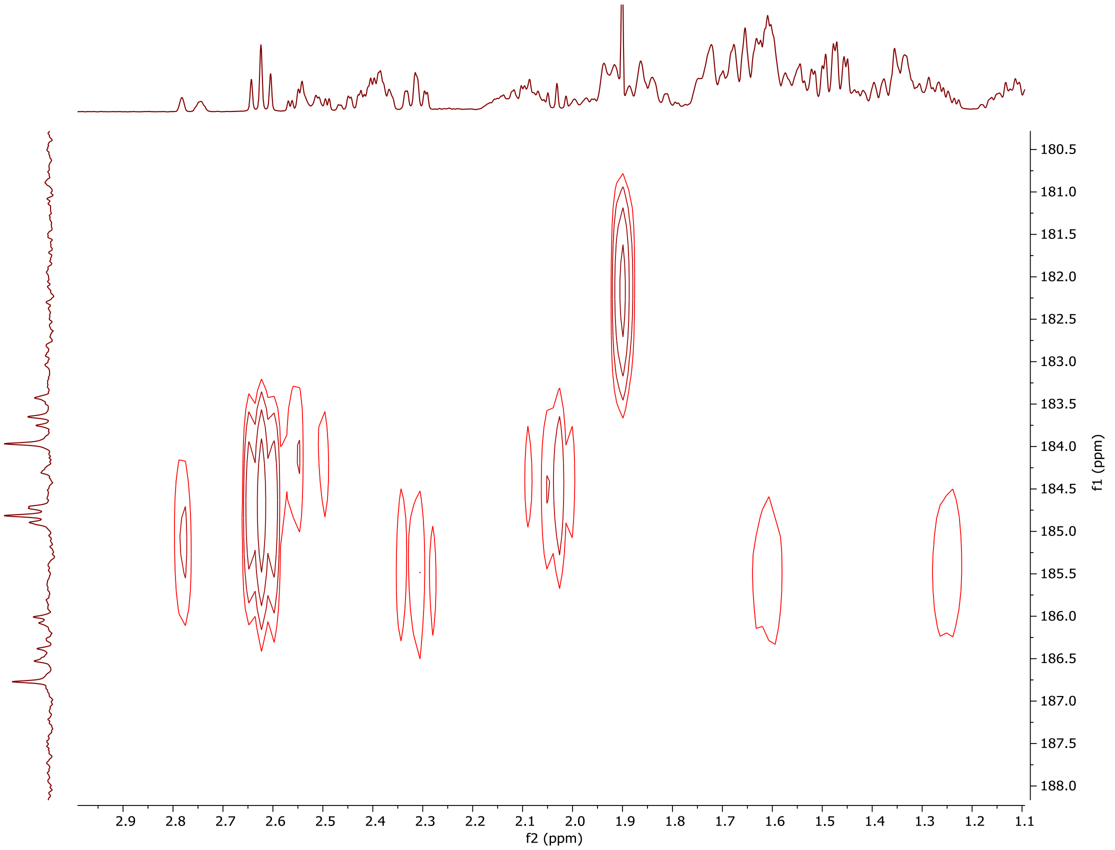
f2 (ppm)
f1 (ppm)

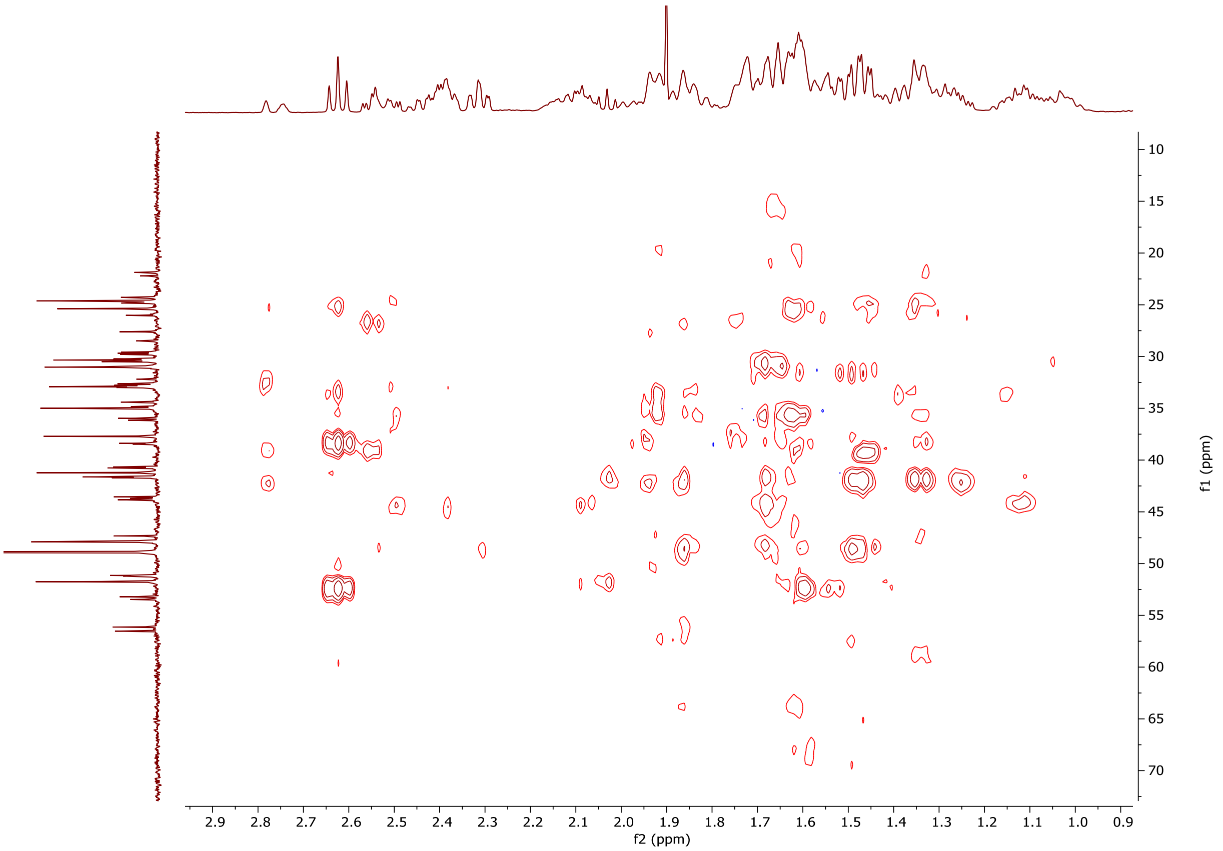

Figure S22: HMBC spectra of compound **10** (600 MHz, 298K, 0.1M NaOD in D_2O)

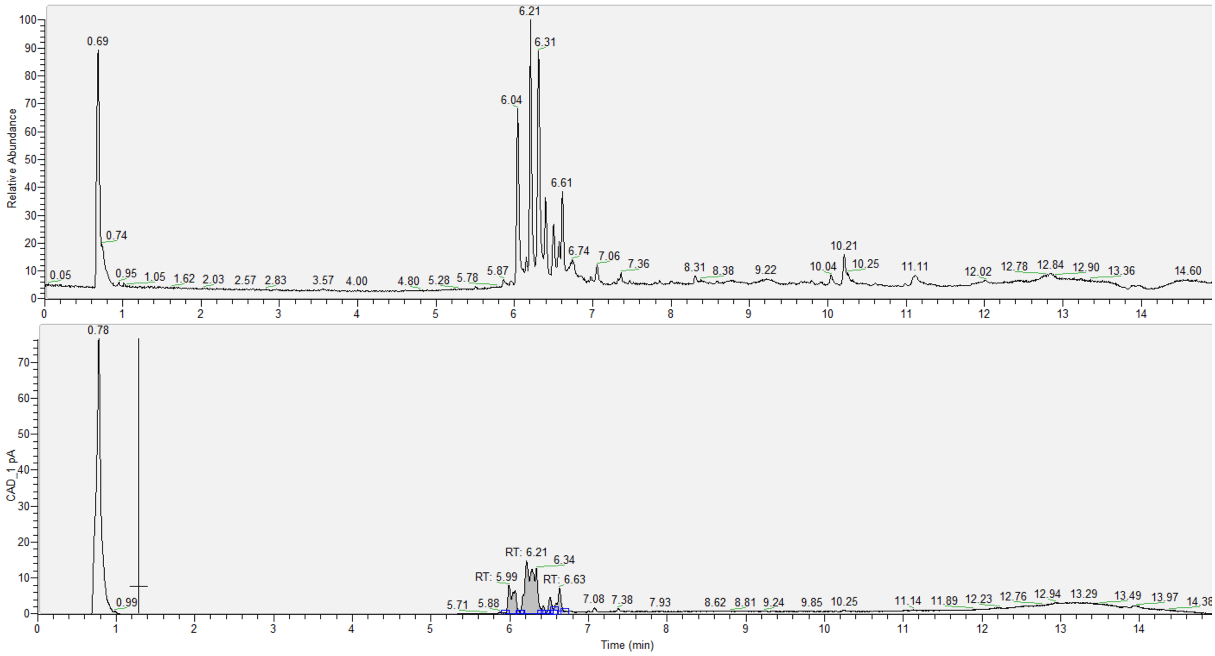

Figure S23: *TIC trace (top) and CID trace (bottom) of CRAM analogue **11**.*

Table S3: *LC-MS data and peak identities for CRAM analogue **11**.*

Apex RT	Start RT	End RT	Area	%Area	*m/z*	Identity
5.99	5.95	6.13	42.099	20.04	313	title compound **11** isomer
6.21	6.14	6.40	130.228	61.99	313	title compound **11** isomer
6.43	6.40	6.46	5.349	2.55	313	title compound **11** isomer
6.51	6.47	6.56	13.382	6.37	313	title compound **11** isomer
6.63	6.57	6.69	19.009	9.05	269	decarboxylated impurity

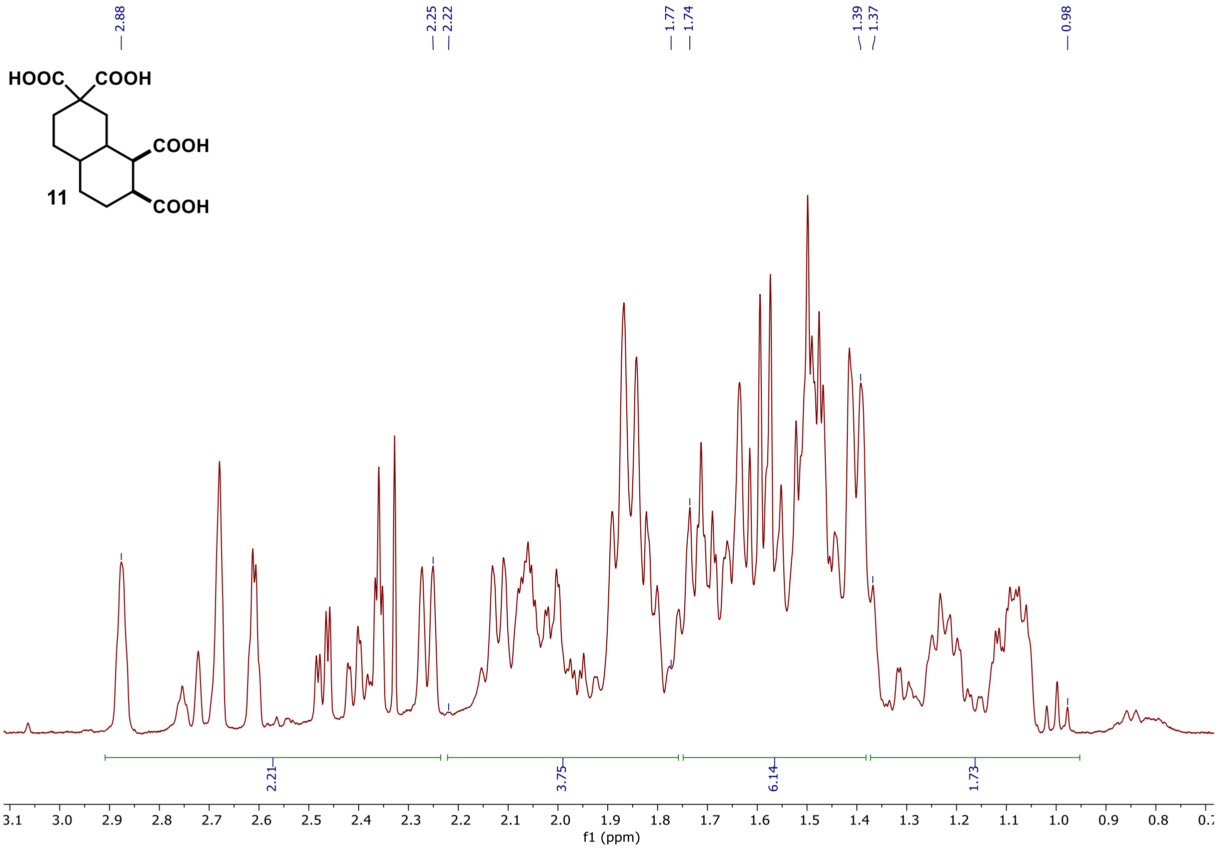

Figure S24: ^{1}H NMR spectra of compound **11** (600 MHz, 298K, 0.1M NaOD in D_2O)

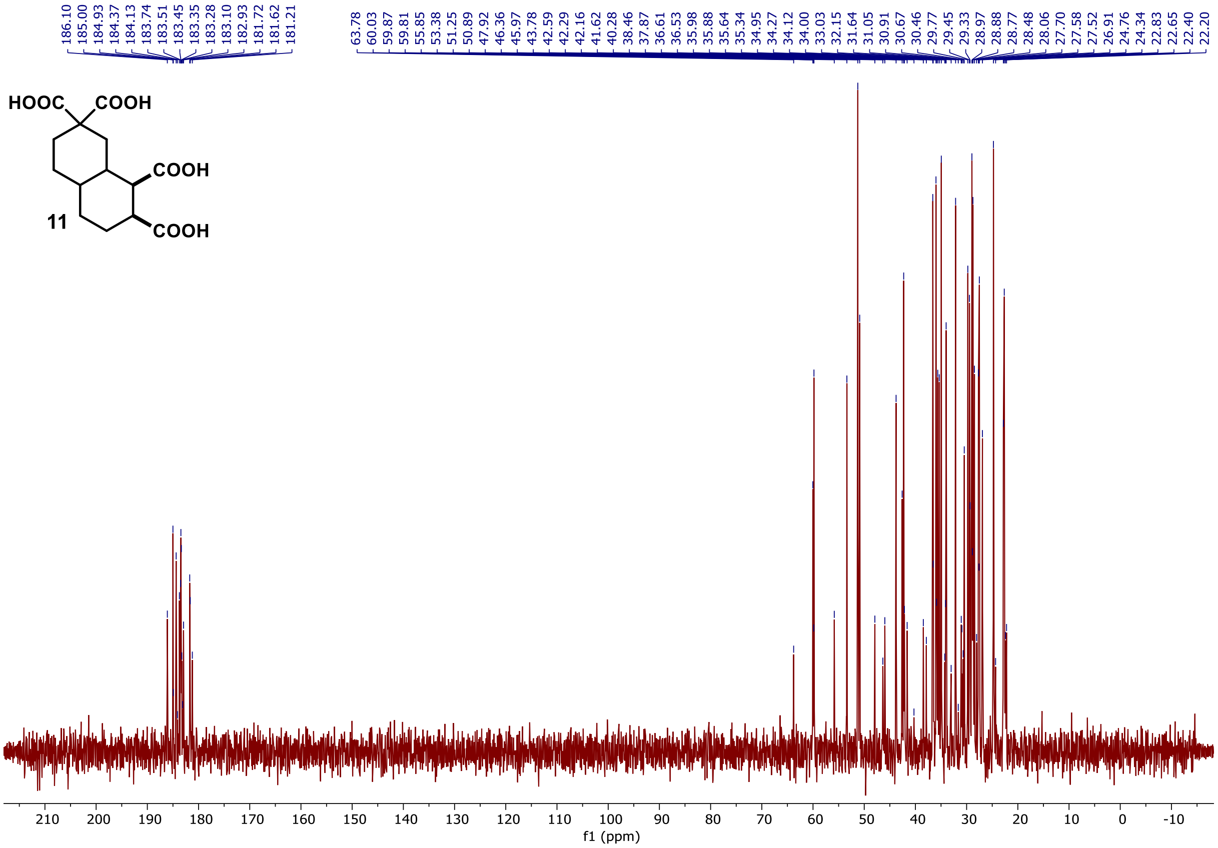

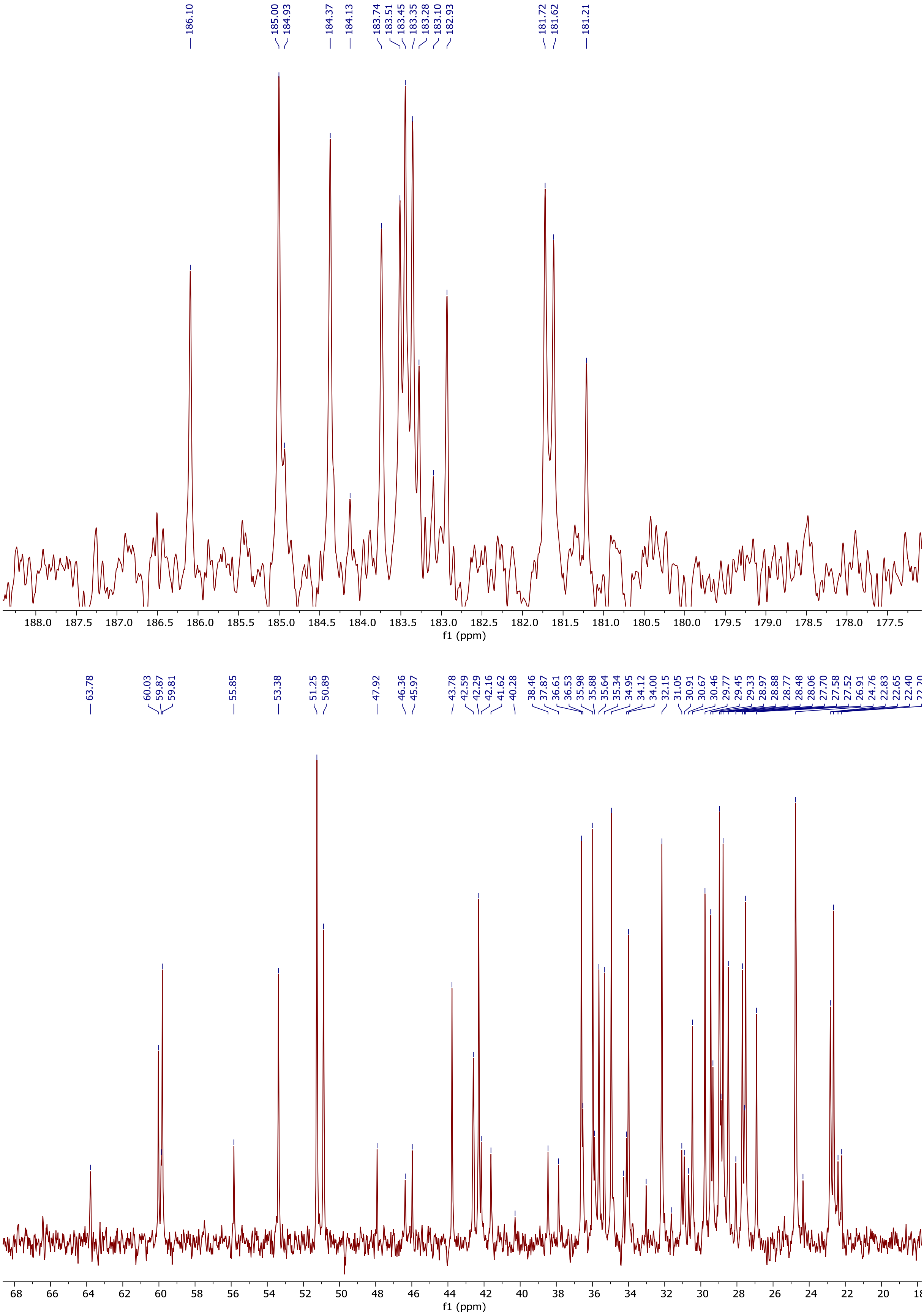

Figure S25: ^{13}C NMR spectra of compound **11** (151 MHz, 298K, 0.1M NaOD in D_2O)

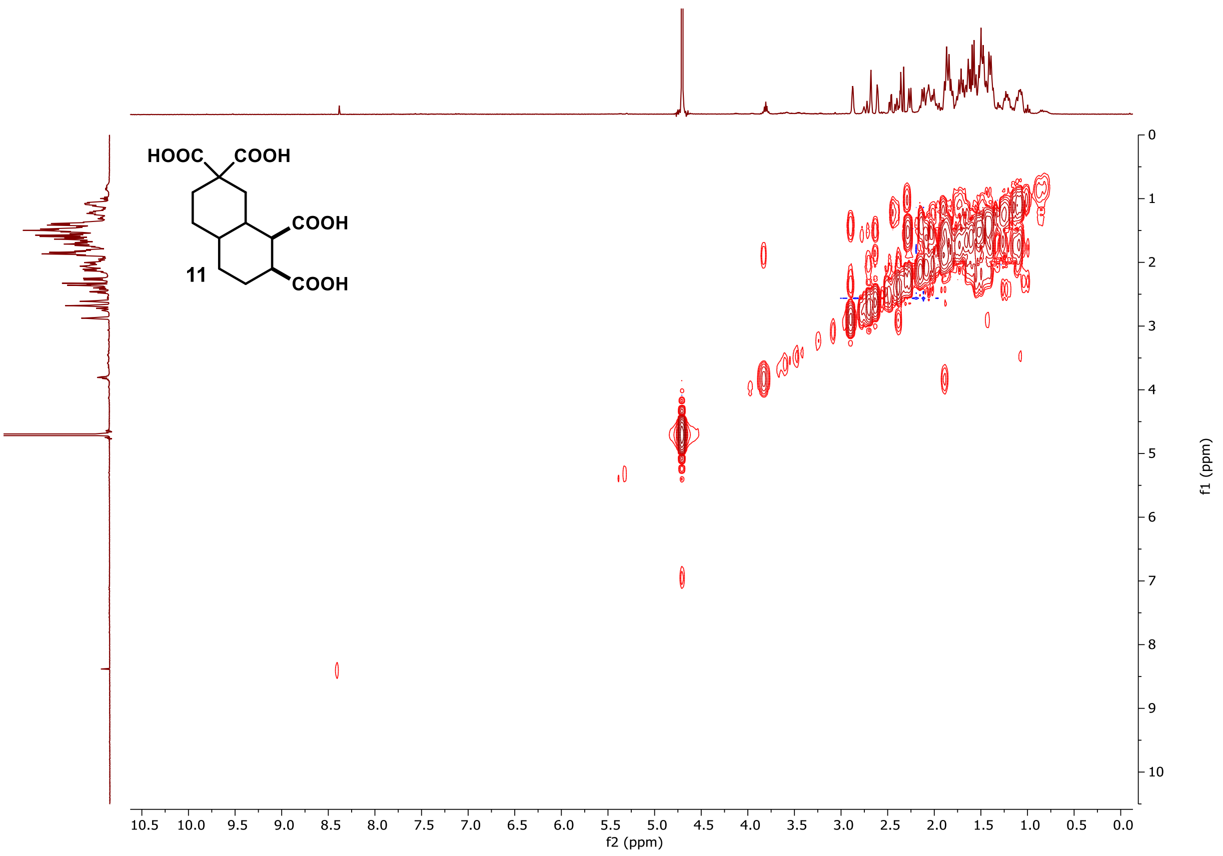

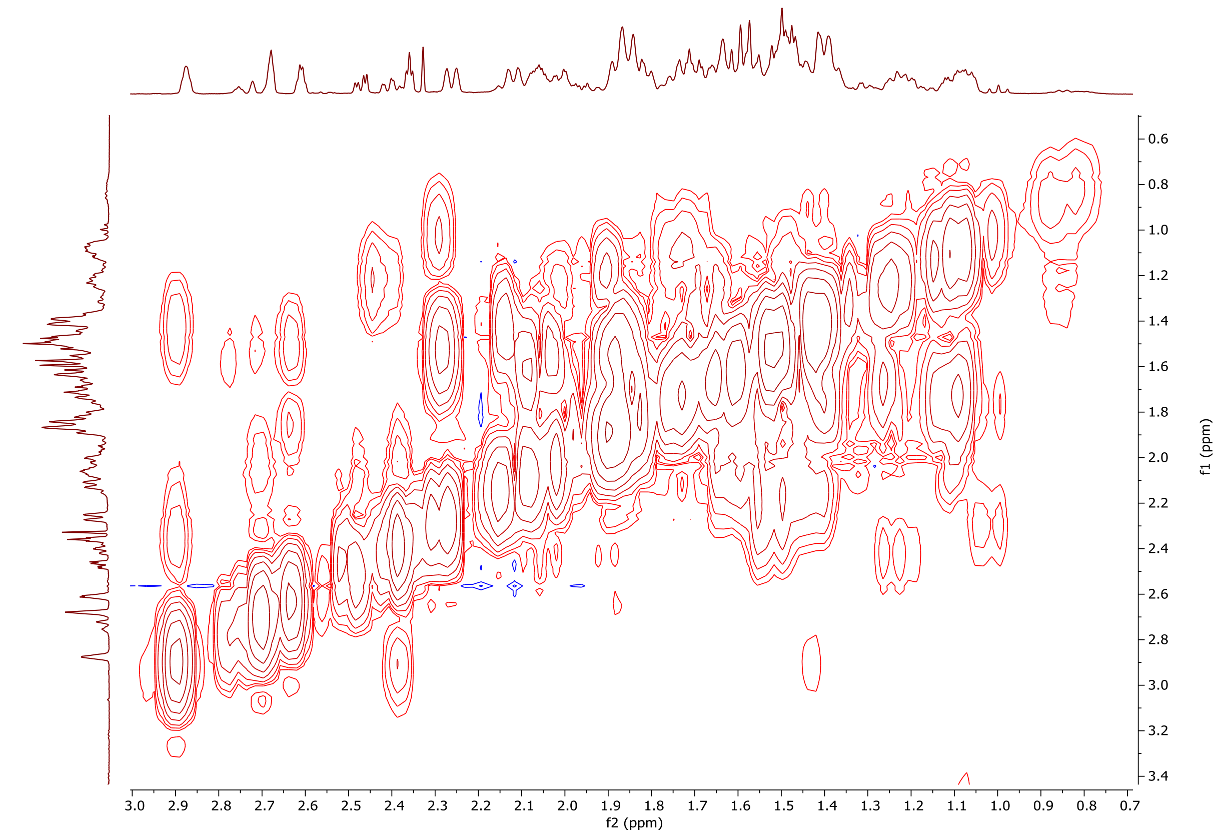

Figure S26: COSY spectra of compound **11** (600 MHz, 298K, 0.1M NaOD in D_2O)

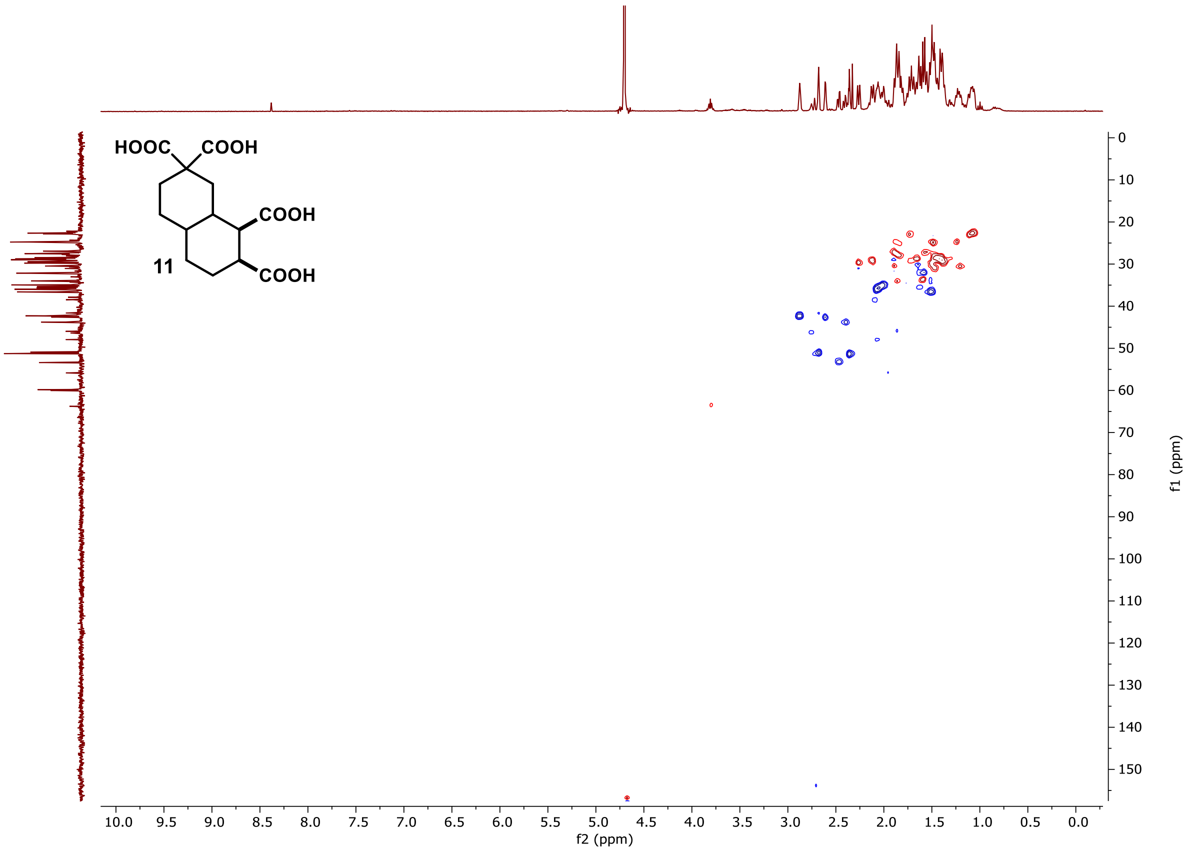

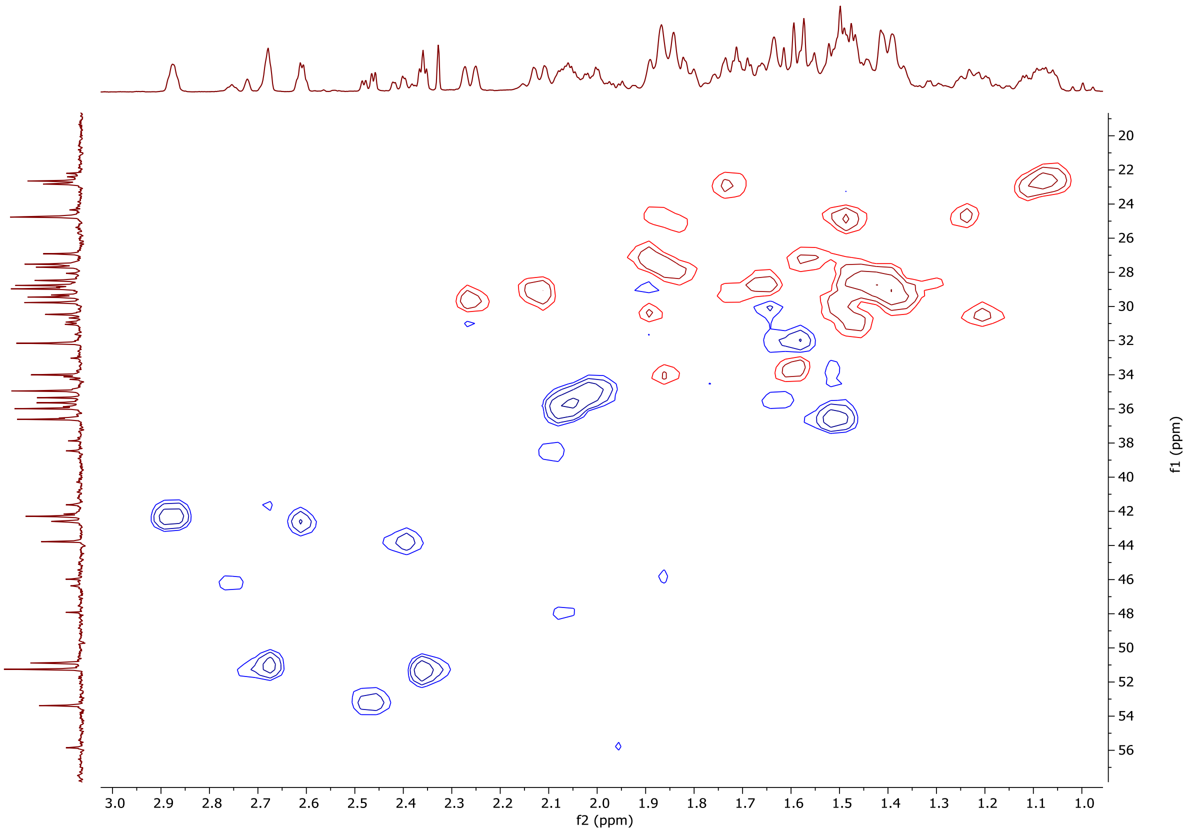

Figure S27: HSQC spectra of compound **11** (600 MHz, 298K, 0.1M NaOD in D_2O)

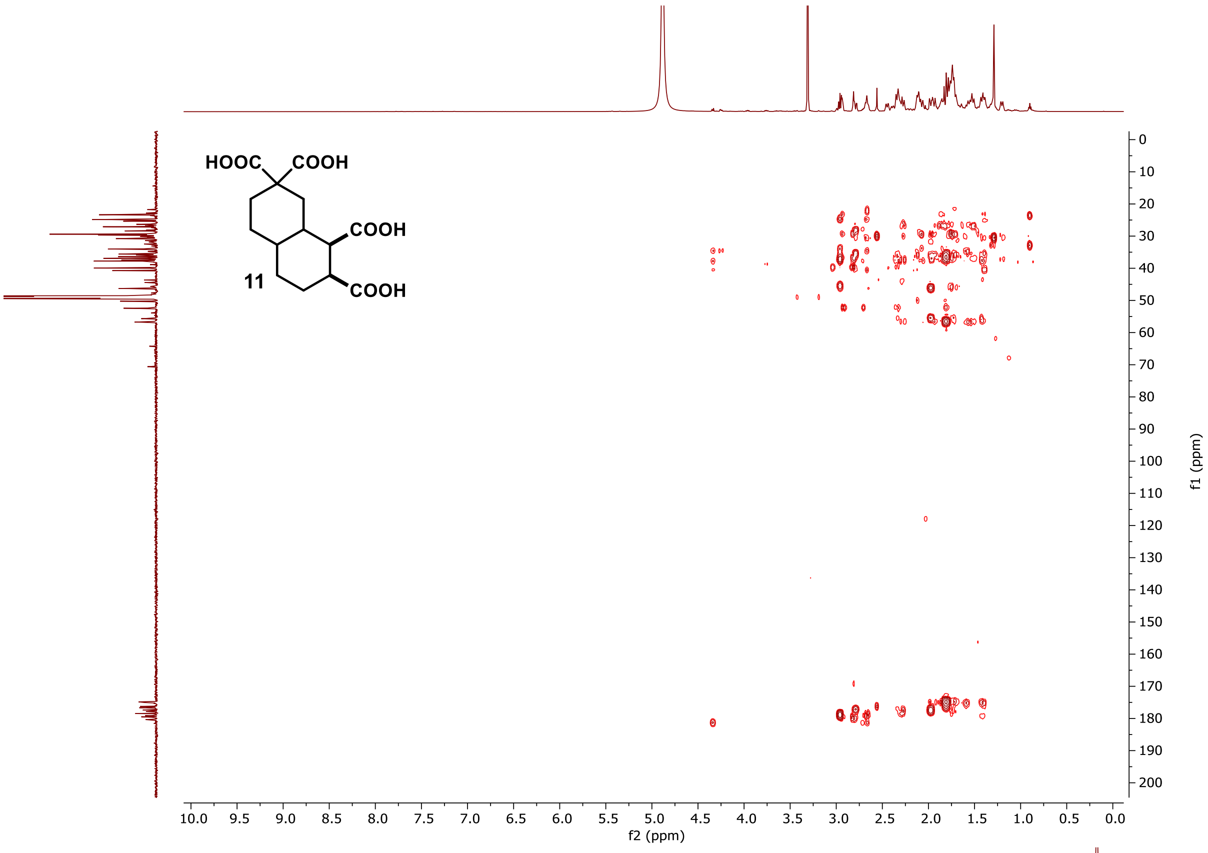
HOOC
COOH
COOH
COOH
11
f2 (ppm)
f1 (ppm)

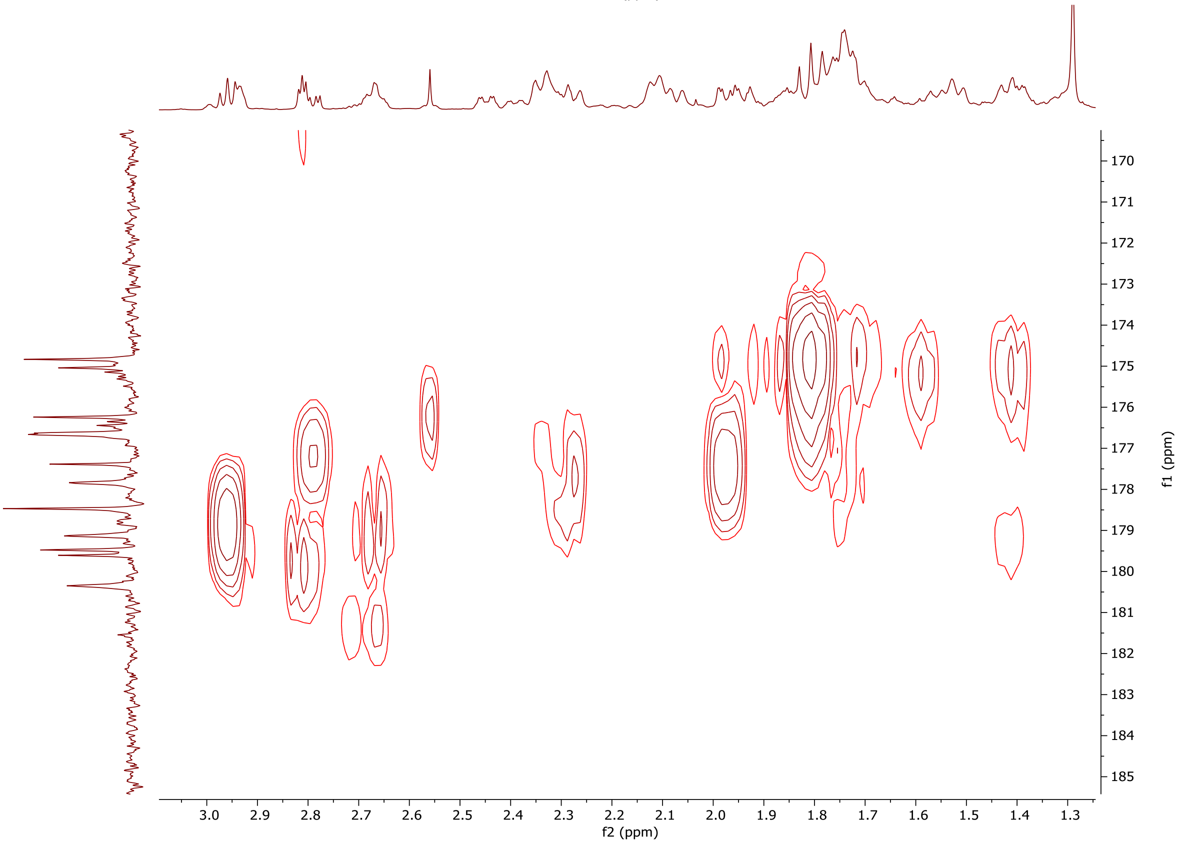
f2 (ppm)
f1 (ppm)

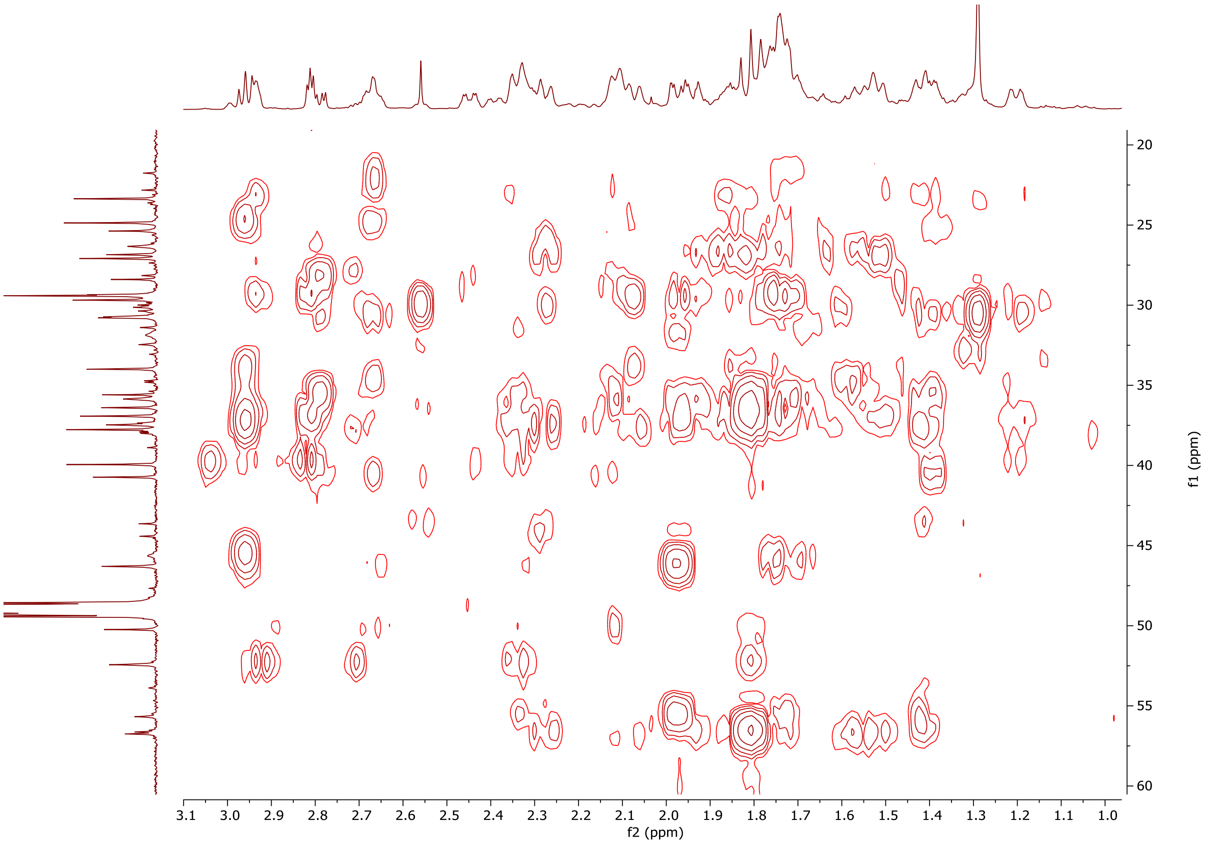

Figure S28: HMBC spectra of compound **11** (600 MHz, 298K, 0.1M NaOD in D_2O)

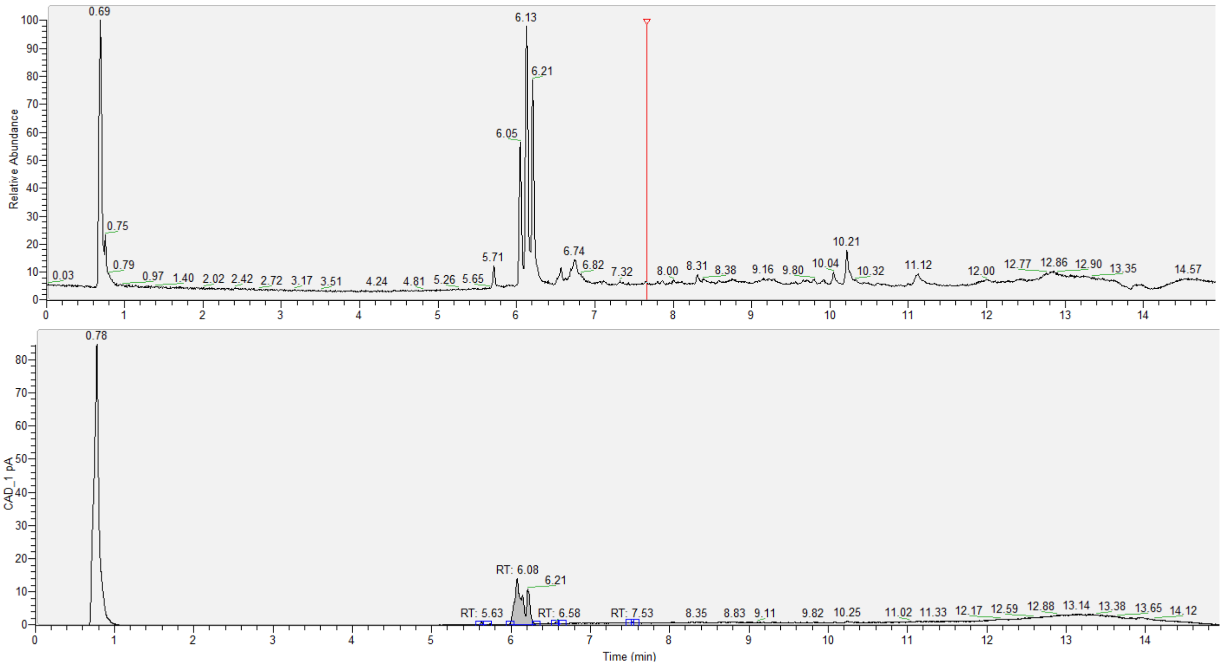

Figure S29: *TIC trace (top) and CID trace (bottom) of CRAM analogue **12**.*

Table S4: *LC-MS data and peak identities for CRAM analogue **12**.*

Apex RT	Start RT	End RT	Area	%Area	*m/z*	Identity
5.63	5.60	5.70	1.854	1.56	313	title compound **12** isomer
6.08	5.98	6.31	114.840	96.56	313	three overlapped title compound **12** isomers
6.58	6.56	6.64	1.676	1.41	339	unidentified impurity
7.53	7.50	7.56	0.563	0.47	327	unidentified impurity

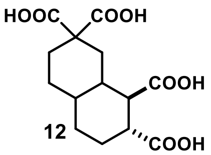

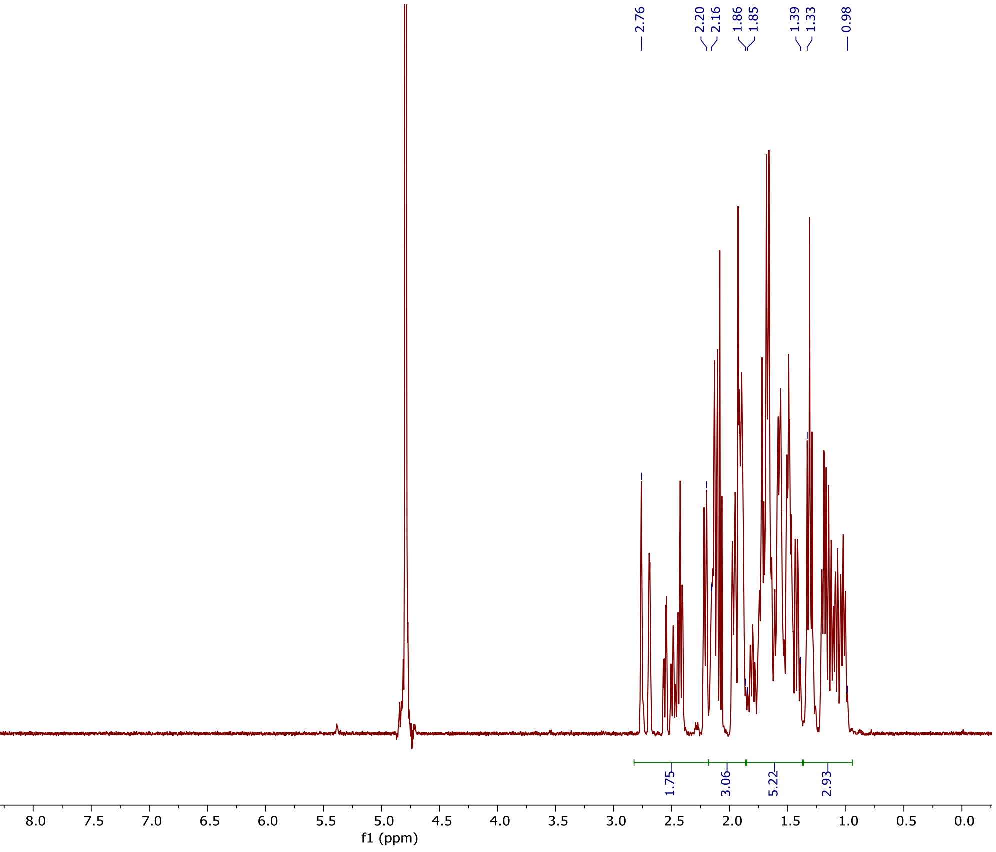

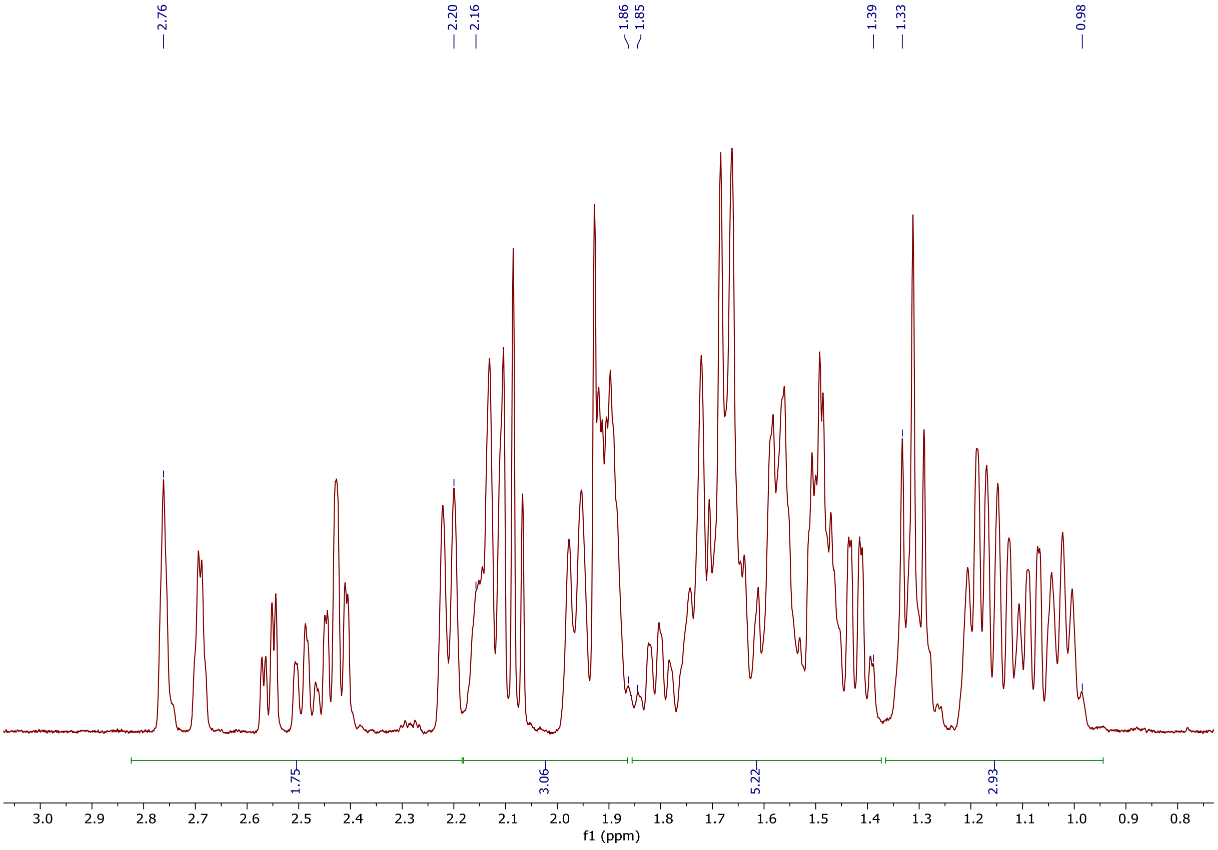

Figure S30: ^{1}H NMR spectra of compound **12** (600 MHz, 298K, 0.1M NaOD in D_2O)

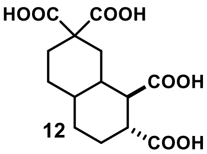
HOOC
COOH
COOH
COOH
12

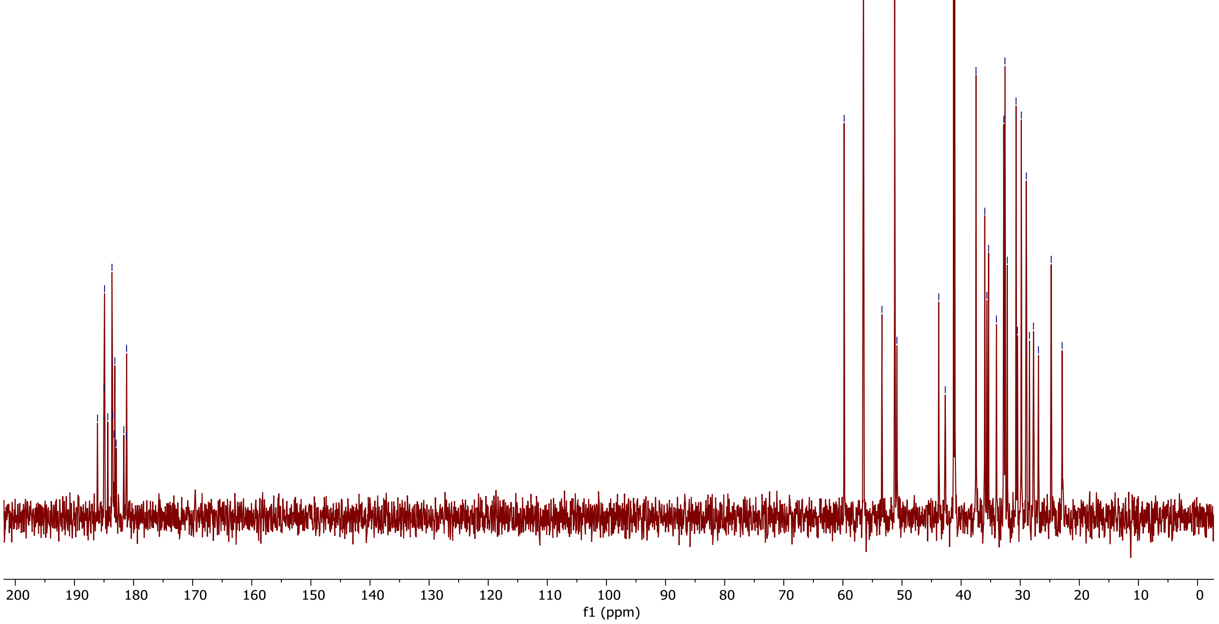
186.10
185.01
184.90
184.36
183.65
183.52
183.28
183.16
182.93
181.64
181.22
181.17
59.78
56.52
53.39
51.23
50.86
43.78
41.27
41.07
37.47
35.99
35.64
35.35
34.02
32.78
32.58
32.18
30.70
30.46
29.81
28.97
28.43
27.75
f1 (ppm)

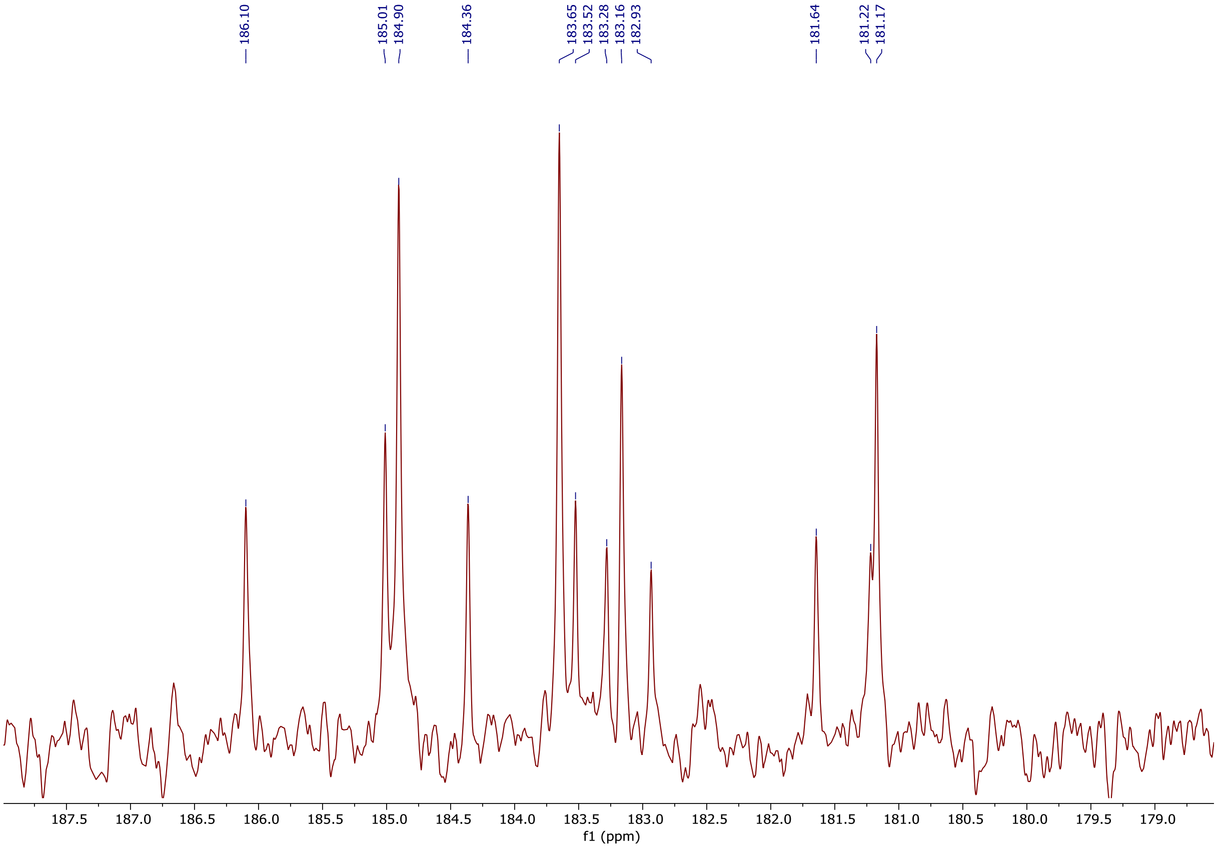
186.10
185.01
184.90
184.36
183.65
183.52
183.28
183.16
182.93
181.64
181.22
181.17
f1 (ppm)

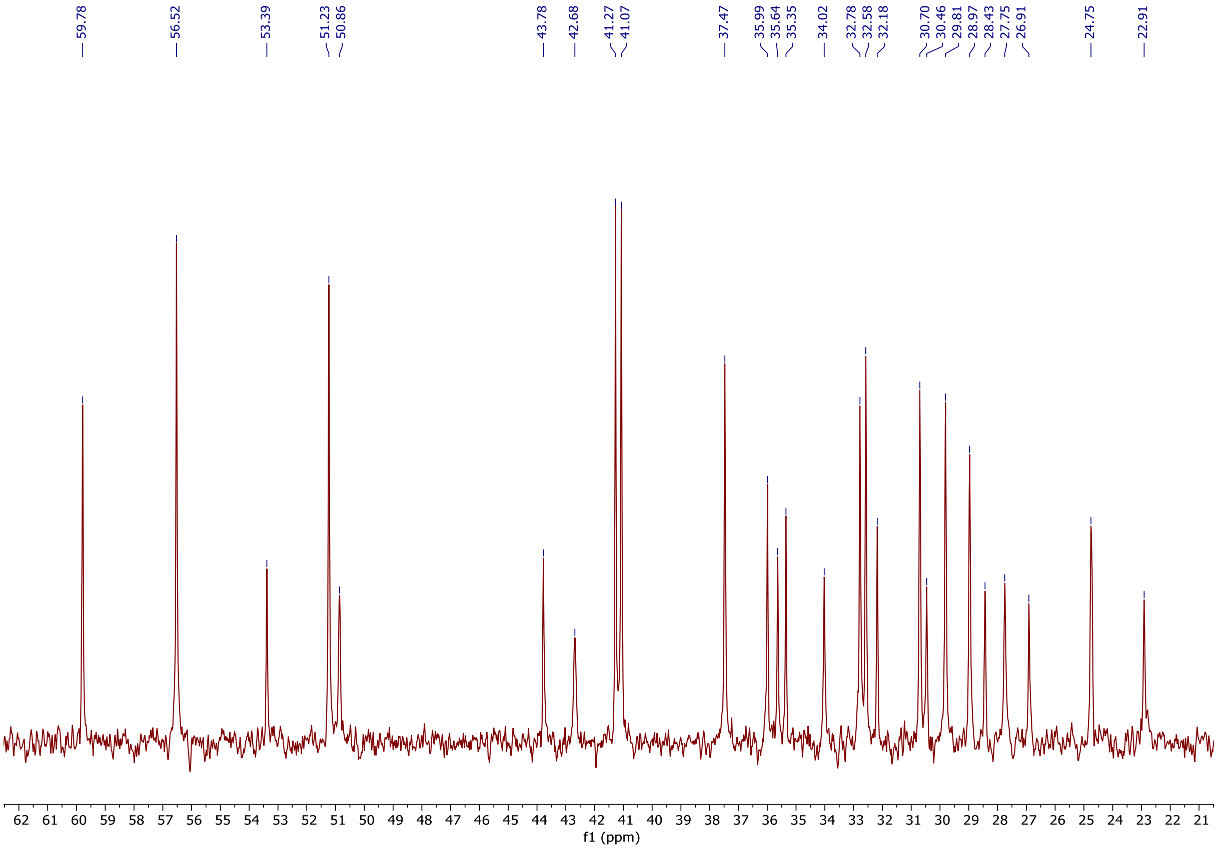

Figure S31: ^{13}C NMR spectra of compound **12** (151 MHz, 298K, 0.1M NaOD in D_2O)

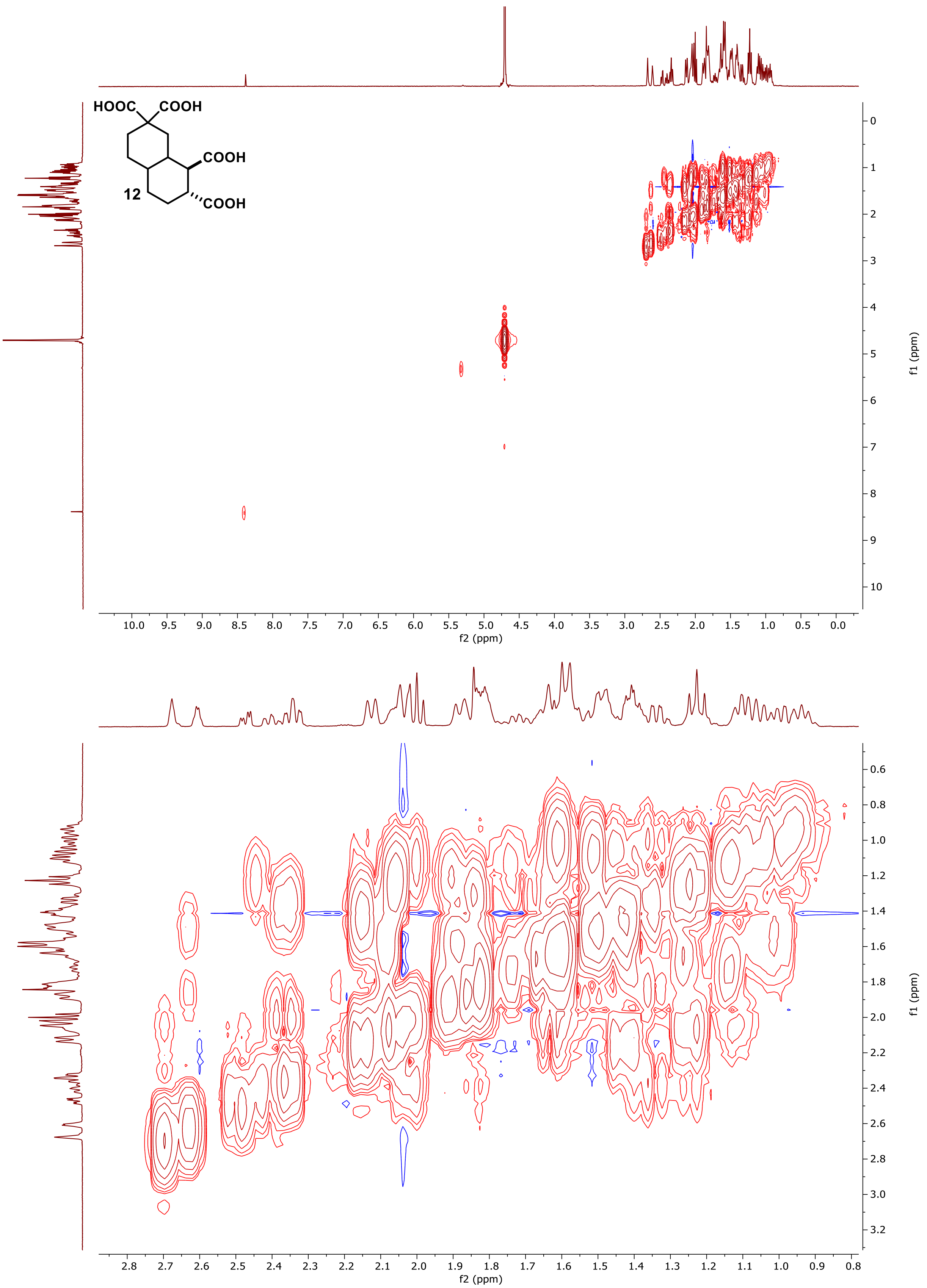

Figure S32: COSY spectra of compound **12** (600 MHz, 298K, 0.1M NaOD in D_2O)

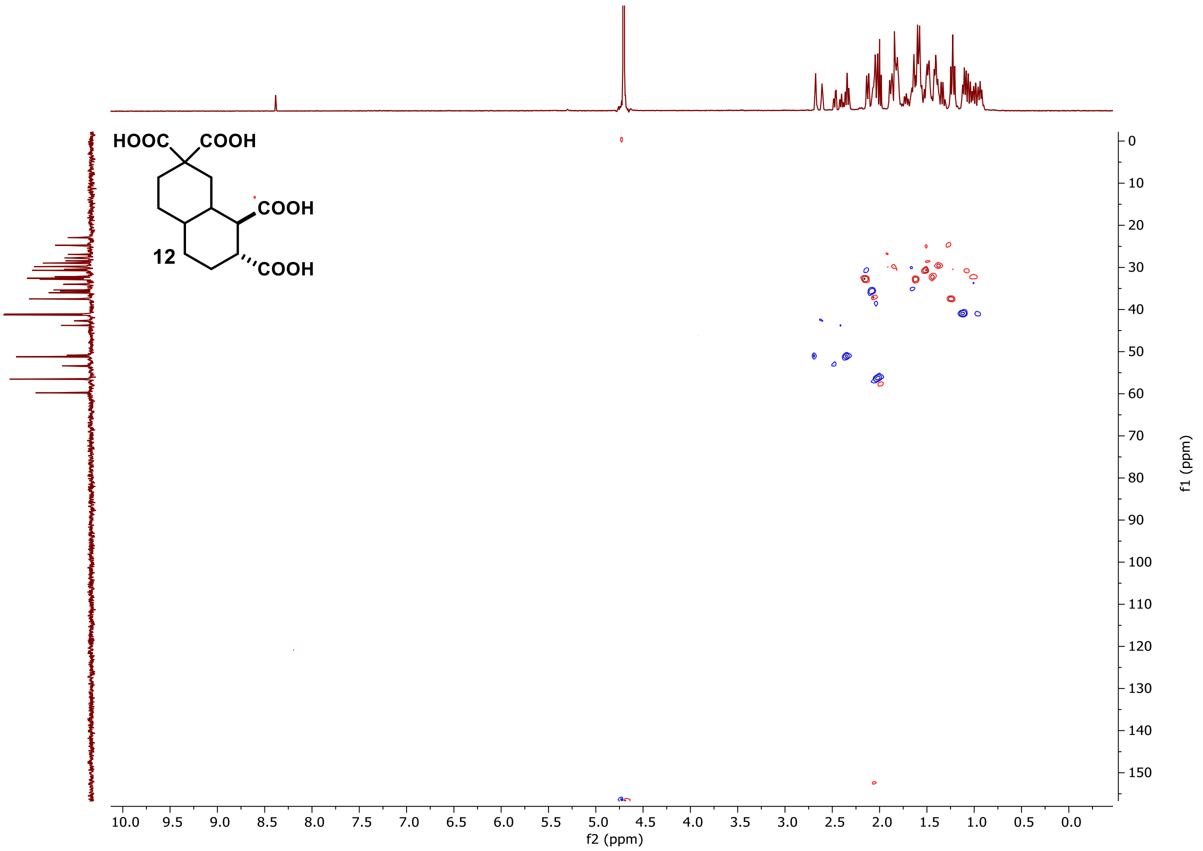

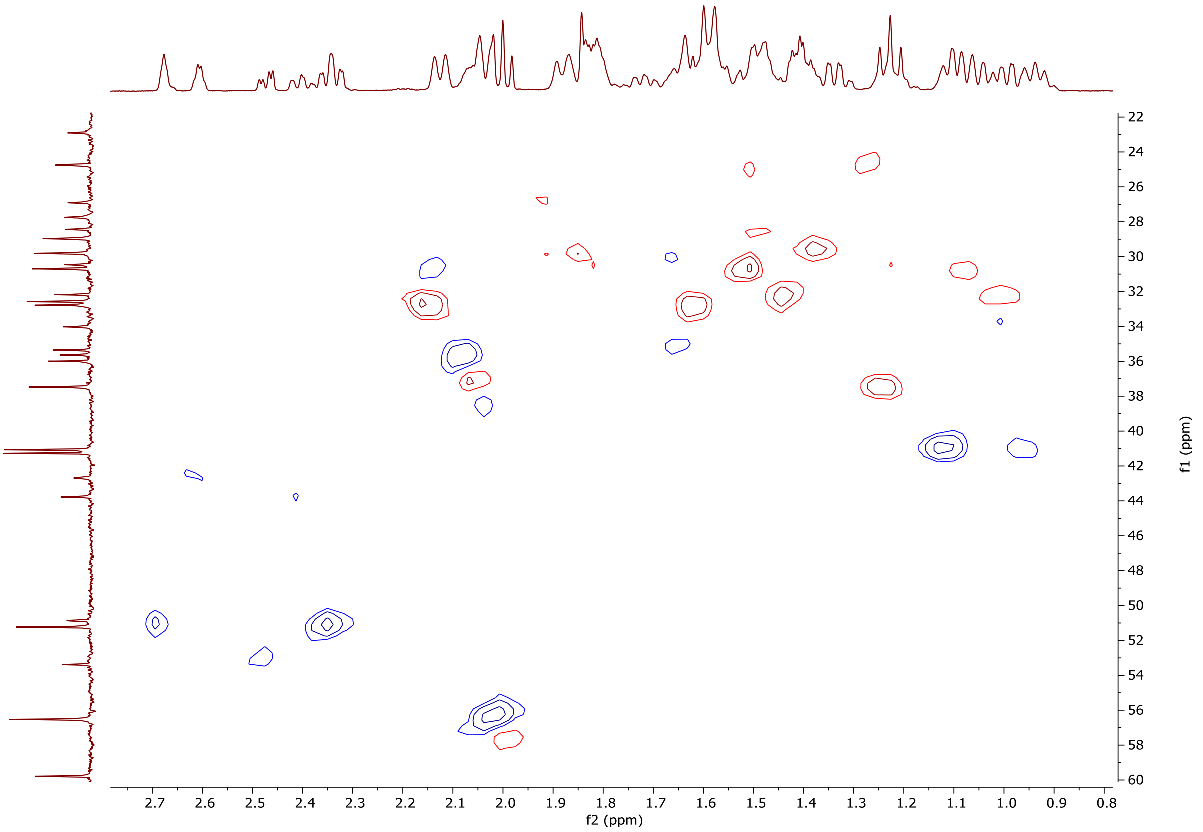

Figure S33: HSQC spectra of compound **12** (600 MHz, 298K, 0.1M NaOD in D_2O)

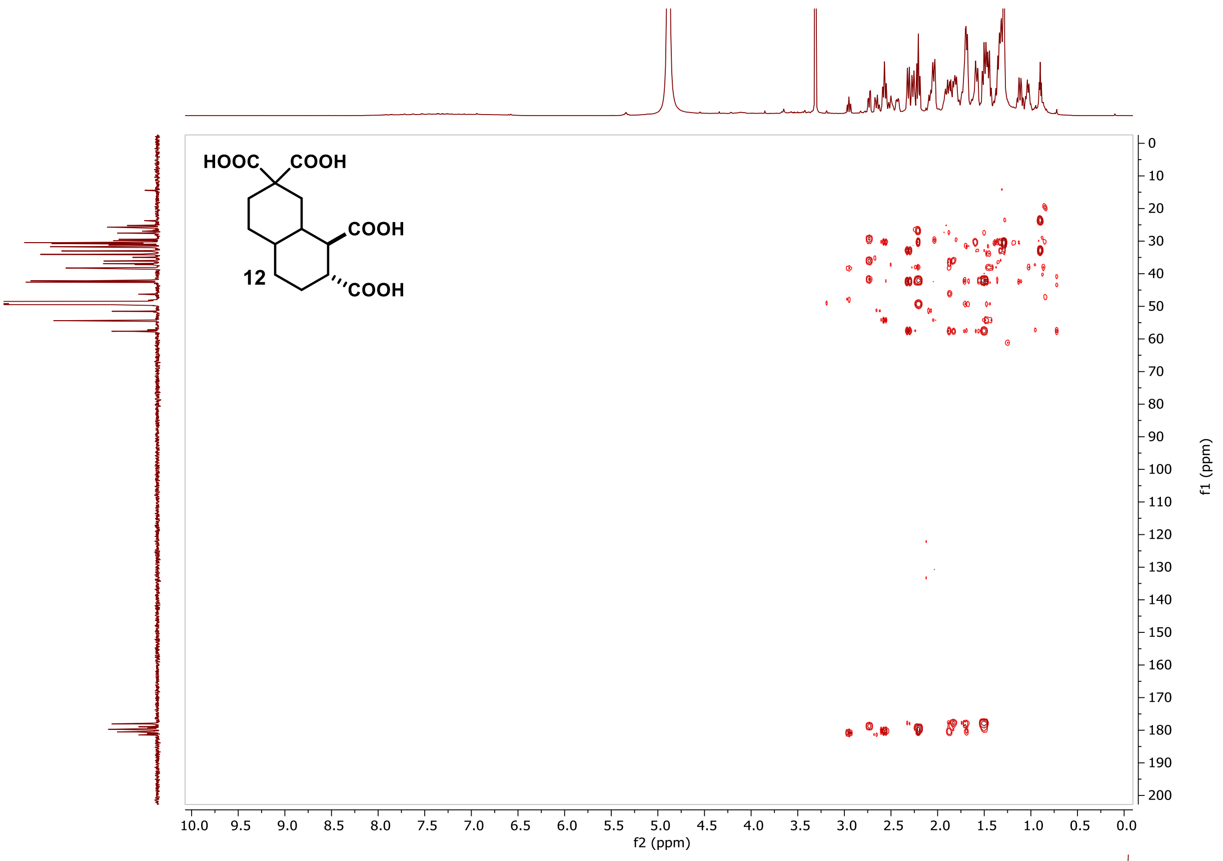
HOOC
COOH
COOH
12
COOH
f2 (ppm)
f1 (ppm)

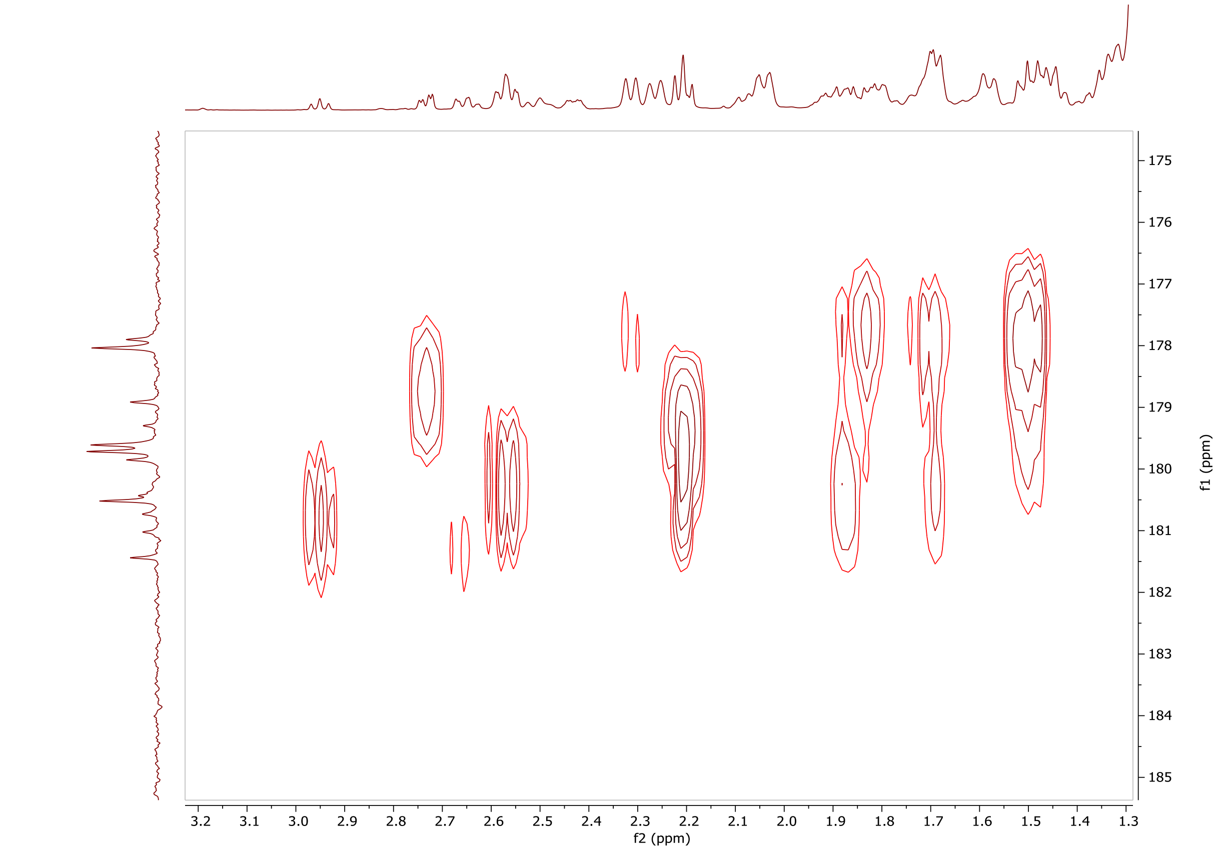
f2 (ppm)
f1 (ppm)

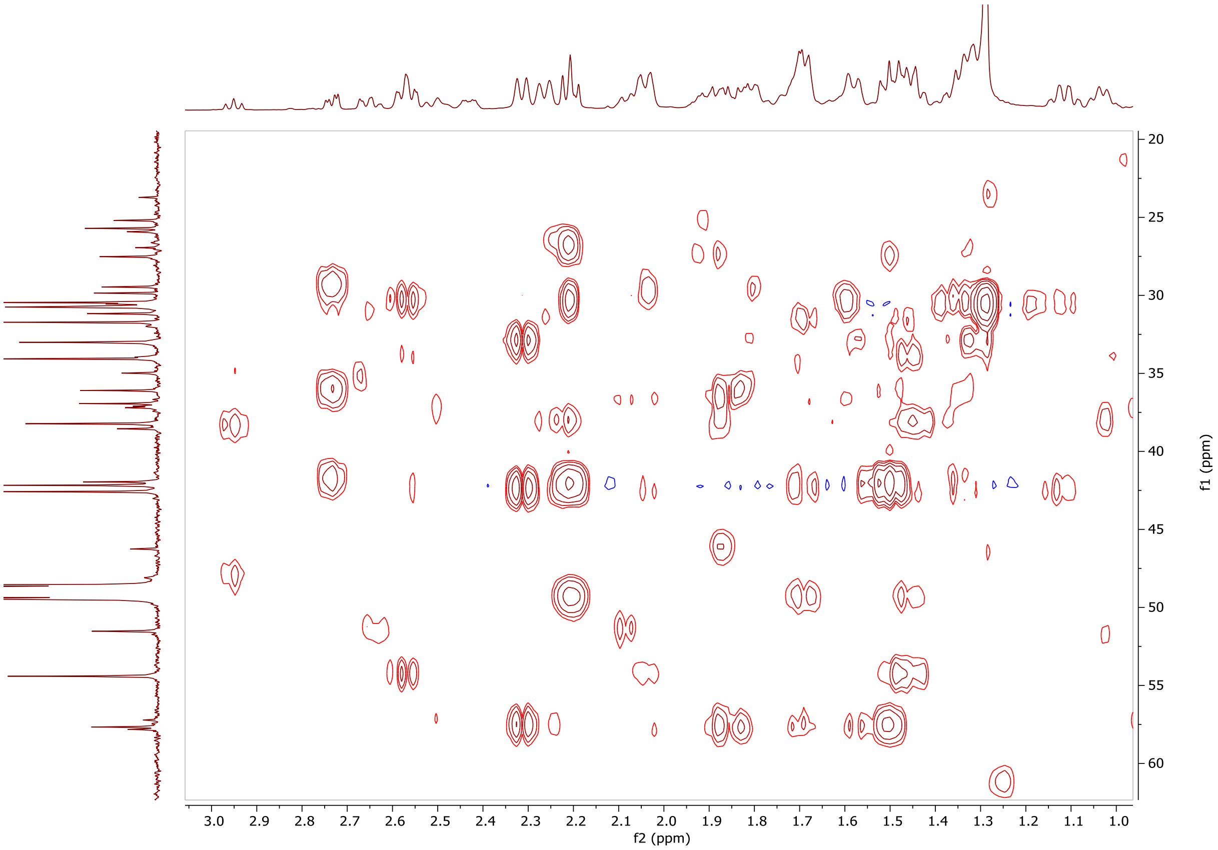

Figure S34: HMBC spectra of compound **12** (600 MHz, 298K, 0.1M NaOD in D_2O)

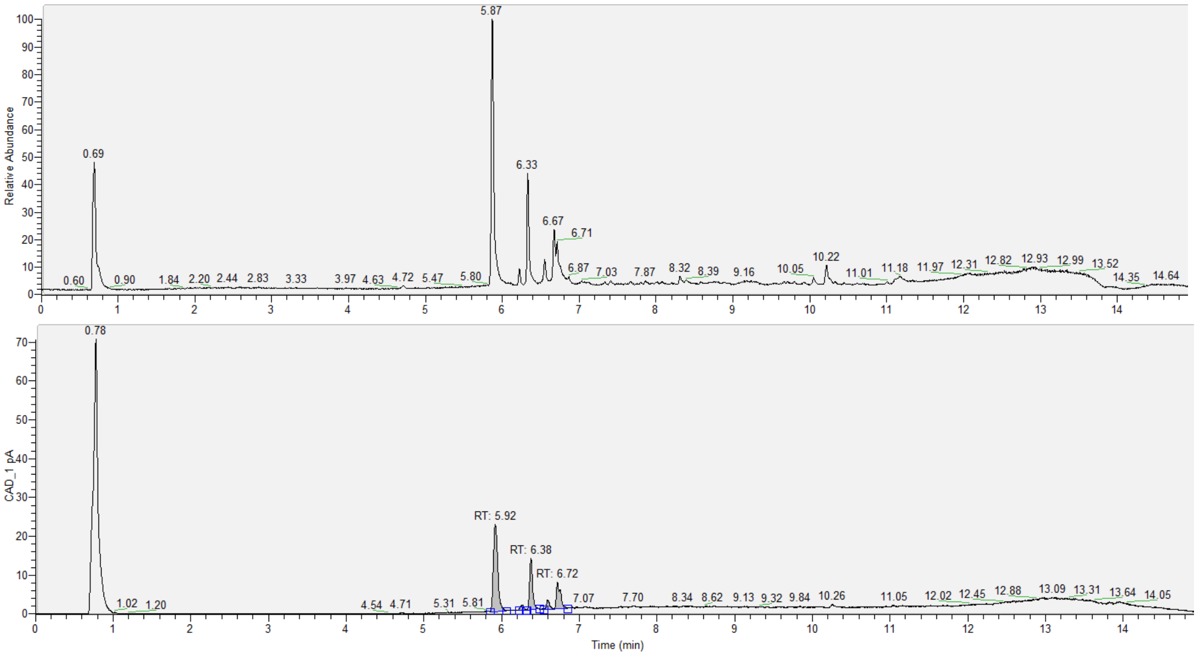

Figure S35: *TIC trace (top) and CID trace (bottom) of CRAM analogue **13**.*

Table S5: *LC-MS data and peak identities for CRAM analogue **13**.*

Apex RT	Start RT	End RT	Area	%Area	*m/z*	Identity
5.92	5.85	6.06	88.657	55.87	267	title compound **13** isomer
6.26	6.23	6.31	4.369	2.75	267	title compound **13** isomer
6.38	6.33	6.49	35.049	22.09	267	title compound **13** isomer
6.59	6.54	6.66	6.216	3.92	267	title compound **13** isomer
6.72	6.68	6.84	24.397	15.37	267	title compound **13** isomer

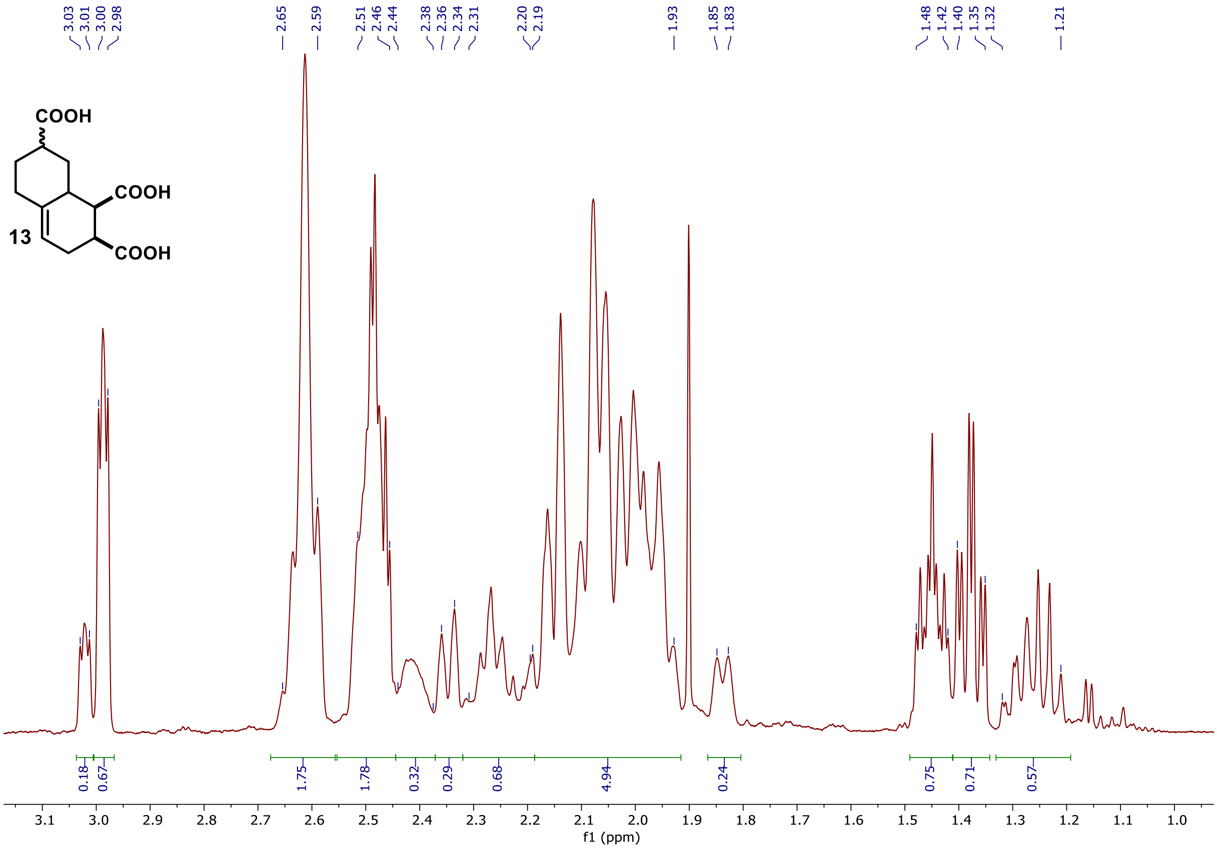

Figure S36: ^{1}H NMR spectra of compound **13** (600 MHz, 298K, 0.1M NaOD in D_2O)

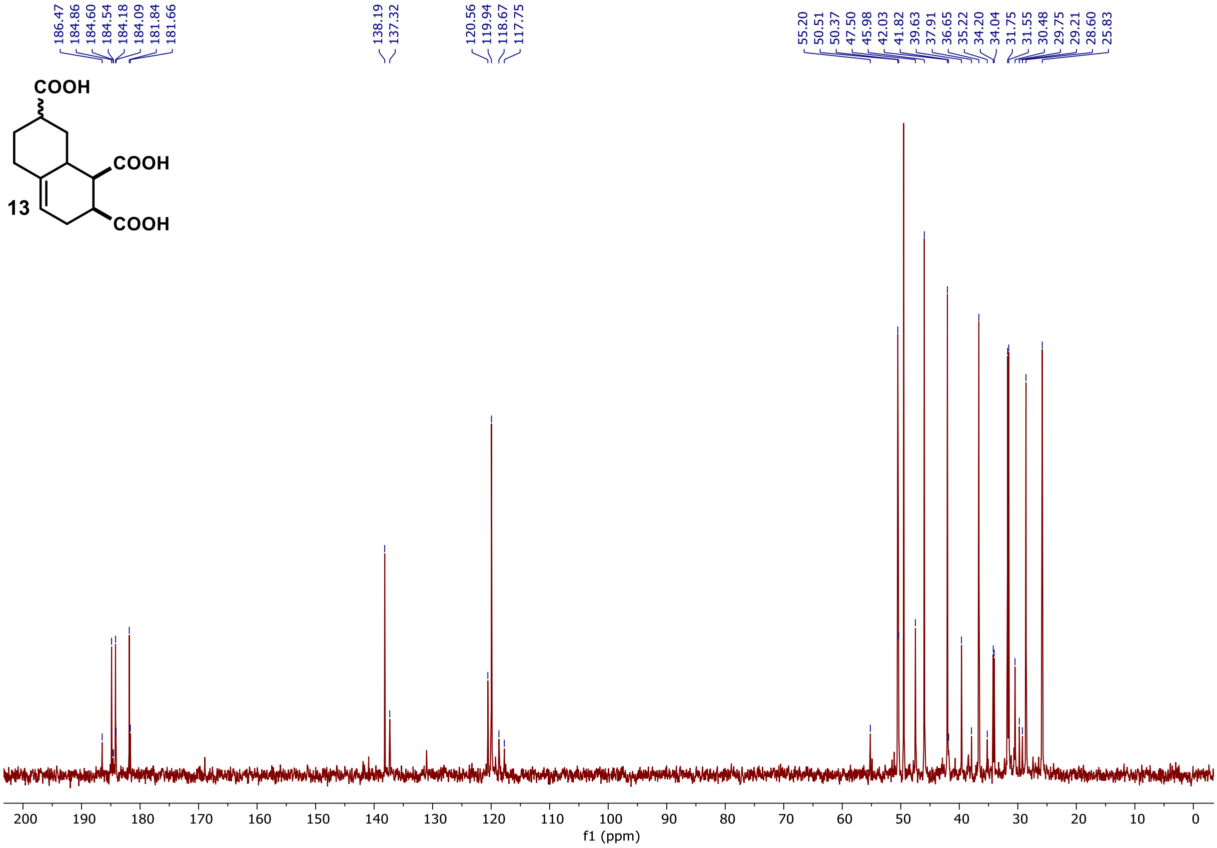

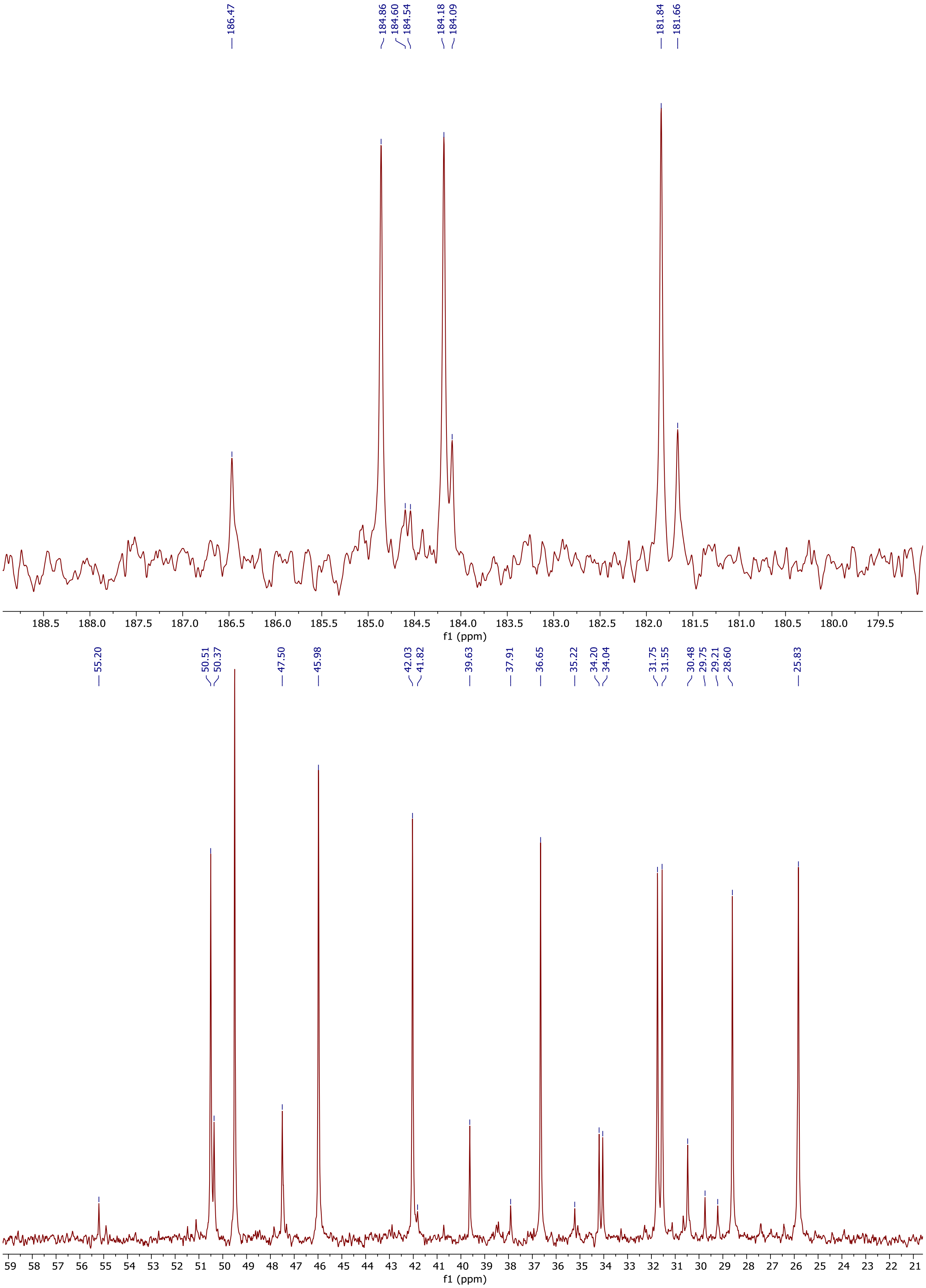

Figure S37: ^{13}C NMR spectra of compound **13** (151 MHz, 298K, 0.1M NaOD in D_2O)

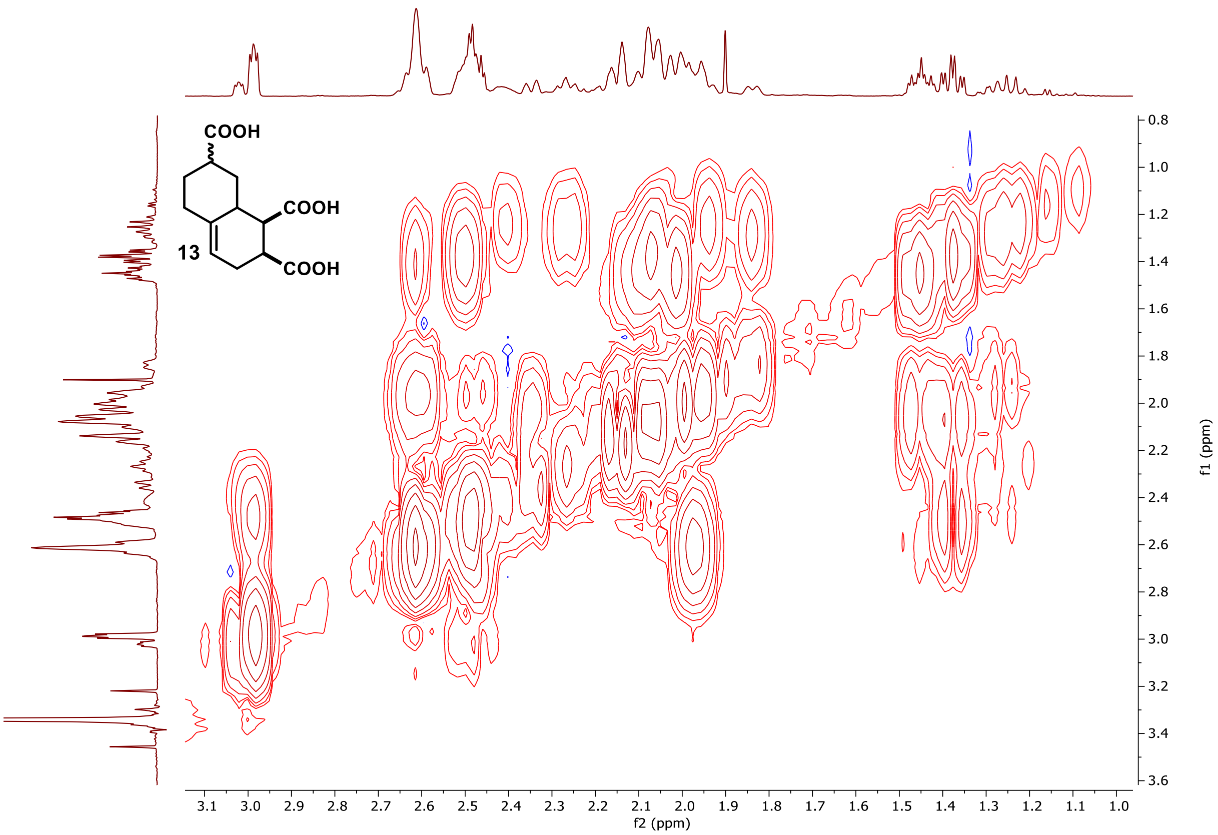

Figure S38: COSY spectra of compound **13** (600 MHz, 298K, 0.1M NaOD in D_2O)

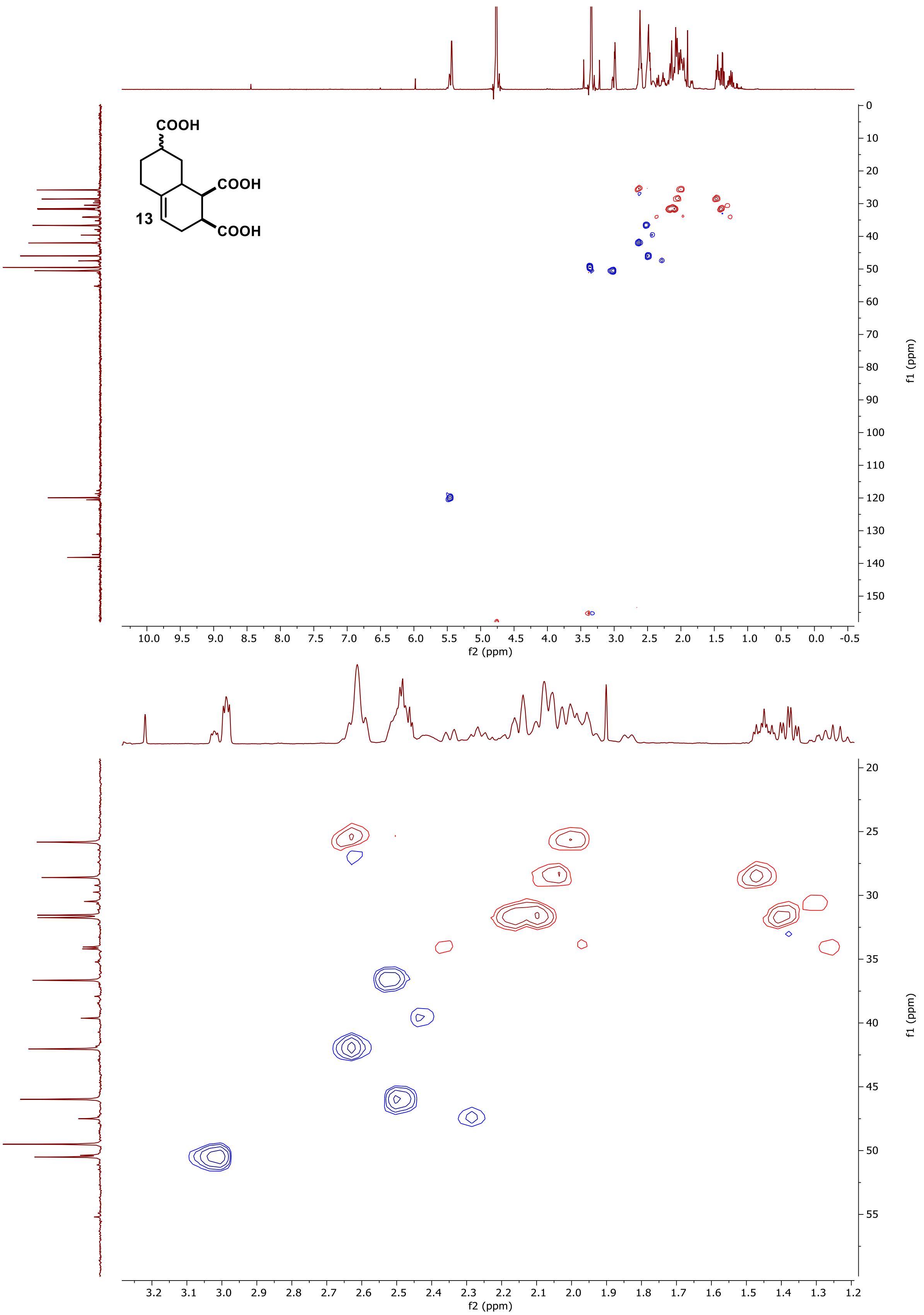

Figure S39: HSQC spectra of compound **13** (600 MHz, 298K, 0.1M NaOD in D_2O)

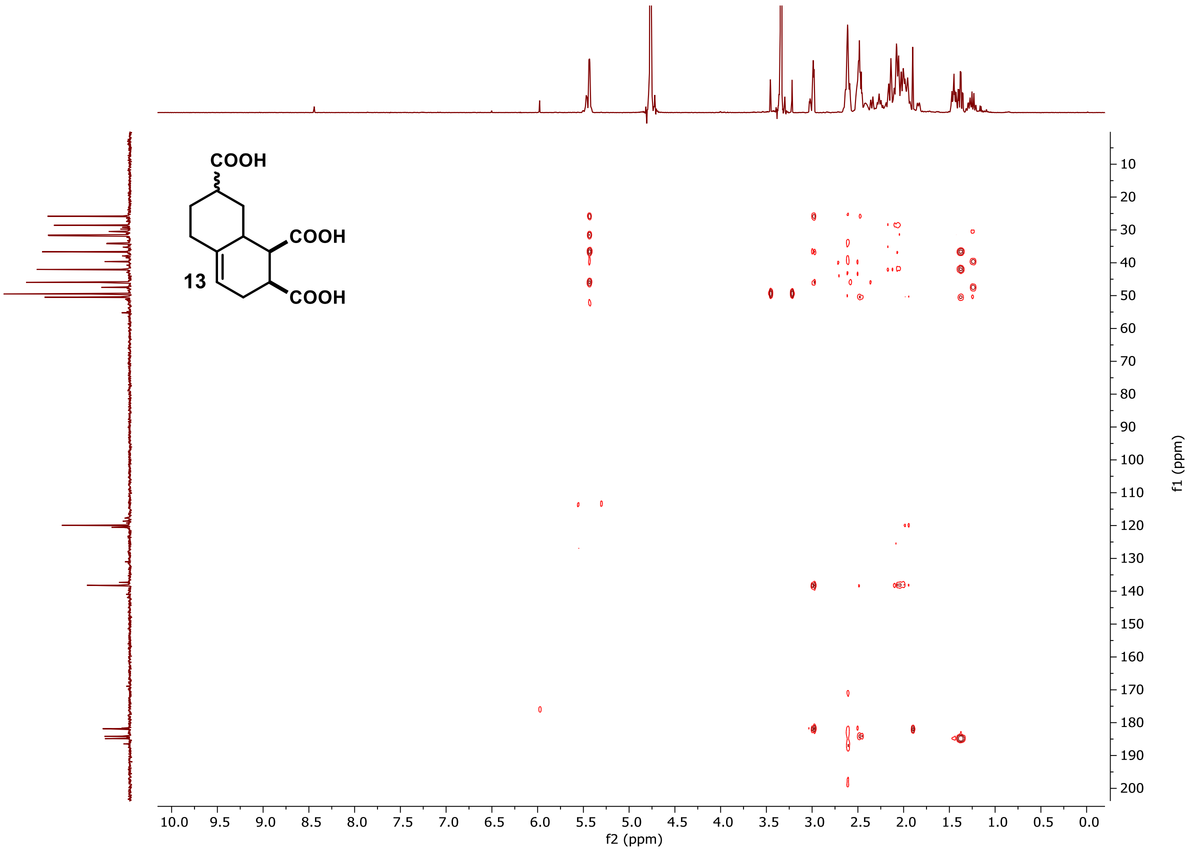
COOH
COOH
COOH
13
f2 (ppm)
f1 (ppm)

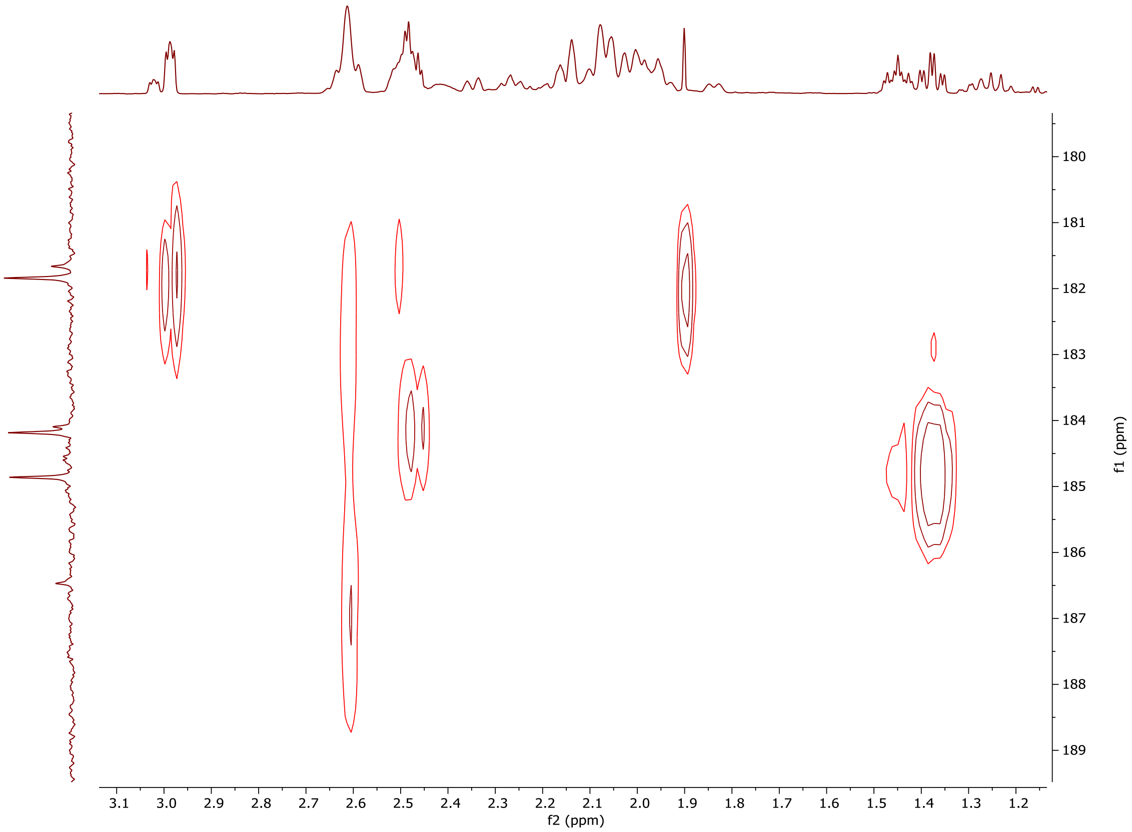
f2 (ppm)
f1 (ppm)

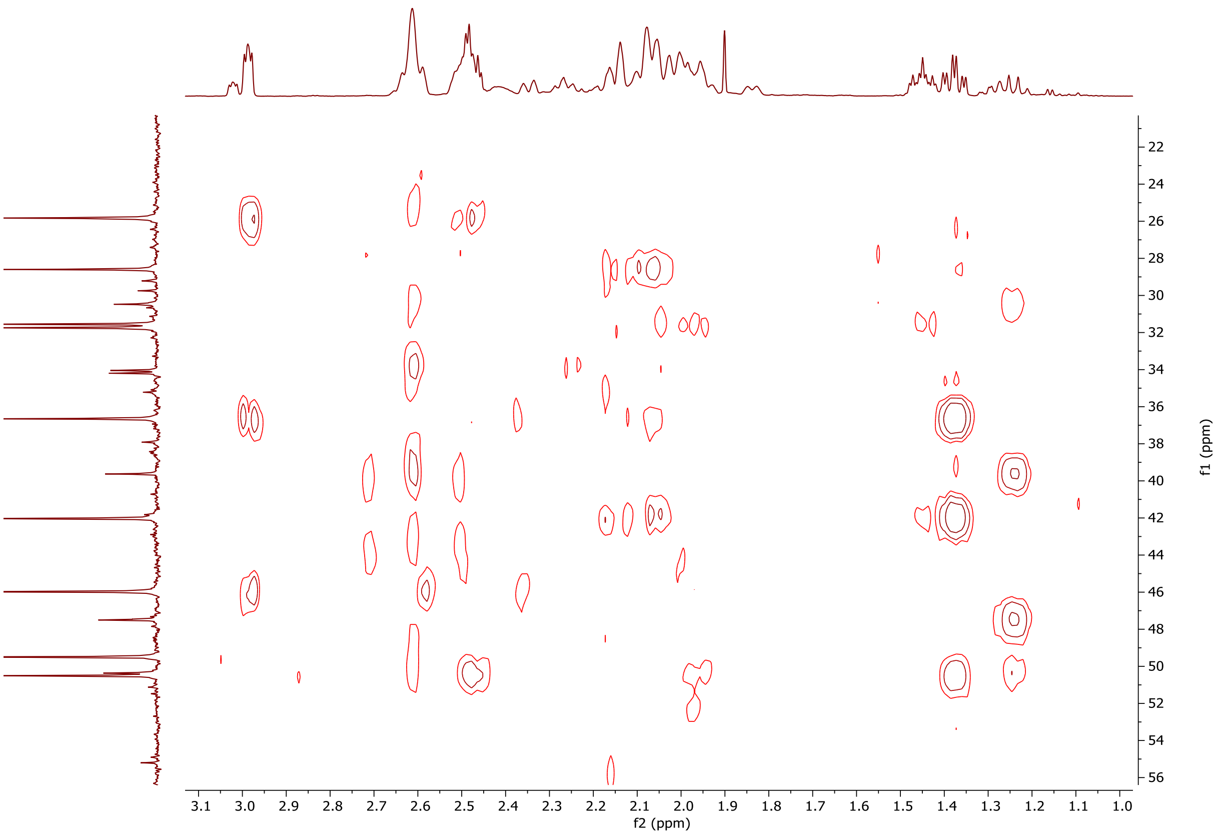

Figure S40: HMBC spectra of compound **13** (600 MHz, 298K, 0.1M NaOD in D_2O)

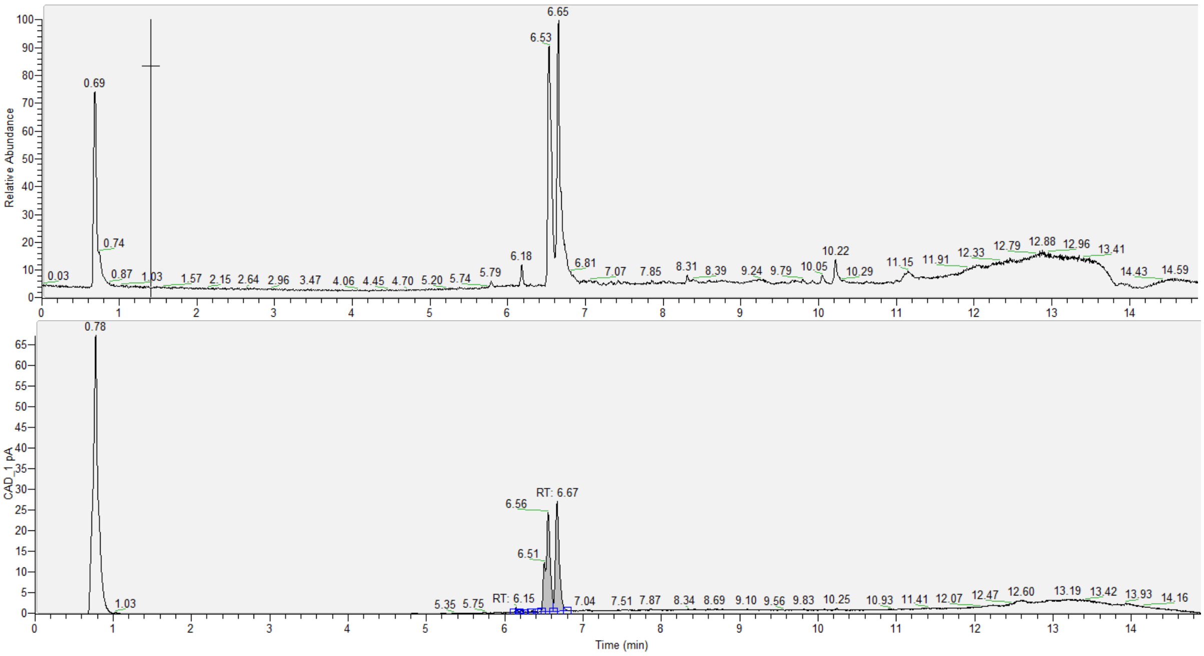

Figure S41: *TIC trace (top) and CID trace (bottom) of CRAM analogue **14**.*

Table S6: *LC-MS data and peak identities for CRAM analogue **14**.*

Apex RT	Start RT	End RT	Area	%Area	*m/z*	Identity
5.75	5.71	5.79	1.175	0.60	267	decarboxylated title compound **14** impurity
6.15	6.12	6.19	2.514	1.29	379	decarboxylated title compound **14** impurity
6.21	6.19	6.25	1.286	0.66	311	decarboxylated title compound **14** impurity
6.35	6.33	6.41	0.946	0.48	-	unidentified impurity
6.56	6.46	6.62	99.706	50.99	311	title compound **14** isomer
6.67	6.62	6.79	89.917	45.98		title compound **14** isomer

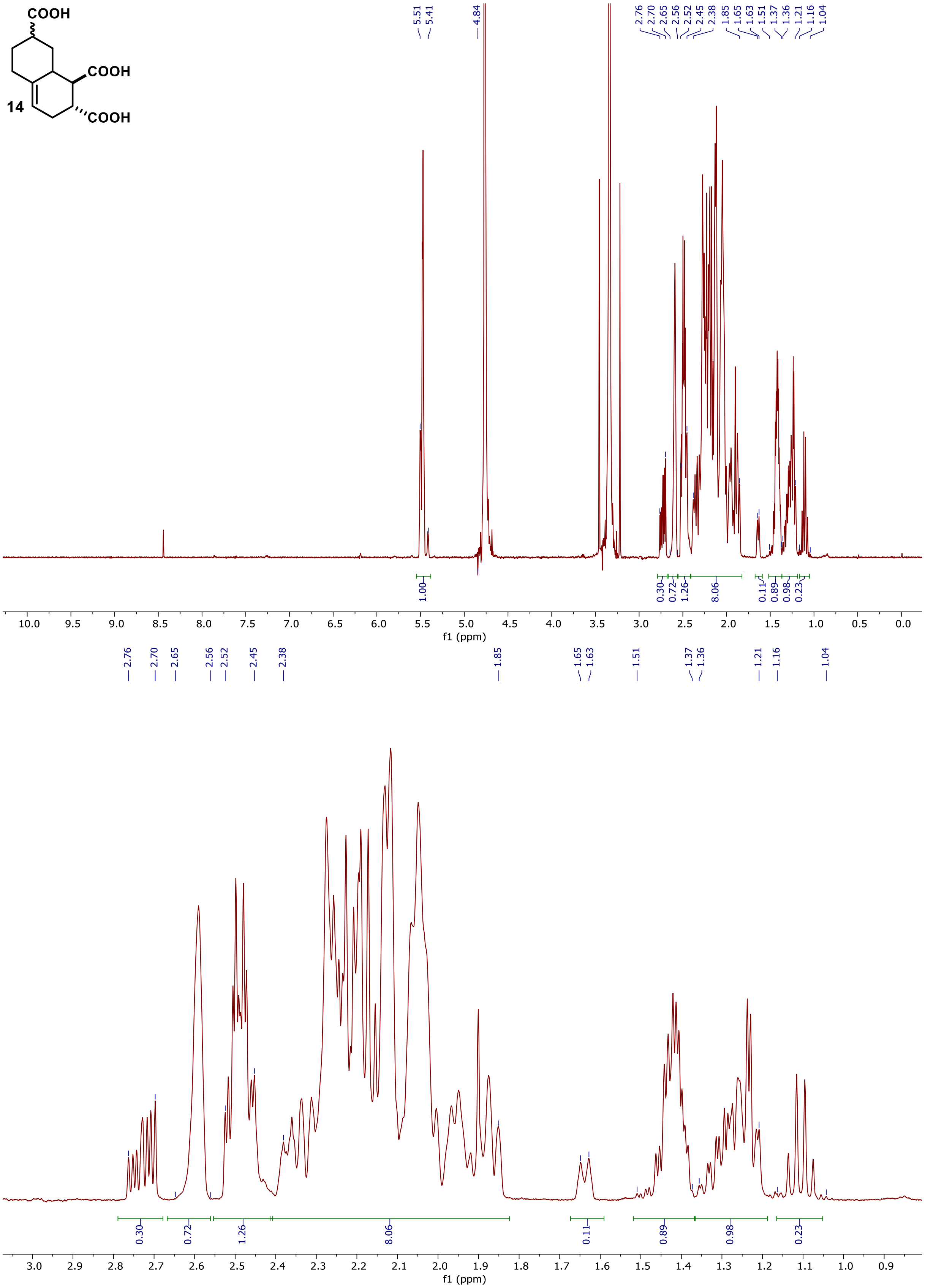

Figure S42: ^{1}H NMR spectra of compound **14** (600 MHz, 298K, 0.1M NaOD in D_2O)

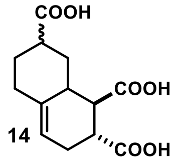
COOH
COOH
COOH

14

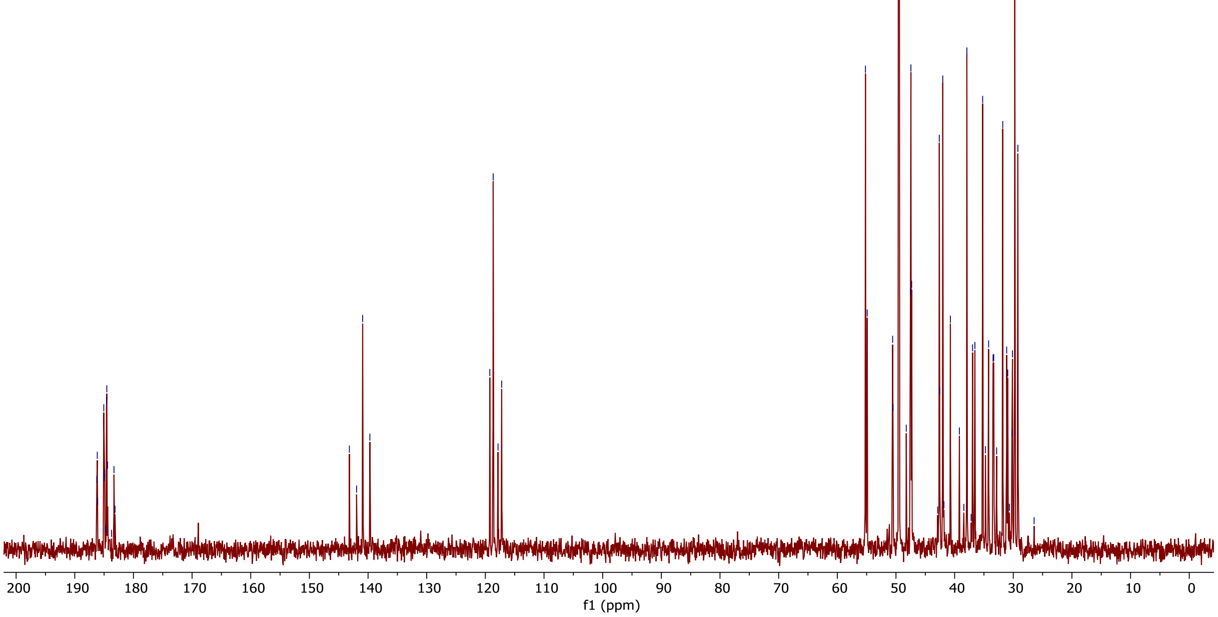
186.24
186.16
186.10
185.05
184.95
184.91
184.71
184.59
184.54
184.41
183.74
183.32
183.14
143.18
141.96
140.92
139.69
119.24
118.67
117.85
117.22
55.20
54.90
50.57
50.49
48.25
47.53
47.45
47.32
42.89
42.62
42.55
42.02
41.82
40.72
39.19
38.43
37.91
37.19
36.94
36.54
35.22
34.74
34.21
33.44
33.33
32.83
31.80
31.12
30.94
30.67
30.24
30.14
29.75
29.21
26.45
200 190 180 170 160 150 140 130 120 110 100 90 80 70 60 50 40 30 20 10 0
f1 (ppm)

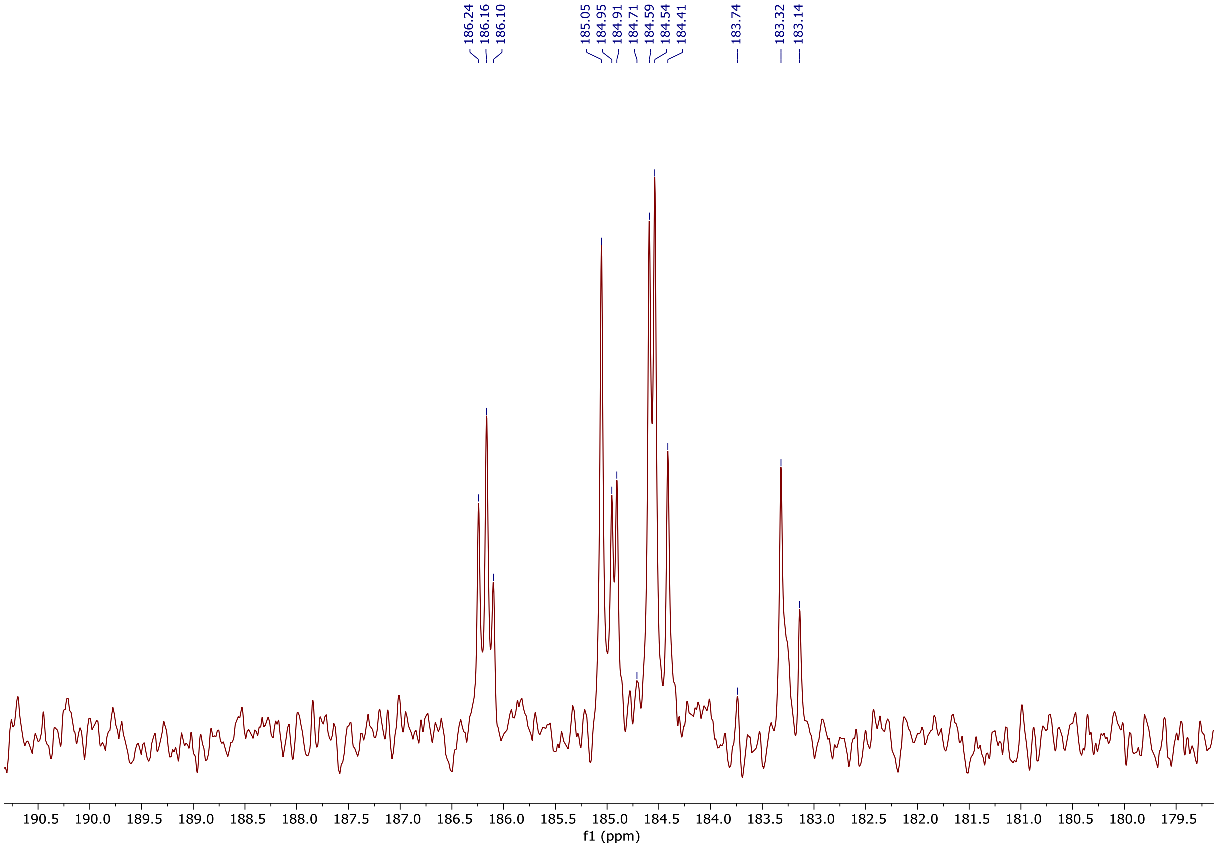
186.24
186.16
186.10
185.05
184.95
184.91
184.71
184.59
184.54
184.41
183.74
183.32
183.14
190.5 190.0 189.5 189.0 188.5 188.0 187.5 187.0 186.5 186.0 185.5 185.0 184.5 184.0 183.5 183.0 182.5 182.0 181.5 181.0 180.5 180.0 179.5
f1 (ppm)

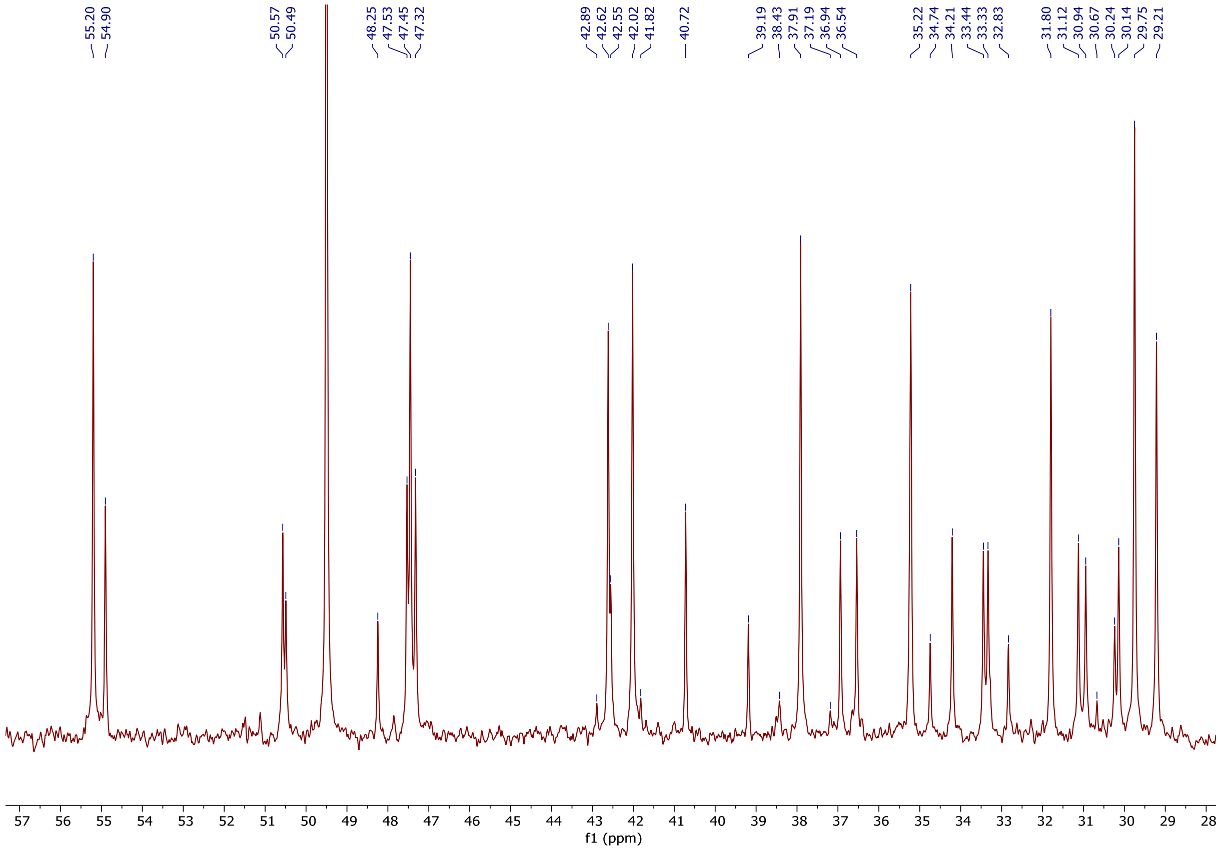

Figure S43: ^{13}C NMR spectra of compound **14** (151 MHz, 298K, 0.1M NaOD in D_2O)

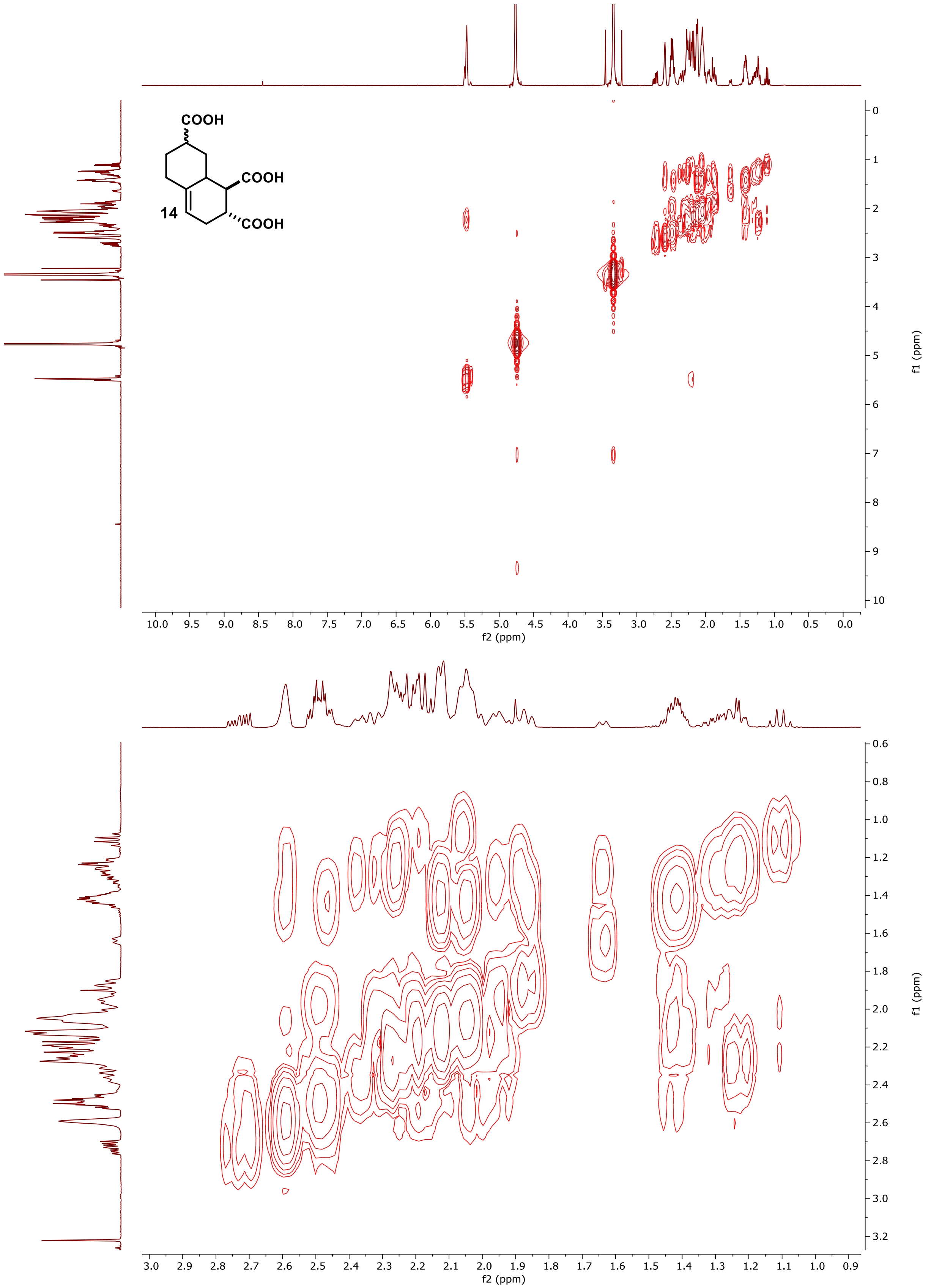

Figure S44: COSY spectra of compound **14** (600 MHz, 298K, 0.1M NaOD in D_2O)

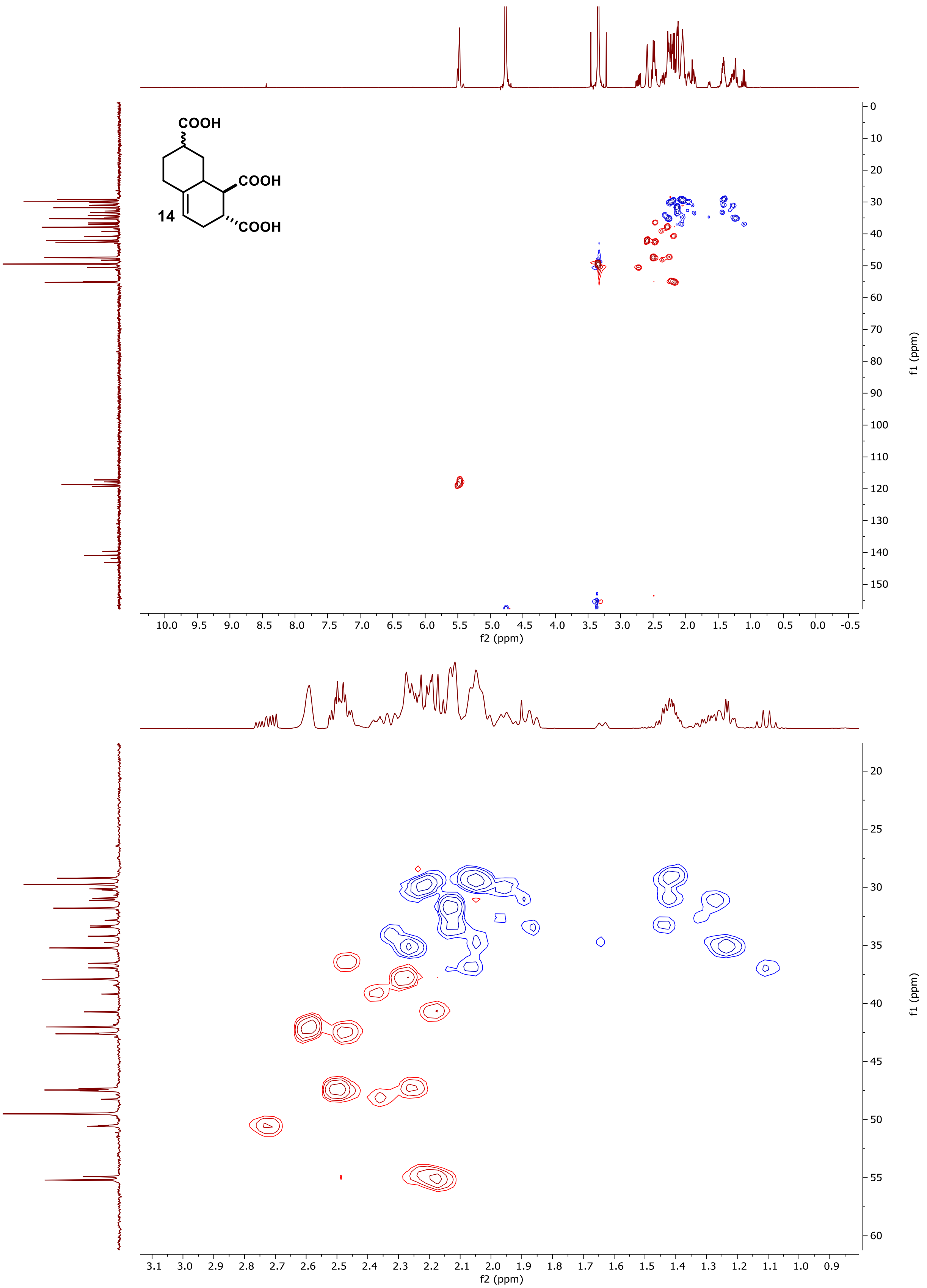

Figure S45: HSQC spectra of compound **14** (600 MHz, 298K, 0.1M NaOD in D_2O)

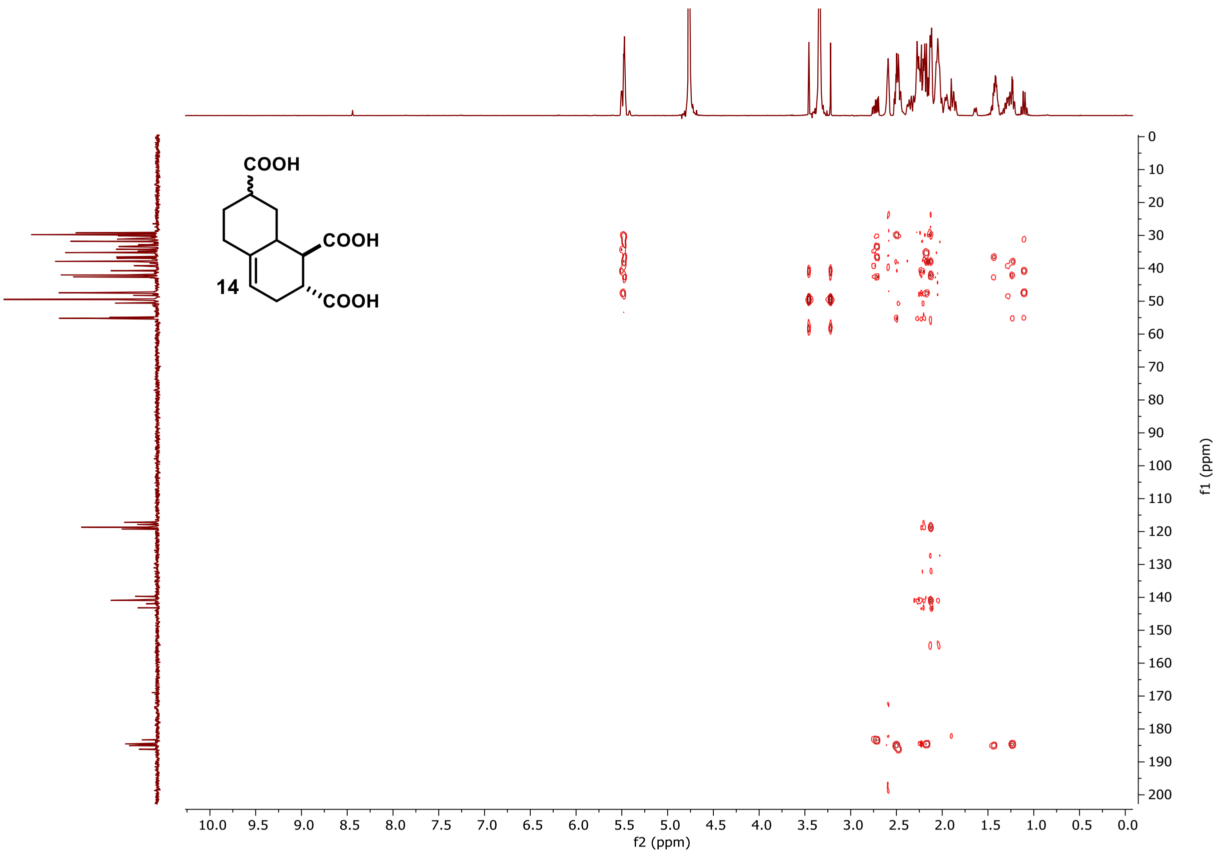
COOH
COOH
14
COOH
f2 (ppm)
f1 (ppm)

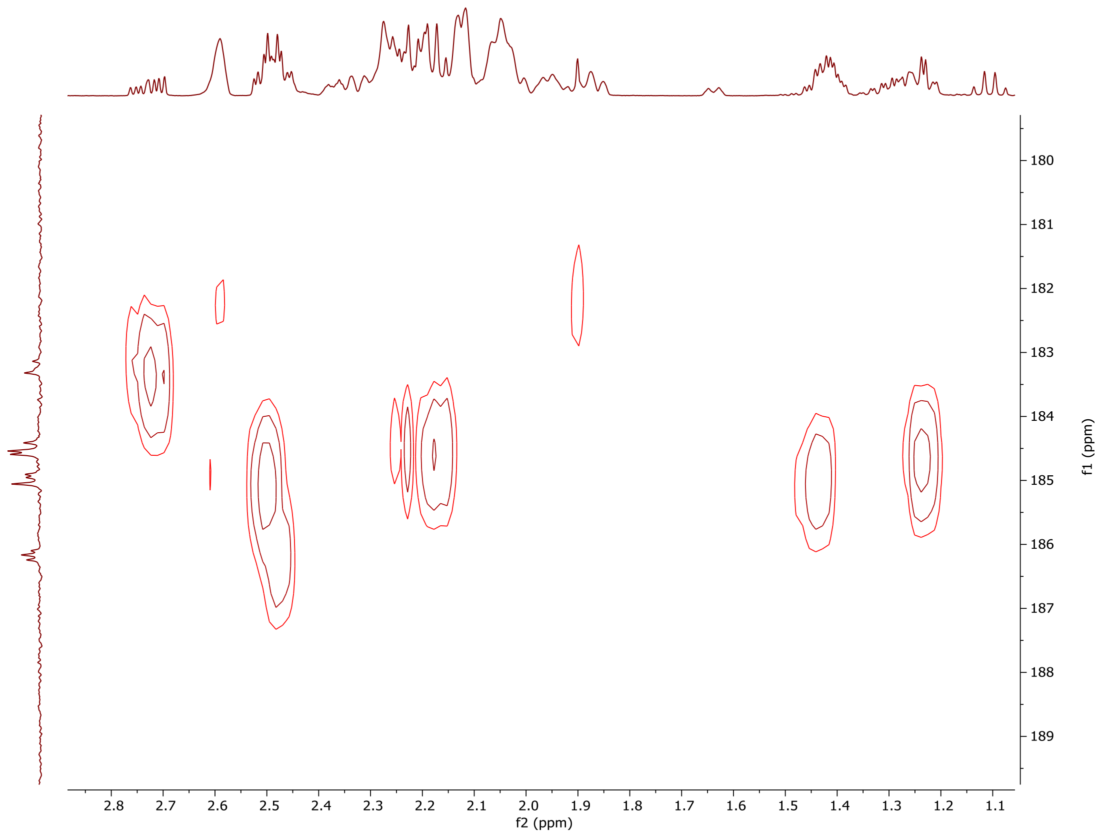
f2 (ppm)
f1 (ppm)

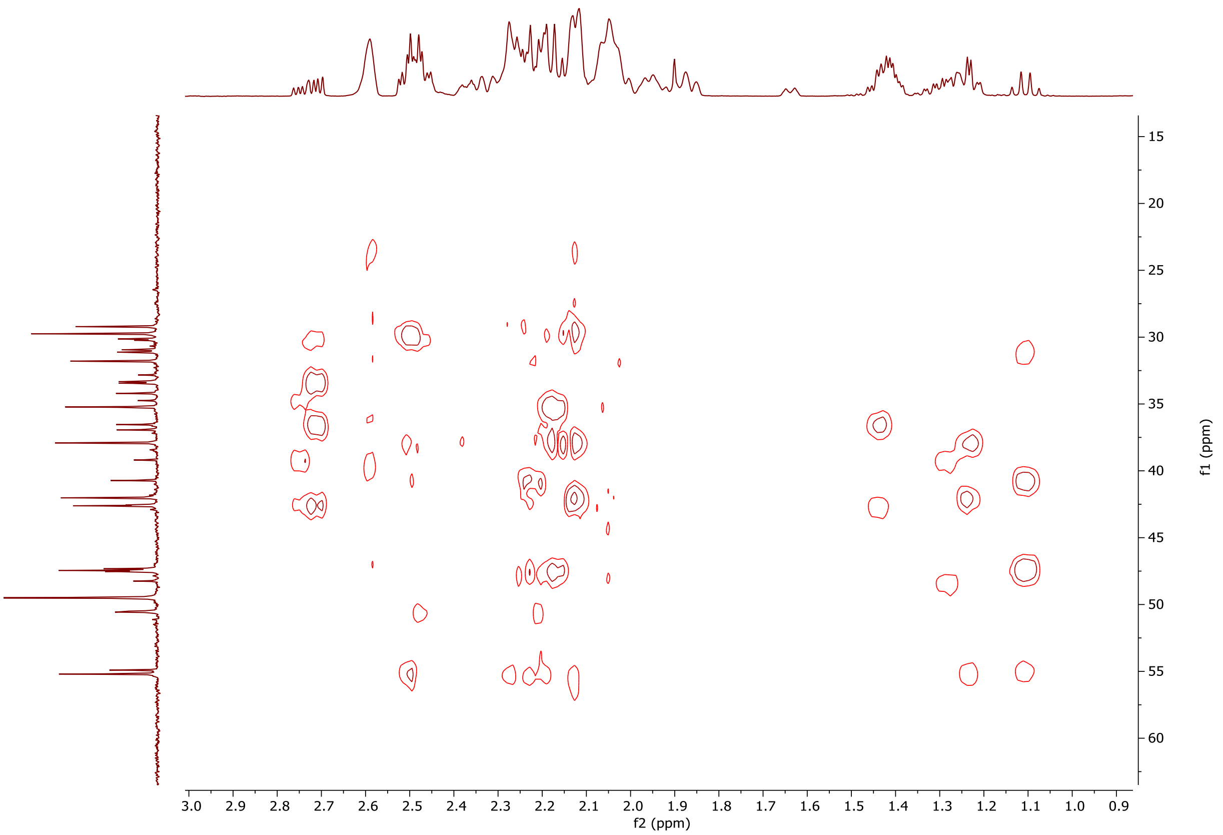

Figure S46: HMBC spectra of compound **14** (600 MHz, 298K, 0.1M NaOD in D_2O)

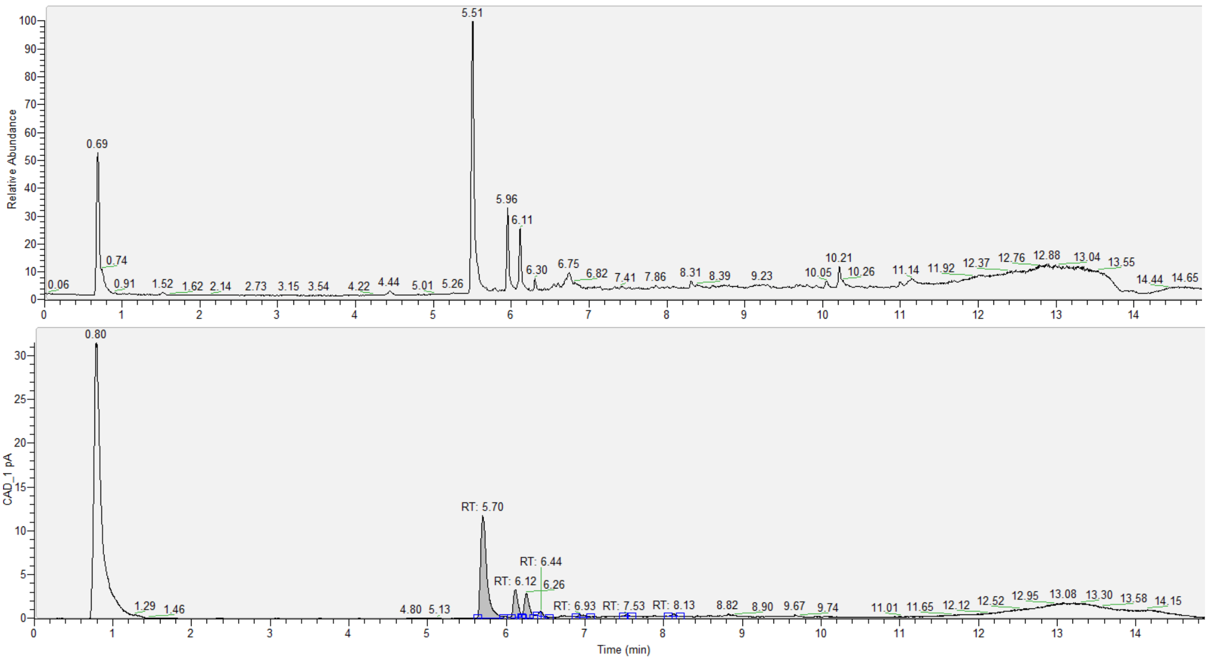

Figure S47: *TIC trace (top) and CID trace (bottom) of CRAM analogue* ***15****.*

Table S7: *LC-MS data and peak identities for CRAM analogue* ***15****.*

Apex RT	Start RT	End RT	Area	%Area	*m/z*	Identity
5.70	5.63	5.96	65.586	67.96	311	title compound **15** isomer
6.12	6.06	6.20	13.440	13.93	311	title compound **15** isomer
6.26	6.20	6.35	12.193	12.63	311	title compound **15** isomer
6.44	6.39	6.55	2.099	2.17	337	mono ethyl ester
6.93	6.88	7.07	1.430	1.48	325	unidentified impurity
7.53	7.48	7.60	0.771	0.80	353	unidentified impurity
8.13	8.05	8.21	0.988	1.02	367	unidentified impurity

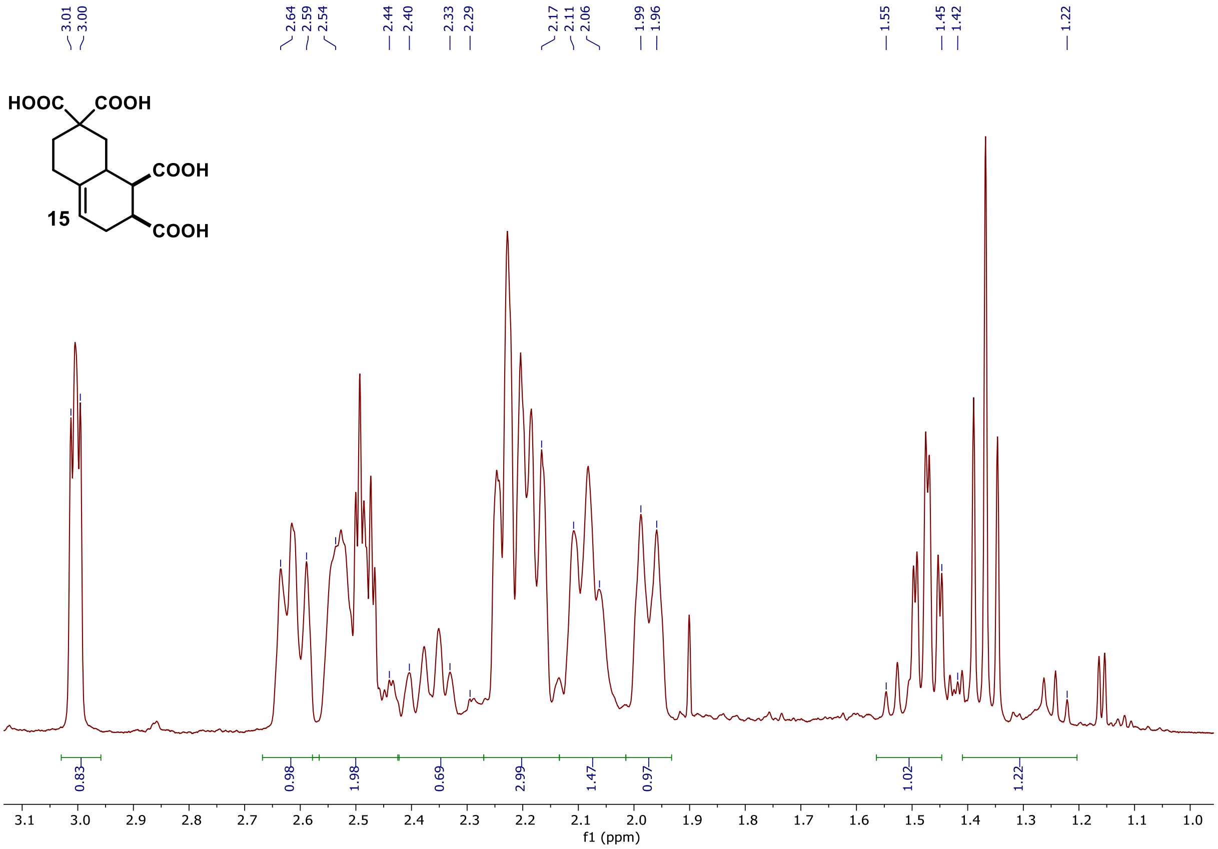

Figure S48: ^{1}H NMR spectra of compound **15** (600 MHz, 298K, 0.1M NaOD in D_2O)

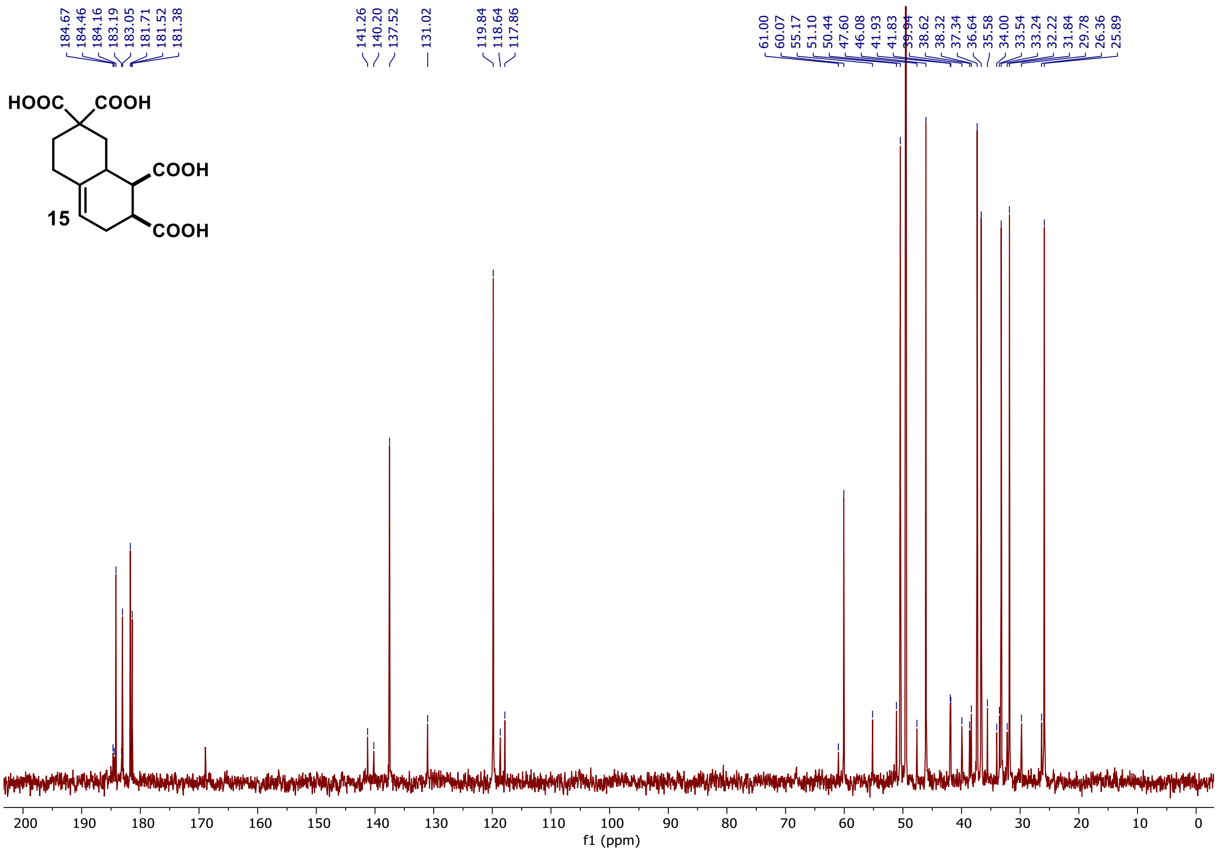

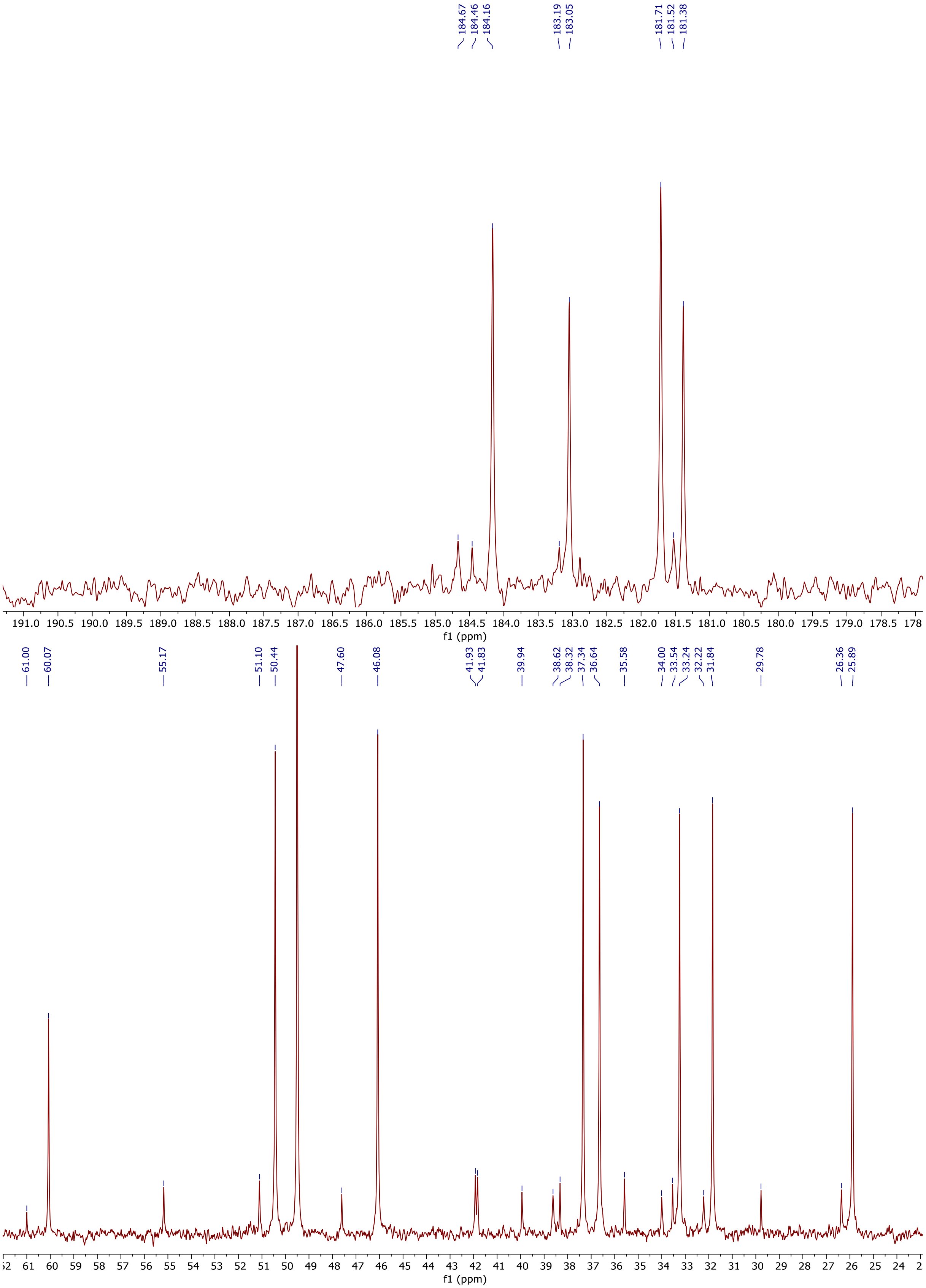

Figure S49: ^{13}C NMR spectra of compound **15** (151 MHz, 298K, 0.1M NaOD in D_2O)

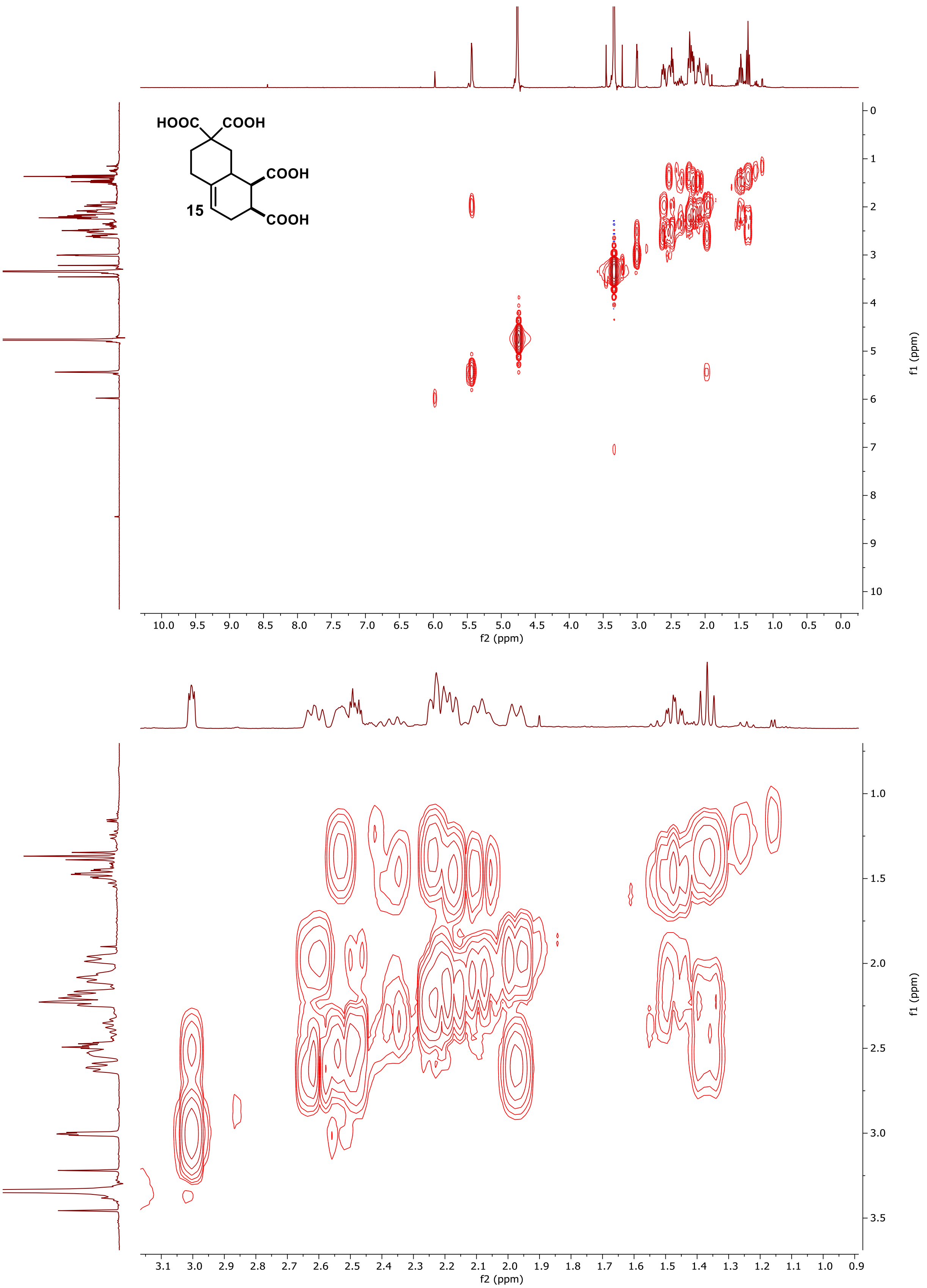

Figure S50: COSY spectra of compound **15** (600 MHz, 298K, 0.1M NaOD in D_2O)

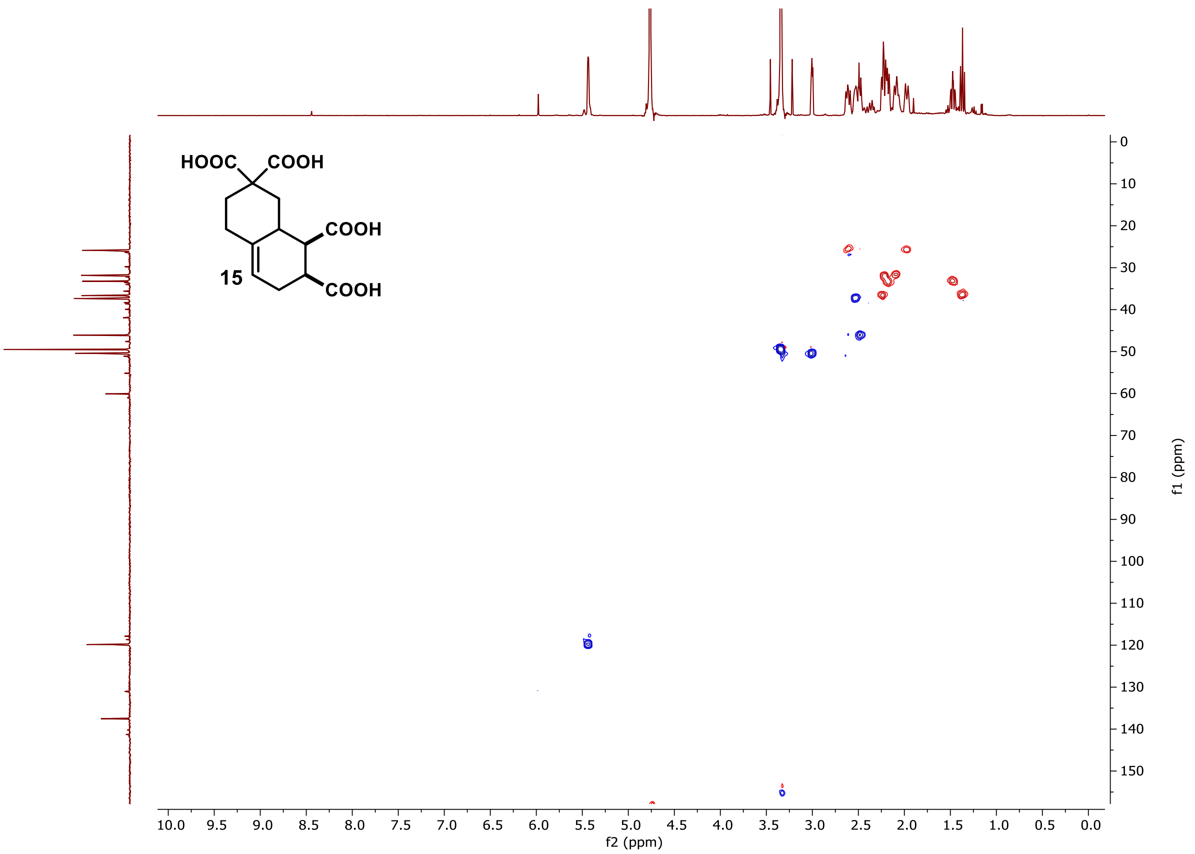

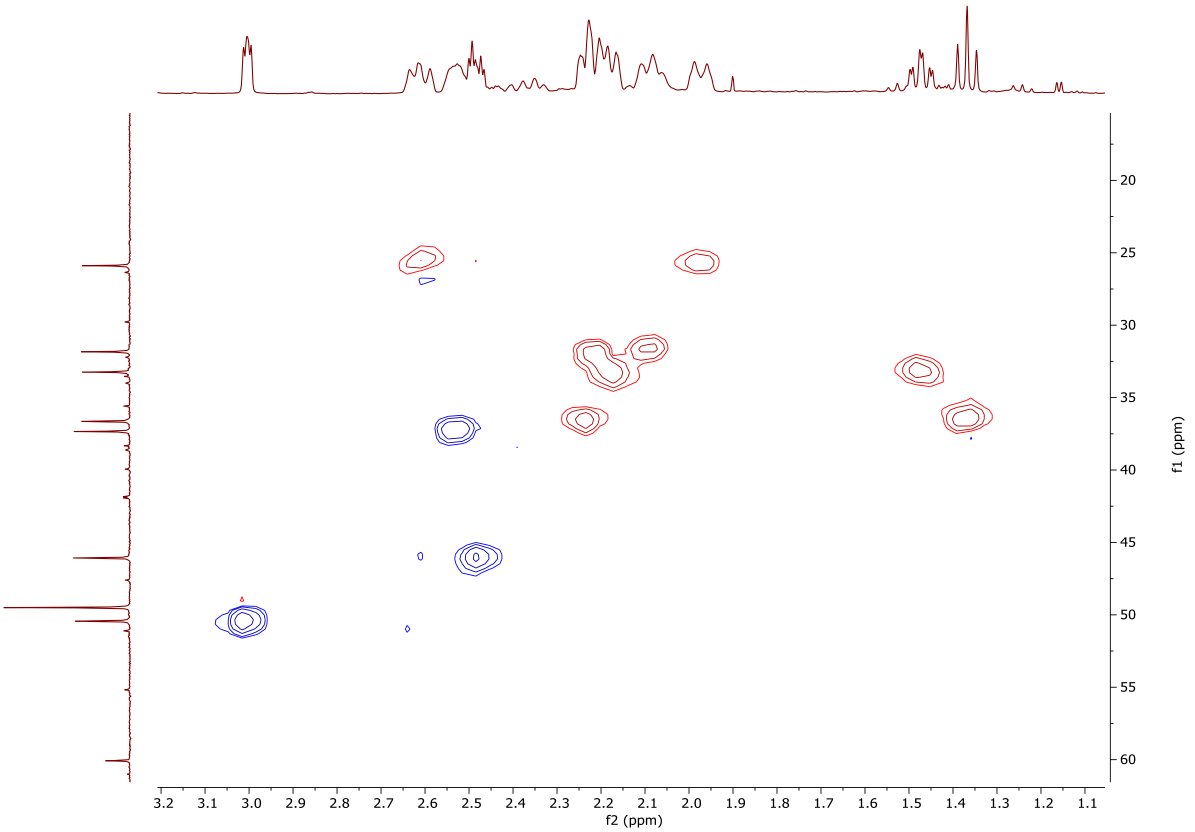

Figure S51: HSQC spectra of compound **15** (600 MHz, 298K, 0.1M NaOD in D_2O)

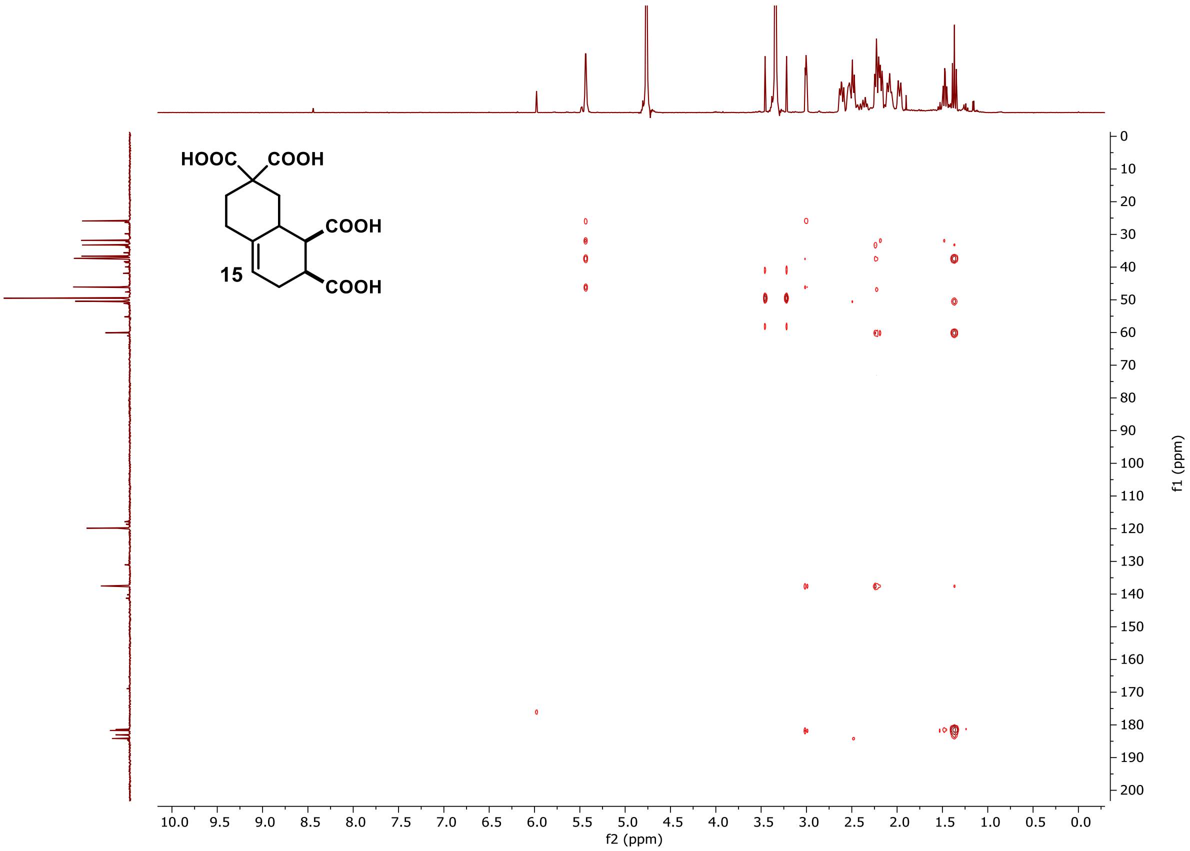
HOOC
COOH
COOH
15
COOH
f1 (ppm)
f2 (ppm)

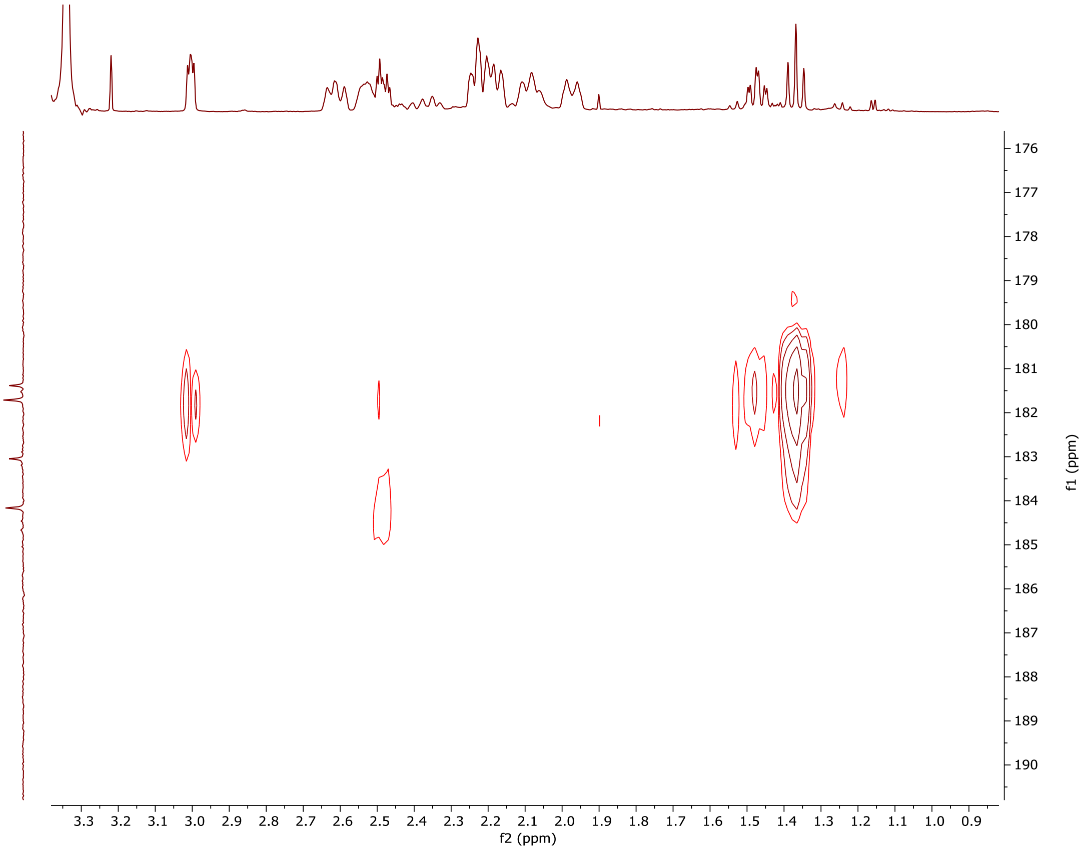
f1 (ppm)
f2 (ppm)

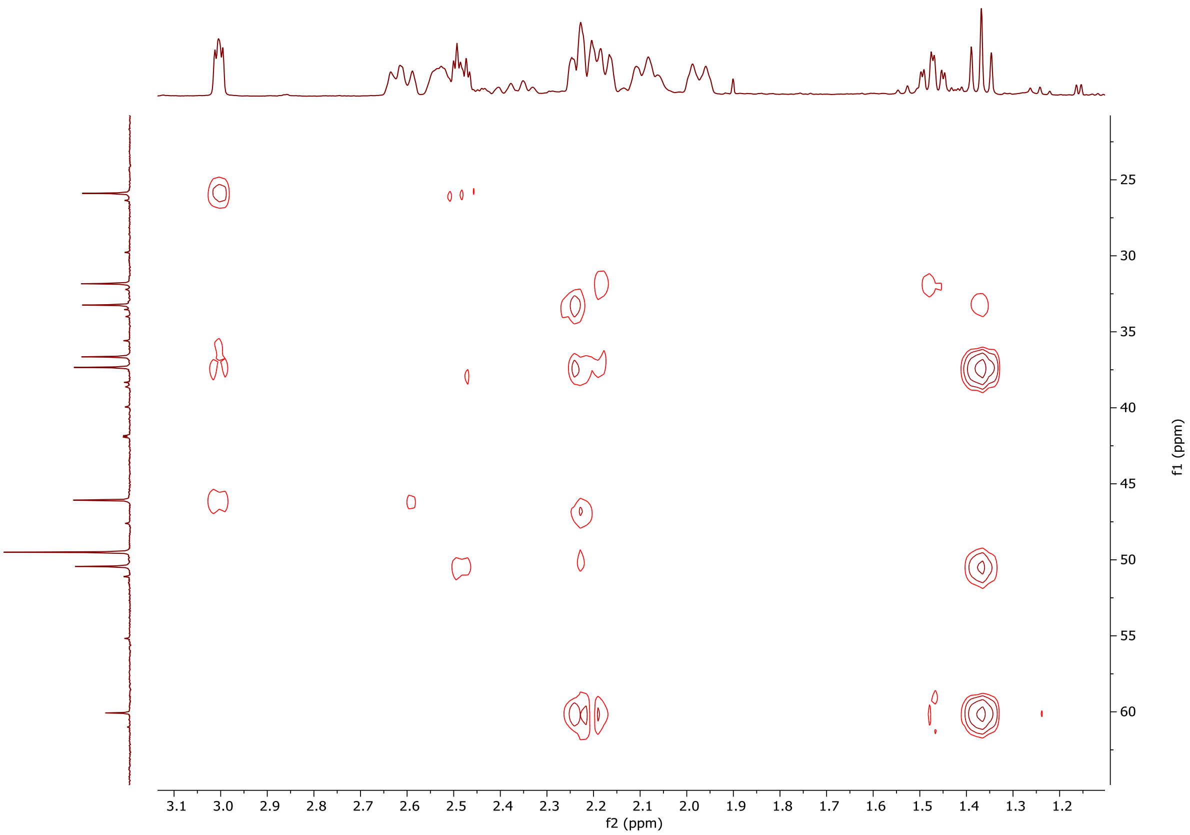

Figure S52: HMBC spectra of compound **15** (600 MHz, 298K, 0.1M NaOD in D_2O)

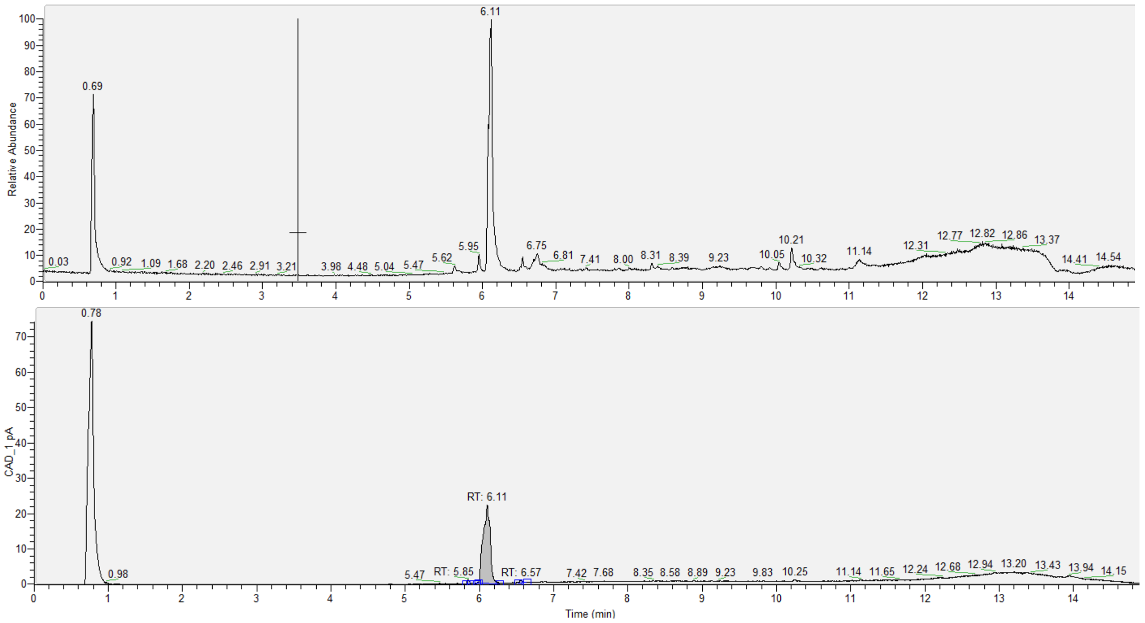

Figure S53: *TIC trace (top) and CID trace (bottom) of CRAM analogue **16**.*

Table S8: *LC-MS data and peak identities for CRAM analogue **16**.*

Apex RT	Start RT	End RT	Area	%Area	*m/z*	Identity
5.85	5.82	5.89	2.247	1.50	307	unidentified impurity
5.97	5.96	5.99	0.243	0.16	311	title compound **16** isomer
6.11	5.99	6.27	144.947	96.96	311	title compound **16** isomer
6.57	6.52	6.64	2.061	1.38	337	unidentified impurity

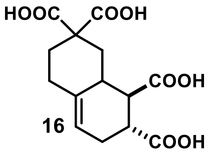

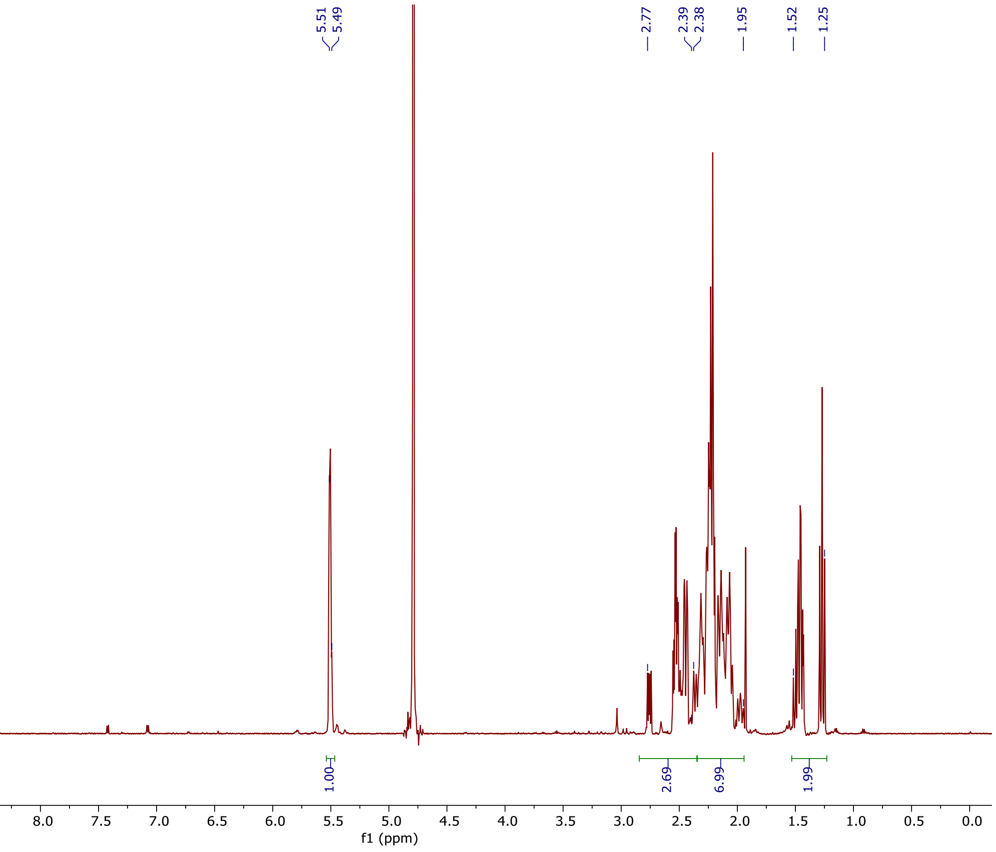

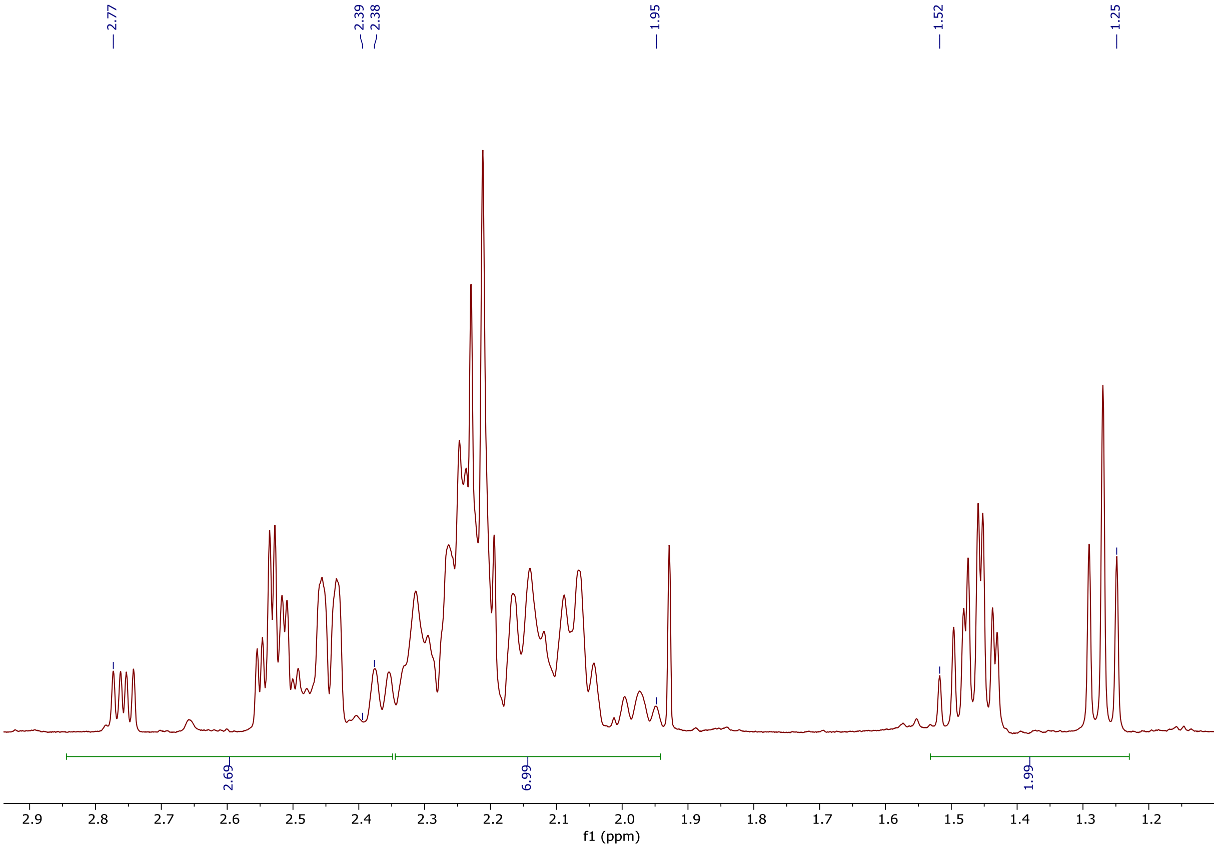

Figure S54: ^{1}H NMR spectra of compound **16** (600 MHz, 298K, 0.1M NaOD in D_2O)

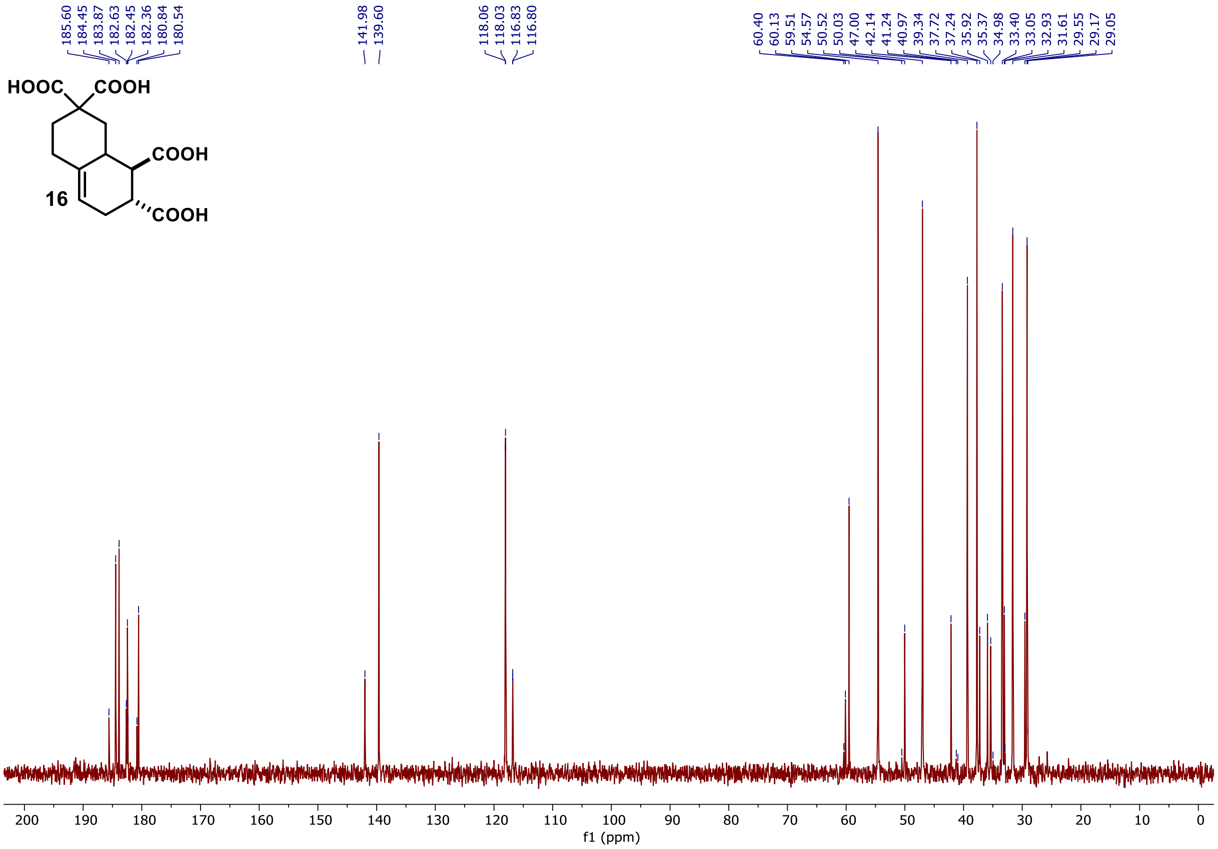
HOOC
COOH
COOH
COOH
16
185.60
184.45
183.87
182.63
182.45
182.36
180.84
180.54
141.98
139.60
118.06
118.03
116.83
116.80
60.40
60.13
59.51
54.57
50.52
50.03
47.00
42.14
41.24
40.97
39.34
37.72
37.24
35.92
35.37
34.98
33.40
33.05
32.93
31.61
29.55
29.17
29.05
200 190 180 170 160 150 140 130 120 110 100 90 80 70 60 50 40 30 20 10 0
f1 (ppm)

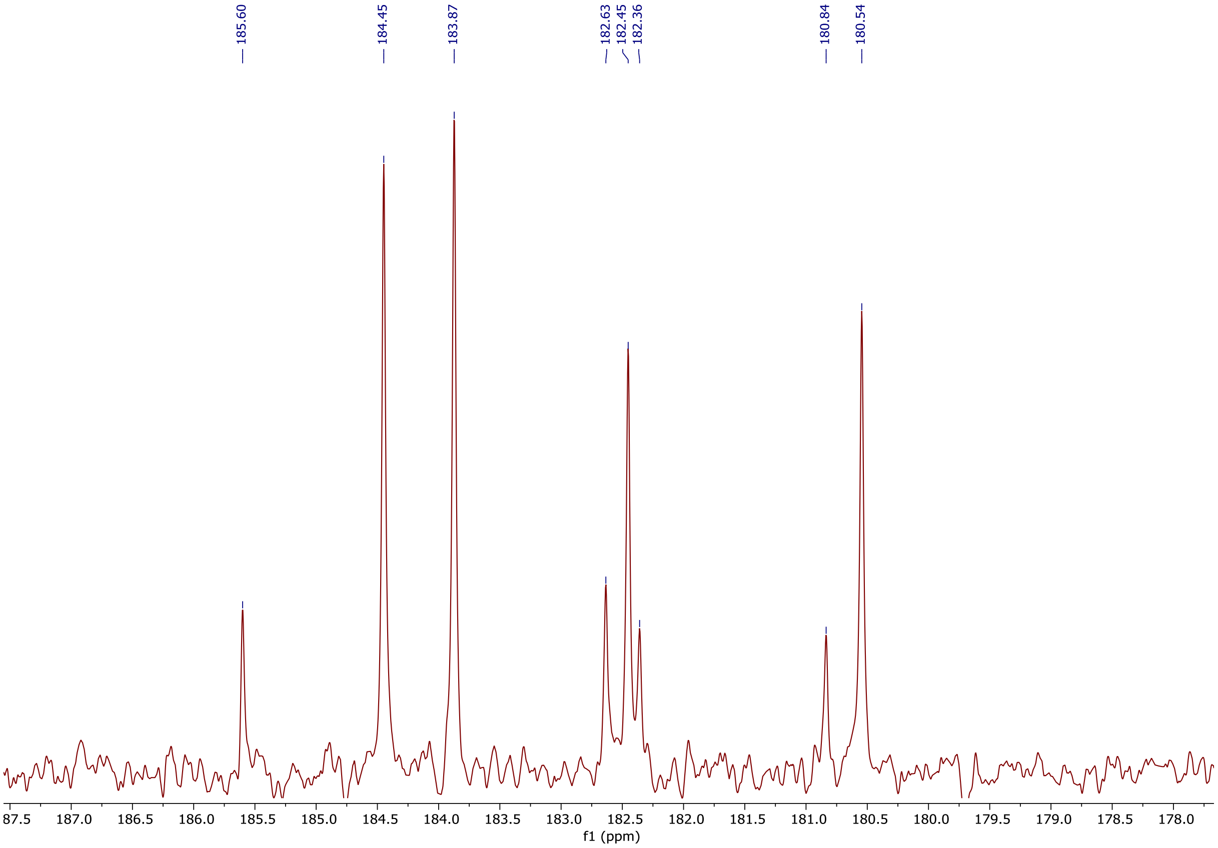
185.60
184.45
183.87
182.63
182.45
182.36
180.84
180.54
87.5 187.0 186.5 186.0 185.5 185.0 184.5 184.0 183.5 183.0 182.5 182.0 181.5 181.0 180.5 180.0 179.5 179.0 178.5 178.0
f1 (ppm)

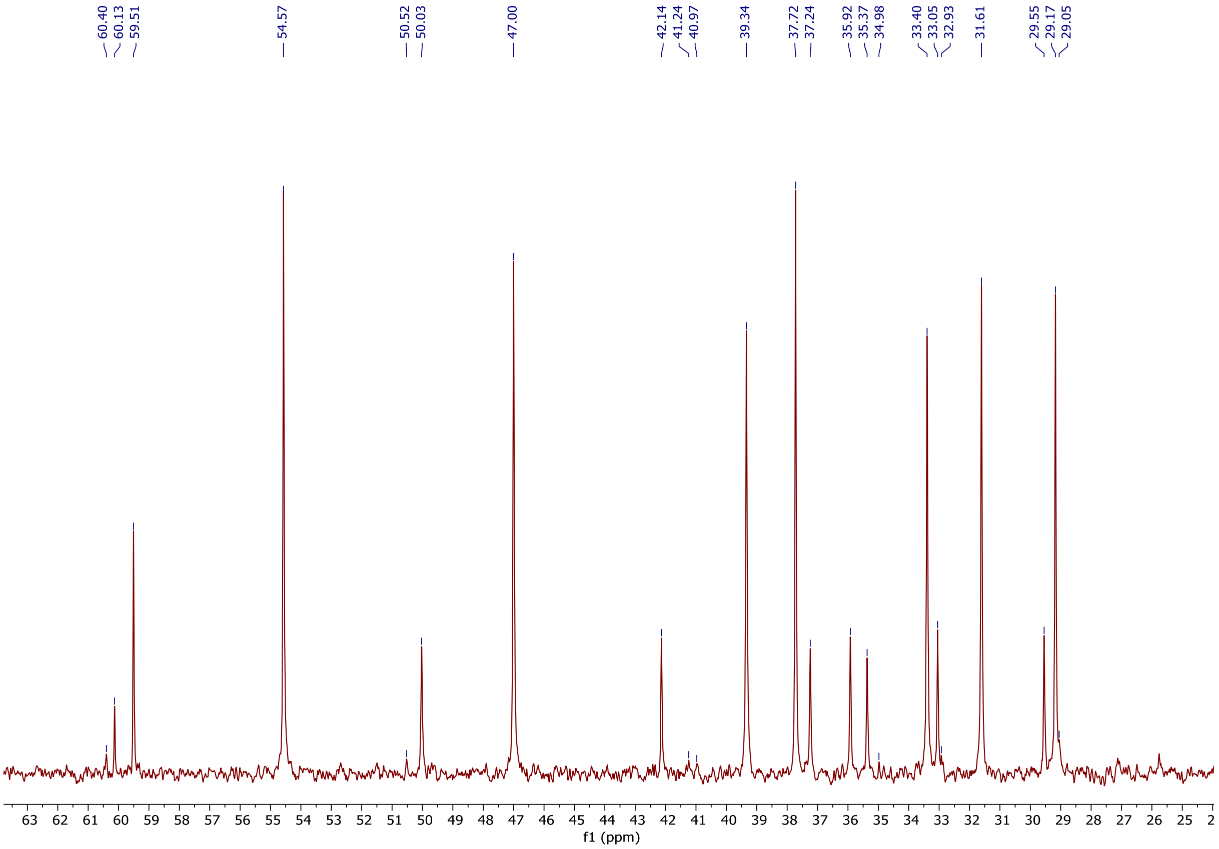

Figure S55: ^{13}C NMR spectra of compound **16** (151 MHz, 298K, 0.1M NaOD in D_2O)

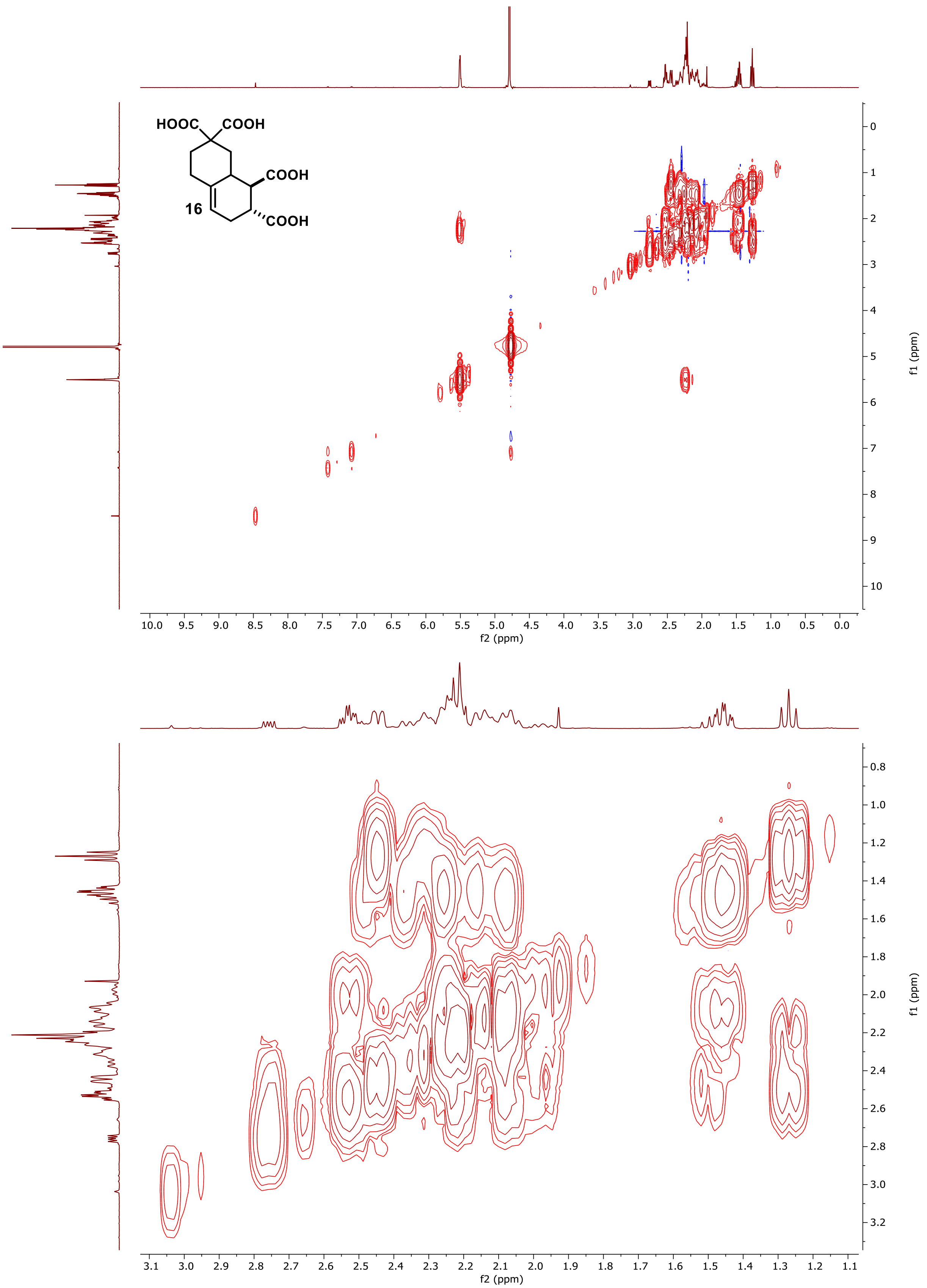

Figure S56: COSY spectra of compound **16** (600 MHz, 298K, 0.1M NaOD in D_2O)

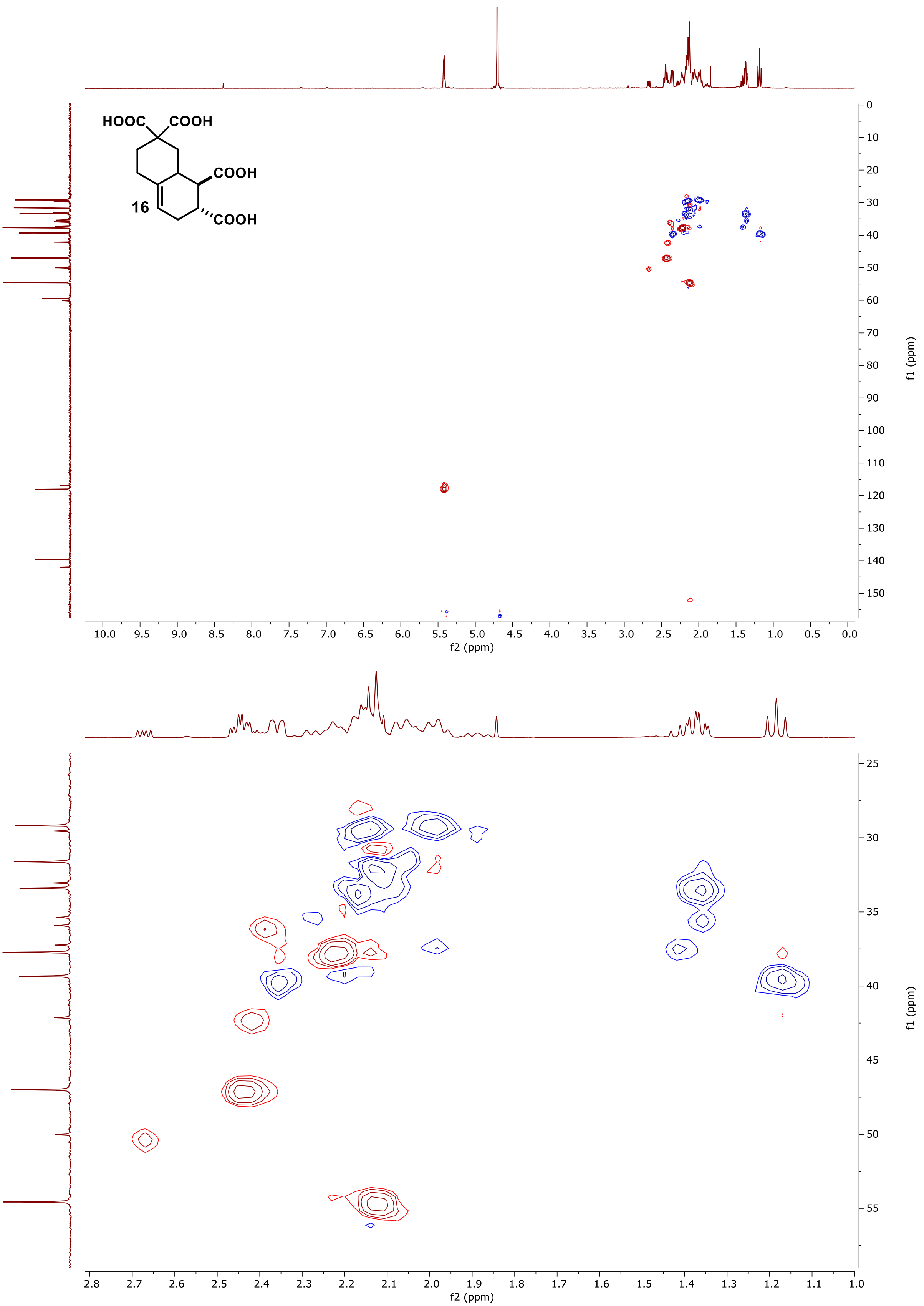

Figure S57: HSQC spectra of compound **16** (600 MHz, 298K, 0.1M NaOD in D_2O)

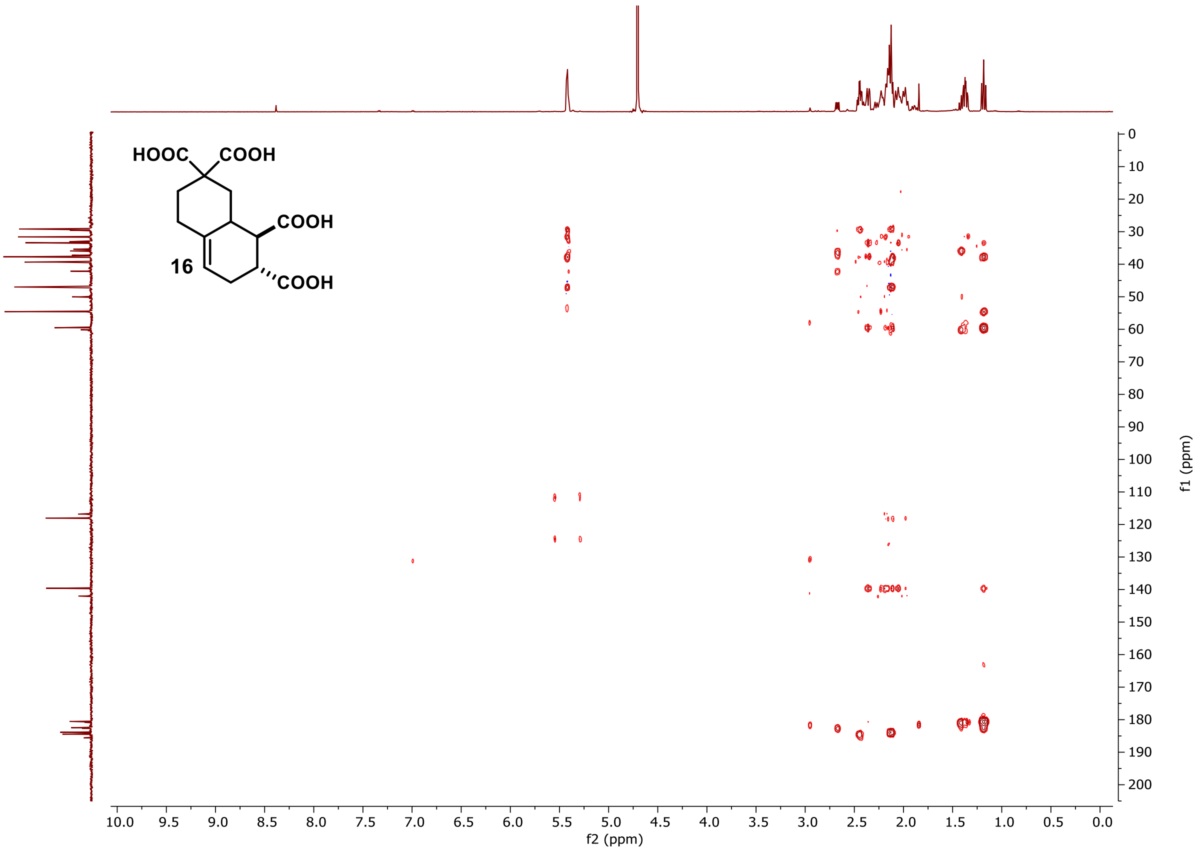
HOOC
COOH
COOH
16
COOH
f2 (ppm)
f1 (ppm)

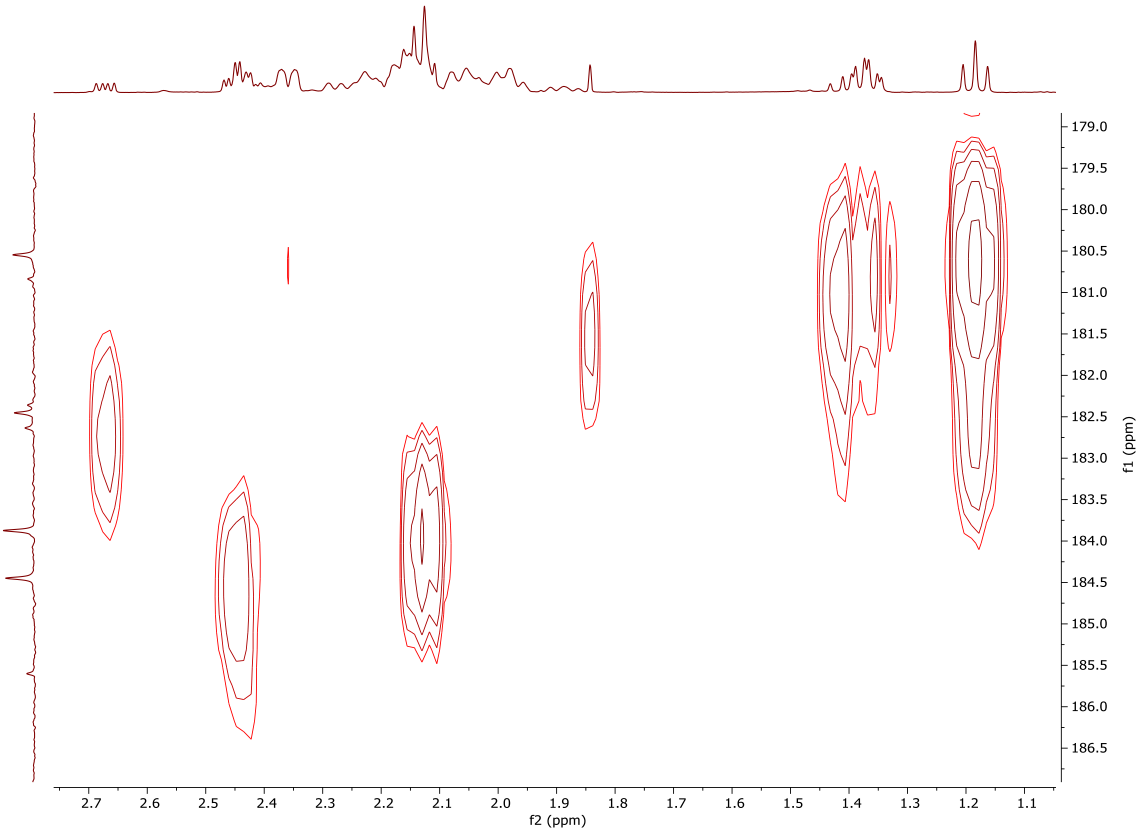
f2 (ppm)
f1 (ppm)

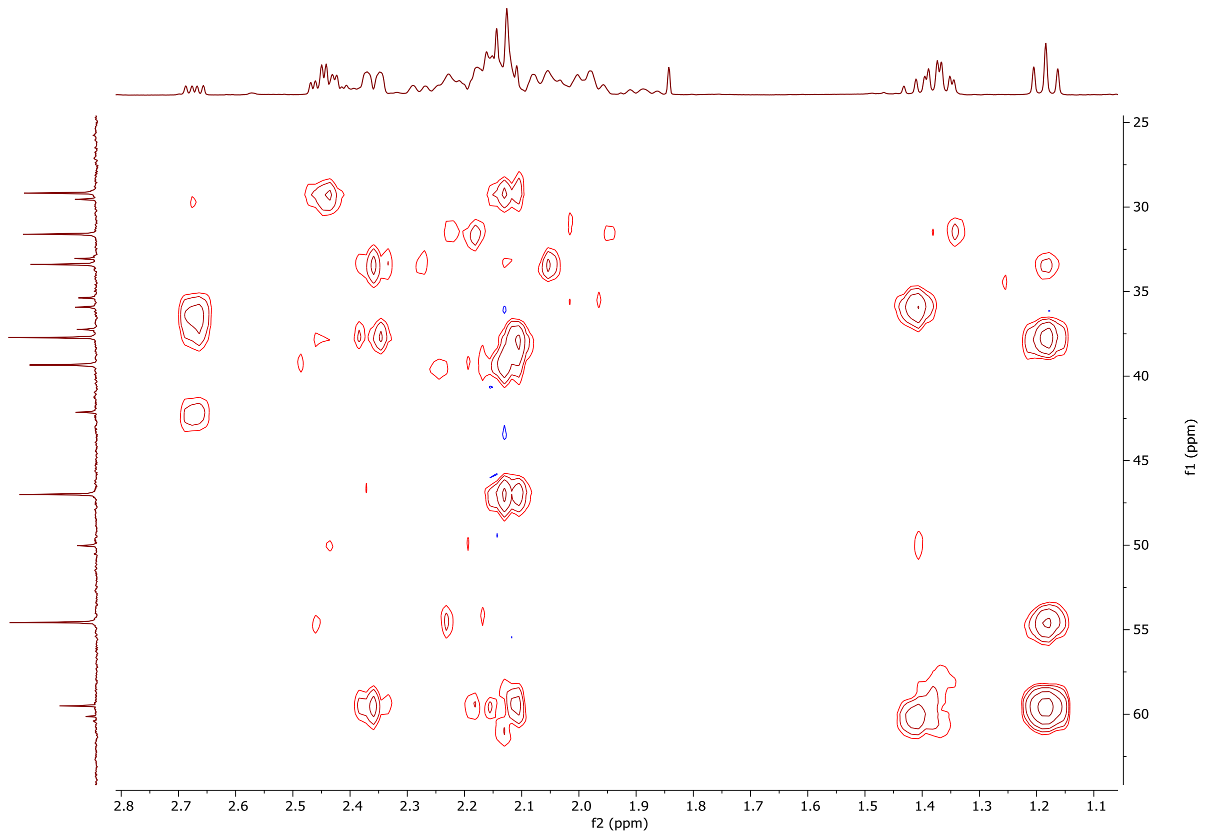

Figure S58: HMBC spectra of compound **16** (600 MHz, 298K, 0.1M NaOD in D_2O)

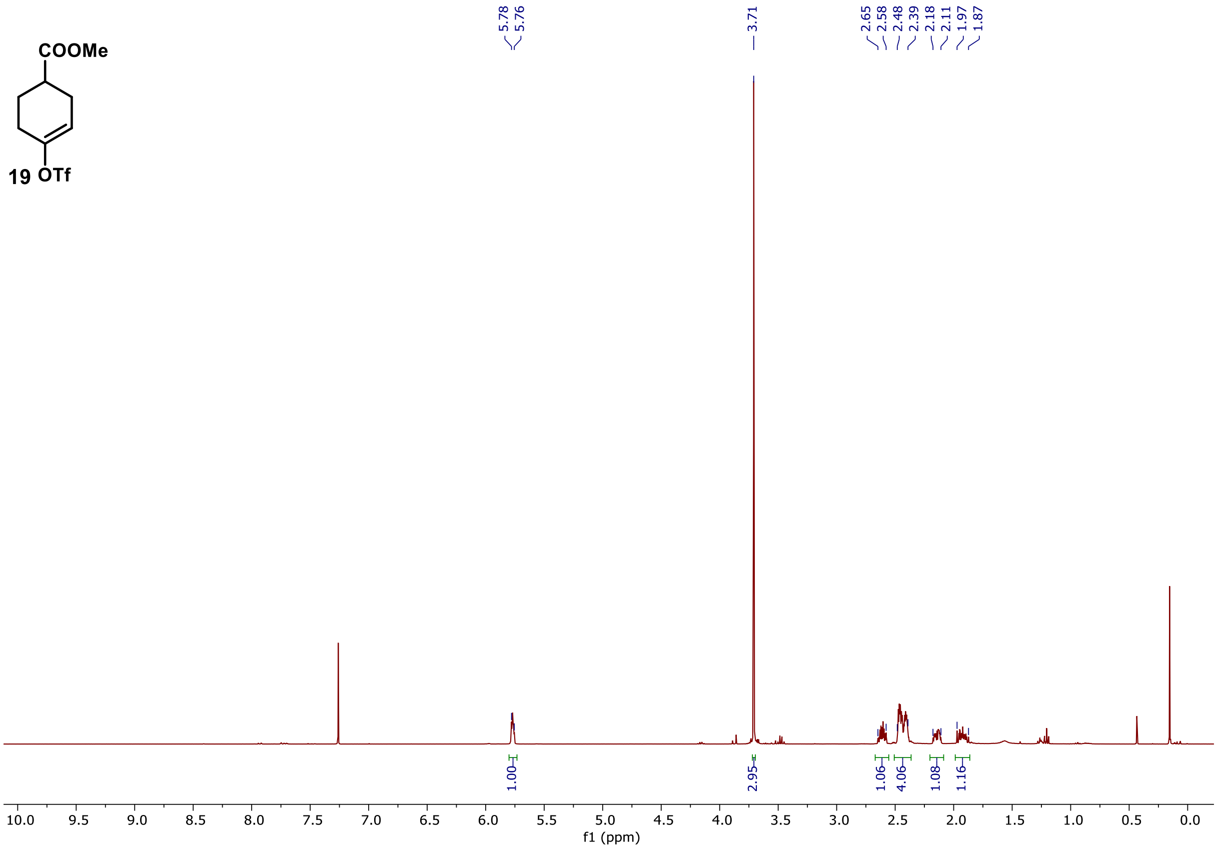

Figure S59: ^{1}H NMR spectrum of compound **19** (400 MHz, 298K, $CDCl_3$)

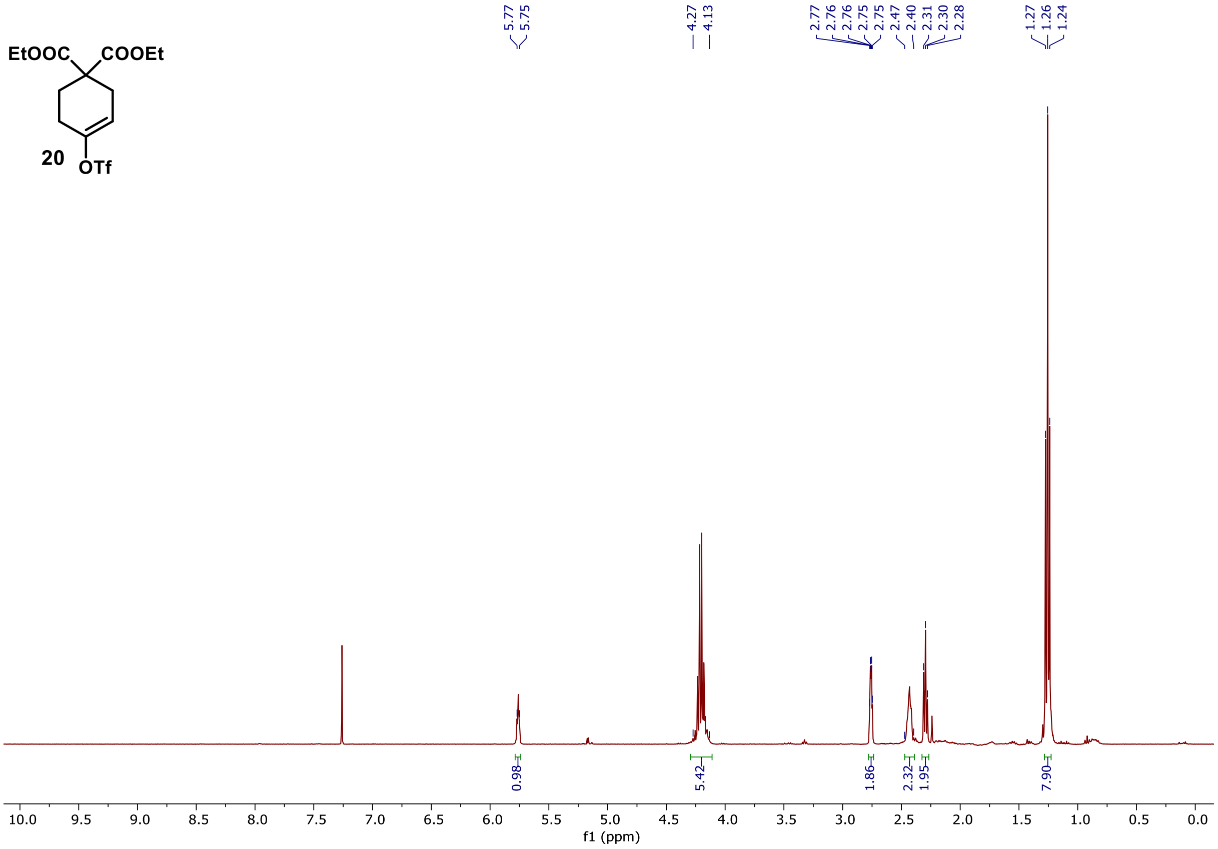

Figure S60: ^{1}H NMR spectrum of compound **20** (400 MHz, 298K, $CDCl_3$)

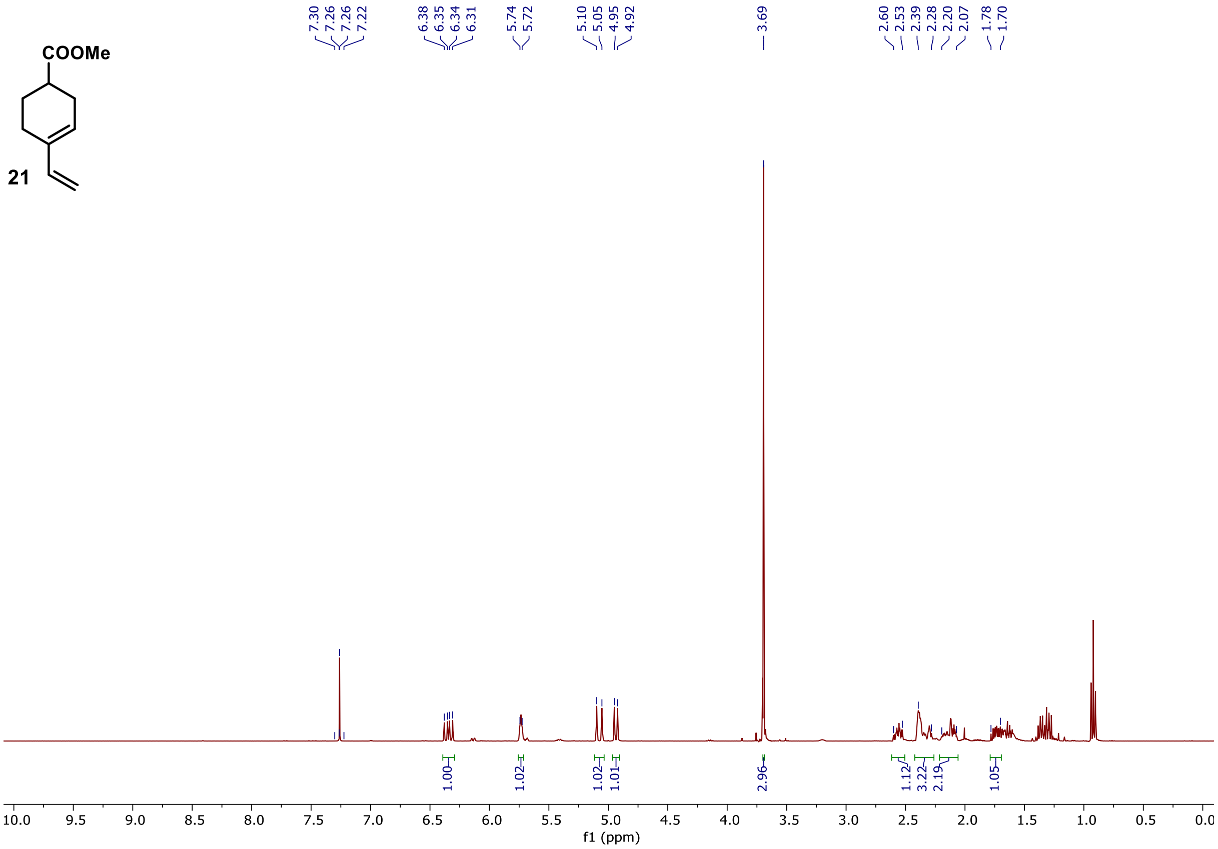

Figure S61: ^{1}H NMR spectrum of compound **21** (400 MHz, 298K, $CDCl_3$)

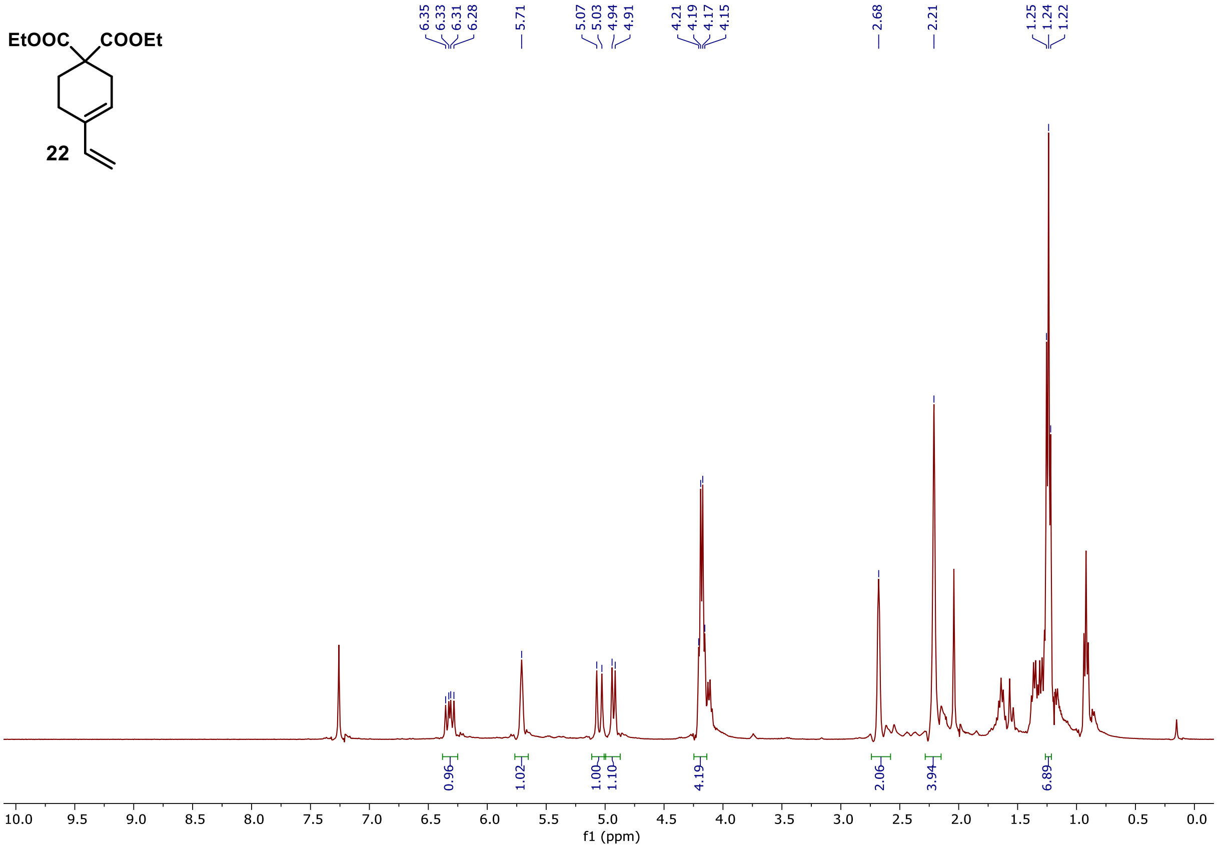

Figure S62: ^{1}H NMR spectrum of compound **22** (400 MHz, 298K, $CDCl_3$)

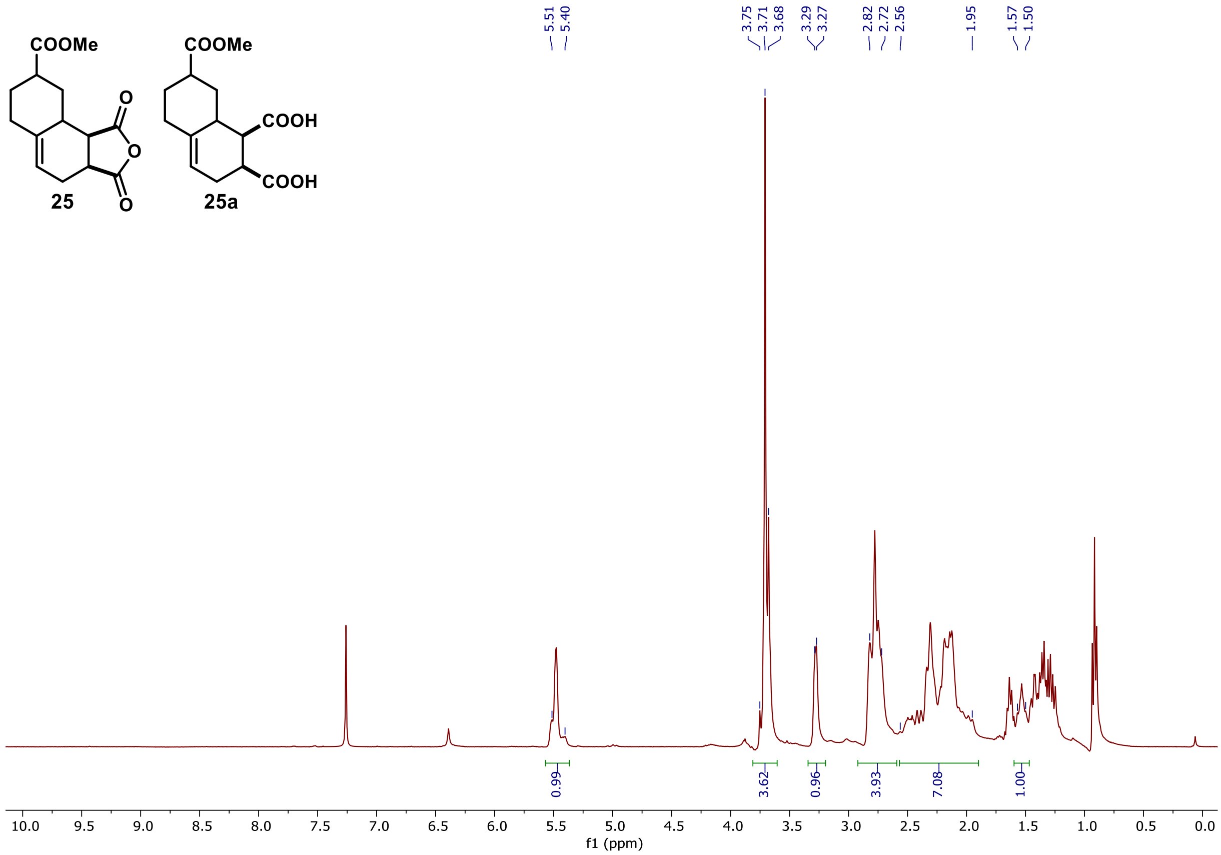

Figure S63: ^{1}H NMR spectrum of compound **25/25a** (400 MHz, 298K, $CDCl_3$)

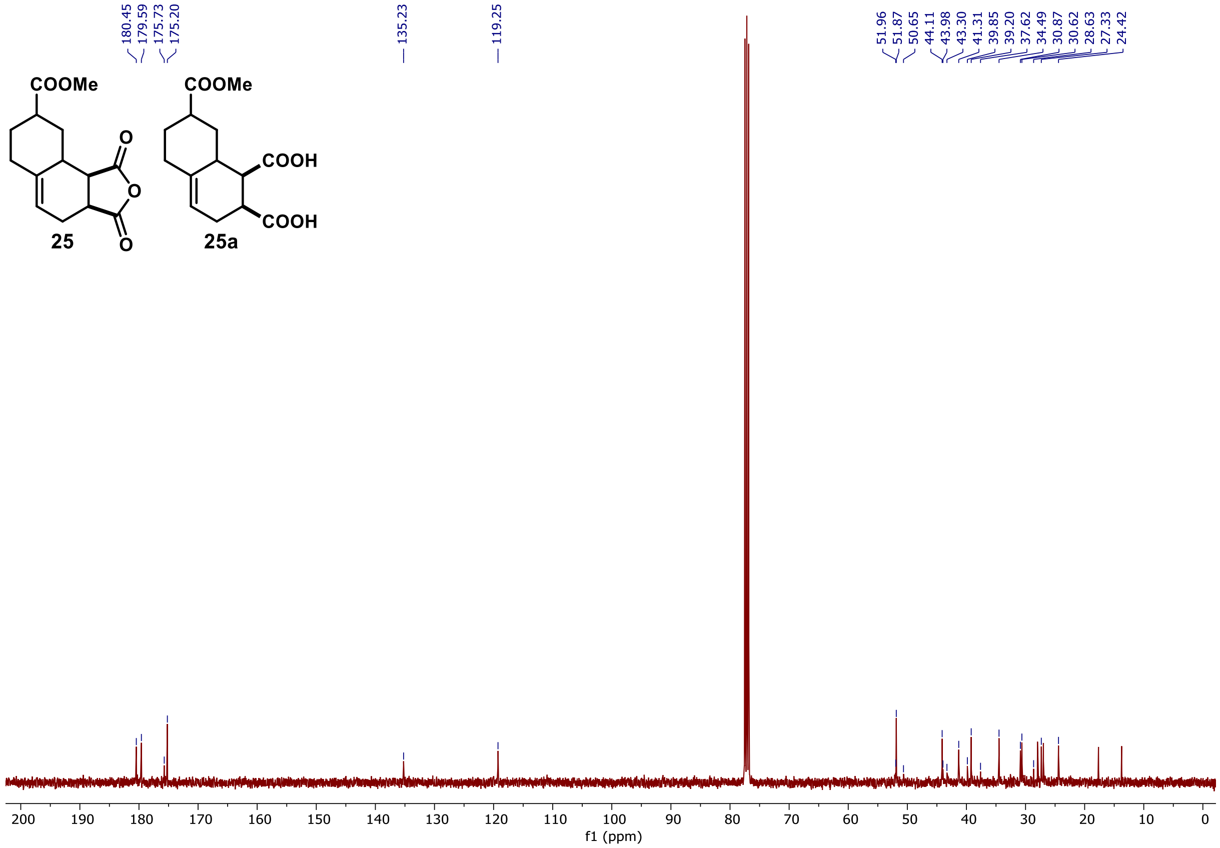

Figure S64: ^{13}C NMR spectrum of compound **25/25a** (101 MHz, 298K, $CDCl_3$)

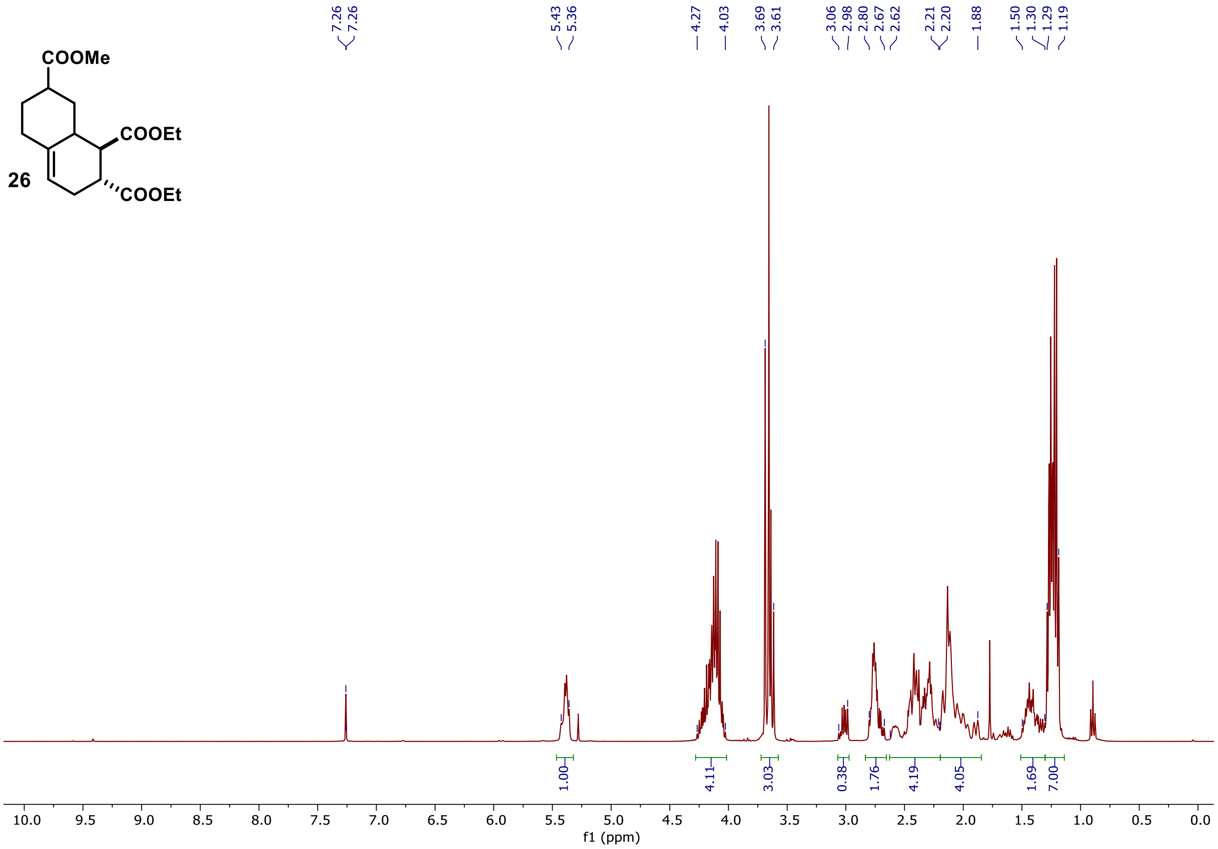

Figure S65: ^{1}H NMR spectrum of compound **26** (400 MHz, 298K, $CDCl_3$)

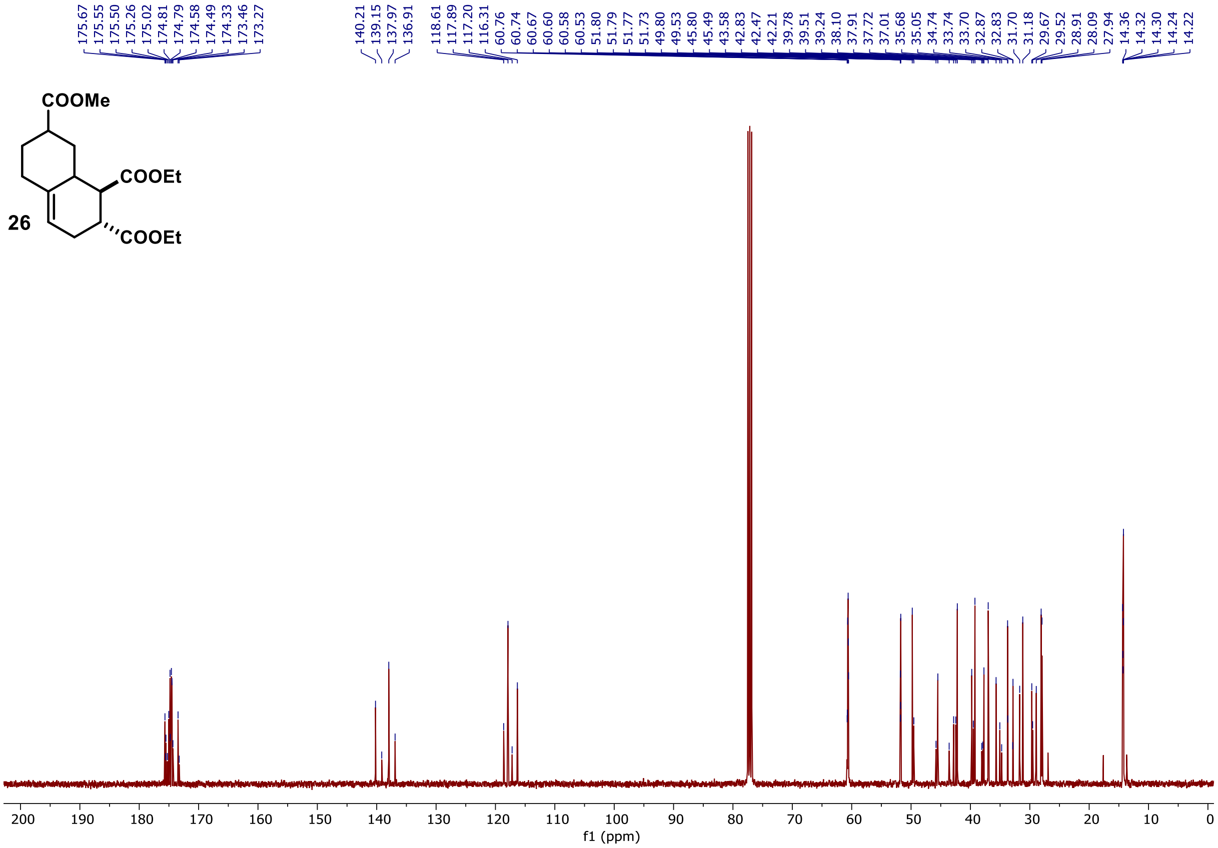

Figure S66: ^{13}C NMR spectrum of compound **26** (101 MHz, 298K, $CDCl_3$)

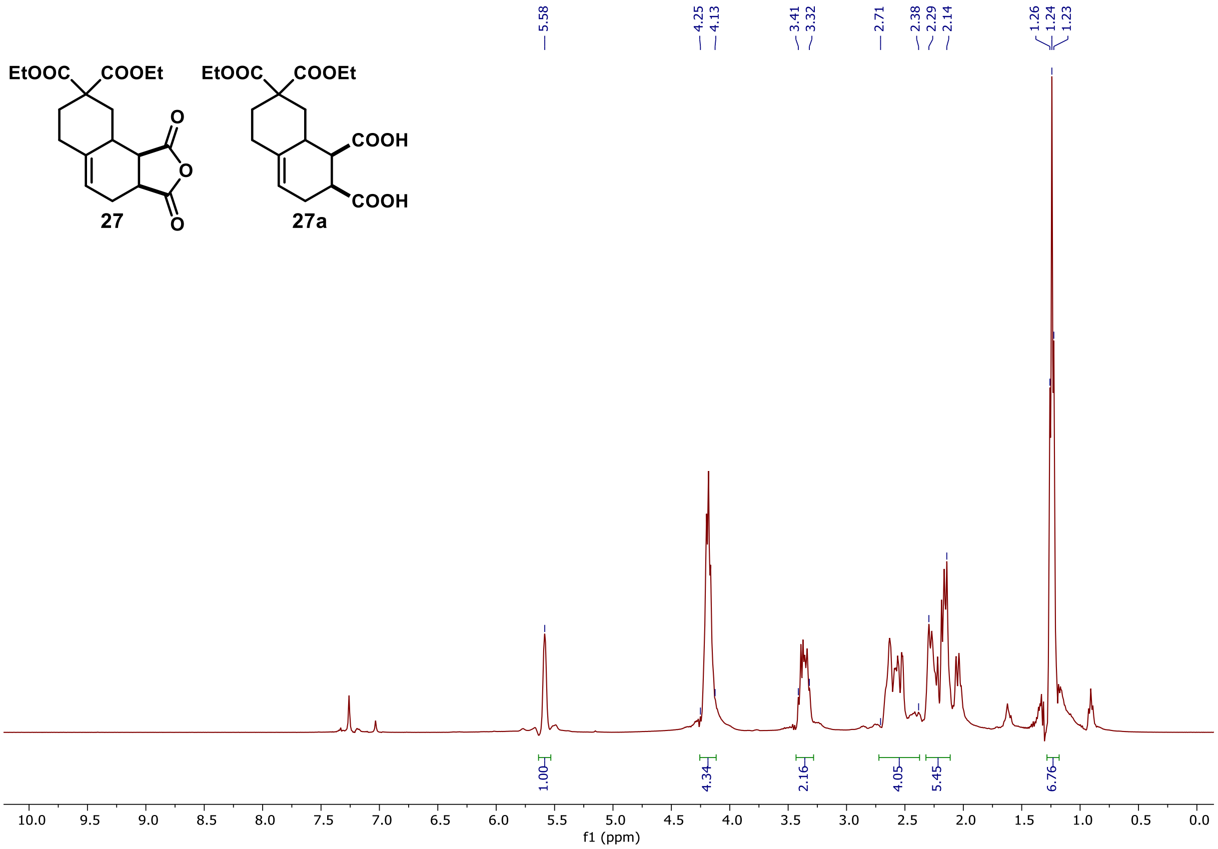

Figure S67: ^{1}H NMR spectrum of compound **27/27a** (400 MHz, 298K, $CDCl_3$)

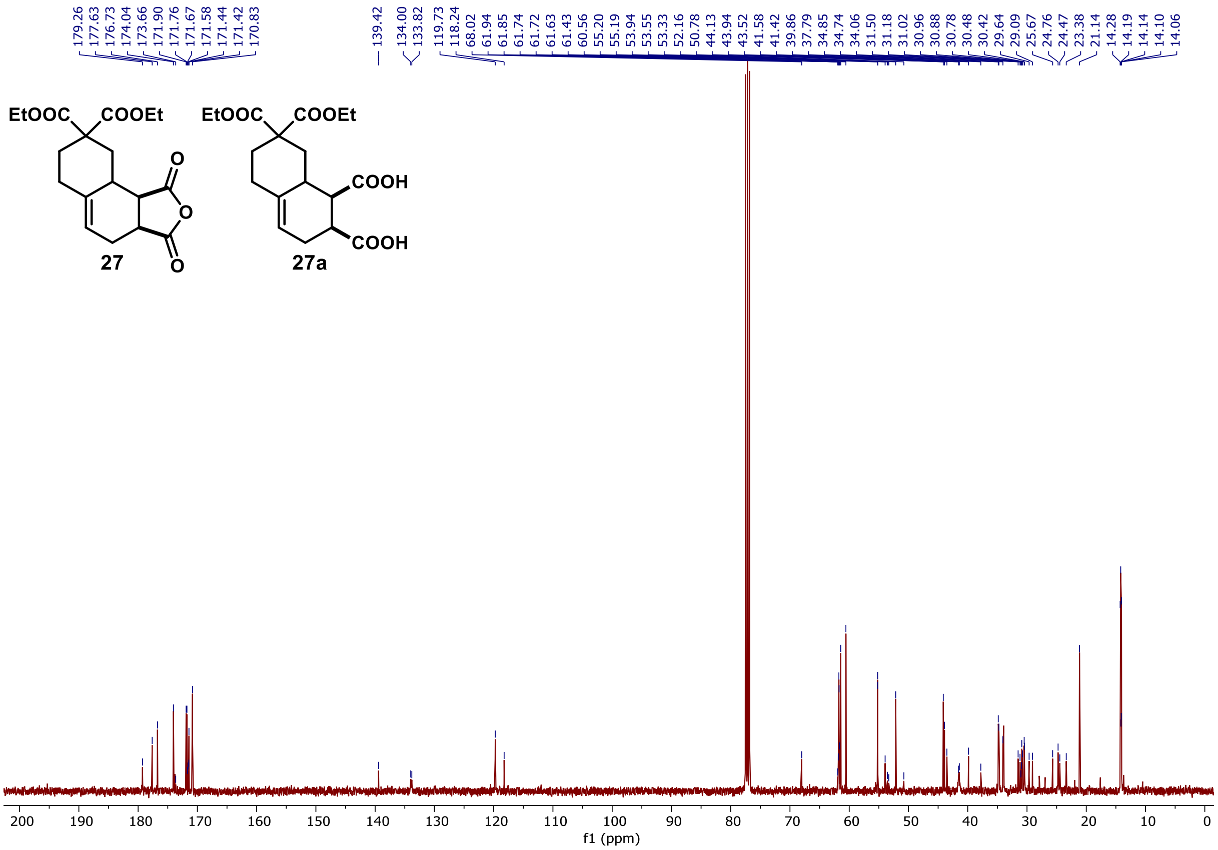

Figure S68: ^{13}C NMR spectrum of compound **27/27a** (101 MHz, 298K, $CDCl_3$)

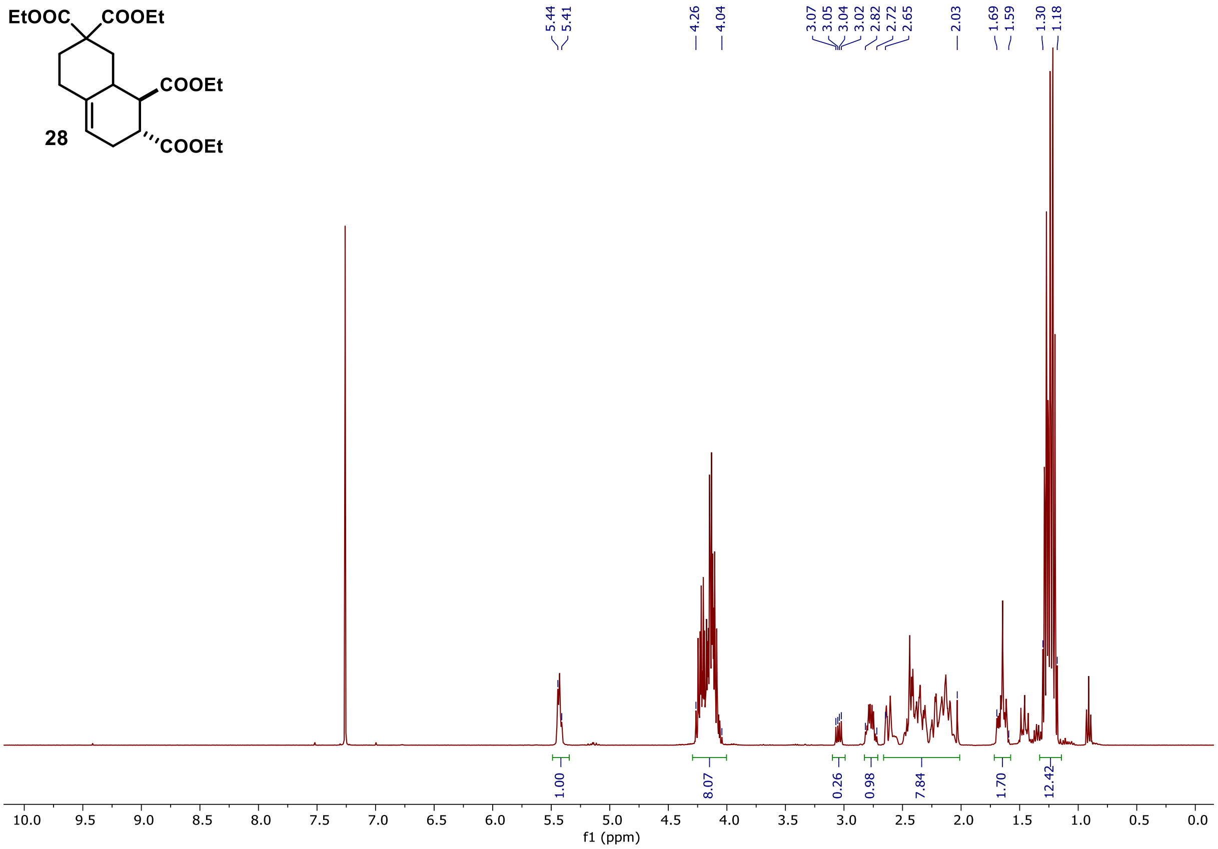

Figure S69: ^{1}H NMR spectrum of compound **28** (400 MHz, 298K, $CDCl_3$)

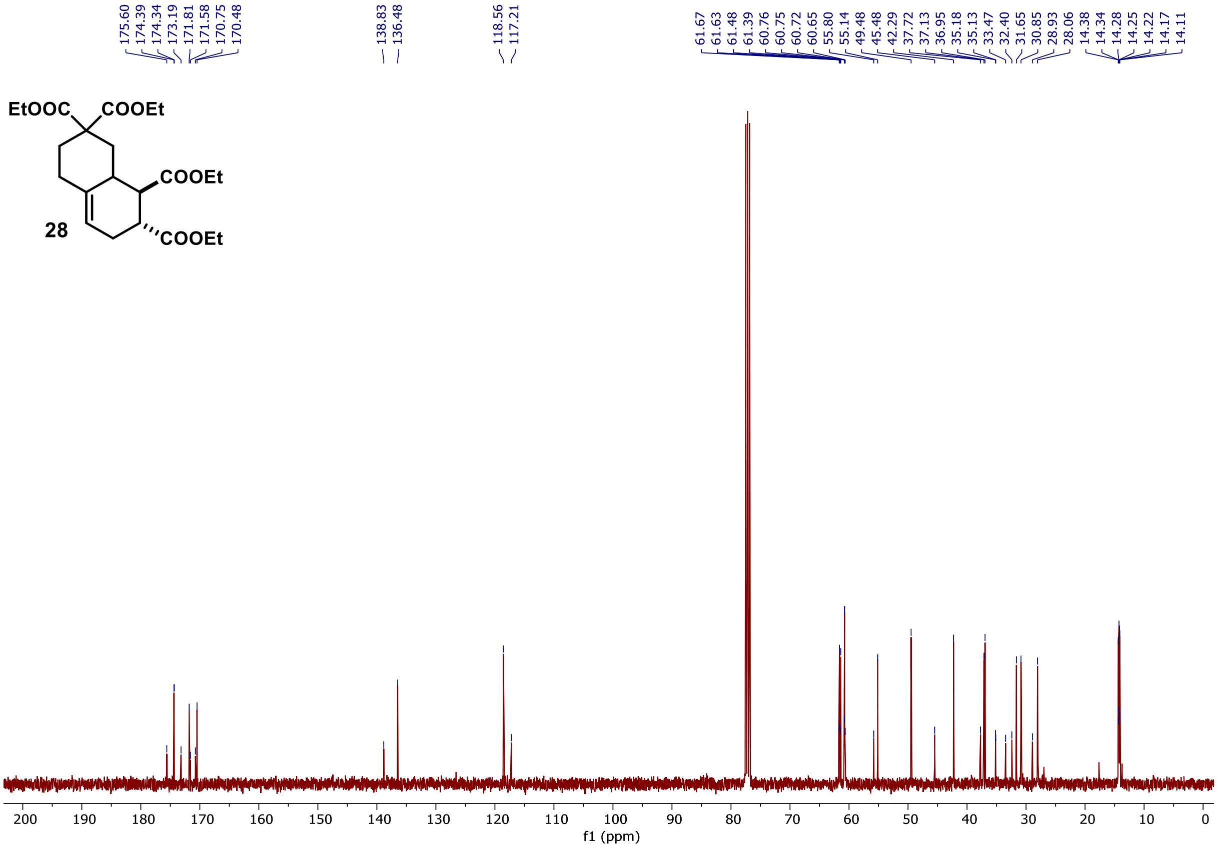

Figure S70: ^{13}C NMR spectrum of compound **28** (101 MHz, 298K, $CDCl_3$)

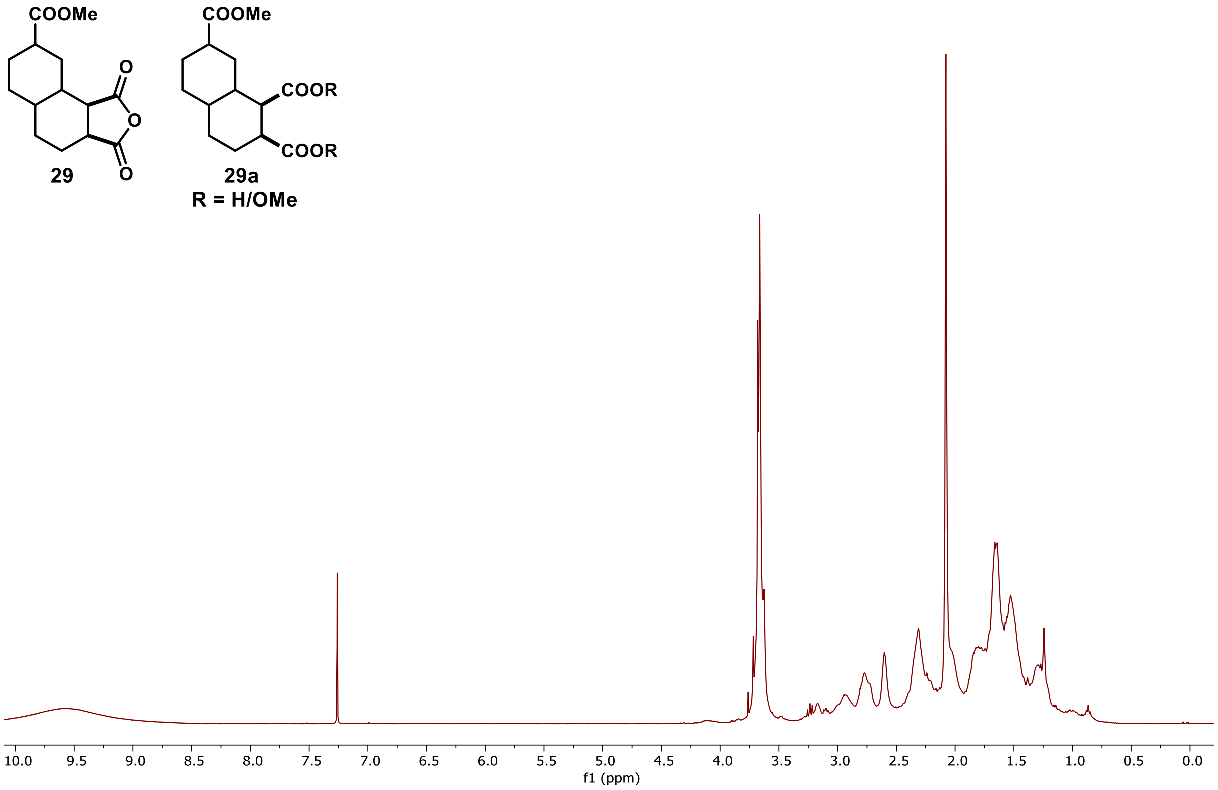

Figure S71: Crude ^{1}H NMR spectrum of compound **29/29a** (400 MHz, 298K, $CDCl_3$)

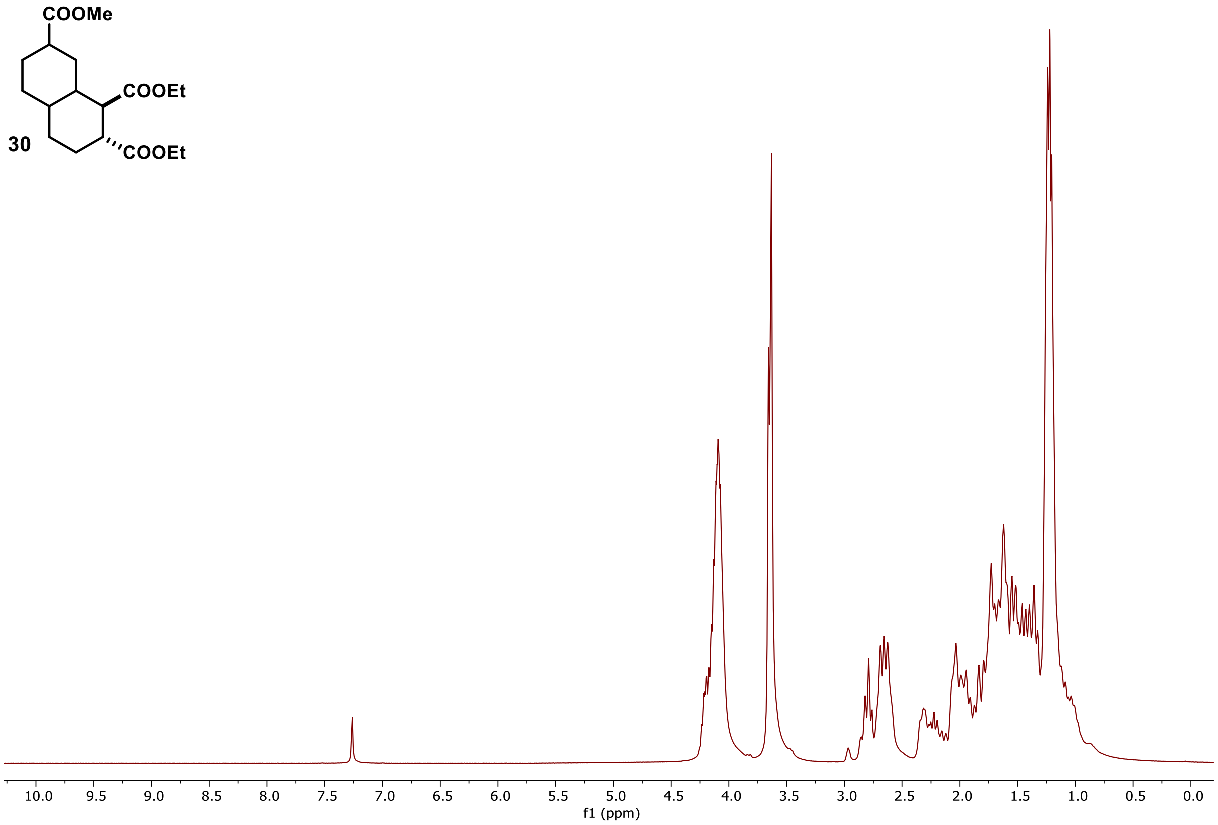

Figure S72: Crude ^{1}H NMR spectrum of compound **30** (400 MHz, 298K, $CDCl_3$)

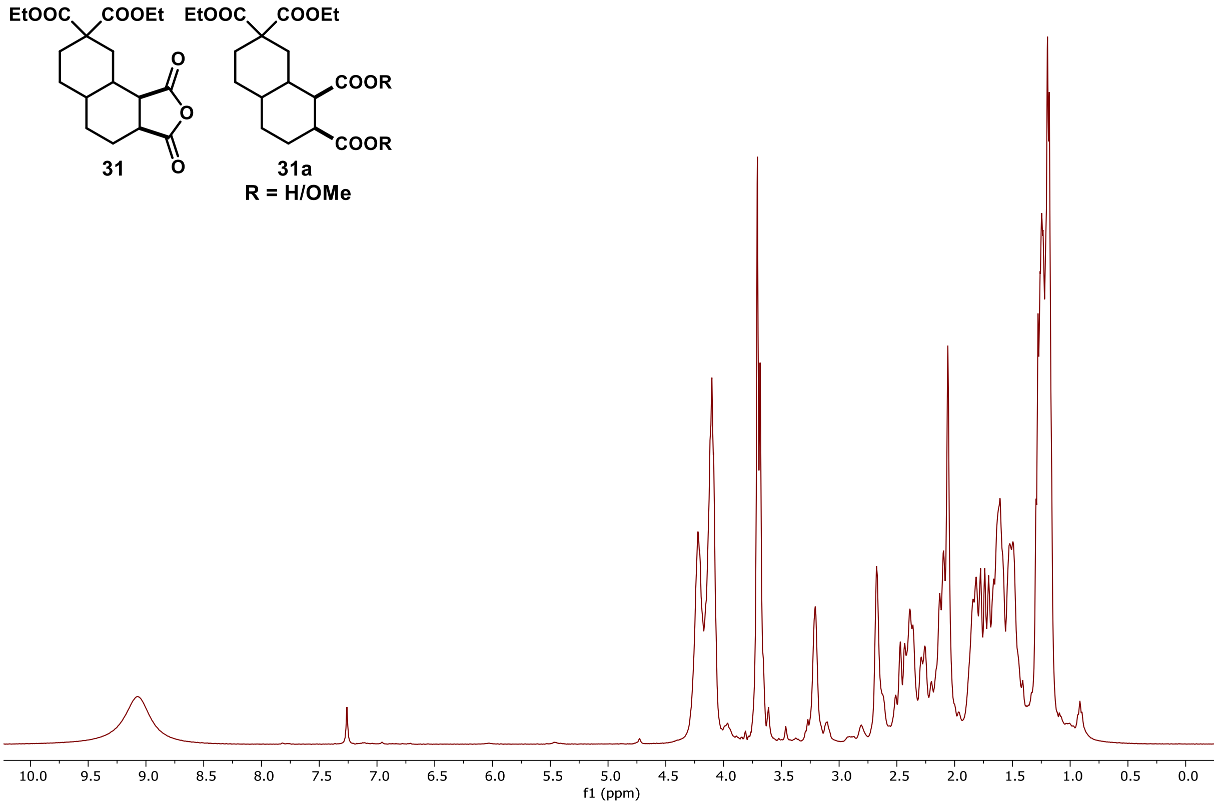

Figure 73: Crude ^{1}H NMR of compound **31** (400 MHz, 298K, $CDCl_3$)

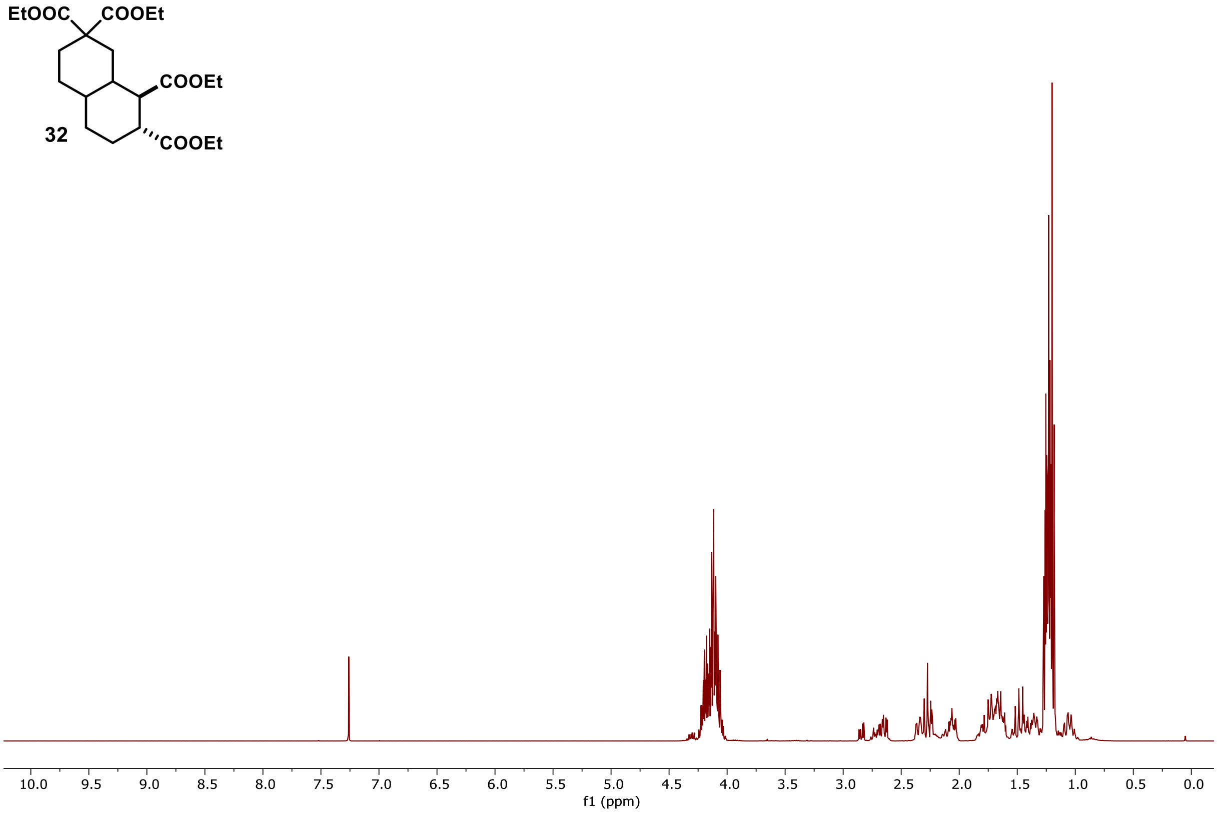

Figure 74: Crude ^{1}H NMR of compound **32** (400 MHz, 298K, $CDCl_3$)

Bibliography

(1) Rabal, O.; Sánchez-Arias, J. A.; Cuadrado-Tejedor, M.; de Miguel, I.; Pérez-González, M.; García-Barroso, C.; Ugarte, A.; de Mendoza, A. E.-H.; Sáez, E.; Espelosin, M. Design, Synthesis, Biological Evaluation and in Vivo Testing of Dual Phosphodiesterase 5 (PDE5) and Histone Deacetylase 6 (HDAC6)-Selective Inhibitors for the Treatment of Alzheimer's Disease. *Euro. J. Med. Chem.* **2018**, *150*, 506–524.

(2) Chen, X.; Ding, Y.; Duvadie, R.; Gai, Y.; Harrison, T.; Liu, Q.; Larrow, J.; Mao, J. Y. C.; Patel, S.; Ye, J.; Zecri, F.; Zheng, X.; Zheng, R.; Zhou, Y. Preparation of Cyclic Bridgehead Ether as DGAT1 Inhibitors. US8993619B2, October 31, 2013.

(3) Bradford, T. A.; Payne, A. D.; Willis, A. C.; Paddon-Row, M. N.; Sherburn, M. S. Practical Synthesis and Reactivity of [3] Dendralene. *J. Org. Chem.* **2010**, *75* (2), 491–494.

(4) Muratov, K.; Gagosz, F. Confinement-Induced Selectivities in Gold (I) Catalysis—The Benefit of Using Bulky Tri-(Ortho-biaryl) Phosphine Ligands. *Angew. Chem.* **2022**, *134* (28), e202203452.

(5) Hertkorn, N.; Benner, R.; Frommberger, M.; Schmitt-Kopplin, P.; Witt, M.; Kaiser, K.; Kettrup, A.; Hedges, J. I. Characterization of a Major Refractory Component of Marine Dissolved Organic Matter. *Geochim. Cosmochim. Acta.* **2006**, *70* (12), 2990–3010.

(6) Matsumoto, T.; Komatsu, S. Preparation of Tetracarboxylic Acids and Their Dianhydrides by Mechanochemical Alkali Decomposition of Tetraesters. JP2008201719, September 4, 2008.

(7) Senchyk, G. A.; Lysenko, A. B.; Boldog, I.; Rusanov, E. B.; Chernega, A. N.; Krautscheid, H.; Domasevitch, K. V. 1,2,4-Triazole Functionalized Adamantanes: A New Library of Polydentate Tectons for Designing Structures of Coordination Polymers. *Dalton Trans.* **2012**, *41*, 8675–8689.

(8) Talon, M.; Koornneef, M.; Zeevaart, J. A. D. Endogenous Gibberellins in Arabidopsis Thaliana and Possible Steps Blocked in the Biosynthetic Pathways of the Semidwarf Ga4 and Ga5 Mutants. *Proc. Natl. Acad. Sci. U. S. A.* **1990**, *87*, 7983.

(9) Hirotani, M.; Furuya, T.; Shiro, M. Studies on the Metabolites of Higher Fungi. Part 10. Cryptoporic Acids H and I, Drimane Sesquiterpenes from Ganoderma Neo-Japonicum and Cryptoporus Volvatus. *Phytochem.* **1991**, *30*, 1555.

(10) Ding, G.; Liu, Y.; Lu, Y.; Sheng, L. Two New Isomers of Chebulic Acid from Terminalia Chebula. *J. China Pharm. Univ.* **2001**, *32*, 333–335.

(11) Hu, X.; Mu, H.; Wang, Y.; Wang, Z.; Yan, J. Colorless Polyimides Derived from Isomeric Dicyclohexyl-Tetracarboxylic Dianhydrides for Optoelectronic Applications. *Polymer* **2018**, *134*, 8–19.

(12) Zherebker, A.; Kostyukevich, Y.; Kononikhin, A.; Kharybin, O.; Konstantinov, A. I.; Zaitsev, K. V.; Nikolaev, E.; Perminova, I. V. Enumeration of Carboxyl Groups Carried on Individual Components of Humic Systems Using Deuteromethylation and Fourier Transform Mass Spectrometry. *Anal. Bioanal. Chem.* **2017**, *409*, 2477–2488.

(13) Herges, R.; Reif, W. Photoresponsive Carboxylic Acids. *Liebigs Ann.* **1996**, 761–768.

(14) Ayats, C.; Camps, P.; Font-Bardia, M.; Munoz, M. R.; Solans, X.; Vazquez, S. Alternative Syntheses of the D2d Symmetric 1,3,5,7-Tetraiodotricyclo[3.3.0.03,7]Octane. *Tetrahedron* **2006**, *62*, 7436–7444.
